# During the Formation of Vasculogenic Mimicry by Melanoma Cells, the Silencing of Two Sets of Developmental Genes Is Coupled Either with an Increase or a Decrease in Contacts with the Nucleoli

**DOI:** 10.3390/ijms262311289

**Published:** 2025-11-22

**Authors:** Nickolai A. Tchurikov, Elena S. Klushevskaya, Viktoriya N. Lukicheva, Antonina N. Kretova, Elizaveta N. Poperekova, Vladimir R. Chechetkin, Galina I. Kravatskaya, Amalia A. Vartanian, Ildar R. Alembekov, Yuri V. Kravatsky

**Affiliations:** 1 Department of Epigenetic Mechanisms of Gene Expression Regulation, Engelhardt Institute of Molecular Biology Russian Academy of Sciences, Moscow 119334, Russia; 2 Department of Experimental Diagnosis and Therapy of Tumors, N.N. Blokhin National Medical Research Center of Oncology of the Ministry of Health of Russia, Moscow 115478, Russia

**Keywords:** rDNA clusters, RNA-seq, 4C-rDNA, vasculogenic mimicry, melanoma, epigenetics, development

## Abstract

Vasculogenic mimicry is the capacity of growing cancer cells to overcome the lack of normal capillaries and hypoxia by forming networks of sinuses lacking endothelial cells. The formation of vasculogenic mimicry by melanoma cells is coupled with the upregulation of the genes involved in ribosome biogenesis, the downregulation of hundreds of developmental genes, and strong changes in the inter-chromosomal contacts of developmental genes with rDNA clusters. The epigenetic mechanism driving the regulatory role of inter-chromosomal contacts of genes with nucleoli is not yet known. This study aimed to determine whether these contacts are associated with either the silencing or the activation of the expression of developmental genes. Here, two different sets of developmental genes are subjected to silencing either by increasing or decreasing their contacts with nucleoli. Our data indicate that one set of developmental genes mainly associated with system development is silenced by the contacts, while another set of genes involved mainly in the generation of neurons is silenced by the loss of contacts with the nucleoli. We conclude that mechanisms of silencing of these sets of developmental genes lead to the loss of the established differentiated state of melanoma cells and to the formation of more aggressive cancer cells. The data also indicate the important role of the nucleoli in the global regulation of gene expression during differentiation and cancer.

## 1. Introduction

Different mechanisms for the regulation of gene expression drive normal differentiation and cancer genesis. Nucleoli are involved in the global regulation of gene expression, although the particular mechanisms involved are not yet known [1,2,3]. Initially, numerous inter-chromosomal contacts of nucleoli were detected in *Drosophila*, and they were later more precisely mapped using the 4C (circular chromosome conformation capture) approach in different cell types [4,5,6,7,8]. Interestingly, the most prominent contacts corresponded to the genomic regions possessing developmental genes [6,7,9]. This finding has led to the study of the induced differentiation and malignization of human cells and the role of rDNA-contacting genes in these events [7,10,11].

The malignization of different solid tumors could occur through the formation of vasculogenic mimicry (VM) phenotypes [12,13]. The fast growth of tumors with increasing hypoxia could induce the generation of non-endothelial cell-lined channels delimited by an extracellular matrix [13,14]. Melanoma Mel Z cells, which are capable of forming VM when cultivated in 3D on Matrigel, were used for analyzing the transcriptome [10]. We found that the formation of VM phenotype leads to strong changes in gene expression patterns, including the upregulation of hundreds of genes involved in ribosome biogenesis and numerous genes controlling development.

In five human acrocentric chromosomes (13, 14, 15, 21, and 22), rDNA clusters are located and form nucleoli in each cell cycle. Recently, in a 4C experiment, the rDNA-contacting genes in Mel Z cells grown either in 2D on plastic or in 3D on Matrigel were identified [11]. Strong changes in inter-chromosomal contacts of nucleoli were detected during the formation of the VM phenotype. About 400 genes that retained stable contacts with nucleoli were involved in development and may control the initial stage of differentiation of Mel Z cells [11].

Here, we present the results of an analysis of rDNA-contacting genes that increased or decreased their contacts with nucleoli during the formation of the VM phenotype. Our data suggest that the silencing of different sets of rDNA-contacting developmental genes is associated either with an increase or a decrease in contacts with nucleoli during the establishment of the less differentiated state of aggressive cancer cells.

## 2. Results

### 2.1. A Total of 163 Downregulated Genes Had an Increased Number of Contacts with Nucleoli During the Formation of the VM Phenotype

The VM phenotype is formed by Mel Z cells while growing in 3D on Matrigel. In RNA-Seq experiments using Mel Z cells cultivated either on plastic or Matrigel, it was found that 1795 genes were downregulated while 976 were upregulated during the formation of the VM phenotype by Mel Z cells [10]. Recently, in 4C-rDNA experiments, we detected rDNA-contacted genes in cells growing either on plastic or Matrigel [11].

**Figure 1:**
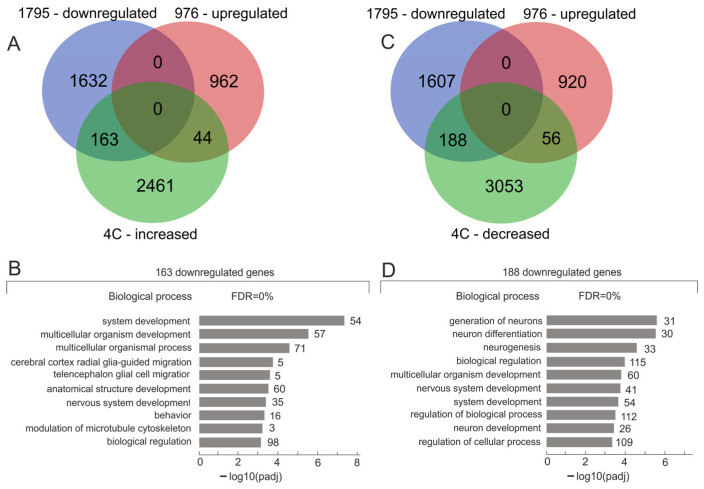
The identification of downregulated genes that changed their number of contacts with nucleoli during the formation of the VM phenotype. (**A**) The Venn diagram shows the intersections of downregulated and upregulated genes detected in Mel Z cells grown on Matrigel with genes whose number of contacts with nucleoli increased. Lists of the corresponding genes are presented in Table S2. (**B**) Top 10 Gene Ontology (GO) biological process associations of 163 genes using g:Profiler (https://biit.cs.ut.ee/gprofiler/gost, accessed on 15 August 2025). The values to the right of the bars show the number of genes associated with a process. A complete list of GO items and corresponding genes is shown in Table S3. (**C**) The Venn diagram shows the intersections of downregulated and upregulated genes detected in Mel Z cells grown on Matrigel with genes whose number of contacts decreased. Lists of the corresponding genes are presented in Table S4. (**D**) Top 10 Gene Ontology (GO) biological process associations of 188 genes using g:Profiler (https://biit.cs.ut.ee/gprofiler/gost, accessed on 15 August 2025). The values to the right of the bars show the number of genes associated with a process. A complete list of GO items and corresponding genes is shown in Table S5.

Now, to identify downregulated genes that achieved an increased number of contacts with nucleoli while growing on Matrigel, we used a Venn diagram. Figure 1A shows that at a threshold of FC 2.0 and a *p*-value = 0.05, 3297 genes had an increased number of contacts with nucleoli (Table S1). We found that during the formation of the VM phenotype, 163 genes were downregulated and had an increased number of contacts with nucleoli.

These genes, as expected from our previous data on rDNA-contacting genes, correspond to families of developmental genes. To conduct a detailed analysis, we searched Gene Ontology (GO) databases. The search performed using g:Profiler software, version 0.2.3, gprofiler version e113 eg59 p19 f6a03c19 (https://biit.cs.ut.ee/gprofiler/gost, accessed on 15 August 2025), using the “Highlight driver terms in GO” option [15], revealed that this group of genes is driven by “system development” (*p*-value = 3.98 × 10^−7^), “cell junction” (*p*-value = 3.25 × 10^−5^), and a “cytoskeleton” (*p*-value = 6.28 × 10^−4^) (Figure 1B and Figure S1). Among the top 10 Gene Ontology (GO) biological process associations with 163 genes are genes controlling cell migration (Figure 1B). The list of these 163 genes includes the tumor suppressor BRCA1, which also interacts with histone deacetylase complexes; the RUNX2 gene, a member of the RUNX family of transcription factors; the SETD5 gene, which may function as a histone methyltransferase; the NAV2 gene, derived from the neuron navigator gene family, which plays a role in cellular growth and migration; the NCKAP5 gene, which is involved in microtubule bundle formation and microtubule depolymerization; and seven ZNF genes.

This group of 163 genes is involved in several important pathways, including pathways in cancer, melanogenesis, the longevity regulation pathway, the cAMP signaling pathway, and some others (Figure S2).

**Figure 2:**
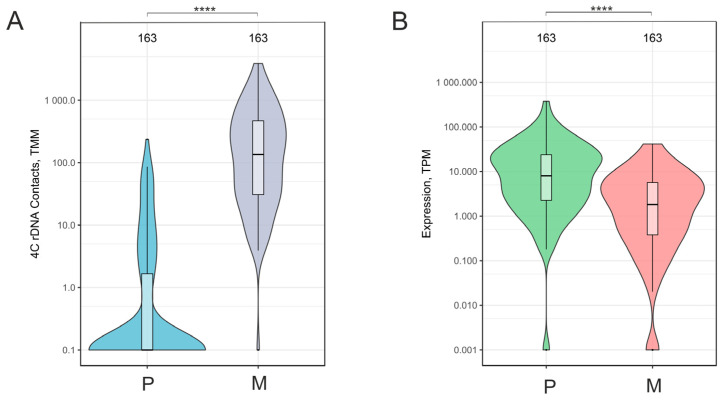
Violin plots of the contacts with nucleoli (**A**) and the expression (**B**) of the group of 163 downregulated genes in Mel Z cells cultivated either on plastic (P) or Matrigel (M). The number of corresponding rDNA-contacting genes are indicated at the top. These diagrams are related to Figure 1A,B and Table S6. TMM—trimmed means of M-values; TPM—Transcripts Per Million. **** *p*-values < 10^−6^.

Changes in the number of contacts with nucleoli and alterations in the expression of this group of downregulated genes occur to a great extent. Therefore, we used a logarithmic scale to visualize the rate of these changes. Violin presentations of these data are shown in Figure 2. Mostly, the genes in this group had no contacts or exhibited rare contacts with nucleoli while cells were cultivated on plastic (Figure 2A). In contrast, after 15 h of growing cells on Matrigel, these genes had considerably increased associations with nucleoli. These enhanced contacts with nucleoli coincided with the significant downregulation of the genes (Figure 2B). We conclude that the evidence strongly argues in favor of the possibility that contacts with nucleoli induced the concerted silencing of this group of 163 genes, which control system development, cell junction, and cytoskeleton.

### 2.2. Downregulated Genes Controlling System Development Had a Decreased Number of Contacts with Nucleoli During the Formation of the VM Phenotype

**Figure 3:**
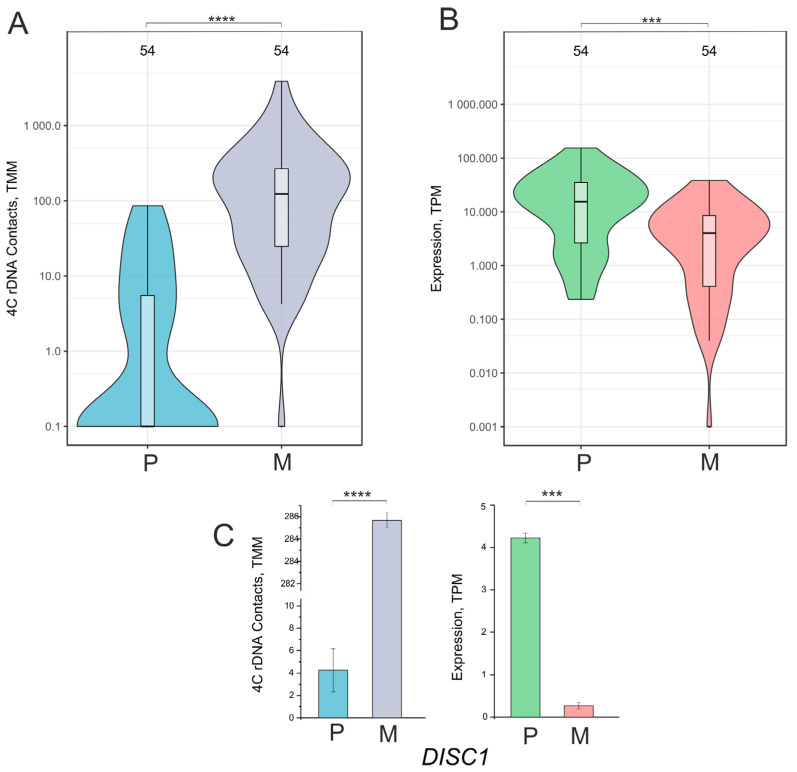
Violin plots of contacts with nucleoli (**A**) and the expression (**B**) of the 54 downregulated genes controlling system development in Mel Z cells cultivated either on plastic (P) or Matrigel (M). These diagrams are related to Figure 1A,B and Table S3. TMM—trimmed means of M-values; TPM—Transcripts Per Million. (**C**) Changes in contacts with nucleoli and the expression of the *DISC1* gene in the 54 genes controlling system development. **** *p*-values < 10^−6^; *** *p*-values < 10^−4^.

For 54 of these 163 genes controlling system development (see above), we performed separate analyses (Table S3). As expected, we observed that these genes also had greatly increased contacts with nucleoli and were subjected to silencing (Figure 3, Table S6). A significant number of these genes were not involved in inter-chromosomal contacts with nucleoli but acquired them after being grown on Matrigel (Figure 3A). These 54 genes were active in cells cultivated on plastic and were silenced after their transfer Matrigel (Figure 3B).

Among these genes are *WNT2B*, controlling cell growth and differentiation; *ANK3*, derived from a family of ankyrins, which play a key role in cell motility and proliferation; and *DISC1*, which plays an important role in neurogenesis and microtubule network formation. The gene also plays a role as a modulator of the AKT-mTOR signaling pathway. Figure 3C shows that this gene had dramatically increased contacts with nucleoli and was subjected to silencing.

These data independently support the conclusion that nucleoli may be involved in the silencing of rDNA-contacting genes.

### 2.3. A Total of 188 Downregulated Genes Controlling the Generation of Neurons Had a Decreased Number of Contacts with Nucleoli During the Formation of VM Phenotype

**Figure 4:**
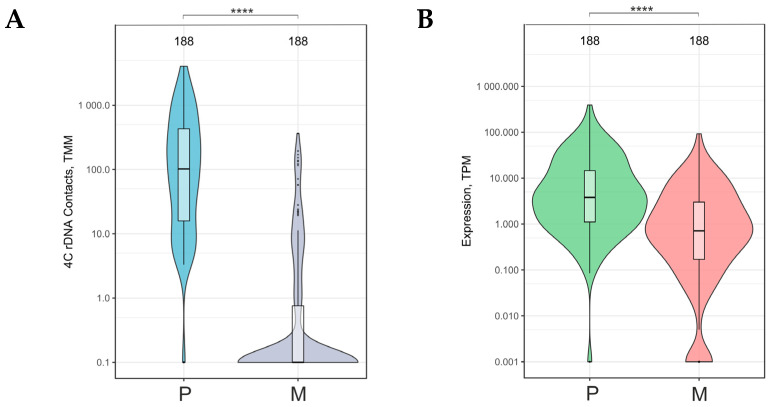
Violin plots of contacts with nucleoli (**A**) and the expression (**B**) of the 188 downregulated genes in Mel Z cells cultivated either on plastic (P) or Matrigel (M). The number of corresponding rDNA-contacting genes is indicated at the top. These diagrams are related to Figure 1C,D and Table S6. TMM—trimmed means of M-values; TPM—Transcripts Per Million. **** *p*-values < 10^−6^.

Next, we analyzed another group of downregulated genes: 188 genes detected based on the intersections between downregulated genes, upregulated genes, and rDNA-contacting genes that had a decreased number of contacts with nucleoli. The Venn diagram in Figure 1B,C shows the results. This group of rDNA-contacting genes lost almost all inter-chromosomal contacts with nucleoli when the cells were transferred to Matrigel (Figure 4A). This effect coincided with the silencing of this group of genes driven by the “generation of neurons” (*p*-value = 3.25 × 10^−5^), “biological regulation” (*p*-value = 1.1 × 10^−4^), and “cell junction” (*p*-value = 3.6 × 10^−4^) (Figure S3).

This group of 188 downregulated rDNA-contacting genes includes 10 *ZNF* genes, including *MAPK10*, a member of the kinase family that is involved in proliferation, differentiation, transcription regulation, and development. The MAPK10 kinase is specifically expressed in a subset of neurons in the nervous system, while *KLF7* and *KLF12*, members of the Kruppel-like transcriptional regulator family, regulate cell proliferation, differentiation, and some other key genes involved in differentiation (Table S5). We suppose that the downregulation of these developmental genes may be responsible for the formation of the less differentiated state and more aggressive behavior of Mel Z cancer cells, shaping the VM phenotype.

Figure S4 shows the pathways in which these 188 genes are involved. Among them are the cAMP signaling and MAPK pathways. Comparing cAMP pathways shown in Figures S2 and S4 revealed that 163 and 188 downregulated genes are involved in different branches of this pathway. We suppose that the concerted downregulation of these two big groups of developmental genes enabled via different mechanisms (either via increased or decreased inter-chromosomal contacts with nucleoli) hampers the efficiency of the same pathway.

### 2.4. Downregulated Genes Controlling the Generation of Neurons Had a Decreased Number of Contacts with Nucleoli During the Formation of the VM Phenotype

**Figure 5:**
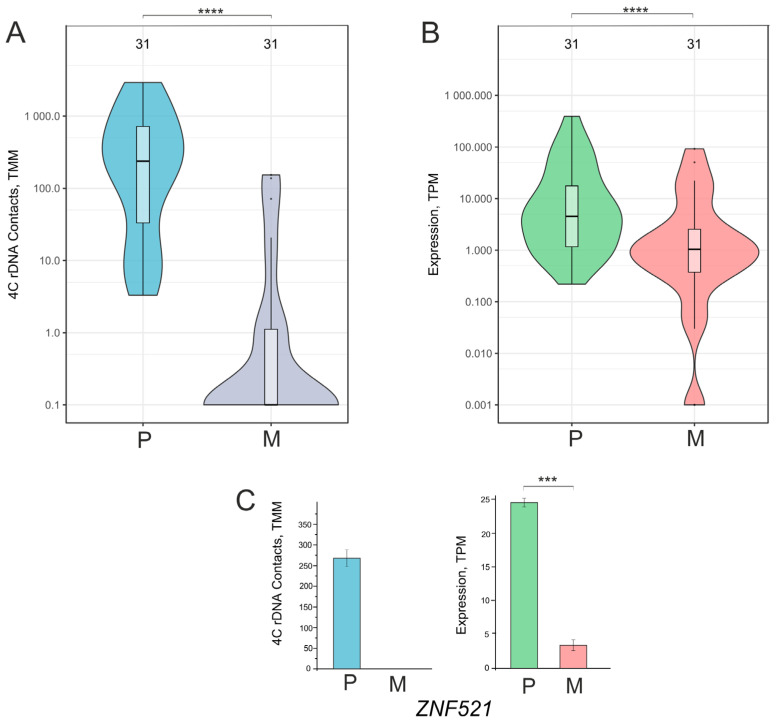
Violin plots of contacts with nucleoli (**A**) and the expression (**B**) of the group of 31 downregulated genes controlling the generation of neurons in Mel Z cells cultivated either on plastic (P) or Matrigel (M). These diagrams are related to Figure 1C,D and Table S6. TMM—trimmed means of M-values; TPM—Transcripts Per Million. (**C**) Changes in contacts with nucleoli and the expression of the *ZNF521* gene from the group of 31 genes controlling the generation of neurons. **** *p*-values < 10^−6^; *** *p*-values < 10^−4^.

A group of driver genes controlling the generation of neurons that originated from 188 downregulated (Figure 1D, Table S5) was used for the detailed analysis. Figure 5A shows that this group of 31 genes mostly possessed about 100 inter-chromosomal contacts with nucleoli, which were almost completely lost during the formation of the VM phenotype. At the same time, these genes were downregulated or completely silenced (Figure 5B). An analysis of one of these genes—*ZNF521*—is shown in Figure 5C. This gene specifies a transcription factor that can act as either an activator or a repressor, depending on the genomic context. The gene is highly associated with nucleoli and completely loses these inter-chromosomal interactions after the cultivation of cells on Matrigel, with this loss being accompanied by downregulation.

Among these 31 genes, only 8 are involved in the axon guidance pathway (Figure S5).

### 2.5. Concerted Downregulation of Two Big Groups of Developmental Genes Was Performed Simultaneously Using Different Mechanisms and Resulted in the Formation of Less Differentiated Cancer Cells

**Figure 6:**
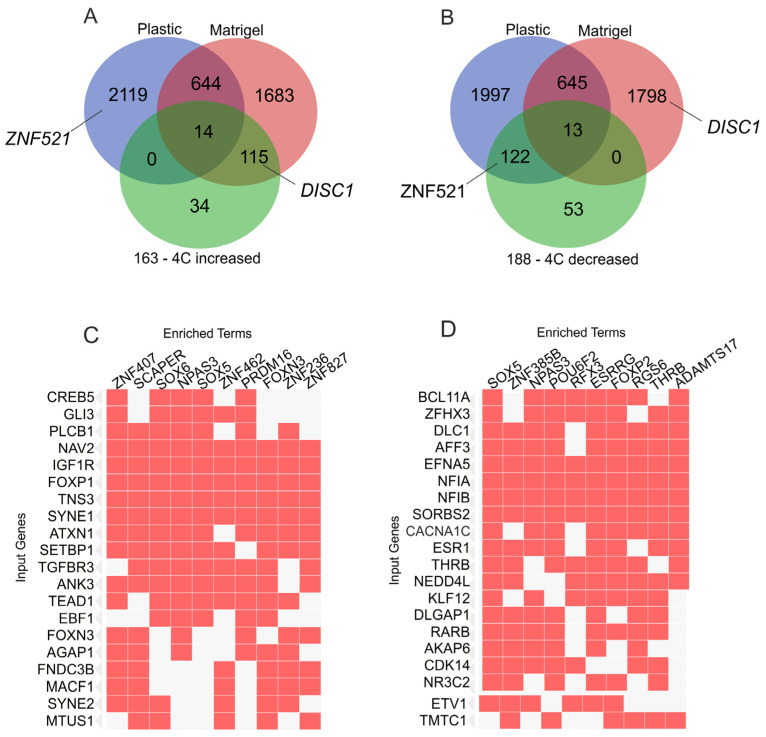
The properties of two groups of downregulated genes. The Venn diagrams show the intersections of rDNA-contacting genes possessing ≥ 30 contacts with nucleoli in Mel Z cells grown either on plastic or Matrigel with two groups of downregulated genes: 163 genes (**A**) and 188 genes (**B**). The positions of the *ZNF561* and *DISC1* genes described above are indicated. The corresponding data on intersections with 163 genes and 188 genes are shown in Tables S7 and S8, respectively. (**C**) A total of 163 genes are simultaneously regulated by different transcription factors (“enriched terms”). The top 20 genes are shown (“input genes”). The data were obtained by searching for corresponding genes in Enrichr Submissions TF-Gene Cooccurrence (https://maayanlab.cloud/Enrichr, accessed on 14 October 2025). A complete list of the corresponding TFs is shown in Table S9. (**D**) A total of 188 genes are simultaneously regulated by different transcription factors (“enriched terms”). The top 20 genes are shown (“input genes”). The data were obtained by searching for corresponding genes in Enrichr Submissions TF-Gene Cooccurrence (https://maayanlab.cloud/Enrichr, accessed on 14 October 2025). A complete list of the corresponding TFs is shown in Table S10.

We observed that both increasing and decreasing inter-chromosomal contacts of developmental genes with nucleoli coincided with the downregulation of corresponding genes. Figure 6 summarizes the relationships between rDNA-contacting genes possessing ≥ 30 contacts with nucleoli in Mel Z cells cultivated either on plastic or Matrigel and two groups of downregulated genes, which contained 163 genes and 188 genes, respectively. This makes it clear that most of the 163 genes had their contacts increased by up to ≥30 interactions (Figure 6A). This event may have caused the genes’ downregulation while the cells were cultivated on Matrigel. This finding reflects the potential silencing capacity of nucleoli.

On the other hand, most of the 188 genes had decreased contacts with nucleoli while cultivated on Matrigel and were subjected to silencing (Figure 6B). It may follow that nucleoli were involved in the upregulation of these 188 genes when the cells were cultivated on plastic, as the loss of these interactions during growth on Matrigel led to the repression of these genes.

Previously, it was found that rDNA-contacting genes are simultaneously regulated by different transcription factors [7,9,10,11]. These two big groups of downregulated genes are associated with multiple transcription factors (Figure 6C,D). Tables S9 and S10 show that 543 and 906 transcription factors regulate the groups of 163 and 188 genes, respectively. These lists of transcription factors are essentially overlapping. It is known that different transcription factors are bifunctional and act as activators or repressors depending on the chromatin level. This is true for many elements, such as the ZNF521 factor from the group of genes controlling the generation of neurons. Interestingly, these sets of non-overlapping genes have two of the top ten transcription factors in common—SOX5 and NPAS3 (Figure 6C,D). Normally, these factors are associated with the activation of transcription. Here, we detected the downregulation of rDNA-contacting genes induced by Matrigel and the possibility for the cells to grow in 3D. These data indicate that when Mel Z cells were cultivated on plastic, these two groups of genes were active (Figure 2B and Figure 4B).

## 3. Discussion

### 3.1. Matrigel Induces an Epigenetic Switch in Mel Z Cells

Widespread epigenetic mechanisms are triggered in response to different internal or external signals that induce rather quick alterations in gene expression patterns in a cell during differentiation or cancer genesis. We used a model of melanoma Mel Z cells, which can form the VM phenotype during 9–15 h of cultivation on Matrigel [10]. Matrigel is used as a matrix for 3D cell cultures and has the capacity to alter cellular phenotype and gene expression in different cancer cells that form the VM phenotype, including Mel Z cells [10,16,17,18,19,20]. Cultivation on Matrigel induces several signaling pathways. It was reported recently that at some stage during the formation of the VM phenotype, the AKT pathway is activated, promoting the proliferation and migration of cancer cells [21,22]. Among the rDNA-contacting genes analyzed in this study, we detected the *DISC1* gene, which is a type of system development gene and acts as a modulator of the AKT-mTOR signaling pathway. We also found that upon cultivating Mel Z cells on Matrigel, two big groups of developmental rDNA-contacting genes are associated with several other pathways—cAMF signaling, melanogenesis, longevity regulation, MAPK signaling, GnRH signaling, cholinergic synapse, and Cushing syndrome (Figures S2 and S4). It should be emphasized that Matrigel only mimics in vivo VM formation. Studying the different types of cancer cells that form this phenotype could uncover the crucial underlying mechanisms. Currently, we perform such searches to understand the role of nucleoli in the formation of the VM.

It was demonstrated that about 400 developmental genes stably retain contacts with nucleoli in Mel Z cells grown on Matrigel [11]. We assume that these genes are likely involved in maintaining the achieved state of differentiation in Mel Z cells. Our data indicate that the changes in the number of inter-chromosomal contacts between numerous rDNA-contacting genes and nucleoli lead to the silencing of these groups of developmental genes. It follows that Mel Z cells grown on Matrigel and forming the VM phenotype should become less differentiated and more aggressive cancer cells.

### 3.2. How Could Nucleoli Downregulate or Upregulate Developmental Genes?

Our data strongly indicate that the interactions with nucleoli are associated with the regulation of numerous developmental genes. It was suggested that these contacts could be associated with both activation or repression of corresponding genes [2,3], as well as that nucleoli could organize multiple micro-drops around them, with these micro-drops possessing either repressors or activators in particular regions around this membrane-less the largest nuclear organelles [11]. Our data confirm the supposition that nucleoli are associated with both the activation and repression of the contacting genes. Previously, it was demonstrated that more frequent contacts between the genes controlling development, anatomical structure development, and cell morphogenesis and the nucleoli led to their silencing [11].

It was found that rDNA-contacting genes are co-expressed with numerous miRNA species [11] and simultaneously regulated by hundreds of transcription factors in different combinations (Figure 6, Tables S9 and S10). It is known that there is an interplay between miRNA expression, DNA methylation, and heterochromatin formation [23]. Previously, it was found that the same set of about five hundred developmental genes from three different human cell lines frequently shape contacts with nucleoli, and about one hundred of these common genes are highly associated with silencing by the H3K27me3 mark, which is involved in the formation of facultative heterochromatin [24]. rDNA-contacting regions often possess rather long stretches of H3K27ac mark—a marker for active enhancers [4]. All these data taken together with the data on co-regulation with hundreds of transcription factors strongly support the hypothesis suggesting that nucleoli, using mechanisms of phase separation, shape multiple micro-drops around them. These micro-drops possess hundreds of either activators or repressors of transcription [11]. Now, we test this supposition experimentally.

### 3.3. How Could Dynamic Inter-Chromosomal Contacts with Nucleoli Be Formed?

Currently, we do not know what particular mechanisms are involved in the establishment of the dynamic long-range inter-chromosomal interactions of different chromosomal regions with nucleoli. How is a particular chromosomal region targeted to the nucleoli? One possible means of achieving precise navigation to the nucleoli is a mechanism mediated via non-coding RNAs. Potentially, RNA molecules could readily recognize any genomic region based on nucleotide complementarity to a region. Pervasive transcription could provide a cell with RNA replicas of the entire genome [25,26,27,28]. These RNA molecules could serve as navigators in the nucleus and cytoplasm. It was found that rDNA-contacting genes are co-expressed with hundreds of lincRNAs and that there is a strong correlation between the genomic positions of rDNA-contacting genes and particular lincRNAs [29]. These data suggest that corresponding lincRNAs act as navigators that connect chromosomal regions to nucleoli.

Another possibility is a protein-mediated mechanism of navigation. It was demonstrated that ZNF274 anchors target DNA sequences at the nucleolus and facilitates their compartmentalization [30,31]. New experiments are required to further elucidate this point. Currently, we study one genomic region in frequent contact with the nucleoli in different cell types and the properties of a lincRNA coming from this region.

## 4. Materials and Methods

### 4.1. Mel Z Cell Culture

The cells were obtained from the N.N. Blokhin National Medical Research Center at the Oncology Department of the Ministry of Health, Russia [10], and were initially propagated on a plastic surface in an RPMI-1640 medium supplemented with 10% fetal calf serum, 2 mM of glutamine, and 0.1% gentamicin sulfate at 37 °C in a humidified atmosphere containing 5% CO_2_. Up to 10 million cells with 70–75% confluency were divided into two equal samples and seeded either on plastic or Matrigel. They were cultivated under identical conditions for 15 h; then, the medium above the cells was removed, and the cells cultivated on plastic and Matrigel were liberated through 20 min of incubation at 37 °C in 5 mL of Versene solution in Dulbecco’s phosphate-buffered saline containing 0.05% trypsin. The cells were then used for 4C experiments and RNA isolation. Petri dishes coated with Matrigel (BD Bioscience, Bedford, MA, USA) were prepared as follows: the Matrigel (8.7 mg/mL) was thawed at 4 °C, and 6 mL was quickly added to each 10 cm dish and allowed to solidify for 30 min at room temperature; then, it was placed in a humidified 5% CO_2_ incubator for 1 h at 37 °C. The RNA-Seq analysis process for cells grown on plastic and Matrigel was previously described in [10].

### 4.2. 4C-rDNA Procedure

4C-rDNA experiments were performed according to the procedures previously described in [9,11]. Mel Z cells were fixed in 1.5% formaldehyde, and the nuclei were isolated. Then, digestion with an EcoRI enzyme and the ligation of extensively diluted DNA to favor intramolecular ligations were performed. To shorten the ligated DNA fragments, digestion with FaeI endonuclease was performed, followed by the ligation of diluted DNA samples to favor circularization and minimize dimerization. The 4C-rDNA raw data were deposited in the GEO database under accession number GSE295545.

### 4.3. 4C Mapping and Processing

The processing of 4C-rDNA-associated reads was performed as previously described [11]. Adapter trimming was carried out separately for forward (R1) and reverse (R2) reads using Cutadapt v4.9 [16]. BBTools [32] version 39.01 repair.sh script was employed to re-pair forward and reverse reads using the “repair” option, while all singletons were excluded from further analysis.

Paired-end filtered reads were aligned to the GRCh38/hg38 genome (https://ftp.ensembl.org/pub/release-112/fasta/homo_sapiens/dna/, accessed on 13 November 2025) using bwa 0.7.17-r1188 [33] with the mem algorithm. Post-alignment processing of BAM files was performed using SAMtools v1.17 [34], filtering out unaligned reads and supplementary alignments (-F 2052) and sorting alignments by coordinate (samtools sort). Alignments entirely overlapping low-complexity genomic regions from the Dfam v3.8 database [35] were removed using BEDTools v2.31.0 [36] (intersect -wa -v -f 1.0), ensuring that only alignments fully contained within low-complexity regions were filtered out.

deepTools2 v3.5.5 [37] was employed for the quality control of replicates.

Aligned 4C-rDNA-associated reads were intersected across replicates using an in-house R script employing the data.table v1.15.2 [38] and dplyr 1.1.4 [39] libraries, with only intersecting alignments retained for downstream analysis.

Gene quantification was performed using featureCounts v2.0.6 [40] with the following parameters: -a hg38.112.gtf -t gene -g gene_id, -O -p –countReadPairs --fraction --readExtension5 2500, and --readExtension3 2500; these parameters reflected the ±2.5 kb resolution of the six-cutter EcoRI restriction enzyme used in the 4C-rDNA experiments.

For downstream Gene Ontology (GO) analysis, gene IDs were mapped to ISO gene names using an in-house R script employing the dplyr v.1.1.4 R [39] package.

### 4.4. Violin Plots for 4C-rDNA Data

Because direct comparison of datasets with varying read depths is not appropriate, normalization was performed using the trimmed mean of M-values (TMM) method [41], which was implemented using the edgeR v3.40.2 [42] R package. This approach normalized gene counts across all datasets, enabling direct comparison of 4C contacts associated with genes between different experiments.

Violin plots were generated from TMM-normalized data using R scripts that utilized the dplyr v1.1.4 [39] and ggplot2 v3.5.0 [43] R packages. The non-parametric, independent, two-group Mann–Whitney U-test was applied to assess whether different subsets of 4C-rDNA-associated genes originated from the same distribution. Obtained *p*-values were adjusted for multiple comparisons using Holm’s most stringent family-wise error rate (FWER) correction [44] via the standard pairwise.wilcox.test() function in R. This analysis confirmed that in each pairwise comparison, the subsets did not originate from the same distribution (*p* << 1 × 10^−20^), indicating their statistical independence.

### 4.5. Violin Plots for Gene Expression DATA

Deep sequencing gene expression data for both plastic-grown and Matrigel-grown cells were obtained from the GEO database under accession numbers GSE221876 and GSE221872, respectively. The processing of the raw data to TPM (Transcripts Per Million) values was described in detail previously in [10]. Gene lists for each functional category were obtained using the g:Profiler functional enrichment analysis web server [17].

Violin plots were generated from TPM-normalized data using an in-house R script incorporating the dplyr v1.1.4 [39], ggplot2 3.5.0 [43], and tidyR 1.3.1 [45] R packages.

To assess whether gene expression datasets corresponding to different gene lists originated from the same distribution, a non-parametric Mann–Whitney U test was applied. The resulting *p*-values were adjusted for multiple comparisons using Holm’s family-wise error rate (FWER) correction [36], as implemented in the standard pairwise.wilcox.test() function in R. The performed assessment validated that in each pairwise comparison, the subsets did not originate from the same distribution (*p* << 1 × 10^−20^), indicating their statistical independence.

### 4.6. Code Accessibility

All scripts and appropriate data files have been deposited in a public GitHub repository (https://github.com/lokapal/MelZ.4C.2025, accessed on 13 November 2025).

## Data Availability

4C-rDNA data were deposited in the Gene Expression Omnibus (GEO) repository under accession number GSE295545. RNA-Seq data were deposited in the GEO repository under accession numbers GSE221876 and GSE221872.

## Supplementary Materials

The following supporting information can be downloaded at https://www.mdpi.com/article/10.3390/ijms262311289/s1.
