# Supplementary material for: During the Formation of Vasculogenic Mimicry by Melanoma Cells, the Silencing of Two Sets of Developmental Genes Is Coupled Either with an Increase or a Decrease in Contacts with the Nucleoli
Source: Int J Mol Sci. 2025 Nov 22;26(23):11289. doi: 10.3390/ijms262311289 (PMC12692539; doi:10.3390/ijms262311289)
Supplement: Supplementary file 1 [file ijms-26-11289-s001.zip › Supplemental Information.pdf]

**-Supplemental Information for:**

# During formation of vasculogenic mimicry by melanoma cells the silencing of two sets of developmental genes is coupled either with increase or decrease of the contacts with nucleoli

**Nickolai A. Tchurikov, [1*] Elena S. Klushevskaya [1], Viktoriya N. Lukicheva [1], Antonina N. Kretova [1], Elizaveta N. Poperekova [1], Vladimir R. Chechetkin [1], Galina I. Kravatskaya [1], Amalia A. Vartanian [2], Ildar R. Alembekov [1], and Yuri V. Kravatsky [1]**

[1] Department of Epigenetic Mechanisms of Gene Expression Regulation, Engelhardt Institute of Molecular Biology Russian Academy of Sciences, Moscow, 119334, Russia
[2] Department of Experimental Diagnosis and Therapy of Tumors, N.N. Blokhin National Medical Research Center of Oncology of the Ministry of Health of Russia, Moscow, 115478, Russia
*Correspondence: tchurikov@eimb.ru

**This PDF file includes:**

Figures S1-S5

Tables S1–S10

| ID | Source | Term ID | Term Name | $p_{adj}$ (query_1) |
|---|---|---|---|---|
| 1 | GO:BP | GO:0048731 | system development | $3.980\times10^{-7}$ |
| 2 | GO:BP | GO:0022030 | telencephalon glial cell migration | $1.038\times10^{-3}$ |
| 3 | GO:BP | GO:0040007 | growth | $4.241\times10^{-2}$ |
| 4 | GO:CC | GO:0030054 | cell junction | $3.247\times10^{-5}$ |
| 5 | GO:CC | GO:0005856 | cytoskeleton | $6.275\times10^{-4}$ |
| 6 | GO:CC | GO:0099081 | supramolecular polymer | $3.870\times10^{-2}$ |

**Figure S1.** Gene Ontology driver terms for 163 downregulated genes. The search was performed using Gene Ontology driver terms (https://biit.cs.ut.ee/gprofiler). Related to Figure 1B.

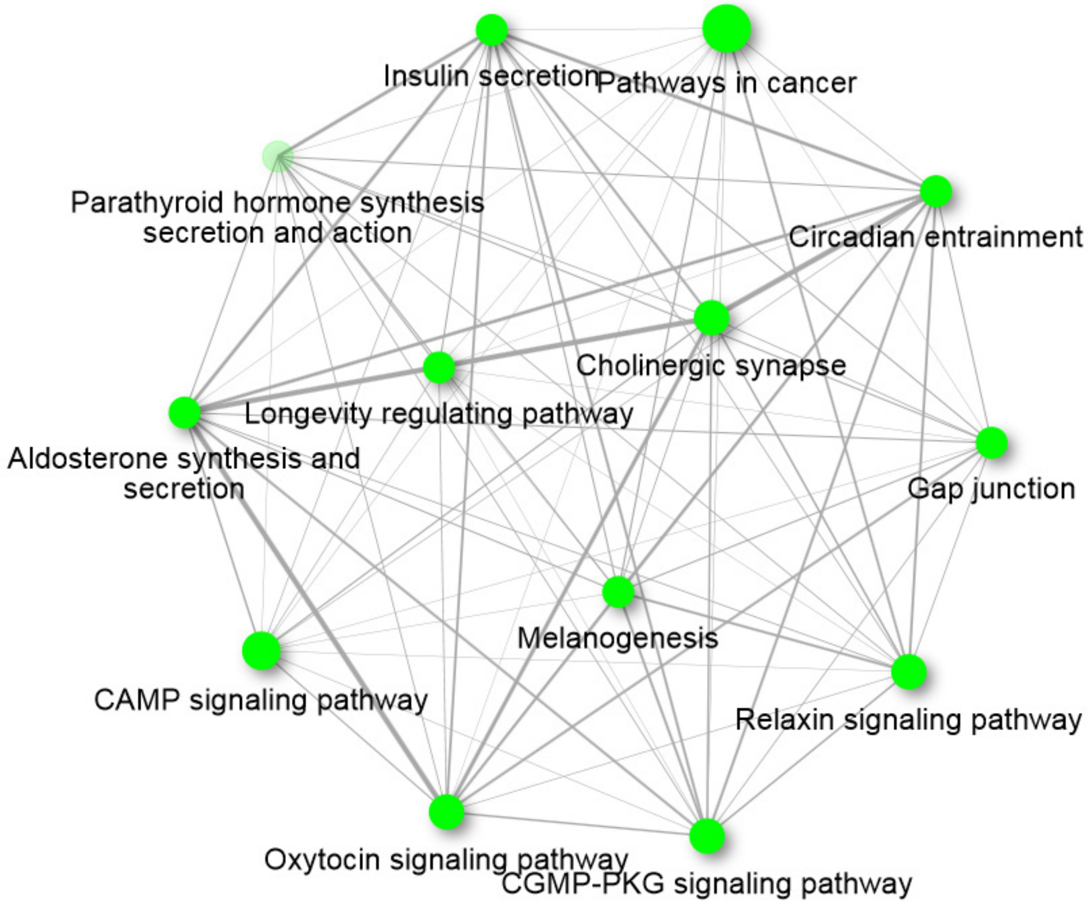


**Figure S2.** KEGG pathways associated with 163 downregulated genes. The search was performed using GO search in **ShinyGO 0.80** (https://bioinformatics.sdstate.edu/go80/).

| ID | Source | Term ID | Term Name | $p_{adj}$ (query_1) |
|---|---|---|---|---|
| 1 | GO:MF | GO:0140110 | transcription regulator activity | $6.184\times10^{-4}$ |
| 2 | GO:MF | GO:0043167 | ion binding | $1.538\times10^{-3}$ |
| 3 | GO:MF | GO:0000978 | RNA polymerase II cis-regulatory region sequence... | $2.619\times10^{-3}$ |
| 4 | GO:MF | GO:0005516 | calmodulin binding | $1.353\times10^{-2}$ |
| 5 | GO:MF | GO:0106310 | protein serine kinase activity | $4.089\times10^{-2}$ |
| 6 | GO:BP | GO:0048699 | generation of neurons | $4.016\times10^{-5}$ |
| 7 | GO:BP | GO:0065007 | biological regulation | $1.094\times10^{-4}$ |
| 8 | GO:BP | GO:0141193 | nuclear receptor-mediated signaling pathway | $5.510\times10^{-4}$ |
| 9 | GO:BP | GO:1901701 | cellular response to oxygen-containing compound | $6.353\times10^{-3}$ |
| 10 | GO:BP | GO:0007154 | cell communication | $1.166\times10^{-2}$ |
| 11 | GO:BP | GO:0003300 | cardiac muscle hypertrophy | $3.365\times10^{-2}$ |
| 12 | GO:CC | GO:0030054 | cell junction | $3.581\times10^{-4}$ |
| 13 | GO:CC | GO:0098590 | plasma membrane region | $1.614\times10^{-2}$ |

**Figure S3**. Gene Ontology driver terms for 188 downregulated genes. The search was performed using Gene Ontology driver terms (https://biit.cs.ut.ee/gprofiler). Related to Figure 1D.

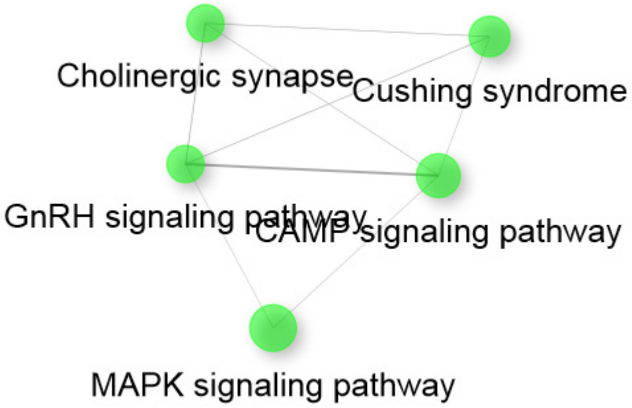


**Figure S4.** KEGG pathways associated with 188 downregulated genes. The search was performed using GO search in **ShinyGO 0.80** (https://bioinformatics.sdstate.edu/go80/).

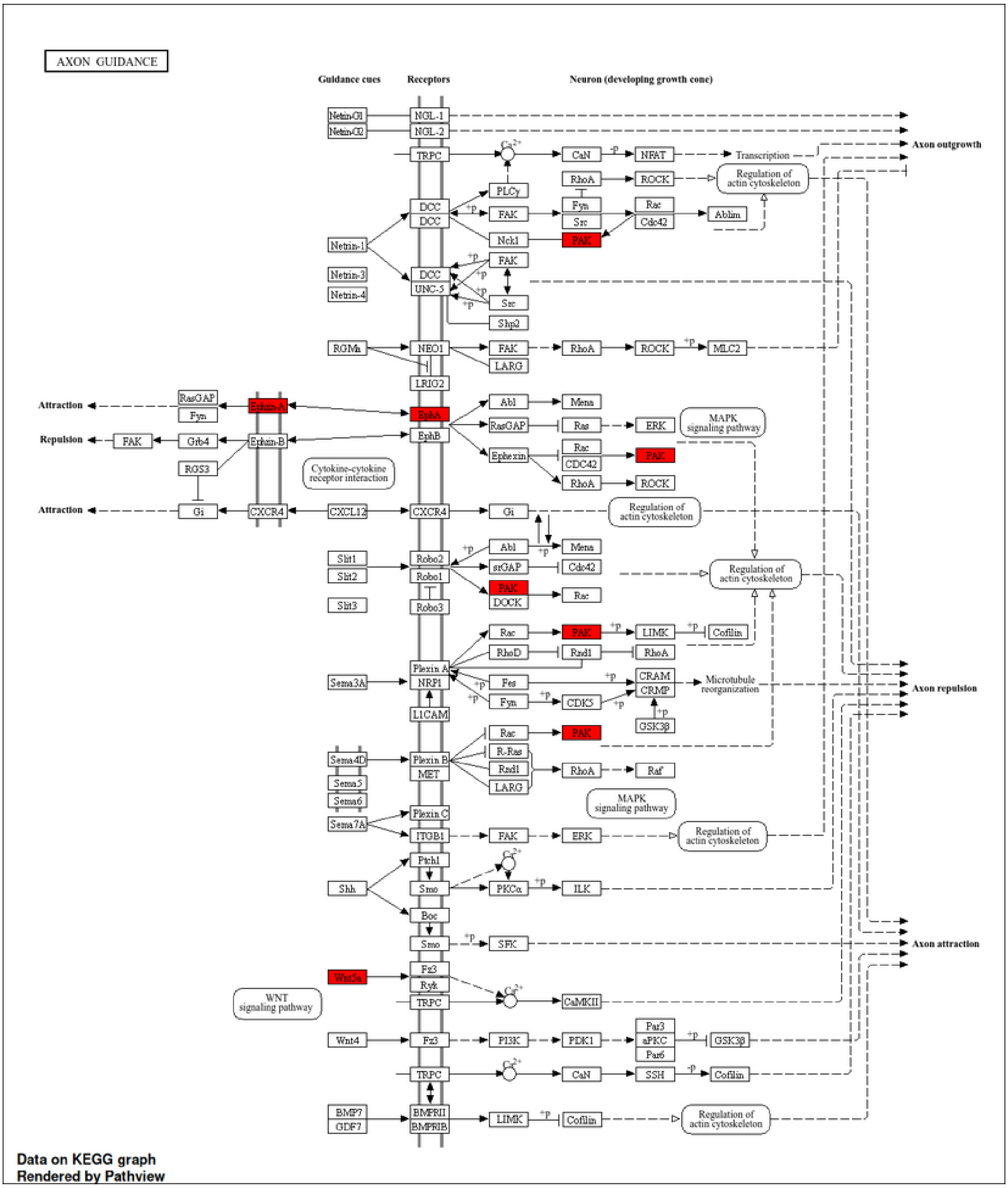


Your genes are highlighted in red. Downloading pathway diagram from KEGG can take 3 minutes.

**Figure S5.** KEGG pathways associated with 31 downregulated genes. The search was performed using GO search in **ShinyGO 0.80** (https://bioinformatics.sdstate.edu/go80/).

## Tables S1–S10

**Table S1**. List of rDNA-contacting genes in Mel Z cells grown either on plastic or on Matrigel. 4C-rDNA reads were processed as described in the Materials and Methods section. The Excel file is attached separately.

**Table S2**. Venn diagram showing the intersections of downregulated and upregulated genes detected in Mel Z cells grown on Matrigel with genes whose number of contacts with nucleoli were increased. Related to Figure 1A.

| Names | total | elements |
|---|---|---|
| 1795 down 4C incr-2684 | 163 | *NHLRC3 ENSG00000286540 TNS3 TEAD1 BRCA1 ELAPOR2 TOX3 RALY-AS1 CDC42BPA FAM218A ENSG00000286830 SLC46A2 R3HDM2 FBXL20 CHD1-DT TTC28-AS1 SRGAP2C PPIEL ZFHX2 NR2F2-AS1 SETBP1 ADCY1 GRID1 ANO4 FOXN3 KC6 LINC00589 SRGAP2B DNPEP-AS1 SRGAP1 ARHGEF35-AS1 SYNE1 KRT89P PDZD2 RUNX2 WEE2-AS1 ACOXL FNDC3B FHIP1A NBPF14 LINC00663 ZNF519 MACF1 MEF2C-AS1 STARD4-AS1 TFDP2 LRRC37A2 ANKRD28 ZNF609 KTN1-AS1 RBMS2 TSHZ2 EOLA2-DT ZNF516 ZEB1-AS1 EDNRB SFMBT2 SETD5 EBF1 SMPX FCHSD2 SLC4A7 MTUS1 CPED1 KLHL32 TMEM100 CACNA1D IVNS1ABP TMEM150C INPP4B COL4A4 ANKFN1 IGF1R NLGN1 LINC00342 WNT2B ENSG00000286248 AZIN2 Y_RNA ANKRD27 ANK3 GTF2IRD1 ZNF19 TEX21P HIP1 FBXO32 ARHGEF10 PTPN13 VAV3 LINC01252 APBA1 CYP39A1 LRRC37A4P MBD5 ZNF320 STON1 DISC1 FMN1 ZFPM2 ZNF827 DSE NAV2 RNF150 GNG2 FRS3 ACKR2 MCTP2 ZFPM2-AS1 BEND5 SSBP2 ARHGAP32 SPRY4-AS1 CSGALNACT1 PABIR3 BAZ2B CAPS2 CAMK4 PCYOX1L GLI2 TGFBR3 ATXN1 S100PBP PLEKHG1 PLEKHA5 COL27A1 PHLDB2 ESR2 MOB3B RASSF3 SYNE2 COL15A1 CBLB ZNF221 SRGAP2 CNTNAP4 TUBA3D PLEKHH2 GLI3 ENSG00000287729 GAB2 CREB5 ADGRG6 EPB41 DOCK9 PPARA NCKAP5 MAP2K5 PLCB1 EDA CNTRL CNTNAP3 RBM47 GNAO1-DT SEMA3D FOXP1 SCN8A ZRANB2-DT AGAP1 RIPOR2 PARD3B HSPG2 FRMD4B GOLGA8B* |
| 4C incr-2684 976 up | 44 | *EOLA2 DUS3L METTL25 ACTR5 EIF4A3 H2BC18 CYP19A1 SCRN1 ZBTB7C AP1S3 SHQ1 FASTKD5 TIMM23 CKMT1A BRMS1L STAMBPL1 UTP15 UNC5B PRDX1 OLFM1 SRP68 NOP16 NUP153-AS1 HSPH1 BAK1 CACTIN CHRNA5 C3orf52 LANCL2 ZNF582 CFL1 TIMM23B PVR JPT1 FAM135B SZRD1 GABPB1 IDH3A SERPINE1 SMN1 PKM KCNK5 FUNDC2 LINC00973* |
| 1795 down | 1632 | *SAMD4A PKNOX2 APBB2 ENSG00000256433 GALNT3 LDB3 GP1BA CHRNA10 ENSG00000279407 TRIB3 ENSG00000239291 ZEB1 CDK14 DNAJC3-DT FAT4 ENSG00000279278 PDE1C ENSG00000279149 ENSG00000241634 KNDC1 MED13L TMEM254-AS1 NFIA TCEANC2 HEIH ENSG00000280384 ENSG00000276900 INSIG1-DT MCPH1-DT NUDT9P1 TRIQK ENSG00000224691 ENSG00000279253 CCDC92B ARHGEF26-AS1 ENSG00000213963 ANKRD6 NEIL1 ENSG00000282885 ENSG00000286990 ENSG00000263235 ENSG00000271327 ROR2 CORO2A MAOA ENSG00000284471 ENSG00000275582 TMPO-AS1 LINC01011 ENSG00000259720 ENSG00000272320 ENTPD8 RFX3 KCNMA1-AS1 MYO15B IL24 ZNF284 WDFY2 ENSG00000283341 THRB SLC13A4 GTF2IP20 RAG1 IRS1 IGSF11 EDRF1-DT RPL13AP12 CROCCP3 MAPK10 ENSG00000271992 CACNB1 GLIS2 ENSG00000289183 CTC-338M12.4 LINC02895 MNX1-AS1 CAPN12 KCNQ5 ZBTB20 TARID ENSG00000267002 IGDCC3 ENSG00000232995 KIF14 DENND3-AS1 RTN4RL1 MSANTD2-AS1 TTTY14 LCA5L ENSG00000286158 PTPRH ENSG00000233967 SORBS2 ACAD11 CHL1-AS1 ARMH1 ENSG00000279989 ENSG00000289132 KRCC1 TJP3 FXYD2 TWF2-DT JPX DCX PLSCR4 DUS4L PPARGC1A MIR503HG ENSG00000272498 ZMIZ1-AS1 KCTD21-AS1 PCAT1 ENSG00000267340 KLHL6 HRH2 SMIM38 ENSG00000288979 ATP6AP1L ENSG00000285672 CELSR3 ZNF137P NOXA1 GRIP1 SAMD10 ENSG00000276698 C5 WNT6 SLC2A10 CCDC188 ENSG00000278875 UPK3B ENSG00000189229 ENSG00000280057 ENSG00000232876 TANC2 DLGAP1 RCAN1 LINC02614 ARHGAP25 ANKRD20A5P AGER CHROMR ENSG00000232545 PCDHGA5 FOXN2 HHIPL1 KLRG1 HEXIM2-AS1 FRG1-DT KDM6B ENSG00000270090 TRPC1 ENSG00000266718 NPIPP1 MSS51 PRECSIT ITPR1-DT NOL4L DICER1-AS1 PTP4A3 SLC48A1 YPEL1 ZNF536 HS3ST5 NYAP1 OR2L13 NDC80 LINC00622 ADIRF-AS1 ENSG00000270021 YJEFN3 SOCS3 ENSG00000231305 ZNF839 DACT3-AS1 ALPK1 ARID1B ENSG00000268218 BAIAP2L2 MAGI1 SLC23A3 PJVK TRMT9B AMOTL2 PSRC1 PCDHGA4 ENSG00000276952 MATN4 AMPD3 ENSG00000270605 ST6GALNAC3 TMEM263-DT ENSG00000285813 TMED2-DT ENSG00000280077 ENSG00000270933 INHCAP KLHDC1 ARHGAP8 ENSG00000288808 ENSG00000277978 KIF23 ATL1 ENSG00000287562 HFM1 ACSS3 SPEG CACNA1C GSEC DCST1 CNN3-DT PCED1B ENSG00000282556 LINC00964 IMPG2 ZNF385C ENSG00000233110 ENSG00000239467 KRT80 KCNQ4 TUBB2B C9orf72 ENSG00000215838 ENSG00000271780 NPIPB15 MKLN1-AS ENSG00000266934 TAS2R4 PNMA2 ENSG00000228137 ENSG00000223884 KRTCAP3 ENSG00000258559 ENSG00000286004 ENSG00000245552 HIC1 LRP1 ZNF790-AS1 ADHFE1 SPRY1 LINC02391 ENSG00000255429 NBEA LINC02405 ZMAT1 CACNA1C-AS1 CLASP1 MRPL23-AS1 SIRT5 INTS6-AS1 CHD2 ENSG00000230699 TIGD4 MFF-DT ERBB2 FAM225A ZSWIM9 PIF1 ZHX2 LINC01102 ENSG00000284946 GTF2IP23 CASP16P CCDC17 TBL1XR1 ENSG00000274414 FBLN2 ENSG00000286159 CCDC9B MBTD1 MAP3K12 PRX RAD50 ENSG00000289177 PSMG3-AS1 LINC00243 CRELD1 MRPS31P5 ENSG00000260563 WDR27 CEP192 IFT88 ENSG00000289059 SLC24A1 FAM66C HDAC11 PTPN6 CACNB2 TBC1D32 ENSG00000276449 ENSG00000205444 LINC00659 SOX6 LINC01687 ENSG00000227885 ENSG00000257298 TEX52 FRG1JP ENSG00000288538 VCAN LURAP1L-AS1 NHS ZNF594 ADAM8 HSPA7 PCDHGA11 NUDT13 MT1F MFSD14C C2orf68 MAMDC4 CLIP3 CNIH3-AS1 DDX11-AS1 BEND3P3 SRP14-DT ZNF252P-AS1 CLEC2D CPB2-AS1 ENSG00000273243 PDLIM1P4 MGAT3 ENSG00000213062 ENSG00000286909 ZNF37BP FAXDC2 ENSG00000230490 PTPRJ ENSG00000286364 ANKRD33B KCNQ3 ZNF135 TP53TG3D PER1 ENSG00000262587 HDAC10 OIP5-AS1 JAKMIP3 GNG12-AS1 PCDHGB3 NEURL1B ZNF789 GEMIN7-AS1 ENSG00000274276* |

| | | |
|---|---|---|
| | | *ST6GAL1 LINC02163 COL24A1 ARHGAP24 JMJD7-PLA2G4B FAM47E RN7SKP296 CKMT2 ENSG00000273325 ENSG00000280099 ENSG00000266313 C2orf88 LINC01622 ENSG00000274220 NOXRED1 ENSG00000256139 ENSG00000280053 BAIAP2-DT LENG8-AS1 ZNF486 HSD3BP5 DNAAF9 NEMP1 JRKL KMT2C TMEM147 GULP1 NR1D1 NEK2 C1orf162 ENSG00000283959 LINC01320 COL12A1 ENSG00000279794 YPEL2 ENSG00000286388 ENSG00000263280 LYPLAL1-DT DUSP8 LINC01219 AFF3 ESPL1 ARHGEF10L ERBB4 DNASE1 ENSG00000271533 LPP ZNF554 ENSG00000287855 HECA SCML2P2 TNIK MPPED2 EPB41L4A MBNL2 ARVCF MEX3B ENSG00000260051 LINC00308 HSPE1P26 ENSG00000273451 PPM1L-DT CCDC110 NBEAL1 CLHC1 UCN STX18-AS1 PTK2B FAM20A SH2D1A SLC9A5 SH3RF3 CDK15 ZSCAN30 DIP2C ENSG00000287766 MSH5-SAPCD1 FAM13A-AS1 ODAD1 STAG2 MAML2 ENSG00000262877 NFATC1 ENSG00000228793 LINC00482 FAHD2CP HTR2C RPS4XP16 MIR635 CFAP61 CMTM1 CTIF RAP1GAP2 SLCO4A1-AS1 ATP4A ENSG00000236829 NLRP6 ENSG00000277152 SAMD13 NEIL3 CEBPB-AS1 ZNF8 FZD2 NOVA2 PIBF1 PRKG2 ENSG00000272625 MTND5P1 SLC12A6 ENSG00000271947 KCTD13-DT ENSG00000232470 HMCN1 LAMB2P1 GSN-AS1 ZMYND8 ENSG00000259868 ENSG00000274225 LINC01695 SIRT4 RSKR FAM24B ARHGAP33 TCF12 LINC01356 PLEKHG2 CCNB3 CORO2B NUPR1 SH3RF3-AS1 COL4A3 EPM2A-DT ENSG00000273472 DNAJA2-DT UNC5C-AS1 ENSG00000232528 SYNE4 ENSG00000279080 ALDH1A2 ENSG00000285763 EGR1 CCL17 MIR3681HG MAGOH-DT NR4A1AS RPL23P2 MSI1 ENSG00000279381 ENSG00000278390 CYP4V2 SCNN1D EWSAT1 TM7SF2 MAP4K1 ACCS HECW2 PKD1L2 PPM1M PDE9A UBR5 ENSG00000272372 PCMTD1-DT HERPUD2-AS1 PEAK1 TOB1-AS1 SNORC PKD1P3 CHL1 VANGL2 DLG4 HOGA1 LINC01530 ENSG00000235119 ENSG00000285999 ENSG00000287262 LNP1 ENSG00000286373 SAMD9L MAML3 ENSG00000231703 SHANK1 ENSG00000287878 ARHGEF39 ENSG00000250961 ENSG00000269925 ANXA2R ENSG00000279048 CELF2-AS1 TSSK3 ENSG00000239415 ENSG00000255182 ZNRD1ASP EBF3 ENSG00000287110 PAK3 ENSG00000288880 ENSG00000215493 LNCOC1 ENSG00000271259 ENSG00000269918 UACA NPIPA1 TMEM169 TNKS LAMTOR5-AS1 ENSG00000273355 C5orf34 ENSG00000289506 ZNF818P KLF12 RUNDC3B ADAMTS20 RIMKLB CLMN AACSP1 TNS1 ENSG00000285679 PPP1R10 ZNF436-AS1 SSR4P1 ENSG00000230454 CASC2 OSGEPL1 RN7SL3 HERC2P4 ENSG00000244055 KRTAP5-AS1 CAPN10-DT VARS2 ATP6V0E2-AS1 MST1 BRPF3-AS1 JAZF1 DYNC2H1 PTPRVP ENSG00000270110 ENSG00000272791 CPEB1-AS1 SLITRK6 STX16-NPEPL1 ALDH1A3-AS1 GK-AS1 GDPD1 ENSG00000279926 FLJ43315 ENSG00000279759 SNRPGP4 ENSG00000277639 ENSG00000273893 HLA-F-AS1 CHAD HMGB2 FBXL2 LPAR2 PRICKLE2 PPT2-EGFL8 USP38-DT LINC02656 RNF165 ZMYM3 FAM200B ZNF429 NNT-AS1 RNF213 PRKCE FGGY-DT WDR31 SRCIN1 TIGD7 GLIS1 UHRF1BP1 CD37 DUSP19 ENSG00000272002 CCDC154 GRB10 ADGRD1 TBL1X EPHA3 LINC02604 PARGP1 MCPH1-AS1 ENSG00000274460 RPS6KA2-IT1 LEAP2 SEPTIN5 ENSG00000225649 EZH1 ENSG00000242861 LNCTAM34A LINC02918 DDIT4 CACNB4 ENSG00000261786 CCDC140 ENSG00000287385 SDAD1P1 ADGRB3 KLF7 ENSG00000275481 ENSG00000261799 MSH5 HIVEP2 ENSG00000279930 BLACAT1 ENSG00000272983 SOX5 KCNJ4 IQCA1 SEPSECS MTMR9LP PABPC1L TMPRSS5 ENPP5 SKOR1 CCDC18-AS1 SEPTIN7-DT FGD5P1 LINC00920 ST20 LINC01355 GDPD3 CYP2D7 ENSG00000226744 HTR6 TMEM198B FCGR2C VPS13B-DT ARAP2 NR5A2 EFEMP2 LOH12CR2 SHROOM3 PDCL3P4 LINC02021 ENSG00000272425 ENSG00000250397 EXTL3-AS1 ENSG00000261094 FRK TBX19 CDK5R1 TMEM116 PAX8 ENSG00000273261 IRF1-AS1 ENSG00000251417 ABHD12B KIF18A TNFRSF11A ZNF280B C19orf54 ENSG00000269896 LINC02878 KRT8P12 KMT2A ENSG00000269978 ENSG00000258101 ENSG00000261270 PRKAR1B-AS1 COX6B2 ENSG00000205041 ACBD4 KANSL1L-AS1 MTCYBP21 KIAA1614 ADAMTS13 C21orf62-AS1 CBLN3 LINC02289 C3orf18 DNASE1L2 CYB5RL ENSG00000289250 LINC02649 RNF157-AS1 OFCC1 ENSG00000279041 INSR ENSG00000260279 PHF13 ENSG00000286198 CLDN20 CIT ZNF607 ENSG00000197815 KIF28P MYO1F KLF15 ENSG00000274922 GBAP1 ENSG00000275180 HOXB9 BRME1 ENSG00000257176 LINC02099 RNU6-850P ENSG00000261118 INTS6L CATSPER2 ENSG00000289405 RAP2C-AS1 CDNF ZFHX3 HEMK1 TCP11L2 SLFN5 LINC02175 MMP25-AS1 CARF TUBG1P ENSG00000261211 ANKRD34A DDR2 LHFPL3 CFP ZNF37A DOCK3 ENSG00000264548 PRICKLE1 PDK4-AS1 MCF2L2 RN7SL262P CD63-AS1 NKX3-2 RASGRF2 ANKRD55 TRPS1 GTF2IP12 ERN1 KANTR SNHG14 PCDHGA6 CEP57 AR ENSG00000287820 GATM ENSG00000245317 ENSG00000279811 FRG2B KLHL3 ENSG00000279838 DAPK1 RMI2 RNF139-DT P3H3 ENSG00000287036 HCG20 ICAM5 UBE2Q2P1 POU5F1B CENPF ENSG00000232546 PPIL6 PRKAR2A-AS1 ENSG00000265298 LINC02610 CNOT6L CHKB-CPT1B ERVH48-1 AMZ1 CNTNAP1 ENSG00000268912 MGC16275 TESMIN ENSG00000270116 CMTM8 ENSG00000236013 CSPG4 FAM214A PELI2 SIDT1 TECTA KIRREL1 ENSG00000288993 ASNSP1 ENSG00000233539 DNAI1 IGF2BP3 TBC1D8-AS1 ENSG00000286482 LINC01569 HCN1 SYCP3 ITLN2 ENSG00000250041 TAGAP-AS1 ENSG00000227741 ENSG00000269954 ENSG00000261770 PEF1-AS1 BAHCC1 PAPSS2 ACSL3 UTRN CDC14A ENSG00000267248 TLR1 ENTPD4-DT ENSG00000258634 TMEM240 LINC01198 GPR37L1 CECR2 ZFP14 CBX3P2 SCX SCUBE3 RAB30-DT GRASLND ENSG00000279386 ENSG00000238018 TRIM2 BTN3A1 ENSG00000287997 ENSG00000280239 ATOH8 ENSG00000273442 HMGN1P1 PPM1K CARMIL1 STARD13 ZNF75A HESX1 IFT140 C14orf132 WDR19 PKD1P5 GPR19 ENSG00000254528 ENSG00000277050 SENP7 JARID2-DT RPL5P18 ZNF467 HES7 APRG1 ENSG00000223522 ENSG00000287609 ENSG00000215068 POU6F1 ENSG00000280537 PLD1 AKNA HPS4 RARB IMPDH1P8 IDI2 ENSG00000258744 CPAMD8 NOTCH2NLC ENSG00000280353 RARA-AS1 PCDHGC5 MEGF10 GGTA2P GNAO1 GDF9* |

| | | |
|---|---|---|
| | | *ENSG00000224950 ENSG00000268858 TMEM140 ZC3H6 ENSG00000280435 LINC01786 ASAP2 PSMD6-AS2 GABBR1 ENSG00000248161 ST7-AS1 ENSG00000271734 CARD14 ENSG00000263551 LINC00601 ARHGAP42 SLC16A1-AS1 PLA2G6 MALINC1 ENSG00000287202 CCDC113 GVQW3 BMP8A ENSG00000285804 NEDD4L GNRH1 FBLL1 BEX2 CFAP44 ENSG00000253200 ENSG00000241889 MST1P2 GABPB1-AS1 LINC00887 ENSG00000263089 GLI1 WNT5A ENSG00000274292 SLC25A34 SLC7A4 ENSG00000267404 ENSG00000261056 IL21R DNMT3A ENSG00000285774 WNK4 LINC00174 AGAP4 ZFP90 MEIOB NET1 ESR1 LMNTD2-AS1 FAM186B PAPLN-AS1 ENSG00000228327 CDC42-AS1 ENSG00000258811 ZNF514 PRSS27 HCG27 GTF2IP13 ENSG00000198358 NBPF17P SLC7A11 L3MBTL4 SLC6A16 SCARF2 NATD1 SCART1 ENSG00000288839 NLGN3 TM9SF5P EGFEM1P FIGN TMEM178A C1orf220 BTBD8 CKMT2-AS1 LINC00672 BNIPL GPT ENSG00000257027 NNMT ENSG00000228852 LRRC39 LINC02381 CREB3L4 SLC44A3-AS1 TNFRSF25 COPB2-DT CCR7 PROCA1 RECQL5 MIS18A-AS1 RGS1 STK32A-AS1 ZNF862 BBX RERE ZNF608 ENSG00000235902 CBX3P7 SLC25A42 KIAA1109 ENSG00000259495 MOCS2-DT ENSG00000111788 NR3C2 ZNF577 INPP5F IQGAP3 ZNNT1 ENSG00000287837 VASH2 DOC2A MIR4453HG TPTE2P1 DBP LRRC63 ZNF563 EGFL8 RENO1 TAS2R31 EFNA5 CENPE TMCO1-AS1 ENSG00000253476 SASH1 SLC22A23 PCDHGB7 INSM1 ABCA10 WBP2NL ZNF493 ENSG00000289067 AFDN-DT DSTNP1 CD109-AS1 C6orf163 PRKACB-DT TBCE PLCB4 LINC02251 HOXA2 ARHGEF2-AS2 RWDD3-DT ENSG00000261026 LINC02352 DMXL2 LINC01909 KIF12 ENSG00000285684 CASC15 ENSG00000278058 BTN2A2 MUC6 MEX3A ENOX1 BACE1-AS FCHO1 LINC02525 OR2L2 TRIM66 AKAP6 LBX2 ZNF132 RAB3GAP2 SLC22A15 ENSG00000262703 LINC00523 NAPSA C1QTNF6 CRACD LINC00858 SLC25A27 FNBP1P1 LRRC37B ZNF501 CEP170P1 ENSG00000267683 ELOVL2 CBX7 LINC01144 ENSG00000223945 ENSG00000271590 ANKRD44 ENSG00000255521 PCMTD1 FER GSDMB ENSG00000255557 ENSG00000270540 FAM72A CDK5RAP3 ENSG00000267546 TENT5B SMIM2-AS1 ZNF606 ENSG00000278918 KRT7 ZNF169 RAC3 DLGAP5 ZNF396 SGCD ENSG00000286757 LINC00339 LIMD1-AS1 NKPD1 ENSG00000262691 LRRC20 ENSG00000232611 LINC01447 SHROOM4 ICA1 NAV3 ENSG00000288748 ZSCAN23 ENSG00000273374 MX1 PRIMPOL ENSG00000198580 NLRP1 CCN1 LINC01583 GRIN3B ZNF615 FRG1GP APH1B ENSG00000273203 TPM2 CT75 ENSG00000272502 NCOA2 ENSG00000279518 ENSG00000280157 SGMS1-AS1 ACACB DLC1 SCARNA7 KATNAL2 SPRY4 ENSG00000279833 ZKSCAN8P1 ENSG00000285943 ENSG00000270171 NKILA CUTALP ENSG00000222044 SHISA7 ENSG00000237807 ZNF620 RGS17 LINC02728 CLDN1 SMG1P7 ENSG00000285184 LINC01341 LINC00638 ATP8B3 SNX18P1Y SNTB1 SLC20A1-DT ENSG00000251143 CHIAP1 LTK ALKBH6 ZNF860 JDP2 C13orf46 P2RX7 ENSG00000253854 TBX6 SPTBN4 ENSG00000244560 TSHR TMTC1 EPHA10 GSTM2 ETV1 IGSF22 SLC35E2A BACH2 NALT1 PHACTR2P1 PPP1CB-DT FXYD6P2 MIR3189 ENSG00000238260 ZCWPW2 ENSG00000277007 ENSG00000261474 ENSG00000277287 ZNF45-AS1 ENPP3 SATB1 YPEL3 XYLT1 ENSG00000272720 ENSG00000260855 BCDIN3D-AS1 EYA2 ZNF471 ENSG00000272529 LINC02293 LINC00847 TMTC2 MYADM PPP3CB-AS1 L3MBTL1 ATXN7L3-AS1 MEIS1-AS3 ENSG00000284526 PCP2 ENSG00000271971 ENSG00000212978 ENSG00000272264 ENSG00000272054 ZSCAN18 ENSG00000278869 SLC28A3 ENSG00000288884 ENSG00000276317 KCNAB3 ENSG00000225931 TUG1 ENSG00000267868 KEL C22orf46 ENSG00000280399 ENSG00000277938 ENSG00000286753 B4GALNT4 LINC02343 MDGA2 ENSG00000285725 NAV2-AS6 ENSG00000279161 PIGAP1 CLDN4 ARHGAP30 ENSG00000268970 ZNF277 ZNF704 NMRK1 SH3GL1P2 ZNF197 NEURL2 GABPB2 ENSG00000277496 SYPL1P2 LINC00173 LINC00906 LINC00898 TRPV6 LMLN ENSG00000260285 LDLRAD4 CHMP1B2P IQCH HSD17B1P1 CCDC26 TEAD3 FAM72B MYO5B MATN1-AS1 FAM86B1 ENSG00000232098 SEC31B NUDT7 ZNF665 TLR6 ENSG00000278041 ENSG00000259820 CAMK2D CCDC122 NTNG2 ENSG00000286174 HOXB3 ENSG00000261468 KLHDC7B ZNF716 KIF16B ARHGEF2 CDC25C PAXIP1-AS2 ENSG00000177788 ENSG00000280149 LINC01517 CCNG2 ENSG00000286985 ENSG00000251194 WDR88 C15orf65 FAM86B3P HOXB13 PPARG TMEM44 ODAD4 TANC1 LINC01366 LINC01572 ENSG00000267681 ENSG00000227329 LINC00565 ENSG00000289378 HDAC9 DLX4 HSD3BP4 ZCCHC18 KIAA1549L MYT1 ENSG00000233817 ENSG00000280332 ENSG00000258853 MAP2K6 AGO4 ZBED3-AS1 EN2-DT KAZALD1 ENSG00000276564 C11orf71 LINC01322 UGDH-AS1 ENSG00000286535 CDK6 TEAD2 KLLN LYST RAD51-AS1 ST8SIA1 ENSG00000264112 ENSG00000259341 STPG3 GCAWKR UNC13D LMOD1 NUDT6 CSRP1-AS1 RGL3 PRKCZ-AS1 BEST1 ACRBP RBM20 ENSG00000267666 CPT1B STK32A ENSG00000285980 SPATA6 ZNF234 SUCLG2-DT TPRXL IHO1 KBTBD11 ENSG00000287737 LINC00393 PDGFA-DT AGPAT4 NOX4 GTSE1-DT LINGO1 SALL2 ZNF546 CYP2E1 NRG2 CTH ENSG00000279315 GABARAPL1 APPBP2-DT ENSG00000289042 ENSG00000269397 ENSG00000255028 EIF1B-AS1 ENSG00000253573 AHSA2P OBSCN IL1RAP ENSG00000259668 SEMA6A-AS1 ENSG00000260077 DEF6 RPH3AL TRERF1 SNRPGP14 PTCH1 ENSG00000261959 FOXD2-AS1 ENSG00000288756 ENSG00000267199 PHBP13 ENSG00000273373 TNFRSF13C INTS4P1 MST1L LINC01249 ENSG00000241886 RAPGEF4 ENSG00000289318 RGS9BP ENSG00000197813 ANKRD31 C16orf86 ENSG00000286220 AGAP2 ZNF521 LHFPL3-AS1 ENSG00000275910 ENSG00000253395 ERVW-1 ENKD1 ENSG00000286207 PDXDC2P-NPIPB14P CORO6 ENSG00000279360 ANGPT1 LINC01531 FBXO43 CHAC1 MAST1 LTB4R SHF PHLPP1 PLIN1 ENSG00000284634 SPTY2D1OS MIR3936HG ENSG00000278998 ENSG00000093100 ARSI MIR3142HG ZNF610 LINC01625 PDE4DIPP6 TMEM51-AS1 ID2-AS1 PCDHGA2 OGT KRT8P46 FRG1DP BSG-AS1 ENSG00000259772 SCAPER ENSG00000216775 ENSG00000279672 CLDN15 IKZF2 ENSG00000259088 MKRN2OS YPEL4 ASPM TLE1 ENSG00000260604 LINC00933* |

| | | |
|---|---|---|
| | | *ENSG00000257252 PRIM1 ERICH2 AFAP1L2 ENSG00000261476 SPATA25 LAMB1 ADM2 RPS10P7 ENSG00000223947 MPRIPP1 GRID2IP PARP11-AS1 SBF2 ENSG00000286017 ADRA2B PCF11 RETREG1-AS1 TRIM7 NFIB CCDC191 PCSK4 ENSG00000261114 ENSG00000287957 CACNG8 ENSG00000259065 ENSG00000271833 TTC28 SGCA EXPH5 SLC16A13 PLCL1 TEX22 DDIT3 ENSG00000287236 STK4-DT IFIT1 ZNF460-AS1 DENND3 ENSG00000282897 ENSG00000262580 ENSG00000267504 GMDS-DT DOCK10 GNMT EPG5 COL2A1 L3MBTL2-AS1 ENSG00000236529 ENSG00000286485 PRKD1 LRRC4B ZNF674 RAPGEF3 ENSG00000289161 FBXO41 ITIH2 PRR5L TTC21A SLC12A5-AS1 ENSG00000285925 ENSG00000278635 LINC00205 ENSG00000272668 ENSG00000268575 HOXB6 ZNF528-AS1 ENSG00000255026 TMEM161B-DT SLX4IP LINC01002 LINC02615 ENSG00000289370 ENSG00000287650 ZEB2 ENSG00000279588 DBIL5P2 JMJD1C CICP14 PDE11A PAIP2B HJURP ARID4A OR1F1 RHOQ-AS1 CREBRF IFT81 KLC2-AS1 ENSG00000251364 TTN SRGAP3 NBR2 ZNF93 ENSG00000280128 ETFBKMT BTBD19 ENSG00000267277 MTSS1 SLC9A3-AS1 WNT5A-AS1 HMMR MORN4 CEP295NL RN7SL67P CCDC146 FAM227A ENSG00000272405 CDK19 GOT1-DT RHOBTB3 LINC02882 PKDCC ENSG00000280225 CCDC171 FBXO48 PAPLN PAX3 EFCAB13 TMCC1-DT SLC16A8 ENSG00000284968 BMPR1B ENSG00000243243 GATA6-AS1 PLAC4 DUSP16 BCL11A ENSG00000287910 LINC01234 LINC00648 SAMMSON GPT2 PTK6 THBS3-AS1 ENSG00000227775 ENSG00000235381 NECTIN3-AS1 BCAT1 ENSG00000280061 SMG7-AS1 FMNL2 ENSG00000284959 ENSG00000223393 ENSG00000286545 GAB1 ENSG00000267649 MYH7B JPH3 C20orf204 C21orf58 CYP24A1 ATP10B RFTN2 NPAS3 TNS2 ATP8B1 C2orf74 SEMA4C CCNYL2 EIF2AK3-DT PDGFC ENSG00000268204 CSPG4P13 C8orf44 ENSG00000275719 PHF21A DACT1 ANKRD23 RNF32-AS1 VPS13B N4BP3 HCG25 OSBPL5 CDHR2 ELOA-AS1 PIK3IP1* |
| 976 up | 932 | *PNMA1 GNL3 IFNA20P PDCL3 STOX1 AEN IRAK1 BRIX1 COPRS KBTBD8 PNPT1 CCDC97 ING3 LYAR RRP7A MRPL1 UFSP1 CMPK2 ITGA3 RABGGTA POU3F1 GRPEL1 RELT LMNB2 ENSG00000279692 CAVIN3 GFPT2 PPIL1 FAM98A PGBD5 H2BC21 ALG2 PRR19 PMF1 MIR193A GCLM ID1 TIMM22 ZNF35 PSMD11 ELAC2 EMC8 LINC01679 ZNHIT2 NCDN DOHH SETMAR RFX5-AS1 ENSG00000261762 ABCF2 ASNSD1 HGH1 TMEM11 MRPL15 H2BC11 COMMD5 ENSG00000256955 SLC9A3R1 PRR5 CHMP7 FKBP4 LONRF2 SF3A3 ENSG00000267405 BST1 HCFC1 KRT15 TRMT6 ARMC6 PXN-AS1 LINC00475 IFITM10 SNORD88A PPP1R14BP3 CHMP6 MANF EIF4E2 ENTPD7 FAM174C ENSG00000275106 FETUB MRTO4 C1QBP ENSG00000275993 PSTK PAQR3 NECAB2 ENSG00000267505 UCN2 ZNF511 IRF7 HSPA2 EIF2S1 EIF5A MCM10 DNAJC25 ENSG00000223461 E2F4 PSMD12 GCH1 ENSG00000289117 PODXL CSRNP1 NSUN5 SF3B5 ZNF18 TOMM22 CHPF2 MCAT VCP GAL RAVER1 PDXP GCLC ENSG00000257497 SEC61A1 ENSG00000279212 GALNT10 BANF1 PES1 WFS1 URB2 H2BC4 H2BC12 INTS7 ENSG00000286181 MAN1A1 PYCR3 CCDC103 ASH1L-AS1 ABHD5 CBWD1 GPS2P1 ZPR1 ENSG00000280486 LINC01979 CYRIA PRMT5 DCAF4 RNF126 RAP1BL SERPINI1 ENSG00000280010 IPPK EFHD2 SESN1 PUS3 NKX3-1 WDR74 ABCB9 NECTIN4 ABCF1 UFD1 MRPL14 PSD4 MPHOSPH6 HS6ST1 PSMC2P1 DEGS1 DDIAS NUDC EIF3J SNX11 LDHAP7 FARSA H2AX SLC30A1 TRMO KLHL18 CCNE2 TBX3 SWSAP1 FUT11 GPER1 C15orf39 MYDGF CCT3 NCL PCCA-DT MCM4 ID2 DDX21 KTI12 MESP1 CANT1 SLAMF7 ENSG00000227218 POLR2A SEC14L2 ENSG00000263823 RPUSD2 EMC6 SAMD15 MED31 ENSG00000279539 CA8 UQCRFS1P1 BCL7B SHPK C14orf119 C1orf53 TMEM41A EHD1 CDKN1A ASH2LP1 TRIML2 ARPC5L RPP25 EBNA1BP2 MAGEA12 CIAPIN1 PTRH2 SNORA33 ZNF668 SRM AKAP8 ENSG00000286996 ZNRD2 SEMA3F RIMKLA TOMM40 RPL23A ENSG00000215014 MARS2 NFKBIA SDF2L1 MRPL36 MON1A ZNF804A TTC4 WDR3 SNORA73B MT2A ALG1 PRCC DDX56 GMEB1 GEMIN5 ENSG00000263826 DDX20 MYBBP1A EEF2KMT CCT6A RCBTB2 H1-2 PNP ST6GALNAC1 METTL1 ANKRD37 CHST8 SNORD83A EIF6 SEC24D POLR2L MRPL4 LINC02119 ATP6V0D1 POLR1C ID3 TM9SF1 SPATA5L1 L3MBTL2 SFPQ AKR1B10 RARS1 DOLK RPP40 YARS2 HMOX2 ALMS1-IT1 CDK12 TAGLN2 ATP6V0B PELO MRPL12 CCDC137 CKB PSMB6 CCT2 SPRTN SYN1 LETM1 SLC25A44 VSNL1 H2BC5 LSG1 MERTK PUS1 ENSG00000279467 TUSC2 STON2 NRBP1 SLC25A22 ENSG00000255224 RRP36 MFSD2A TMEM47 CNTF FA2H ENSG00000261737 ENSG00000279605 MPV17L2 MEPCE RRAGA ABCE1 LDHA SPHK1 ENSG00000274270 INPP1 TMEM214 IER5L WDR4 RRS1 HSPA1A LRRC15 POLR3E OGFRP1 DHX37 C3orf80 FTSJ3 ENSG00000233388 RRP1B DKC1 THOP1 TRIAP1 ENSG00000213087 ENSG00000265749 MRPS26 DHX34 BRINP1 HSPA8P9 MT1X KEAP1 NOP56P3 EEF1A1P8 ENSG00000267397 NUP50 ARRDC4 ENSG00000279641 RN7SL832P SRP19 DIMT1 PYM1 TNFRSF12A TXNIP SRSF8 PTBP1 NSDHL LINC02735 LYPD1 SF1 DHX9 SMIM24 ENSG00000233825 PHRF1 IQCN ABCG2 MRPL18 PGAM5 RBM12 PECAM1 PRPF4 NOCT DDX28 UBE2S CDYL2 CCR1 NCS1 POMP NAP1L3 GMPPB AHSA1 MGAT2 NOP56 TATDN2 SYT6 TAF13 NUBP1 SHLD1 EIF5 PREB FHOD1 GTPBP4 SPNS2 UTP3 C16orf91 MUL1 TPT1P9 PUS7 TUBB6 DCTPP1 FDXACB1 CLU DPH2 CORO1A ZNF784 NCBP2 HNRNPAB ENSG00000264985 GADD45GIP1 FEN1 EFNB2 BAG2 ELOF1 GLYR1 SGMS2 TMED7-TICAM2 LRWD1 LRRC59 TXNDC9 TRAC RTP4 CEBPA TMEM201 SURF6 NOS1AP RRP12 NDUFB2-AS1 CARD8-AS1 ACTB SLC39A3 NSMCE3 FAAP100 PSMD1 DSEL ENSG00000237493 SRFBP1 BCL10-AS1 PRELID1 ATP6V0D2 NFKBIE TSR1 PRR7-AS1 POLR2C SHISA2 ENSG00000283064 ENC1 PNO1 PIGW RASL11A ENSG00000261888 MIR22HG LHFPL3-AS2 TRMT10C TNFSF9 ENSG00000279133 PDCD6IP-DT TOE1 TM4SF19 ZNF341 CALHM2 CRISPLD2 DDX3X PGD RND1 SAR1B UBE2J2 GNL2 PDE12 LIPT2 KCNJ2 GSKIP UTP20 ENSG00000264577 BYSL PDF PMPCA ENSG00000289164 PRADC1 SSC4D CYCS CLUH ENSG00000264666 ZNF576 PHACTR3-AS1 NOC4L POLR3K PRDX1P1 PUF60 BLOC1S2 RIOK1 SNHG25 SDAD1 ENSG00000279249 KPNA2 KPNA3* |

| | | |
|---|---|---|
| | | *ENSG00000279443 ENSG00000248968 ATP5MC1 TMEM250 BCLAF1 TPI1 IPO4 SRSF6 POLE3 BOP1 PLIN2 FAM98B HSP90AB2P HNRNPR PSMD3 EEF1AKMT4 AVEN MRPS12 ENDOG FGFR3 PTGES ENSG00000279965 CDC6 TENT5C HSPA8 LYSMD2 MAFA SAC3D1 FXN NOC3L RABEP2 KRT17 RBM38 PHACTR1 RDH8 ALYREF ZNF672 HEXIM1 RPL5P8 SNHG9 S100A3 GRWD1 TIMM17A PDSS1 SMG8 PGAM1 NGFR SHLD3 SEC13 PSMC4 PTMAP4 FNTB SERTAD1 HRCT1 FDX1 ENSG00000217275 MAP1A TRPM2 NOL11 PSMD2 USP18 POP7 ENSG00000262140 SURF4 BCAS2 TCF15 LDHAP3 SRRT SNRPG RBM3 BMS1 GEMIN4 SELENOS CTU2 CDR2L POR MPDU1 NAA15 LCMT2 MRPL9 RUNDC1 ABT1 RCC1 ENSG00000261889 MBLAC1 B9D2 HYAL2 ENSG00000279953 ENSG00000249050 SLC2A1 INTS5 TIMM8A MMP11 NUP35 NCOA5 CTR9 HAS3 SRCAP UBASH3B HMOX1 RABIF SERPINB8 U2AF2 ZMYND19 TUBB8P7 TSSC4 AAR2 TMEM70 RBM14 COA4 YRDC OSGIN1 ADAMTS15 SDHAF2 RRP1 FSCN1 RINL NOC2LP1 CTLA4 GLMN CCNQ TAX1BP3 WDR77 LINC01615 ENSG00000260136 DYNLL1 CTPS1 ENSG00000286570 EMP3 TUBA1C SNRNP25 DPF1 STIP1 CHST7 CSKMT S100A2 NOP14 COPS3 DMRT2 SOCS1 NPLOC4 TRIM28 PHLDA2 TST POP1 COA7 UTP11 MRPL27 RIOX1 SMTN CYB5R2 DNTTIP2 ZNF143 GOSR2 ISOC1 PET117 SCO2 CLSPN LENG1 TMEM115 ZNF628 EXOSC4 POLR1G SRA1 BRD2 LAMC2 OLFML3 FAM118B USP5 ADM LAP3 TUBB4B IMP3 MIRLET7BHG CAPN15 SBSN PTMAP5 ZMPSTE24 H3C3 PSMB3 AP5S1 ENSG00000287721 MED19 ENSG00000271851 ARMC5 BEND3 KRT12 RSL1D1 NAT1 ZFY EPHB2 MIEN1 CALM1 PLEKHA6 C8orf33 NME1 FAM222A TMEM11-DT ENSG00000276931 PIM2 CPNE7 GINS2 NOP56P1 MIR210HG MRNIP S1PR3 STEAP1 BLOC1S4 SIAH2-AS1 LTV1 ENSG00000232748 RYR3 ENSG00000288873 RNF25 MSC ATXN7L2 PTRHD1 NOSIP EIF3B RPL36A FASN ILF2 SMIM3 SQSTM1 HPS6 C7orf26 H2AW PUSL1 BRMS1 XIRP1 ATP1B1 PRPF19 ENSG00000260912 C2orf27A ENSG00000289554 HM13-AS1 MRPS17 MRGPRX4 ENSG00000259704 ENSG00000276853 NOP2 ENSG00000273568 CLP1 TMEM273 ENSG00000266651 ENSG00000178412 DHX30 IGFL2-AS1 MRPL54 PPP2R1B RRP9 LSM10 TUBA1B CRY1 MSX2 SCAND1 FBXO33 LINC01465 FAM169A ENSG00000222032 CHCHD7 SLBP CARD6 YJU2 NPTX2 RPUSD1 RAB27B RPLP0P6 SMIM12 LRATD1 HSPA8P1 CALR SNRPD1 BZW1 PCNA GPATCH4 FER1L4 CHUK HR POLH-AS1 CCDC86 STX11 KCNC3 DNAJA1 ALDH1B1 PLEKHA7 CYB561D2 KDM8 NOC2L ATF5 SPTBN5 PDCD2L CCNYL1 ISG20L2 ENSG00000274015 NT5C1B PRMT6 ICOSLG UTP14A NXF1 MRPL17 ZNF653 TFRC ADRM1 AIMP2 ENSG00000240652 INO80C ENSG00000272990 KPNA1 TAF6L TMPRSS2 NXT1 DGCR11 ENSG00000272768 ENSG00000278974 SNORD12C ADPRS GAR1 EIF4G1 HSP90AA1 LRRC47 PHF5A PGP CBLN1 TYROBP NIP7 TMEM158 HNRNPD-DT DPP3 PDE2A TUBB2A SACS MYH15 TBCC PLK2 CSTF2 TFB2M NUP188 SRXN1 LINC02577 TRIM21 C12orf43 MB ANKRD30B PCDH19 ARRDC3 TCAF2 XPA ZBTB9 DDN MSX1 AUNIP EPHX1 VPS18 SELENOTP1 SLC27A4 GEMIN7 PPIF HSPBP1 VPREB3 SLC20A2 ENSG00000260273 PCDH10 PRR7 SRPRB ADAT3 NOL6 SLC25A33 MYH7 PTS DDX54 MROH9 GLRX5 ENSG00000259041 PA2G4 MRPS2 POLR3H RILP ENSG00000289307 ACER2 SRSF2 NAP1L1P3 ARC PDCD11 EXOG C8orf76 ENSG00000272604 BRPF3 HSPA1B DDX23 GAPDHP63 SPRR3 NOLC1 VWA5B2 HSP90AB3P ENSG00000286064 UBIAD1 SNORD104 NPTX1 FAM217B SCAMP2 ENSG00000228477 RGMA GMNN KRT10 CCT5 ZNF295-AS1 FDXR MCRIP2 TLCD3A PSMD7 SNRPB MICALL2 TIMM21 CCN3 TLCD1 SF3B4 PSMC3 MFSD5 TMEM199 EXOSC6 PMEPA1 TACO1 NUDT15 ZNFX1 DCTN5 PSME3 CHAC2 TMED1 GEM CCL3 ONECUT2 ENSG00000277117 CCDC71 FAM180A SLC52A2 RAB5IF EIF4A1P7 SLC10A3 SLC25A5-AS1 PNPLA2 CFAP45 SPOCD1 ZNF622 ENSG00000250031 POLR3B* |
| 4C incr-2684 | 2461 | *RIT2 SLC18A1 SLMAP OR2AF1P LINC01201 KLHL13 ABCB7 AL355499.1 PBX4 PTPRR ACOT12 LOC124903770 ENO1P2 OR11P1P MYO9B GPSM2 LINC00683 COL4A5 LINC01708 ERG ENSG00000293384 PARN ENSG00000291325 CYTH1 ENSG00000286717 CHD9 AL390816.1 STK16 XK SEMA4D MMS22L MET MAX PDE4DIPP4 SCYL3 ENSG00000286637 LOC112268276 ATXN10 DIP2A KIAA1210 LOC105370954 EIF4G3 ACSM3 AC231532.2 CNBD2 BAZ1B SNX16 AC116035.1 WWC3-AS1 LINC02476 FKBP14-AS1 LINC02487 SLC8A1 FGGY SPAG11B LINC01915 IFTAP CEP350 AL356108.1 ZNG1F SUMO1P2 LINC00261 LINC01944 LINC02997 STAU2 RN7SL275P LINC01121 SIAH3 AC034154.1 PIWIL3 CETN3 AC116424.1 ARL14EP-DT RPL4P1 AC108025.1 CHMP1B AC015987.1 SLC9B1P5 AC004584.3 MMP16 AL136984.1 MIR654 DRD3 OR7E25P ENSG00000286104 AC091231.1 FAXC ATP11C PRORP ZNF534 RSRC1 POLR2J4 LINC02334 DNAH11 POLR2J POLG AC092957.1 EXOC2 PTPN5 DBIP2 RPS3AP26 AL358934.1 MYLK-AS1 TACR1 FSIP1 ENSG00000289332 AC131025.3 SNX25 AC068672.2 DPYD LINC02064 SDC2 AC131571.1 PDYN-AS1 ABHD12 GPHB5 SLC22A14 AP002954.1 AC005999.1 UBN2 COX10 VN1R12P CIITA AL160153.1 FANCC AP006219.1 ARAP1 UQCRHL LINC00305 AC073488.11 HEPHL1 KLHL7 RN7SL354P CCR3 ANKLE2 HBG2 GACAT3 CEP85 LOC101928565 RPRD1B ENSG00000287618 CR381653.1 LINC01828 CIP2A LOC105371855 AHNAK PPP1R3B-DT ZSCAN5A-AS1 YBX1P5 AHR LRRC53 ENSG00000285996 DLEC1 COX10-DT NFE2L2 AC004943.3 GRPEL2-AS1 AC069335.1 SCHIP1 MIR376A2 AC004594.1 SLC46A2-AS1 OLFML2A PARG UIMC1 TFEC NFYC AC022523.1 OTOA BLOC1S5-TXNDC5 UPP2 LOC105374367 MLIP-AS1 UBTD1 AC073488.10 LINC00313 BX640514.1 DTNB GPR158 VRK2 ITSN1 OAS1 AC128707.1 LINC02934 DEUP1 AC092552.1 ATP2B2 AC073488.2 CPVL NBPF13P PREX2 ESPNL ASTN2 LINC01374 AC007314.1 FAM177A1 ELAVL2 AC073488.5 MAP9-AS1 PSKH2 ENSG00000286332 AC135507.1 NAV2-AS4 LOC124903568 CSMD3 FBF1 NTN4 SERP1 GTF2IP1 CNTNAP3B AC090888.1 TBX3-AS1 POLR1A AC025031.1 PLEKHM3 LRP1B ENKUR LOC105375146 CENPBD2P TPTE2 AC017002.6 RAB11FIP4 AC010307.2 CLMAT3 AC116353.4 C10orf53 HSFX4 FGF12 GRPEL2 NPIPB5 SNTA1 PPCDC PARP4 RPL7AP83 MICOS10-NBL1 ENSG00000289178 ENSG00000293110 LINC02578 CLDN10 CCDC144A CBX3P10 ASZ1* |

| | | |
|---|---|---|
| | | *CU638689.3 RF00425 ENSG00000289699 NUB1 ENSG00000293482 MISFA WIF1 FLI1 CSNK2A2 DYM-AS1 LINC02197 PABPC5-AS1 SPATS2L CLIC5 ERICH3-AS1 ESRRB ARHGAP26 TDRD9 LINC-PINT ENSG00000290548 RBP7 TRHDE RAB44 PTPN4 AC105180.1 LEMD1-AS1 LOC652276 LOC107985211 SLC16A11 ITGA2 GRIN2A ENSG00000288643 AL442647.1 ENSG00000290070 KARS1P2 MIR5702 LOC285638 AC093459.1 NELL2 AC026316.5 IL1RAPL1 WDPCP AC119751.4 ENSG00000287108 AC091564.3 GNGT1 KIF5C BLK GPM6A SPATA16 AC068205.2 AC034195.1 AL359706.1 WNT16 RERGL KRT86 SETD3 PMS2P13 MRPS35P2 AC245517.1 CCZ1B AC011444.2 RPS6KA2 RN7SL271P EFR3B PLPPR5 RNF217-AS1 AVL9 LINC01790 TRIM24 RPS4XP20 ZDHHC17 AC005682.1 SMG1P5 HLCS CNBD1 LINC02831 FIG4 AC106864.2 LINC01621 EIF2AK2 CFAP47 AC026398.1 GSK3B-DT AP000844.2 U8 FOXP2 LOC102724452 RNU6-389P FP325331.1 ENSG00000288016 FBXO34 ENSG00000286062 ENSG00000286069 F2RL2 AC025031.4 AC078828.1 KMT2D GUSBP5 LPAR3 C3orf49 CKMT1B AC104116.1 SHE GIPC2 FMR1 C5orf64 AC008825.1 LINC01393 GFOD1 WASH7P ENSG00000288106 TXLNG SP110 SH3BP2 SH3TC2 CEP290 FAM171B CNTNAP3P2 WDFY3 AC239859.1 LRIG3-DT TACC1 RAD9B SH3PXD2B OCLN AC092484.1 CFAP91 AK8 TUT4 AC024598.1 ENSG00000293339 ITGBL1 NETO1 AL109935.2 PTPRQ GBE1 TNPO1 ENSG00000288620 SNX5 CDK13 AC004965.1 AC097634.4 DPY19L2P1 AL096711.2 SNX32 PCGEM1 DNPEP LOC100506321 AF130417.1 FAM230H CELF2-AS2 NEO1 LINC02894 GLT8D1 MYT1L RASA2 STAG1 LINC02428 CLDN14 IQCJ-SCHIP1 AC004702.1 CARM1P1 GUCY1B2 LINC01968 ITPKB LINC01829 PDE3A-AS1 PLEKHB2 CAPN11 UBOX5 TCERG1 AC092807.3 HBE1 AC024257.1 SLC66A1 F10 SLC7A14-AS1 APOO LINC01323 CFAP20DC-DT LINC00970 AC024581.1 LINC00370 SCN1A-AS1 LINC03099 DIO2 FAM184A AC073325.1 CABIN1 PSMD10 IRAG1 FBXO47 CCNO-DT FBXL17 SOX1-OT AC106798.1 EOLA1 AC104389.4 RPS3AP5 SLC9A4 DHX29 GAS2 STPG2 AP004833.1 LINC01678 NABP1 GPAM MSRB3-AS1 IRF2 HEATR6 GET1P1 SPATA6L LRP12 TCF12-DT ENSG00000289842 AC093766.1 ENSG00000291120 LOC100287944 AL353133.2 TSPEAR TBC1D22A FILIP1L LINC01492 LRRC1 ESRP1 HSFX3 HAND2-AS1 AL589666.1 NPIPB4 UBE2A GOLGA6L1 RBM41 AC099329.2 AC011477.4 RGS12 PPP6R3 LNCOG AC008133.1 AC245102.2 LINC02963 CCDC157 OSBP2 AC007529.2 RAPGEF5 CBX3 LINC02955 ACAP2 SLC39A10 KCNV1 CDCA2 PRKG1-AS1 NBL1 AC020687.1 ENSG00000289788 ZNF496 KCTD8 CHCHD6 ZNF550 MPHOSPH9 KIZ LINC00607 LOC102724289 RNF10 LINC02181 MGC4859 AC084357.2 FAT1 ENSG00000287051 AL137220.1 SLC2A2 ENSG00000291047 PIK3R1 AL078621.3 IL17B AC139143.1 LOC101928253 AGMO ABCB11 CDH19 SARNP CFDP1 PGM5 COL26A1 LINC01259 SHTN1 ATG12 AC073488.4 MASP1 AC034114.2 LOC100419716 PMS2P2 TMEM232 AC104365.1 USP50 CD200R1 LINC00587 BTD CALD1 STPG2-AS1 POLR3F CCDC144NL-AS1 AC068725.1 SH3GL2 ZNF847P CLBA1 HIBADH ENSG00000288577 LINC02233 CRTC1 VCF1 GSE1 LINC01611 N4BP2L1 PITPNC1 DARS1-AS1 TPTE2P6 MYLK3 ENSG00000291178 SLC30A3 NXPE3 AC091046.1 AL137009.1 ENSG00000286353 DOCK7 AC106706.1 PDK3 PTCSC2 MTX2 XKR4 THUMPD3-AS1 FBXL4 ROR1 AC079362.1 CAP2P1 SMAD3-DT RAPGEF4-AS1 TMEM183BP SCAP ADAMTSL1 VENTXP1 BABAM2 AC024382.1 PAX5 AAK1 AC090023.2 RTL8B LINC02755 CRB1 CTTN LINC02436 RNU6ATAC36P PASK USP8 AC026826.2 GPR75 AC116353.5 PARD6B ARID1A THEMIS LINC00404 PLPPR5-AS1 LOC124900945 SMAD6 OR2AJ1 FAM230B RYBP NYAP2 CLDN10-AS1 PSD3 AL355922.4 ABCA13 RFX2 AC012368.1 ACY3 AP002856.2 MEF2C FZR1 BAIAP2L1 ST8SIA5 DBNL TBC1D19 PPP1R9A-AS1 SRGAP2-AS1 AC235565.2 AC037486.1 MRPS36P3 ENSG00000287877 RMDN2-AS1 ELP4 RYK AP2B1P1 PXT1 TAF4B AC087639.2 MALT1 DENND11 AC079466.2 TUBGCP3 ITIH5 WEE2 IQCK MTM1 SLC15A5 ABCB10P4 CHMP4BP1 LINC00384 MRPS10P2 KCNE1 AC110296.1 NDUFAF4P3 LINC02930 PTPRE DOP1B ZSCAN5A ENSG00000289205 MFSD6 SERINC3 AL035401.1 TBC1D31 ZNF347 EXT2 PPIAP67 DIAPH2 CRYZL1 PTPN12 PTPRZ1 RNA5SP232 LINC01749 KAZN-AS1 AC105450.1 ACACA AC026786.2 CD55 PLN EML1 CTDSPL2 DDX39BP1 SHISA3 RPS3AP6 SLC66A3 SLC16A7 ALDH7A1P2 NEDD4 OCLNP1 NEK4 RBBP9 ENSG00000289849 CACNA1E FAM230G AC073488.9 LOC101927609 NF1P2 ENSG00000287526 LINC01203 AC024559.1 LOC105375297 ENSG00000293315 FLYWCH1 DOCK8 LRRC38 LINC01830 HMGB1P5 OVAAL AC078777.1 VWA8 CYSLTR2 DDAH1 DMBT1L1 PARVA LINC02154 PPP1R8 AC002064.1 AC106729.1 CCSER1 LINC02237 SECISBP2 URI1 LOC127903862 SLC9C1 MARK3P1 SCML2 AL031847.2 LINC01809 CHD6 FGD4 SSX11P ENSG00000286033 PDXK PDE4DIPP2 PMS2P6 NDUFAF6 COX17 HACE1 PACS2 NUDT16-DT SNRPF AL121782.1 SCD5 ATG4B ARMC8 AEBP2 SLC7A14 AL133255.1 DSC2 TRG-AS1 GLB1L SERPINA1 NPSR1-AS1 LOC100132172 KRT127P MYO10 LINC02693 LINC00376 CRHR2 RN7SL77P MID1 MYO16 AC020897.1 POGK ENSG00000289376 LINC00862 SLC12A2 EFNB3 OTX1 TRIM37 LOC101926964 ENSG00000286432 LINC02458 LINC02775 AC058822.1 RNU6-687P DLG3 FAR2 AC119751.1 SSR3 TRAT1 LOC105376219 ENSG00000291284 ERHP2 PROSER1 PVRIG FLOT2 SV2B FAM230A LINC01879 AC068987.2 AL445070.1 ILK LINC02552 LEF1 EYA1 DAAM2-AS1 MAPK14 ZNF816-ZNF321P PRR14L MYBPC3 WDR17 ENSG00000290921 AC011499.1 RNF216P1 ZFHX4 SLC25A13 ENSG00000290578 RAPGEF1 ALG13 ENSG00000287776 EFCAB14 GPRC6A AC068633.1 AC009139.2 FRMD6 UNC79 LOC100129616 SH3GL3 AKR7A2 RNU1-55P CERKL ENSG00000286728 SLC1A2 HOMER2P1 AL450442.1 AC027228.2 EIF2S3B ARHGEF3 BTBD9-AS1 GABRB2 LOC101927293 AC010196.1 LINC01938 GRIN2B SPDYE3 UBXN10 SVOP ARSB LINC01091 GPR141 CRISPLD1 ENSG00000289002 AC106895.2 IFNGR2 DENND4A ROR1-AS1 TBC1D20 AC083939.1 AL445218.1 GOLGA6L2 PLD5 RABGAP1 OXR1 LINC00700 AC245748.2 SOWAHCP2 SNX2 LOC729732 GRHPR AL929601.1 SCAMP5 IL17RB DCAF7 PARP2 MINDY3 MYL1 ZSWIM6 AC006041.1 KLHL4 TTC3-AS1 OVOL2 LINC03096 EIF3F MIR376B RDH16 ENSG00000286980 CFAP418-AS1 MTND3P22 RNU6-1117P ENSG00000290808 LNCTSI F10-AS1* |

| | | |
|---|---|---|
| | | *DNAJB6 LINC02612 SNRPGP9 MTCL3 AC023442.3 LINC02279 MIGA1 C8orf34 NPIPB3 RPL23AP7 RNU6-581P OMA1 ENSG00000289530 ENSG00000290967 LINC02683 ZNF724 PRR4 SPCS1 GABRA6 LEMD3 KHDRBS2 PLEKHF2 B3GLCT PIEZO1 SOAT1 BRD10 OR2T11 GGT4P SPTLC1P2 SLC9B2 ENSG00000287042 NEB AC103719.1 PTAR1 AL157359.2 AC090888.3 CSRNP3 AC087762.1 KANSL1L MRGPRX1 AL157944.1 AC018618.1 LINC00861 LINC02253 FAM120C LINC01588 LYPD6 LOC105370409 PDIA5 AL606517.2 PMS2P8 XPR1 LOC127814297 VDR ENSG00000287603 SPESP1 AC097625.1 CCDC149 AC087636.1 NEK11 PDPN CREB3L2 RTN4 ENSG00000286962 GNL3L AC096887.1 LINC03116 PRKAA2 ARHGEF11 SGPP2 CD163 DPT BBS2 CTNNA2-AS1 CLSTN1 SPAG17 RPL17P38 ZNF155 SERGEF SCYL2 AC090912.1 AOPEP SKAP1 AC190387.1 NT5C3A CU634019.5 AC108749.1 LINC02375 KRT18P35 MAOB AC022816.1 AC026415.1 LINC00395 SKIC3 MRPL3 MYO3A SERPINE2 ZNG1E LOC101928866 MICOS10 LOC105377146 NUBPL PLAAT3 GGNBP1 AC129915.3 POGLUT2 AC132153.1 DGKI IL12RB2 PSG2 LINC01221 LINC00363 LOC100507336 RPS3AP25 PIK3CG ANOS1 TPP1 LOC100130691 TBC1D30 STIL TFDP1 TRGV5 IL1R2 TNPO3 ATP6V1E2 NBPF10 HTT NUP160 AL935212.2 FGF14-IT1 MCCD1P1 SDK1 AC132803.1 AC137810.1 CDHR17P NECTIN3 AC016152.1 FREM3 AL136084.3 ABCB10P3 AC121757.1 IQGAP1 MYCBP2 WIPI1 MIR6841 LATS1 ENSG00000286875 AC098850.3 ZSCAN25 EPHA6 ANKRD20A21P LINC01442 AC007881.2 LOC124903099 ENSG00000293441 ENSG00000286215 KLHL12 LOC349160 ENSG00000286097 WDR72 SNX30 AC007092.1 AC007277.1 GNPDA2 TSNARE1 LINC00942 FANCI THNSL1 OR9Q1 SPARCL1 OR4K17 ACAA1 ENSG00000290589 ITGA9-AS1 CPEB2-DT ENSG00000287916 PRSS23 CPHL1P LRRC37A3 TMDD1 ENSG00000287299 EXD3 SPNS3 MTCO3P42 H2AZ1-DT TNFRSF10B IUR1 MAP2 RNU6-973P AC023078.5 GRM5P1 SOX2-OT CFTR ENSG00000286982 NPIPB13 ENSG00000287771 FBXW11 MTCL1 PRDX6-AS1 LINC01392 PTPRO ZFAND3 AC008268.1 SUGP2 PCDH9 LINC01029 AC108517.1 IYD AC026992.1 NKAIN2 SPATA21 MIR518C RBMS3 SLC29A4P1 AL603840.1 HNRNPA1P36 MIR646HG NHSL1 SMG6 ENSG00000287523 HLA-DMA AC093802.1 CEACAM16-AS1 AC010343.3 CFAP20DC TCERG1P2 SNORD17 LOC105370500 ENSG00000286041 RPS4XP23 PEMT ARHGAP6 LOC100420057 LINC01845 ENSG00000289503 LINC00927 POLR2J2 PPP2R2B AL356010.2 CHD5 LINC02196 JAK1 GPD2 IL12A-AS1 ITGA9 ACSL6 AC130448.2 TOR1AIP2 ULK2 RADIL KCNB1 AC015909.5 CANX ZMIZ1 UGP2 LINC00298 TRIM39-RPP21 CES5A RP1L1 AC034228.3 AC084200.1 AL109763.1 AC105919.1 GNPAT HSD17B12 HS6ST3 GRM4 LOC100887080 AC108010.1 AIM2 LINC01283 LINC00911 RF02271 SHC4 LINC02112 LINC00578 TMEM132B PPP1R14C MARK2P11 FRMD6-AS2 AL391095.1 AMT ENPP7P10 TESHL KITLG SV2C SATL1 ARID4B GMDS ZNF326 CSAG2 NDUFS5P5 CNIH3 MAP4K4 PLEKHM2 NR1I2 ZNF266 THSD4 NXPE1 AC106745.1 PENK-AS1 LARS2 ENSG00000286512 LINC01471 ENSG00000286371 HULC LINC01414 AC073488.8 RAET1E AC004852.2 AC068313.1 RFX7 MFHAS1 RELN OR51B5 SRP14P2 HS3ST4 AF107885.2 ENSG00000286556 LRRC37A ENSG00000286239 STAG3L4 PDHB ADD3-AS1 ADAMTSL3 PMS2P7 NXPH1 MTMR3 KMT2CP1 AC012467.2 CSAG3 CRKL MYO18B LINC00871 TENT5A FGD6 SYTL3 NDC1 AC009093.2 SULT1C4 LINC02742 SNAP91 LINC02074 ZNF816 AC104452.1 RPS6KC1 INPP5A CD40LG VRK1 AC100802.1 PAPOLB ENSG00000287950 URM1 LINC03041 GPAT3 ENSG00000287862 DPYSL3 ZNF420 SCN9A AC073488.3 C1orf146 ATP2B1 AC009498.1 SPRING1 AL807742.1 SLC16A10 AC005670.2 PLCE1-AS2 SLC28A2-AS1 ATRN FBLN5 CCDC88A NDUFB9 SNORA36C SLC35B4 UGT3A2 ARNT2 RNGTT SPEF2 RPS20P32 WDSUB1 PITX1-AS1 HPSE2 PLCE1 AC007262.2 TNIP3 FAM106A TRMT2B-AS1 ENSG00000288799 TACC2 AC073488.6 AC009950.1 NAALAD2 NT5DC1 AC099511.1 AC006927.5 AC022568.1 DCAF5 LINC01423 GNAL LINC01202 AP004607.6 CSTF1 PEBP4 STK32C AC020611.2 MIR7110 AC090114.3 OR7A1P RNU6-10P MSH3 MINDY1 UBE2F-SCLY LINC02664 MRPL45 CASTOR2 AC034268.2 DIS3L2P1 AC106894.1 LAX1 AC013644.1 LINC00705 PTPRG-AS1 STK26 CLCC1 AC005008.2 MIR520C AC002428.1 LINC03020 ENOX2 TENM4 AC068152.1 GOLGA6L25 TTC9-DT OGDH DCP1A LOC102723446 RPS12P20 ZCRB1 PPP3CA CES2 MIR526A1 AC133065.1 AC093799.1 GHR DUX4 MKNK1 DYSF NKD1 AL157762.1 ENSG00000286856 ENSG00000287849 RBPJ KMT2CP3 AL356807.1 GTF2IP4 CYRIB SPDYE17 ENSG00000287320 C3 RIN2 PDE6C AL137247.1 AC016629.1 PLEKHG7 DLX6-AS1 KBTBD3 ASCC1 CDH10 LRRC4C DENND1A MZT1 RPL13AP17 LINC02068 ENSG00000290317 PDZRN3 OR52D1 ADAMTS16 AP001341.1 TASP1 LINC00381 NBPF19 MICU1 AC008700.1 EBPL TAF10 MMP24OS ENSG00000287329 TRIP12 BACE2 KDM5A AC009154.1 ZNF549 AC010328.3 ZNF747-DT AP002761.2 AC004875.1 XAGE1A LINC02026 LOC102724354 LOC101927855 FCGBP NOMO1 AL133375.1 ARL6IP6 LINC01579 NEBL CNOT6 SDK1-AS1 PKIB AC007091.1 ENSG00000293024 SPANXA2-OT1 GALNT18 LINC02284 NAALADL2 TBX2-AS1 ASAH2 FRYL LINC01145 TBC1D9B LINC03019 RPL21P3 PXMP2 ASH1L RSPO2 LINC02266 TPMT AC016598.2 AC010636.2 ZNF747 ZNF805 ACSF2 ENSG00000288632 OR5K1 DOCK8-AS2 OR13C9 BMP6 PROX1 OSBPL10 MAP3K7CL ZNF891 RALGPS1 CCDC6 PAQR5-DT GUSBP1 SLC35F1 MUC5B CTSB AC026474.1 AL133353.1 DNAJC11 LINC00613 ASAP1 Z96074.1 GALNT17 LRRC2 VSIG1 CU633906.3 CFAP20DC-AS1 LRRC42 AQP8 CREBBP RPS10-NUDT3 AP000317.1 ITGB1 BVES RBM26 AC134698.4 AP003108.3 AC022031.2 RNA5SP29 CYB561 AC010632.3 SLC25A36P1 DRC7 AL109837.2 UBA5P1 AC093274.1 LMAN2L LINC00240 NFKBIZ LINC02346 LINC01823 ENSG00000291175 RHCE CORO1C KANK3 ENSG00000287247 NSG2 GPATCH11 LHFPL6 SPIDR CCDST C17orf80 AP001180.5 NEFL ATP6V1A TMEM185B IMPA1P1 STK3 CHN2 NBEAP3 FCAR AC119751.7 FSIP2-AS1 C1GALT1 HNF4G SMCO4 MSR1 EXOSC3 AP2B1 MED15P7 GABRG3 AC011453.1 ENSG00000290790 SGCZ PSMD7-DT AL138752.2 ENSG00000293093 LINC03095 DPY19L1P1 AC245060.5 CR936218.1 SH2D3C KCNAB1 APTX AC007000.3 TRPC4 RAMP3* |

| | | |
|---|---|---|
| | | *MTCO3P39 AL121718.1 ZNF705A ENSG00000289336 PCDH11X XYLT2 CPB1 MIR222HG LIMS1 LINC01456 ENPP2 DOCK4 UBL4B ARPC1A LINC01900 LINC00382 ENSG00000286458 BCL11B CERS3 GLCE PLS3 AC062021.1 TSPAN2 PCSK2 KCNIP1 PWWP2A LOC105373170 LRRTM4-AS1 SLC29A4P2 AC073283.3 MIR7851 GFRA2 TBC1D10A ENSG00000287188 EPHB1 CASP8AP2 AC007422.2 REEP1 LSAMP RPH3A ENSG00000287014 CTTNBP2 SLCO6A1 LINC01088 TULP2 NME9 CYTH4 NAP1L1 SLC38A6 LINC03109 MIPEP AL121594.1 HDAC2-AS2 VPS29 PLPP4 PWRN4 CCDC102B SNHG26 LINC01985 AC132219.1 COPB2 UBR1 RTCA-AS1 AC005972.3 CNN2 COL4A2 SLC1A6 TCF7L1 KMT2CP2 PAGE1 MRPS10 DYRK3 IP6K2 AC114501.2 KRTAP10-12 AC069444.2 LNC-LBCS CNTLN CHRNA6 AP000688.3 CMSS1 AP002765.1 ENSG00000288960 LINC02929 AC002428.2 ENSG00000287478 TLN2 HDAC4 MIR376A1 GDNF KLHL1 F11-AS1 LINC01276 TSKS FYB1 RIOK3P1 AL935212.1 FTO CALCB MARCHF5 RBM45 SEM1 AC073488.1 GYS2 IQCE LVRN LINC02326 MYLK UNC5C CFAP69 ACSBG1 MINDY4 LINC03051 CSMD2 TLR7 FLG2 IGHV3-62 SLC37A2 P3H2 AC064859.1 LINC00299 LARS2-AS1 PPIAP76 ENSG00000288067 LINC01793 AC006441.4 DTYMK KRT20 ENSG00000290821 WDR7 DIAPH2-AS1 SFI1 SLAIN2 HMG20A CEP85L TUSC3 COPG2 SNX18P5 PLEKHG4B ZFHX2-AS1 SYNCRIP CCDC38 RBBP8 AC090517.4 MED13 RNASEH2B PHF2 TAAR2 RAI2 AFDN ENSG00000288591 DEPDC1-AS1 CALN1 LRP2 CYTIP MTDH ENSG00000290606 ENSG00000287918 ENSG00000288804 SEMA6D GOLGA6L6 LIG1 EHMT1 ZDHHC13 WWC3 IL17RA LINC02715 LINC02069 SERPINA9 GALNT14 CLPX AC026410.1 RASA4EP AC009084.1 VWC2 REPS1 LINC01170 FBXL7 RN7SL25P RPA3 TOMM40P2 RNU6-1229P TMEM108 AC069209.1 RPL29P26 CC2D2B CLUHP10 TPTE BFSP1 CHMP1B-AS1 DOCK7-DT LCP2 GAB3 MB21D2 SEZ6L AC005828.4 AC007848.1 SLC39A8 STS TMEM123 AC002350.1 AC092675.1 AFG2B MANCR XAGE1B AL122018.1 AC009242.1 TAS2R13 AC233702.7 U2SURPP1 LINC00412 MAP4K3-DT MAP3K7 UPRT GTF2I TMEM59L ADAM17 LINC00968 ENSG00000286185 FAM78B MEI1 SEMA4F AL133353.2 ELAVL1 LIPJ ZFHX3-AS1 AC004922.1 TRAK1 STXBP1 OR6N1 AL353072.2 ENSG00000287923 AC008696.2 IDH1 NUAK2 AL109914.1 FP236315.1 LMOD2 AC092329.1 LINC00424 ENSG00000289846 LOC112268173 TSNAX-DISC1 MIR4713HG ABCC1 LINC01958 HDGFL2 MUC13 AC074131.1 NUP205 RBAK-RBAKDN NHLH1 CLDN14-AS1 AP005203.1 ELP2 SLCO5A1 ATP8A2 NHSL2 LINC02401 BX323845.2 RPSAP37 AC021443.1 SNRNP200 AL354676.1 ORC4 LINC01723 AC135586.2 AC022031.1 AC021231.1 LINC01204 AL591463.1 CYP8B1 ENSG00000286500 ZNF350-AS1 APC PEX5L-AS1 GNA12 LINC01680 LINC00540 AC090371.2 IL18R1 TTLL5 INO80 AL513325.1 C8orf74 NAA11 AP005328.1 LOC728554 MOCS2 ERGIC2 SULT2B1 SPTSSB PEAR1 CDA AC096711.3 LINC01591 BANCR PPARGC1B STON1-GTF2A1L CTRB1 AC093523.1 BMS1P17 AC078962.2 SYT16 AC239727.1 AC005225.3 AC091551.1 AL359237.1 AC124254.1 AC108081.1 BCAS3 AC092650.1 NOL10 ENSG00000286208 AC068759.1 LNX1 MIR376C AC087386.1 LOC105374191 ERC1 ENSG00000287694 SMIM13 AF228730.5 MIR8058 PARM1-AS1 ZNF440 ROS1 RUBCNL AGO3 UBOX5-AS1 C9 CYYR1-AS1 CASTOR3P CKAP4 MCTP1 TMPRSS11A LINC01837 ENSG00000293001 RPL39L ACER1 IQGAP2 CDYL RLF AC087289.4 CLPB ENSG00000290507 FAM230E CACTIN-AS1 AC092645.1 LINC00326 SPINK1 TNPO1-DT ANKRA2 C12orf42 AC026719.1 TAF5L AP000265.1 KMT2E LINC02841 LINC03060 CTNND2 CU633904.2 DNAH14 FRMD3 LARRPM AC010197.2 ERI1 DELEC1 RNF216-IT1 UGGT1 PACSIN2 RDH13 TIA1 AC127029.3 ENSG00000293401 RPL36AP47 MIR181A1HG DPYSL2 ENSG00000288700 NEK2P4 ENSG00000287169 SYNPO2 NUP210L AC008574.1 CNTNAP3C GIMAP8 AL049775.2 RAB8B AC017091.1 METTL8 AL023574.1 NSD2 ZNF516-DT ASCC3 ACYP2 GRM8 SLC23A2 PHACTR3 RMDN2 AL139352.1 LOC124900206 AC107023.1 EIF3J-DT PAIP2 AC005261.3 ENSG00000291209 AARS1 KCND1 PTPN11 ENSG00000286499 TMEM45A TMEM267 TTLL6 KIAA1958 PPIAP73 JADE3 CARD18 PHF3 ENSG00000287352 PSMC2 ENSG00000287831 MAPKAP1 LINC01853 ALK EXOC6B LINC01950 EVC2 LINC03017 GON4L RIC1 ENSG00000293214 AC007179.2 ENSG00000291262 AC007780.1 SEMA3A ENSG00000286812 AC104031.1 LINC02109 ENSG00000287362 MGAT5 AC104304.1 ACOX1 IGHV3-42 USP24 HAO1 MAEL NUP153 CLPTM1 MAD2L1-DT LINC01581 FBXO38-DT HSD17B4 EFHD1 LCN12 DCLK1 RNF215 AL500522.1 TRAV32 TRMT10B UFL1-AS1 COL21A1 RAI14 AL691403.1 ENSG00000292277 LOC285626 GPATCH2L ENSG00000286286 PIGR NRP2 GTF3A LOC101928386 SIPA1L2 CAVIN1 MIR548AD GYPE MSL3-DT AC006946.3 VPS41 LINC00879 SLC25A24P1 SNORD30 PLXNC1 KCTD5 C1orf21 METTL15 TMEM165 AC008537.1 AC016745.2 LINC01229 FOXJ2 RNU4-69P CEP162 HECTD4 ELOVL7 AC092111.3 NR3C1 DLX6 MIR300 AC068944.1 KMT2CP5 BORCS5 AC011466.2 FSTL4 ZC3HAV1 ABCB10P1 ARHGAP28 PGM5-AS1 MTOR LINC01797 AF212831.1 FAM230J USP13 ENSG00000287907 TRAV5 NBPF20 KSR1 LINC02217 FAF1 AC006116.9 TUFT1 SNAP25 RP1 AC004691.1 ADGRL4 PGM5P4-AS1 DEFB124 SGSM1 ARL10 ADH7 TIMM23B-AGAP6 MFSD4B-DT AC011476.2 CHST4 CCDC144BP AC005050.1 OR11H6 ENSG00000287258 AC083795.2 ENSG00000290565 GOLGA8A CLDND1 PIP5K1A GH2 BTBD2 MMACHC AC034234.1 AC131025.1 DISP1 ENSG00000293037 LINC01470 RAB6B HS6ST2 AC008539.1 KYNU AC015468.3 LOC105370906 SMN2 ENSG00000288894 ADAL AC090193.2 TBXAS1 ASCC2 AC046136.1 SPRED1 ENSG00000289744 ADAM10 GALC GNG4 NUDT19P4 WASHC1 AL445928.2 CDH26 CBY2 FAM53C ENSG00000293242 RNF32-DT SNX19P4 PDS5B CRIM1 ENSG00000289487 ENSG00000291233 NALF2 MEI4 RBM46 SUGP1 PFKFB3 SPANXD POTEA ABCB5 LOC105369292 HEATR4 RNF115 SPECC1 TRIM61 DPY19L2 ENSG00000287544 ESD CYYR1 CYP2G1P CDHR3 LINC00689 PTPRT PER2 AL139300.1 ERICH3 LINC01076 ANKRD24 IPO5P1 LHPP KAT6A BCOR KCNK12 NTRK3 RXFP1 LINC02932 SLC35F3 LINC01493 LINC01807 TADA1 STAG3L3 LINC02821 DIS3L2 ENSG00000290702 RAET1E-AS1 CTCF SNTB2 ENSG00000287291 ENSG00000286954 AC073263.1 PDCD10 PDE1A RSBN1L* |

| | | |
|---|---|---|
| | | *ENSG00000286584 LOC107985643 PRAMEF11 TFG EPHX2 RPS3AP21 LMTK2 AC023055.1 AL133268.3 HHAT UBE4A LINC00632 NWD2 PSMA3P1 CCDC68 THSD7B FAM227B SLC4A10 COPG2IT1 AL357153.3 GOLGA7 FERMT2 GCNA AC245519.1 GPR82 AC068587.3 AC130710.1 LOC124906209 SORCS1 GLUD1 AC008056.2 TSPOAP1 ST3GAL2 DNM3 AC016885.1 SYT1 CAMKMT SREK1IP1 PHIP DENND5A LINC00309 SS18 LOC124900792 SFRP5 RPL23AP38 ADAM6 GOLGA6L24 AGBL3 RFC2 ZYG11B CD244 AP000311.1 LINC01643 RN7SKP173 LINC01933 SHISA6 IGLV3-1 NFASC AC122134.1 OR2A1-AS1 HDX SCPEP1 WEE1P2 SLC28A2 AC094108.1 AL355499.2 RAI1 AC104339.1 ZNF229 LINC00513 GORAB-AS1 NXPE4 PHF6 HMGB4 EPB41L2 CADM3 ENSG00000288075 ENSG00000287533 FGF7 TENT5D MARCHF10 PACSIN1 TBCA ENSG00000287290 STXBP6 MGA LINC02549 DENND2B-AS1 AL137782.1 FAM230D FOXP4-AS1 AC021660.2 RTTN CACNA1A AC091544.5 AL596087.2 RN7SKP284 AC141586.1 LOC100533679 LIMCH1 TNFSF13B GNPNAT1 Z93403.1 AC006288.1 ENSG00000291170 MTMR7 PTH2R AC005909.1 ELOVL6 LOC100420587 AC125603.4 IAH1 MICAL2 COL14A1 AC073488.7 IGHV7-40 RNU2-38P SLC22A25 AC005828.3 ENSG00000286209 EIF2A JARID2 MICAL3 CELSR1 CYP1B1-AS1 CYTOR FIRRM HDAC2 KMT2CP4 RANBP17 TPO AP003385.1 TGOLN2 FGD1 GSTA3 AC008415.1 TRIM44 TRIM39 INSYN2B AC009090.6 NBPF26 AC105213.1 ENSG00000286134 ENSG00000286648 PROM1 ENSG00000287368 LINC01422 OR2T8 NPSR1 MSH2 RPL7P15 GLIDR MGAT4A ACP3 EIF4E3 USP48 KCNH5 RRM1 MAGEA1 CADM1 KLHL29 INPP5D CFAP161 THUMPD1 AL356309.1 CLYBL OR4C6 SPDYE9 RN7SL797P ATF6 PRKAR1B GBF1 IPO11 IQCJ SLF1 LINC02805 CCDC88C ABHD15-AS1 BACH1 CUL9 PRAMEF4 ENSG00000289228 INMT-MINDY4 EWSR1 DTX2P1-UPK3BP1-PMS2P11 TM7SF3 CYTH3 KLC1 SLC26A5 AKAP13 AC091047.1 HBS1L MLANA GPAT4-AS1 GCG SLC39A11 ARFGEF1 MRPL45P2 ATP12A AC090709.1 LINC01735 CCDC150 ENSG00000289293 TKFC ENSG00000290832 LINC02218 SMARCAD1-DT PTPN3 ENSG00000292979 SEPHS1P1 PMS2P10 STAC AC010547.2 AF241728.1 AL139042.1 RALGAPA1 RTL4 NNT SSX2IP KSR2 ST8SIA2 TTC17 ELP1 RPL7L1P16 IRF8 HIVEP3 LOC124904332 AC074386.1 PDE8B C2orf42 PHF5AP1 AC005999.2 AC018629.1 AL357052.1 AL008638.3 HEPACAM2 KAT7P1 AC023824.1 RFX4 CCDC198 AC118282.1 OR6K6 ZNF30 PHB1P13 ACTA2 REM1 LINC01482 FREM1 AC091564.7 CEP192P1 LINC02899 NDUFA10 CBR1-AS1 ENSG00000287579 CD69 B3GALNT2 AL132671.2 DBR1 EPS15L1 CYB561A3 AC021660.3 ENSG00000291188 XIRP2 Z99571.1 AC079950.1 AL078601.2 LINC01248 AL356272.1 KDM3A ENSG00000293483 MZT2A CAST OR2L3 AL354984.2 AC005394.1 TAOK1 UNC45A DLEU1 KBTBD11-OT1 ADGRA3 GPR39 CASK MIR6744 RPS29P9 SLCO4C1 GCNT1 BPIFB3 AC139493.2 PMS2P14 SLC35F4 AF121898.1 AC007731.3 GGA2 PRDM7 AC008277.1 USH1C FP236315.3 FMN2 AC118758.3 LOC101927468 LINC02307 UBXN2A LINC02269 AC136428.4 LINC01762 AC021188.1 NLRP14 PCNT DNAJC13 LINC02208 AFG2A LOC345471 NRXN1-DT PSME3IP1 TRDV3 ENSG00000286780 ENSG00000287469 LINC01478* |

**Table S3.** GO associations with biological processes (BP), molecular functions (MF), and cellular components (CC) of 163 rDNA-contacting genes detected in Mel Z cells grown on plastic. Related to Figure 1B.

| GO.ID | Description | padj | Genes |
|---|---|---|---|
| BP | | | |
| GO:0048731 | system development | 3.980125620413678e-7 | *BRCA1,ELAPOR2,SLC46A2,SRGAP2C,ZFHX2,ADCY1,FOXN3,SRGAP2B,SYNE1,RUNX2,MACF1,TFDP2,ZNF609,EDNRB,SETD5,SLC4A7,TMEM100,COL4A4,IGF1R,NLGN1,WNT2B,ANKRD27,ANK3,ARHGEF10,PTPN13,VAV3,APBA1,MBD5,DISC1,FMN1,ZFPM2,NAV2,ARHGAP32,CSGALNACT1,GLI2,TGFBR3,ATXN1,PLEKHA5,COL27A1,SYNE2,COL15A1,CBLB,SRGAP2,GLI3,ADGRG6,PPARA,MAP2K5,PLCB1,EDA,CNTRL,SEMA3D,SCN8A,RIPOR2,HSPG2* |
| GO:0007275 | multicellular organism development | 0.000004567168866081977 | *TEAD1,BRCA1,ELAPOR2,SLC46A2,SRGAP2C,ZFHX2,ADCY1,FOXN3,SRGAP2B,SYNE1,RUNX2,MACF1,TFDP2,ZNF609,EDNRB,SETD5,SLC4A7,TMEM100,COL4A4,IGF1R,NLGN1,WNT2B,ANKRD27,ANK3,ARHGEF10,PTPN13,VAV3,APBA1,MBD5,DISC1,FMN1,ZFPM2,NAV2,ARHGAP32,CSGALNACT1,CAMK4,GLI2,TGFBR3,ATXN1,PLEKHA5,COL27A1,PHLDB2,SYNE2,COL15A1,CBLB,SRGAP2,GLI3,ADGRG6,PPARA,MAP2K5,PLCB1,EDA,CNTRL,SEMA3D,SCN8A,RIPOR2,HSPG2* |
| GO:0032501 | multicellular organismal process | 0.0001729616116574427 | *TEAD1,BRCA1,ELAPOR2,SLC46A2,FBXL20,SRGAP2C,ZFHX2,ADCY1,GRID1,FOXN3,SRGAP2B,SYNE1,RUNX2,MACF1,TFDP2,ZNF609,ZNF516,EDNRB,SETD5,SMPX,SLC4A7,TMEM100,CACNA1D,TMEM150C,COL4A4,ANKFN1,IGF1R,NLGN1,WNT2B,ANKRD27,ANK3,GTF2IRD1,FBXO32,ARHGEF10,PTPN13,VAV3,APBA1,CYP39A1,MBD5,DISC1,FMN1,ZFPM2,NAV2,ARHGAP32,CSGALNACT1,CAMK4,GLI2,TGFBR3,ATXN1,PLEKHA5,COL27A1,PHLDB2,SYNE2,COL15A1,CBLB,SRGAP2* |

| | | | |
|---|---|---|---|
| | | | *,CNTNAP4,GLI3,GAB2,ADGRG6,EPB41,PPARA,MAP2K5,PLCB1,EDA,CNTRL,RBM47,SEMA3D,SCN8A,RIPOR2,HSPG2* |
| GO:0021801 | cerebral cortex radial glia-guided migration | 0.00103761008864 39878 | *SRGAP2C,DISC1,SYNE2,SRGAP2,GLI3* |
| GO:0022030 | telencephalon glial cell migration | 0.00103761008864 39878 | *SRGAP2C,DISC1,SYNE2,SRGAP2,GLI3* |
| GO:0048856 | anatomical structure development | 0.00174258806370 511 | *TEAD1,BRCA1,ELAPOR2,SLC46A2,SRGAP2C,ZFHX2,ADCY1,FOXN3,SRGAP2B,SYNE1,RUNX2,MACF1,TFDP2,ZNF609,ZNF516,EDNRB,SETD5,SLC4A7,TMEM100,COL4A4,IGF1R,NLGN1,WNT2B,ANKRD27,ANK3,ARHGEF10,PTPN13,VAV3,APBA1,MBD5,DISC1,FMN1,ZFPM2,NAV2,ARHGAP32,CSGALNACT1,CAMK4,GLI2,TGFBR3,ATXN1,PLEKHA5,COL27A1,PHLDB2,SYNE2,COL15A1,CBLB,SRGAP2,GLI3,GAB2,ADGRG6,PPARA,MAP2K5,PLCB1,EDA,CNTRL,RBM47,SEMA3D,SCN8A,RIPOR2,HSPG2* |
| GO:0007399 | nervous system development | 0.00181860030124 95365 | *ELAPOR2,SRGAP2C,ZFHX2,ADCY1,SRGAP2B,SYNE1,RUNX2,MACF1,ZNF609,EDNRB,SETD5,SLC4A7,IGF1R,NLGN1,WNT2B,ANKRD27,ANK3,ARHGEF10,PTPN13,APBA1,MBD5,DISC1,NAV2,ARHGAP32,GLI2,ATXN1,SYNE2,SRGAP2,GLI3,ADGRG6,PLCB1,SEMA3D,SCN8A,RIPOR2,HSPG2* |
| GO:0007610 | behavior | 0.00294655231731 0599 | *FBXL20,ZFHX2,ADCY1,GRID1,EDNRB,ANKFN1,NLGN1,APBA1,MBD5,NAV2,CAMK4,ATXN1,CNTNAP4,GLI3,PPARA,PLCB1* |
| GO:0021815 | modulation of microtubule cytoskeleton involved in cerebral cortex radial glia guided migration | 0.00304811878250 30995 | *SRGAP2C,SYNE2,SRGAP2* |
| GO:0065007 | biological regulation | 0.00382090110151 64006 | *NHLRC3,TEAD1,BRCA1,ELAPOR2,TOX3,SLC46A2,FBXL20,SRGAP2C,ZFHX2,SETBP1,ADCY1,GRID1,ANO4,FOXN3,SRGAP2B,SRGAP1,SYNE1,RUNX2,ZNF519,MACF1,TFDP2,ZNF609,TSHZ2,ZNF516,EDNRB,SFMBT2,SETD5,EBF1,FCHSD2,SLC4A7,MTUS1,TMEM100,CACNA1D,IVNS1ABP,INPP4B,ANKFN1,IGF1R,NLGN1,WNT2B,AZIN2,ANKRD27,ANK3,GTF2IRD1,ZNF19,HIP1,FBXO32,ARHGEF10,PTPN13,VAV3,APBA1,MBD5,ZNF320,STON1,DISC1,FMN1,ZFPM2,ZNF827,NAV2,GNG2,FRS3,ACKR2,MCTP2,BEND5,SSBP2,ARHGAP32,BAZ2B,CAMK4,GLI2,TGFBR3,ATXN1,PLEKHG1,PHLDB2,ESR2,MOB3B,RASSF3,SYNE2,COL15A1,CBLB,ZNF221,SRGAP2,CNTNAP4,PLEKHH2,GLI3,GAB2,CREB5,ADGRG6,EPB41,DOCK9,PPARA,MAP2K5,PLCB1,EDA,RBM47,SEMA3D,SCN8A,AGAP1,RIPOR2,HSPG2* |
| GO:0021799 | cerebral cortex radially oriented cell migration | 0.00420642001003 97405 | *SRGAP2C,DISC1,SYNE2,SRGAP2,GLI3* |
| GO:0051239 | regulation of multicellular | 0.00451237855405 8137 | *BRCA1,ELAPOR2,SLC46A2,ZFHX2,SYNE1,RUNX2,MACF1,ZNF516,EDNRB,TMEM100,CACNA1D,IGF1R,NLGN1,WNT2B,ANKRD27,GTF2IRD1,FBXO32,PTPN13,VAV3,MBD5,DISC1,ZFPM2,ARHGAP32,CAMK4,GLI2,TGFBR3,PHLDB2,CBLB,CNTNAP4,GLI3,EPB41,PPARA,MAP2* |

| | | | |
|---|---|---|---|
| | organismal process | | *K5,PLCB1,RBM47,RIPOR2,HSPG2* |
| GO:0032502 | developmental process | 0.0074226232444408605 | *TEAD1,BRCA1,ELAPOR2,SLC46A2,SRGAP2C,ZFHX2,ADCY1,FOXN3,SRGAP2B,SYNE1,RUNX2,MACF1,TFDP2,ZNF609,ZNF516,EDNRB,SETD5,SLC4A7,TMEM100,COL4A4,IGF1R,NLGN1,WNT2B,AZIN2,ANKRD27,ANK3,HIP1,ARHGEF10,PTPN13,VAV3,APBA1,MBD5,DISC1,FMN1,ZFPM2,NAV2,ARHGAP32,CSGALNACT1,CAMK4,GLI2,TGFBR3,ATXN1,PLEKHA5,COL27A1,PHLDB2,SYNE2,COL15A1,CBLB,SRGAP2,GLI3,GAB2,ADGRG6,PPARA,MAP2K5,PLCB1,EDA,CNTRL,RBM47,SEMA3D,SCN8A,RIPOR2,HSPG2* |
| GO:0050794 | regulation of cellular process | 0.0094696594376797 64 | *NHLRC3,TEAD1,BRCA1,ELAPOR2,TOX3,SLC46A2,FBXL20,SRGAP2C,ZFHX2,SETBP1,ADCY1,GRID1,FOXN3,SRGAP2B,SRGAP1,SYNE1,RUNX2,ZNF519,MACF1,TFDP2,ZNF609,TSHZ2,ZNF516,EDNRB,SFMBT2,SETD5,EBF1,FCHSD2,MTUS1,TMEM100,CACNA1D,IVNS1ABP,INPP4B,ANKFN1,IGF1R,NLGN1,WNT2B,AZIN2,ANKRD27,ANK3,GTF2IRD1,ZNF19,HIP1,ARHGEF10,PTPN13,VAV3,APBA1,MBD5,ZNF320,STON1,DISC1,FMN1,ZFPM2,ZNF827,GNG2,FRS3,ACKR2,MCTP2,BEND5,SSBP2,ARHGAP32,BAZ2B,CAMK4,GLI2,TGFBR3,ATXN1,PLEKHG1,PHLDB2,ESR2,MOB3B,RASSF3,SYNE2,COL15A1,CBLB,ZNF221,SRGAP2,CNTNAP4,PLEKHH2,GLI3,GAB2,CREB5,ADGRG6,EPB41,DOCK9,PPARA,MAP2K5,PLCB1,EDA,RBM47,SEMA3D,AGAP1,RIPOR2,HSPG2* |
| GO:0051128 | regulation of cellular component organization | 0.0096988307999518 33 | *TEAD1,BRCA1,SRGAP2C,GRID1,SRGAP2B,SYNE1,MACF1,SETD5,FCHSD2,IGF1R,NLGN1,ANKRD27,HIP1,ARHGEF10,PTPN13,STON1,DISC1,FMN1,ZNF827,ARHGAP32,TGFBR3,PHLDB2,ESR2,SYNE2,CBLB,SRGAP2,PLEKHH2,PPARA,MAP2K5,PLCB1,AGAP1,RIPOR2* |
| GO:0021814 | cell motility involved in cerebral cortex radial glia guided migration | 0.0105780812720130 35 | *SRGAP2C,SYNE2,SRGAP2* |
| GO:0048513 | animal organ development | 0.0123599838163465 16 | *TEAD1,SLC46A2,SRGAP2C,FOXN3,SYNE1,RUNX2,TFDP2,ZNF609,ZNF516,EDNRB,SLC4A7,TMEM100,COL4A4,IGF1R,WNT2B,DISC1,FMN1,ZFPM2,ARHGAP32,CSGALNACT1,GLI2,TGFBR3,ATXN1,COL27A1,PHLDB2,SYNE2,SRGAP2,GLI3,ADGRG6,PPARA,MAP2K5,PLCB1,EDA,CNTRL,SEMA3D,RIPOR2,HSPG2* |
| GO:0021795 | cerebral cortex cell migration | 0.0167958293653638 62 | *SRGAP2C,DISC1,SYNE2,SRGAP2,GLI3* |
| GO:0050789 | regulation of biological process | 0.0235920456036704 88 | *NHLRC3,TEAD1,BRCA1,ELAPOR2,TOX3,SLC46A2,FBXL20,SRGAP2C,ZFHX2,SETBP1,ADCY1,GRID1,FOXN3,SRGAP2B,SRGAP1,SYNE1,RUNX2,ZNF519,MACF1,TFDP2,ZNF609,TSHZ2,ZNF516,EDNRB,SFMBT2,SETD5,EBF1,FCHSD2,MTUS1,TMEM100,CACNA1D,IVNS1ABP,INPP4B,ANKFN1,IGF1R,NLGN1,WNT2B,AZIN2,ANKRD27,ANK3,GTF2IRD1,ZNF19,HIP1,FBXO32,ARHGEF10,PTPN13,VAV3,APBA1,MBD5,ZNF320,STON1,DISC1,FMN1,ZFPM2,ZNF827,GNG2,FRS3,ACKR2,MCTP2,BEND5,SSBP2,ARHGAP32,BAZ2B,CAMK4,GLI2,TGFBR3,ATXN1,PLEKHG1,PHLDB2,ESR2,MOB3B,RASSF3,SYNE2,COL15A1,CBLB,ZNF221,SRGAP2,CNTNAP4,PLEKHH2,GLI3,GAB2,CREB5,ADGRG6,EPB41,DOCK9,PPARA,MAP2K5,PLCB1,EDA,RBM47,SEMA3D,AGAP1,RIPOR2,HSPG2* |
| GO:1901888 | regulation of cell junction assembly | 0.037572099771082 55 | *SRGAP2C,SRGAP2B,MACF1,SETD5,NLGN1,PTPN13,FMN1,PHLDB2,SRGAP2* |
| GO:0022008 | neurogenesis | 0.0404932048570332 06 | *SRGAP2C,ZFHX2,ADCY1,SYNE1,RUNX2,MACF1,ZNF609,EDNRB,SLC4A7,IGF1R,NLGN1,WNT2B,ANKRD27,ANK3,ARHGEF10,DISC1,NAV2,ARHGAP32,GLI2,SYNE2,SRGAP2,GLI3,ADGRG6,SEMA3D,RIPOR2* |

| | | | |
|---|---|---|---|
| GO:0040007 | growth | 0.0424139822762782 95 | *TEAD1,BRCA1,MACF1,APBA1,MBD5,DISC1,FMN1,ZFPM2,ARHGAP32,GLI2,TGFBR3,COL27A1,ESR2,GLI3,PPARA,MAP2K5,PLCB1* |
| GO:0008347 | glial cell migration | 0.048526692672139 04 | *SRGAP2C,DISC1,SYNE2,SRGAP2,GLI3* |
| | | *CC* | |
| GO:0030054 | cell junction | 0.000032467758354 34947 | *TNS3,CDC42BPA,FBXL20,SRGAP2C,ADCY1,GRID1,SRGAP2B,SYNE1,PDZD2,MACF1,SMPX,FCHSD2,NLGN1,ANK3,HIP1,APBA1,STON1,DISC1,FMN1,GNG2,MCTP2,ARHGAP32,PLEKHA5,PHLDB2,SYNE2,CBLB,SRGAP2,CNTNAP4,EPB41,PLCB1,SCN8A,AGAP1,PARD3B,HSPG2,FRMD4B* |
| GO:0098978 | glutamatergic synapse | 0.000098530155604 53506 | *FBXL20,SRGAP2C,ADCY1,GRID1,SRGAP2B,SYNE1,NLGN1,HIP1,APBA1,DISC1,ARHGAP32,PLEKHA5,CBLB,SRGAP2,PLCB1,SCN8A* |
| GO:0005856 | cytoskeleton | 0.000627504706086 749 | *TNS3,BRCA1,CDC42BPA,SYNE1,PDZD2,MACF1,TFDP2,MTUS1,IVNS1ABP,ANKFN1,ANK3,HIP1,ARHGEF10,PTPN13,DISC1,FMN1,ACKR2,ARHGAP32,GLI2,PHLDB2,RASSF3,SYNE2,TUBA3D,PLEKHH2,GLI3,EPB41,NCKAP5,MAP2K5,EDA,CNTRL,SCN8A,RIPOR2,FRMD4B* |
| GO:0098794 | postsynapse | 0.011247589964422 761 | *ADCY1,GRID1,SYNE1,NLGN1,ANK3,HIP1,APBA1,DISC1,ARHGAP32,PLEKHA5,CBLB,SRGAP2,PLCB1,SCN8A,AGAP1* |
| GO:0045202 | synapse | 0.017991186830609 292 | *FBXL20,SRGAP2C,ADCY1,GRID1,SRGAP2B,SYNE1,FCHSD2,NLGN1,ANK3,HIP1,APBA1,STON1,DISC1,GNG2,MCTP2,ARHGAP32,PLEKHA5,CBLB,SRGAP2,CNTNAP4,PLCB1,SCN8A,AGAP1* |
| GO:0099081 | supramolecular polymer | 0.038695318488358 374 | *SYNE1,MACF1,SMPX,MTUS1,CACNA1D,COL4A4,ANK3,FBXO32,DISC1,FMN1,ACKR2,COL27A1,RASSF3,SYNE2,TUBA3D,NCKAP5,SCN8A* |
| | | *KEGG* | |
| KEGG:04725 | Cholinergic synapse | 0.005750318779348 733 | *ADCY1,CACNA1D,GNG2,CAMK4,CREB5,PLCB1* |
| KEGG:04926 | Relaxin signaling pathway | 0.011996412954390 936 | *ADCY1,EDNRB,COL4A4,GNG2,CREB5,PLCB1* |
| KEGG:04925 | Aldosterone synthesis and secretion | 0.029302123659000 47 | *ADCY1,CACNA1D,CAMK4,CREB5,PLCB1* |
| KEGG:04024 | cAMP signaling pathway | 0.037079888747413 435 | *ADCY1,CACNA1D,VAV3,CAMK4,GLI3,CREB5,PPARA* |
| KEGG:04022 | cGMP-PKG signaling pathway | 0.043983460304375 92 | *ADCY1,EDNRB,CACNA1D,GTF2IRD1,CREB5,PLCB1* |

**Table S4.** Venn diagram showing the intersections of downregulated and upregulated genes detected in Mel Z cells grown on Matrigel with genes whose number of contacts with nucleoli were decreased. Related to Figure 1C.

| Names | total | elements |
|---|---|---|
| 1795 down 4C decr-3331 | 188 | *APBB2 CDK14 PDE1C MED13L NFIA ARHGEF26-AS1 MAOA THRB CROCCP3 MAPK10 CACNB1 KCNQ5 SORBS2 PCAT1 GRIP1 DLGAP1 ZNF536 ARID1B ACSS3 CACNA1C CNN3-DT LINC02405 INTS6-AS1 TBL1XR1 FBLN2 MBTD1 CEP192 TBC1D32 FRG1JP NUDT13 KCNQ3 COL24A1 C2orf88 DNAAF9 GULP1 COL12A1 AFF3 LPP MPPED2 EPB41L4A PTK2B CTIF ATP4A PRKG2 HMCN1 ZMYND8 RSKR ALDH1A2 MIR3681HG UBR5 CHL1 ARHGEF39 PAK3 KLF12 CASC2 ATP6V0E2-AS1 CPEB1-AS1 GDPD1 FLJ43315 PRICKLE2 ZNF429 ADGRD1 EPHA3 CACNB4 ADGRB3 KLF7 SHROOM3 KIF18A ZNF280B KMT2A KIAA1614 OFCC1 ZNF607 Y_RNA ZFHX3 LHFPL3 RASGRF2* |

| | | |
|---|---|---|
| | | *ANKRD55 DAPK1 PPIL6 LINC02610 PELI2 HCN1 TLR1 CECR2 APRG1 PLD1 RARB MEGF10 GGTA2P GNAO1 ASAP2 GABBR1 NEDD4L CFAP44 WNT5A AGAP4 ESR1 SLC7A11 L3MBTL4 EGFEM1P FIGN BTBD8 BNIPL SLC44A3-AS1 COPB2-DT MIS18A-AS1 STK32A-AS1 BBX RERE NR3C2 EFNA5 DMXL2 CASC15 AKAP6 RAB3GAP2 TENT5B SMIM2-AS1 SHROOM4 ZSCAN23 MX1 ZNF615 NCOA2 ACACB DLC1 RGS17 CLDN1 ZNF860 TSHR TMTC1 ETV1 ZCWPW2 EYA2 LINC02293 TMTC2 ZNF197 LINC00906 TRPV6 LDLRAD4 IQCH ZNF665 TLR6 CAMK2D KIF16B PPARG TANC1 LINC01322 CDK6 ST8SIA1 STK32A ZNF234 TPRXL KBTBD11 LINC00393 EIF1B-AS1 IL1RAP SEMA6A-AS1 TRERF1 RAPGEF4 ZNF521 ENSG00000286207 PDXDC2P-NPIPB14P ANGPT1 PHLPP1 IKZF2 AFAP1L2 SPATA25 LAMB1 SBF2 NFIB TTC28 GMDS-DT PRKD1 TMEM161B-DT LINC01002 PDE11A TTN SRGAP3 LINC02882 EFCAB13 BCL11A SAMMSON NECTIN3-AS1 FMNL2 NPAS3 ATP8B1 VPS13B ELOA-AS1* |
| 4C decr-3331 976 up | 56 | *ING3 TRMT6 ARMC6 GCH1 URB2 SERPINI1 HS6ST1 DDIAS SAMD15 EBNA1BP2 RIMKLA ZNF804A GEMIN5 EEF2KMT SFPQ CDK12 STON2 TNFRSF12A SF1 CDYL2 PUS7 SGMS2 TMED7-TICAM2 NOS1AP PSMD1 SRFBP1 POLR2C SHISA2 SAR1B GSKIP AVEN TENT5C PHACTR1 FNTB WDR77 ZNF143 GOSR2 PET117 EPHB2 RYR3 ATP1B1 C2orf27A KCNC3 SPTBN5 TFRC EIF4G1 SACS MYH15 CSTF2 PCDH19 HSPBP1 SLC20A2 PCDH10 BRPF3 PMEPA1 CHAC2* |
| 1795 down | 1607 | *SAMD4A PKNOX2 ENSG00000256433 NHLRC3 GALNT3 LDB3 GP1BA CHRNA10 ENSG00000286540 ENSG00000279407 TRIB3 ENSG00000239291 ZEB1 DNAJC3-DT FAT4 ENSG00000279278 ENSG00000279149 ENSG00000241634 TNS3 KNDC1 TMEM254-AS1 TEAD1 TCEANC2 HEIH ENSG00000280384 ENSG00000276900 INSIG1-DT BRCA1 MCPH1-DT NUDT9P1 TRIQK ENSG00000224691 ENSG00000279253 CCDC92B ENSG00000213963 ANKRD6 NEIL1 ENSG00000282885 ENSG00000286990 ENSG00000263235 ENSG00000271327 ROR2 CORO2A ENSG00000284471 ENSG00000275582 TMPO-AS1 LINC01011 ENSG00000259720 ENSG00000272320 ENTPD8 RFX3 KCNMA1-AS1 MYO15B IL24 ZNF284 WDFY2 ELAPOR2 ENSG00000283341 TOX3 RALY-AS1 CDC42BPA SLC13A4 GTF2IP20 RAG1 IRS1 IGSF11 EDRF1-DT RPL13AP12 ENSG00000271992 GLIS2 ENSG00000289183 CTC-338M12.4 LINC02895 MNX1-AS1 CAPN12 ZBTB20 TARID ENSG00000267002 IGDCC3 FAM218A ENSG00000232995 KIF14 DENND3-AS1 RTN4RL1 MSANTD2-AS1 TTTY14 LCA5L ENSG00000286158 PTPRH ENSG00000233967 ACAD11 CHL1-AS1 ARMH1 ENSG00000279989 ENSG00000289132 KRCC1 TJP3 FXYD2 TWF2-DT ENSG00000286830 JPX DCX PLSCR4 DUS4L PPARGC1A MIR503HG ENSG00000272498 ZMIZ1-AS1 KCTD21-AS1 ENSG00000267340 KLHL6 HRH2 SLC46A2 SMIM38 ENSG00000288979 ATP6AP1L ENSG00000285672 CELSR3 ZNF137P NOXA1 SAMD10 ENSG00000276698 R3HDM2 C5 WNT6 SLC2A10 CCDC188 ENSG00000278875 UPK3B ENSG00000189229 ENSG00000280057 ENSG00000232876 TANC2 RCAN1 FBXL20 LINC02614 ARHGAP25 ANKRD20A5P AGER CHROMR ENSG00000232545 PCDHGA5 FOXN2 HHIPL1 CHD1-DT KLRG1 HEXIM2-AS1 FRG1-DT KDM6B TTC28-AS1 ENSG00000270090 TRPC1 ENSG00000266718 NPIPP1 SRGAP2C MSS51 PRECSIT ITPR1-DT NOL4L PPIEL DICER1-AS1 PTP4A3 SLC48A1 YPEL1 ZFHX2 NR2F2-AS1 HS3ST5 NYAP1 OR2L13 SETBP1 NDC80 LINC00622 ADIRF-AS1 ENSG00000270021 YJEFN3 SOCS3 ENSG00000231305 ZNF839 DACT3-AS1 ALPK1 ENSG00000268218 BAIAP2L2 MAGI1 SLC23A3 PJVK TRMT9B AMOTL2 PSRC1 PCDHGA4 ENSG00000276952 MATN4 AMPD3 ENSG00000270605 ST6GALNAC3 TMEM263-DT ENSG00000285813 TMED2-DT ENSG00000280077 ADCY1 ENSG00000270933 INHCAP GRID1 KLHDC1 ARHGAP8 ENSG00000288808 ENSG00000277978 KIF23 ATL1 ENSG00000287562 HFM1 SPEG GSEC DCST1 PCED1B ENSG00000282556 LINC00964 IMPG2 ZNF385C ENSG00000233110 ENSG00000239467 KRT80 ANO4 KCNQ4 TUBB2B C9orf72 ENSG00000215838 ENSG00000271780 NPIPB15 MKLN1-AS FOXN3 ENSG00000266934 TAS2R4 PNMA2 ENSG00000228137 ENSG00000223884 KRTCAP3 ENSG00000258559 ENSG00000286004 ENSG00000245552 HIC1 KC6 LRP1 ZNF790-AS1 ADHFE1 SPRY1 LINC02391 ENSG00000255429 NBEA ZMAT1 CACNA1C-AS1 CLASP1 MRPL23-AS1 SIRT5 CHD2 ENSG00000230699 TIGD4 MFF-DT ERBB2 LINC00589 FAM225A SRGAP2B ZSWIM9 PIF1 ZHX2 LINC01102 ENSG00000284946 GTF2IP23 CASP16P CCDC17 ENSG00000274414 DNPEP-AS1 ENSG00000286159 CCDC9B SRGAP1 MAP3K12 ARHGEF35-AS1 PRX RAD50 SYNE1 ENSG00000289177 PSMG3-AS1 LINC00243 CRELD1 MRPS31P5 ENSG00000260563 WDR27 KRT89P PDZD2 IFT88 ENSG00000289059 SLC24A1 FAM66C HDAC11 PTPN6 CACNB2 ENSG00000276449 ENSG00000205444 RUNX2 LINC00659 SOX6 WEE2-AS1 LINC01687 ENSG00000227885 ENSG00000257298 TEX52 ENSG00000288538 VCAN LURAP1L-AS1 NHS ZNF594 ADAM8 HSPA7 PCDHGA11 MT1F MFSD14C C2orf68 ACOXL MAMDC4 CLIP3 CNIH3-AS1 DDX11-AS1 BEND3P3 SRP14-DT ZNF252P-AS1 CLEC2D CPB2-AS1 ENSG00000273243 PDLIM1P4 MGAT3 ENSG00000213062 ENSG00000286909 ZNF37BP FAXDC2 ENSG00000230490 PTPRJ ENSG00000286364 ANKRD33B ZNF135 TP53TG3D PER1 ENSG00000262587 HDAC10 OIP5-AS1 FNDC3B JAKMIP3 GNG12-AS1 PCDHGB3 NEURL1B ZNF789 GEMIN7-AS1 ENSG00000274276 ST6GAL1 LINC02163 ARHGAP24 JMJD7-PLA2G4B FAM47E FHIP1A RN7SKP296 CKMT2 NBPF14 ENSG00000273325 ENSG00000280099 ENSG00000266313 LINC00663 LINC01622 ENSG00000274220 NOXRED1 ENSG00000256139 ENSG00000280053 BAIAP2-DT LENG8-AS1 ZNF486 HSD3BP5 NEMP1 JRKL KMT2C TMEM147 NR1D1 NEK2 C1orf162 ENSG00000283959 LINC01320 ENSG00000279794 YPEL2 ENSG00000286388 ENSG00000263280 LYPLAL1-DT DUSP8 ZNF519 LINC01219 ESPL1 ARHGEF10L ERBB4 DNASE1 ENSG00000271533 ZNF554 ENSG00000287855 HECA SCML2P2 TNIK MACF1 MEF2C-AS1 MBNL2 STARD4-AS1 ARVCF MEX3B ENSG00000260051 LINC00308 HSPE1P26 ENSG00000273451 PPM1L-DT CCDC110 NBEAL1 TFDP2 CLHC1 UCN STX18-AS1 FAM20A SH2D1A SLC9A5 SH3RF3 CDK15 ZSCAN30* |

| | | |
|---|---|---|
| | | DIP2C ENSG00000287766 MSH5-SAPCD1 FAM13A-AS1 ODAD1 STAG2 MAML2 ENSG00000262877 NFATC1 ENSG00000228793 LINC00482 FAHD2CP HTR2C RPS4XP16 MIR635 CFAP61 CMTM1 RAP1GAP2 SLCO4A1-AS1 ENSG00000236829 NLRP6 ENSG00000277152 SAMD13 NEIL3 CEBPB-AS1 ZNF8 FZD2 NOVA2 PIBF1 LRRC37A2 ENSG00000272625 MTND5P1 SLC12A6 ENSG00000271947 ANKRD28 KCTD13-DT ENSG00000232470 LAMB2P1 GSN-AS1 ENSG00000259868 ENSG00000274225 LINC01695 ZNF609 SIRT4 FAM24B ARHGAP33 TCF12 KTN1-AS1 LINC01356 PLEKHG2 CCNB3 CORO2B NUPR1 SH3RF3-AS1 RBMS2 COL4A3 EPM2A-DT ENSG00000273472 DNAJA2-DT UNC5C-AS1 ENSG00000232528 SYNE4 ENSG00000279080 ENSG00000285763 TSHZ2 EGR1 CCL17 MAGOH-DT NR4A1AS RPL23P2 MSI1 ENSG00000279381 ENSG00000278390 CYP4V2 SCNN1D EWSAT1 TM7SF2 MAP4K1 ACCS HECW2 PKD1L2 PPM1M PDE9A ENSG00000272372 PCMTD1-DT HERPUD2-AS1 PEAK1 TOB1-AS1 SNORC PKD1P3 VANGL2 DLG4 EOLA2-DT HOGA1 ZNF516 LINC01530 ENSG00000235119 ENSG00000285999 ENSG00000287262 ZEB1-AS1 LNP1 ENSG00000286373 SAMD9L MAML3 EDNRB ENSG00000231703 SHANK1 ENSG00000287878 ENSG00000250961 ENSG00000269925 ANXA2R ENSG00000279048 CELF2-AS1 TSSK3 ENSG00000239415 ENSG00000255182 ZNRD1ASP EBF3 ENSG00000287110 ENSG00000288880 ENSG00000215493 SFMBT2 LNCOC1 ENSG00000271259 ENSG00000269918 UACA NPIPA1 SETD5 EBF1 TMEM169 TNKS LAMTOR5-AS1 ENSG00000273355 C5orf34 ENSG00000289506 ZNF818P RUNDC3B ADAMTS20 RIMKLB CLMN AACSP1 TNS1 ENSG00000285679 SMPX PPP1R10 ZNF436-AS1 SSR4P1 ENSG00000230454 OSGEPL1 FCHSD2 SLC4A7 RN7SL3 HERC2P4 ENSG00000244055 KRTAP5-AS1 CAPN10-DT VARS2 MST1 BRPF3-AS1 JAZF1 DYNC2H1 MTUS1 PTPRVP ENSG00000270110 ENSG00000272791 SLITRK6 STX16-NPEPL1 ALDH1A3-AS1 GK-AS1 ENSG00000279926 ENSG00000279759 SNRPGP4 ENSG00000277639 ENSG00000273893 HLA-F-AS1 CHAD CPED1 HMGB2 FBXL2 LPAR2 PPT2-EGFL8 USP38-DT LINC02656 KLHL32 TMEM100 RNF165 ZMYM3 FAM200B NNT-AS1 RNF213 PRKCE FGGY-DT WDR31 SRCIN1 TIGD7 GLIS1 CACNA1D IVNS1ABP UHRF1BP1 CD37 DUSP19 ENSG00000272002 CCDC154 GRB10 TBL1X LINC02604 TMEM150C PARGP1 MCPH1-AS1 ENSG00000274460 RPS6KA2-IT1 LEAP2 SEPTIN5 ENSG00000225649 EZH1 ENSG00000242861 LNCTAM34A LINC02918 DDIT4 ENSG00000261786 CCDC140 ENSG00000287385 SDAD1P1 INPP4B ENSG00000275481 ENSG00000261799 MSH5 HIVEP2 ENSG00000279930 BLACAT1 ENSG00000272983 SOX5 KCNJ4 IQCA1 SEPSECS MTMR9LP COL4A4 PABPC1L TMPRSS5 ANKFN1 ENPP5 SKOR1 CCDC18-AS1 SEPTIN7-DT FGD5P1 LINC00920 ST20 LINC01355 GDPD3 CYP2D7 ENSG00000226744 HTR6 TMEM198B FCGR2C VPS13B-DT ARAP2 NR5A2 IGF1R EFEMP2 LOH12CR2 NLGN1 LINC00342 PDCL3P4 LINC02021 ENSG00000272425 WNT2B ENSG00000250397 EXTL3-AS1 ENSG00000261094 FRK TBX19 CDK5R1 TMEM116 PAX8 ENSG00000273261 IRF1-AS1 ENSG00000251417 ABHD12B TNFRSF11A C19orf54 ENSG00000269896 LINC02878 KRT8P12 ENSG00000269978 ENSG00000258101 ENSG00000261270 PRKAR1B-AS1 COX6B2 ENSG00000205041 ACBD4 KANSL1L-AS1 MTCYBP21 ENSG00000286248 ADAMTS13 C21orf62-AS1 AZIN2 CBLN3 LINC02289 C3orf18 DNASE1L2 CYB5RL ENSG00000289250 LINC02649 RNF157-AS1 ENSG00000279041 INSR ENSG00000260279 PHF13 ENSG00000286198 CLDN20 CIT ENSG00000197815 KIF28P MYO1F KLF15 ENSG00000274922 GBAP1 ENSG00000275180 HOXB9 BRME1 ENSG00000257176 LINC02099 RNU6-850P ENSG00000261118 INTS6L CATSPER2 ENSG00000289405 RAP2C-AS1 CDNF HEMK1 ANKRD27 TCP11L2 ANK3 SLFN5 LINC02175 MMP25-AS1 CARF GTF2IRD1 TUBG1P ZNF19 TEX21P ENSG00000261211 ANKRD34A DDR2 CFP ZNF37A DOCK3 ENSG00000264548 PRICKLE1 PDK4-AS1 HIP1 MCF2L2 RN7SL262P CD63-AS1 NKX3-2 TRPS1 GTF2IP12 ERN1 KANTR FBXO32 SNHG14 PCDHGA6 CEP57 AR ENSG00000287820 GATM ARHGEF10 ENSG00000245317 ENSG00000279811 FRG2B KLHL3 ENSG00000279838 PTPN13 VAV3 RMI2 RNF139-DT P3H3 ENSG00000287036 HCG20 ICAM5 UBE2Q2P1 POU5F1B CENPF ENSG00000232546 PRKAR2A-AS1 LINC01252 ENSG00000265298 CNOT6L CHKB-CPT1B ERVH48-1 AMZ1 APBA1 CNTNAP1 ENSG00000268912 MGC16275 TESMIN ENSG00000270116 CMTM8 ENSG00000236013 CSPG4 CYP39A1 FAM214A SIDT1 TECTA KIRREL1 ENSG00000288993 ASNSP1 ENSG00000233539 DNAI1 IGF2BP3 TBC1D8-AS1 ENSG00000286482 LINC01569 SYCP3 ITLN2 LRRC37A4P ENSG00000250041 TAGAP-AS1 ENSG00000227741 ENSG00000269954 ENSG00000261770 PEF1-AS1 BAHCC1 PAPSS2 ACSL3 UTRN CDC14A ENSG00000267248 ENTPD4-DT ENSG00000258634 TMEM240 LINC01198 GPR37L1 ZFP14 CBX3P2 SCX SCUBE3 RAB30-DT GRASLND ENSG00000279386 ENSG00000238018 TRIM2 BTN3A1 ENSG00000287997 ENSG00000280239 ATOH8 ENSG00000273442 HMGN1P1 PPM1K CARMIL1 STARD13 ZNF75A HESX1 IFT140 C14orf132 WDR19 PKD1P5 GPR19 ENSG00000254528 ENSG00000277050 SENP7 MBD5 JARID2-DT RPL5P18 ZNF467 HES7 ENSG00000223522 ENSG00000287609 ENSG00000215068 POU6F1 ENSG00000280537 AKNA HPS4 ZNF320 IMPDH1P8 IDI2 ENSG00000258744 STON1 CPAMD8 NOTCH2NLC ENSG00000280353 RARA-AS1 PCDHGC5 GDF9 ENSG00000224950 ENSG00000268858 TMEM140 ZC3H6 ENSG00000280435 LINC01786 DISC1 PSMD6-AS2 ENSG00000248161 ST7-AS1 ENSG00000271734 FMN1 CARD14 ENSG00000263551 LINC00601 ARHGAP42 SLC16A1-AS1 PLA2G6 MALINC1 ZFPM2 ENSG00000287202 CCDC113 GVQW3 BMP8A ENSG00000285804 GNRH1 FBLL1 BEX2 ENSG00000253200 ZNF827 ENSG00000241889 DSE MST1P2 GABPB1-AS1 LINC00887 ENSG00000263089 GLI1 ENSG00000274292 SLC25A34 SLC7A4 ENSG00000267404 NAV2 ENSG00000261056 IL21R RNF150 DNMT3A ENSG00000285774 WNK4 LINC00174 ZFP90 MEIOB NET1 LMNTD2-AS1 FAM186B PAPLN-AS1 ENSG00000228327 CDC42-AS1 ENSG00000258811 ZNF514 PRSS27 GNG2 HCG27 FRS3 GTF2IP13 ENSG00000198358 NBPF17P SLC6A16 ACKR2 SCARF2 NATD1 SCART1 MCTP2 |

| | | |
|---|---|---|
| | | ENSG00000288839 NLGN3 TM9SF5P TMEM178A C1orf220 CKMT2-AS1 LINC00672 ZFPM2-AS1 GPT ENSG00000257027 NNMT ENSG00000228852 LRRC39 BEND5 LINC02381 CREB3L4 SSBP2 TNFRSF25 CCR7 PROCA1 RECQL5 RGS1 ZNF862 ZNF608 ENSG00000235902 CBX3P7 SLC25A42 KIAA1109 ENSG00000259495 MOCS2-DT ENSG00000111788 ZNF577 INPP5F IQGAP3 ZNNT1 ENSG00000287837 VASH2 DOC2A MIR4453HG TPTE2P1 DBP LRRC63 ZNF563 ARHGAP32 EGFL8 RENO1 TAS2R31 CENPE TMCO1-AS1 ENSG00000253476 SASH1 SLC22A23 PCDHGB7 INSM1 ABCA10 WBP2NL ZNF493 ENSG00000289067 AFDN-DT DSTNP1 CD109-AS1 C6orf163 PRKACB-DT TBCE PLCB4 LINC02251 HOXA2 ARHGEF2-AS2 RWDD3-DT ENSG00000261026 LINC02352 LINC01909 KIF12 ENSG00000285684 ENSG00000278058 BTN2A2 MUC6 MEX3A ENOX1 SPRY4-AS1 BACE1-AS FCHO1 LINC02525 OR2L2 TRIM66 LBX2 ZNF132 CSGALNACT1 PABIR3 SLC22A15 ENSG00000262703 LINC00523 NAPSA BAZ2B C1QTNF6 CRACD LINC00858 SLC25A27 FNBP1P1 LRRC37B ZNF501 CEP170P1 ENSG00000267683 ELOVL2 CBX7 LINC01144 ENSG00000223945 ENSG00000271590 ANKRD44 CAPS2 ENSG00000255521 PCMTD1 FER GSDMB ENSG00000255557 ENSG00000270540 FAM72A CDK5RAP3 CAMK4 ENSG00000267546 ZNF606 ENSG00000278918 KRT7 ZNF169 RAC3 DLGAP5 ZNF396 SGCD ENSG00000286757 LINC00339 LIMD1-AS1 NKPD1 ENSG00000262691 LRRC20 ENSG00000232611 PCYOX1L LINC01447 GLI2 ICA1 NAV3 ENSG00000288748 ENSG00000273374 PRIMPOL ENSG00000198580 NLRP1 TGFBR3 CCN1 LINC01583 GRIN3B FRG1GP ATXN1 APH1B ENSG00000273203 TPM2 CT75 ENSG00000272502 ENSG00000279518 S100PBP ENSG00000280157 SGMS1-AS1 SCARNA7 KATNAL2 SPRY4 ENSG00000279833 ZKSCAN8P1 ENSG00000285943 ENSG00000270171 NKILA CUTALP ENSG00000222044 SHISA7 ENSG00000237807 ZNF620 LINC02728 SMG1P7 ENSG00000285184 LINC01341 PLEKHG1 LINC00638 ATP8B3 SNX18P1Y SNTB1 SLC20A1-DT ENSG00000251143 CHIAP1 LTK ALKBH6 JDP2 C13orf46 P2RX7 ENSG00000253854 PLEKHA5 TBX6 SPTBN4 ENSG00000244560 EPHA10 GSTM2 IGSF22 SLC35E2A BACH2 NALT1 PHACTR2P1 COL27A1 PPP1CB-DT PHLDB2 FXYD6P2 ESR2 MIR3189 ENSG00000238260 ENSG00000277007 ENSG00000261474 ENSG00000277287 ZNF45-AS1 ENPP3 SATB1 YPEL3 XYLT1 ENSG00000272720 ENSG00000260855 BCDIN3D-AS1 ZNF471 ENSG00000272529 LINC00847 MYADM PPP3CB-AS1 L3MBTL1 ATXN7L3-AS1 MEIS1-AS3 MOB3B ENSG00000284526 PCP2 ENSG00000271971 ENSG00000212978 ENSG00000272264 ENSG00000272054 ZSCAN18 ENSG00000278869 SLC28A3 RASSF3 ENSG00000288884 ENSG00000276317 KCNAB3 SYNE2 ENSG00000225931 TUG1 ENSG00000267868 KEL C22orf46 ENSG00000280399 ENSG00000277938 ENSG00000286753 B4GALNT4 LINC02343 MDGA2 ENSG00000285725 NAV2-AS6 ENSG00000279161 PIGAP1 CLDN4 ARHGAP30 ENSG00000268970 ZNF277 ZNF704 NMRK1 SH3GL1P2 NEURL2 GABPB2 ENSG00000277496 SYPL1P2 LINC00173 LINC00898 COL15A1 LMLN ENSG00000260285 CHMP1B2P HSD17B1P1 CCDC26 TEAD3 FAM72B MYO5B MATN1-AS1 FAM86B1 ENSG00000232098 SEC31B NUDT7 ENSG00000278041 ENSG00000259820 CCDC122 NTNG2 ENSG00000286174 HOXB3 ENSG00000261468 KLHDC7B ZNF716 ARHGEF2 CDC25C PAXIP1-AS2 ENSG00000177788 ENSG00000280149 LINC01517 CCNG2 ENSG00000286985 ENSG00000251194 WDR88 C15orf65 FAM86B3P HOXB13 TMEM44 ODAD4 LINC01366 LINC01572 ENSG00000267681 ENSG00000227329 LINC00565 ENSG00000289378 HDAC9 DLX4 HSD3BP4 ZCCHC18 KIAA1549L MYT1 ENSG00000233817 ENSG00000280332 ENSG00000258853 MAP2K6 AGO4 ZBED3-AS1 EN2-DT KAZALD1 ENSG00000276564 C11orf71 CBLB UGDH-AS1 ENSG00000286535 TEAD2 KLLN LYST RAD51-AS1 ENSG00000264112 ENSG00000259341 STPG3 GCAWKR UNC13D LMOD1 NUDT6 CSRP1-AS1 RGL3 PRKCZ-AS1 ZNF221 BEST1 ACRBP RBM20 ENSG00000267666 CPT1B SRGAP2 ENSG00000285980 SPATA6 SUCLG2-DT IHO1 CNTNAP4 ENSG00000287737 TUBA3D PDGFA-DT AGPAT4 NOX4 GTSE1-DT LINGO1 SALL2 ZNF546 CYP2E1 NRG2 CTH ENSG00000279315 GABARAPL1 APPBP2-DT ENSG00000289042 ENSG00000269397 ENSG00000255028 ENSG00000253573 AHSA2P OBSCN ENSG00000259668 ENSG00000260077 DEF6 RPH3AL PLEKHH2 SNRPGP14 GLI3 PTCH1 ENSG00000261959 FOXD2-AS1 ENSG00000288756 ENSG00000287729 ENSG00000267199 PHBP13 ENSG00000273373 TNFRSF13C INTS4P1 MST1L GAB2 LINC01249 ENSG00000241886 ENSG00000289318 RGS9BP ENSG00000197813 ANKRD31 C16orf86 ENSG00000286220 AGAP2 LHFPL3-AS1 ENSG00000275910 ENSG00000253395 ERVW-1 ENKD1 CORO6 ENSG00000279360 LINC01531 FBXO43 CHAC1 MAST1 LTB4R CREB5 SHF ADGRG6 PLIN1 ENSG00000284634 SPTY2D1OS MIR3936HG ENSG00000278998 ENSG00000093100 ARSI MIR3142HG ZNF610 LINC01625 PDE4DIPP6 TMEM51-AS1 ID2-AS1 PCDHGA2 OGT KRT8P46 FRG1DP BSG-AS1 ENSG00000259772 EPB41 SCAPER ENSG00000216775 ENSG00000279672 CLDN15 DOCK9 ENSG00000259088 MKRN2OS YPEL4 ASPM PPARA TLE1 NCKAP5 ENSG00000260604 LINC00933 ENSG00000257252 PRIM1 ERICH2 ENSG00000261476 MAP2K5 PLCB1 ADM2 RPS10P7 ENSG00000223947 MPRIPP1 GRID2IP PARP11-AS1 ENSG00000286017 ADRA2B PCF11 RETREG1-AS1 TRIM7 CCDC191 PCSK4 ENSG00000261114 ENSG00000287957 CACNG8 ENSG00000259065 ENSG00000271833 SGCA EXPH5 SLC16A13 PLCL1 TEX22 DDIT3 ENSG00000287236 STK4-DT EDA CNTRL IFIT1 ZNF460-AS1 DENND3 ENSG00000282897 ENSG00000262580 ENSG00000267504 DOCK10 GNMT EPG5 COL2A1 CNTNAP3 L3MBTL2-AS1 ENSG00000236529 ENSG00000286485 LRRC4B RBM47 ZNF674 RAPGEF3 ENSG00000289161 FBXO41 ITIH2 PRR5L TTC21A SLC12A5-AS1 ENSG00000285925 ENSG00000278635 LINC00205 ENSG00000272668 ENSG00000268575 HOXB6 ZNF528-AS1 ENSG00000255026 GNAO1-DT SLX4IP SEMA3D LINC02615 ENSG00000289370 ENSG00000287650 ZEB2 ENSG00000279588 DBIL5P2 JMJD1C CICP14 PAIP2B HJURP ARID4A OR1F1 RHOQ-AS1 CREBRF IFT81 KLC2-AS1 ENSG00000251364 NBR2 ZNF93 ENSG00000280128 ETFBKMT BTBD19 |

| | | |
|---|---|---|
| | | *ENSG00000267277 MTSS1 SLC9A3-AS1 WNT5A-AS1 HMMR MORN4 CEP295NL RN7SL67P CCDC146 FAM227A ENSG00000272405 CDK19 GOT1-DT RHOBTB3 PKDCC ENSG00000280225 CCDC171 FOXP1 SCN8A FBXO48 PAPLN PAX3 TMCC1-DT SLC16A8 ENSG00000284968 BMPR1B ENSG00000243243 GATA6-AS1 PLAC4 DUSP16 ENSG00000287910 LINC01234 LINC00648 ZRANB2-DT AGAP1 GPT2 PTK6 RIPOR2 THBS3-AS1 ENSG00000227775 ENSG00000235381 BCAT1 PARD3B ENSG00000280061 SMG7-AS1 ENSG00000284959 ENSG00000223393 ENSG00000286545 GAB1 ENSG00000267649 MYH7B JPH3 C20orf204 HSPG2 C21orf58 CYP24A1 ATP10B RFTN2 TNS2 C2orf74 SEMA4C CCNYL2 EIF2AK3-DT PDGFC ENSG00000268204 CSPG4P13 C8orf44 ENSG00000275719 PHF21A DACT1 ANKRD23 RNF32-AS1 FRMD4B N4BP3 HCG25 GOLGA8B OSBPL5 CDHR2 PIK3IP1* |
| 976 up | 920 | *PNMA1 GNL3 IFNA20P PDCL3 STOX1 AEN IRAK1 BRIX1 COPRS KBTBD8 PNPT1 CCDC97 LYAR RRP7A MRPL1 UFSP1 CMPK2 ITGA3 RABGGTA POU3F1 GRPEL1 RELT LMNB2 ENSG00000279692 EOLA2 CAVIN3 GFPT2 PPIL1 FAM98A PGBD5 H2BC21 ALG2 DUS3L PRR19 PMF1 MIR193A GCLM METTL25 ID1 TIMM22 ZNF35 PSMD11 ELAC2 EMC8 LINC01679 ZNHIT2 NCDN DOHH SETMAR RFX5-AS1 ENSG00000261762 ABCF2 ASNSD1 HGH1 TMEM11 MRPL15 H2BC11 COMMD5 ENSG00000256955 SLC9A3R1 PRR5 CHMP7 FKBP4 LONRF2 SF3A3 ENSG00000267405 BST1 HCFC1 ACTR5 KRT15 EIF4A3 PXN-AS1 LINC00475 IFITM10 SNORD88A PPP1R14BP3 H2BC18 CHMP6 CYP19A1 MANF EIF4E2 ENTPD7 FAM174C ENSG00000275106 FETUB MRTO4 C1QBP ENSG00000275993 PSTK PAQR3 NECAB2 ENSG00000267505 SCRN1 UCN2 ZNF511 IRF7 HSPA2 EIF2S1 EIF5A MCM10 DNAJC25 ENSG00000223461 E2F4 PSMD12 ENSG00000289117 PODXL CSRNP1 ZBTB7C NSUN5 SF3B5 ZNF18 TOMM22 CHPF2 MCAT VCP GAL RAVER1 PDXP GCLC ENSG00000257497 SEC61A1 ENSG00000279212 GALNT10 BANF1 PES1 WFS1 H2BC4 H2BC12 INTS7 AP1S3 ENSG00000286181 MAN1A1 PYCR3 CCDC103 ASH1L-AS1 ABHD5 CBWD1 GPS2P1 ZPR1 ENSG00000280486 LINC01979 CYRIA PRMT5 DCAF4 RNF126 RAP1BL SHQ1 FASTKD5 ENSG00000280010 IPPK EFHD2 SESN1 PUS3 NKX3-1 WDR74 ABCB9 NECTIN4 ABCF1 UFD1 MRPL14 PSD4 MPHOSPH6 TIMM23 PSMC2P1 DEGS1 NUDC EIF3J SNX11 CKMT1A LDHAP7 FARSA H2AX SLC30A1 TRMO KLHL18 CCNE2 TBX3 SWSAP1 FUT11 GPER1 C15orf39 MYDGF CCT3 NCL PCCA-DT MCM4 ID2 DDX21 KTI12 MESP1 CANT1 SLAMF7 ENSG00000227218 POLR2A SEC14L2 ENSG00000263823 RPUSD2 EMC6 MED31 ENSG00000279539 CA8 BRMS1L UQCRFS1P1 BCL7B SHPK C14orf119 C1orf53 TMEM41A EHD1 CDKN1A ASH2LP1 TRIML2 ARPC5L RPP25 MAGEA12 CIAPIN1 PTRH2 SNORA33 ZNF668 STAMBPL1 SRM AKAP8 ENSG00000286996 ZNRD2 SEMA3F TOMM40 RPL23A ENSG00000215014 MARS2 NFKBIA SDF2L1 MRPL36 MON1A TTC4 WDR3 SNORA73B MT2A ALG1 PRCC DDX56 GMEB1 ENSG00000263826 DDX20 UTP15 MYBBP1A CCT6A RCBTB2 H1-2 PNP ST6GALNAC1 METTL1 ANKRD37 CHST8 SNORD83A EIF6 SEC24D POLR2L MRPL4 LINC02119 ATP6V0D1 POLR1C ID3 TM9SF1 SPATA5L1 UNC5B L3MBTL2 AKR1B10 RARS1 DOLK RPP40 YARS2 HMOX2 ALMS1-IT1 TAGLN2 ATP6V0B PELO MRPL12 CCDC137 CKB PSMB6 CCT2 SPRTN SYN1 LETM1 SLC25A44 VSNL1 PRDX1 H2BC5 LSG1 MERTK PUS1 ENSG00000279467 OLFM1 TUSC2 SRP68 NRBP1 SLC25A22 ENSG00000255224 RRP36 MFSD2A TMEM47 CNTF FA2H ENSG00000261737 ENSG00000279605 MPV17L2 MEPCE RRAGA ABCE1 LDHA SPHK1 ENSG00000274270 NOP16 INPP1 TMEM214 IER5L WDR4 RRS1 HSPA1A LRRC15 POLR3E OGFRP1 DHX37 C3orf80 FTSJ3 ENSG00000233388 RRP1B DKC1 THOP1 TRIAP1 ENSG00000213087 ENSG00000265749 MRPS26 DHX34 BRINP1 HSPA8P9 MT1X KEAP1 NOP56P3 EEF1A1P8 ENSG00000267397 NUP50 ARRDC4 ENSG00000279641 RN7SL832P SRP19 DIMT1 PYM1 TXNIP SRSF8 PTBP1 NSDHL LINC02735 LYPD1 DHX9 SMIM24 ENSG00000233825 PHRF1 IQCN ABCG2 MRPL18 PGAM5 RBM12 PECAM1 PRPF4 NOCT DDX28 UBE2S CCR1 NCS1 POMP NAP1L3 GMPPB AHSA1 MGAT2 NOP56 TATDN2 SYT6 TAF13 NUBP1 SHLD1 EIF5 PREB FHOD1 GTPBP4 SPNS2 UTP3 C16orf91 MUL1 TPT1P9 TUBB6 DCTPP1 FDXACB1 NUP153-AS1 CLU DPH2 CORO1A ZNF784 NCBP2 HNRNPAB ENSG00000264985 GADD45GIP1 FEN1 EFNB2 BAG2 ELOF1 GLYR1 LRWD1 LRRC59 TXNDC9 TRAC RTP4 CEBPA TMEM201 SURF6 RRP12 NDUFB2-AS1 HSPH1 CARD8-AS1 ACTB SLC39A3 NSMCE3 FAAP100 DSEL ENSG00000237493 BCL10-AS1 PRELID1 ATP6V0D2 NFKBIE TSR1 PRR7-AS1 ENSG00000283064 ENC1 PNO1 PIGW RASL11A ENSG00000261888 MIR22HG LHFPL3-AS2 TRMT10C TNFSF9 ENSG00000279133 PDCD6IP-DT TOE1 TM4SF19 ZNF341 CALHM2 CRISPLD2 DDX3X PGD RND1 UBE2J2 GNL2 PDE12 LIPT2 KCNJ2 UTP20 ENSG00000264577 BYSL PDF PMPCA ENSG00000289164 PRADC1 SSC4D CYCS CLUH BAK1 ENSG00000264666 ZNF576 PHACTR3-AS1 NOC4L POLR3K PRDX1P1 PUF60 BLOC1S2 RIOK1 CACTIN SNHG25 SDAD1 ENSG00000279249 KPNA2 KPNA3 CHRNA5 ENSG00000279443 ENSG00000248968 ATP5MC1 TMEM250 BCLAF1 TPI1 IPO4 SRSF6 POLE3 BOP1 PLIN2 FAM98B HSP90AB2P HNRNPR PSMD3 EEF1AKMT4 MRPS12 C3orf52 ENDOG FGFR3 PTGES ENSG00000279965 CDC6 HSPA8 LYSMD2 MAFA SAC3D1 FXN NOC3L RABEP2 KRT17 RBM38 RDH8 ALYREF ZNF672 HEXIM1 RPL5P8 SNHG9 LANCL2 S100A3 GRWD1 TIMM17A PDSS1 SMG8 PGAM1 NGFR SHLD3 SEC13 PSMC4 PTMAP4 SERTAD1 HRCT1 FDX1 ENSG00000217275 MAP1A TRPM2 NOL11 PSMD2 USP18 POP7 ENSG00000262140 SURF4 BCAS2 TCF15 LDHAP3 SRRT SNRPG RBM3 BMS1 GEMIN4 SELENOS CTU2 CDR2L POR MPDU1 NAA15 LCMT2 MRPL9 RUNDC1 ABT1 RCC1 ENSG00000261889 MBLAC1 B9D2 HYAL2 ENSG00000279953 ENSG00000249050 SLC2A1 INTS5 TIMM8A MMP11 NUP35 NCOA5 CTR9 HAS3 SRCAP UBASH3B HMOX1 RABIF SERPINB8 U2AF2 ZMYND19 TUBB8P7 TSSC4 AAR2 ZNF582 TMEM70 RBM14 COA4 YRDC OSGIN1 ADAMTS15 SDHAF2 RRP1 FSCN1 RINL NOC2LP1 CTLA4 GLMN CCNQ CFL1 TAX1BP3 LINC01615 ENSG00000260136 DYNLL1 CTPS1 ENSG00000286570 EMP3 TUBA1C SNRNP25 DPF1 STIP1 CHST7 CSKMT S100A2* |

| | | |
|---|---|---|
| | | *NOP14 COPS3 DMRT2 SOCS1 NPLOC4 TRIM28 PHLDA2 TST POP1 COA7 UTP11 MRPL27 RIOX1 SMTN CYB5R2 DNTTIP2 ISOC1 SCO2 CLSPN LENG1 TMEM115 ZNF628 EXOSC4 POLR1G SRA1 BRD2 LAMC2 OLFML3 FAM118B USP5 ADM LAP3 TUBB4B TIMM23B IMP3 MIRLET7BHG CAPN15 SBSN PTMAP5 ZMPSTE24 H3C3 PSMB3 AP5S1 ENSG00000287721 MED19 ENSG00000271851 ARMC5 BEND3 KRT12 RSL1D1 NAT1 ZFY MIEN1 CALM1 PLEKHA6 C8orf33 NME1 FAM222A TMEM11-DT ENSG00000276931 PIM2 CPNE7 GINS2 NOP56P1 MIR210HG PVR MRNIP S1PR3 STEAP1 BLOC1S4 SIAH2-AS1 LTV1 ENSG00000232748 ENSG00000288873 RNF25 JPT1 MSC ATXN7L2 PTRHD1 NOSIP EIF3B RPL36A FASN ILF2 SMIM3 SQSTM1 HPS6 C7orf26 H2AW PUSL1 BRMS1 XIRP1 PRPF19 ENSG00000260912 ENSG00000289554 HM13-AS1 MRPS17 MRGPRX4 ENSG00000259704 ENSG00000276853 NOP2 FAM135B ENSG00000273568 CLP1 TMEM273 ENSG00000266651 ENSG00000178412 DHX30 IGFL2-AS1 MRPL54 PPP2R1B RRP9 LSM10 TUBA1B CRY1 MSX2 SCAND1 FBXO33 LINC01465 FAM169A ENSG00000222032 CHCHD7 SLBP CARD6 YJU2 NPTX2 RPUSD1 RAB27B RPLP0P6 SMIM12 LRATD1 HSPA8P1 CALR SNRPD1 BZW1 PCNA GPATCH4 FER1L4 CHUK HR POLH-AS1 CCDC86 STX11 DNAJA1 ALDH1B1 PLEKHA7 CYB561D2 SZRD1 KDM8 NOC2L ATF5 PDCD2L CCNYL1 ISG20L2 ENSG00000274015 NT5C1B PRMT6 ICOSLG GABPB1 UTP14A NXF1 MRPL17 ZNF653 ADRM1 AIMP2 ENSG00000240652 INO80C ENSG00000272990 KPNA1 TAF6L TMPRSS2 NXT1 DGCR11 ENSG00000272768 ENSG00000278974 SNORD12C ADPRS GAR1 IDH3A HSP90AA1 LRRC47 PHF5A PGP CBLN1 TYROBP NIP7 TMEM158 HNRNPD-DT DPP3 PDE2A TUBB2A TBCC PLK2 TFB2M NUP188 SRXN1 LINC02577 TRIM21 C12orf43 MB ANKRD30B ARRDC3 TCAF2 XPA ZBTB9 DDN MSX1 AUNIP EPHX1 VPS18 SELENOTP1 SLC27A4 GEMIN7 PPIF VPREB3 ENSG00000260273 PRR7 SRPRB ADAT3 NOL6 SLC25A33 MYH7 PTS DDX54 MROH9 GLRX5 ENSG00000259041 PA2G4 MRPS2 POLR3H RILP ENSG00000289307 ACER2 SRSF2 SERPINE1 NAP1L1P3 SMN1 ARC PDCD11 EXOG C8orf76 ENSG00000272604 PKM HSPA1B DDX23 GAPDHP63 SPRR3 NOLC1 VWA5B2 HSP90AB3P ENSG00000286064 UBIAD1 SNORD104 NPTX1 FAM217B SCAMP2 ENSG00000228477 RGMA GMNN KRT10 CCT5 ZNF295-AS1 FDXR MCRIP2 TLCD3A PSMD7 SNRPB MICALL2 TIMM21 CCN3 TLCD1 SF3B4 PSMC3 MFSD5 TMEM199 EXOSC6 TACO1 NUDT15 ZNFX1 KCNK5 DCTN5 PSME3 FUNDC2 TMED1 GEM CCL3 ONECUT2 ENSG00000277117 CCDC71 FAM180A SLC52A2 LINC00973 RAB5IF EIF4A1P7 SLC10A3 SLC25A5-AS1 PNPLA2 CFAP45 SPOCD1 ZNF622 ENSG00000250031 POLR3B* |
| 4C decr-3331 | 3053 | *MSRB1 AL157886.1 FSTL1 CDH12P2 CASC20 C10orf90 AF240627.1 MEFV BBOX1 AC243829.5 LINC01889 PFKP ABCC6 FLJ40194 MIR3171HG AC012501.1 ESYT1 AC025423.2 LOC101928894 STYK1 RPL19 SMIM17 HS3ST3A1 RTN1 RNU7-188P AC100775.1 AC012409.2 RGN TRGC2 ZSWIM1 LOC105375623 AC008667.3 MSRB3 EPN2 ENSG00000293331 MREG HNRNPCL4 NOSTRIN ENSG00000290149 BANK1 NINL MAP3K3 WSCD1 ENSG00000287515 OSBPL8 SLC12A8 LRRN1 PCMTD1P7 SRPK1 AC125634.1 HERC2P2 ENSG00000286309 SYN3 LINC00210 ENSG00000291144 LINC00698 POTED SUGCT AL133216.1 AL353626.3 STX8 AC032019.1 FP671120.1 HCG24 AC243562.2 LOC102724642 FOXK2 PHF20 CPNE4 Metazoa_SRP TXNDC8 TMEM266 RBM17 AC021517.1 ENSG00000290700 SMYD3 GARS1 RAPGEF6 ARHGEF9 TAFA2 ABR PAK6-AS1 AC090696.1 KRT8P32 TLR8-AS1 ISM1 PABPC5 BLTP3B LINC01163 AC012355.1 CDK8 LINC02438 MRGPRX11P HYDIN2 ENSG00000287308 AC011477.7 HCCAT5 TRAPPC9 PCDHB1-AS1 SLC16A5 FAM182B ENSG00000287879 LINC01545 C8orf44-SGK3 FNDC1 TMEM255A PREP AC092138.2 CBLL1 MAP3K13 EIF1AX PPM1A BCL2 ARL13B RPL15P21 ZNF239 SAMSN1 RN7SL556P AL133257.1 C12orf75 AC010291.1 THADA LIMD1 XRN2 IMMP1L TF SNX10-AS1 CHST11 MYBPC1 USP42 IPP CNOT1 RCSD1 RICTOR SPTBN1 UBTFL3 AF228730.2 SAMM50 CDC42EP3 ENSG00000286111 AC013472.2 AC021269.2 AC244035.2 UNC13B MIR3117 MYO5C LINC00841 ITPRID1 USP32 HNRNPA1P42 AHCYL2 DAPP1 CD101-AS1 ATF7 Z68871.1 GNG12 FGF1 IL6R CHRNB2 CDC14C AC104455.1 SEZ6 MIR153-2 CLDN6 LINC01967 CDH8 ARHGAP15 GNB4 AGTPBP1 AL356490.1 ATF7-NPFF LOC101929710 FHL1 HK3 PPP1R37 RBPMS ATP10A REXO1L5P TTC36-AS1 LINC02457 SH3PXD2A SPINK5 MRPS24 OPA3 SKAP2 PRND PIGL DYRK4 ATP7B ENSG00000291259 ABCC6P1 PRCPP1 AC018442.2 RIC3 PPM1H GPBP1L1 RPS6P19 KCNJ6 KCNK10 AC016550.2 ETFA EXOC1 RPL21P82 BRD4 ZAN DOK4 NPFFR2 AC007405.2 LINC00517 GOLGA8CP AC092378.1 ANKRD26P3 AL137802.1 ENSG00000288005 AKIRIN1 SLCO1B3-SLCO1B7 TNRC18 MIR9-3HG AC084211.1 LINC02828 ZNF568 ADCY3 CAMK2B CASC19 ELMO1-AS1 ZNF962P OR4N3BP ARHGAP11A-SCG5 ARHGEF26 TLK1 LOC124901321 CRAT37 URGCP-MRPS24 ARHGAP11A-DT DDI2 AC008764.4 GPATCH8 PGM1 CPD DCT AC073071.1 TANK AC073834.1 FAM193A GOLGA8EP ENSG00000286515 LNCATV PECR LRP5L SLC38A9 LINC01982 UBE2E2-DT GPR135 CDRT15P9 LINC00348 ZNF264 CBR4-DT QRSL1P2 LINC02133 HBP1 PLA2G4C SDR16C5 RGPD2 ABCG8 XPO4 OTUD7A KALRN AC060234.3 AL583785.1 AC007221.2 AC090772.2 AC107909.2 USH2A SUMF1 OPN3 MIR6129 LINC01378 UBE2Q2P2 MAP4K3 NUP133 AC092078.2 XXYLT1 FAM157A ENSG00000287286 LINC01194 LOC101929200 CASC9 GALNT9 FPGT-TNNI3K SLC16A2 AC099499.1 FMO9P SNORD114-10 LOC442028 NOTCH2 SULF2 CD93 LOC105374069 LOC105379362 FKBP5 SMS TAF1D LINC03062 TMOD2 SLC44A5 ALG9 AC092423.1 MEGF11 SDK2 MELK AC093423.3 HMBOX1 SIM1-AS1 ENSG00000290766 AP003900.1 ZNF551 LINC00882 RNU2-49P NPNT AC019211.1 DSC1 EPB41L3 SRRD NUTM2B-AS1 MAP6 AC012363.2 PARVB MORC1 DEFB130A CDH11 AC066613.1 AC108066.1 RN7SL266P PYDC2-AS1 PCNX2 SHANK3 KANK4 ENSG00000286329 OR7E5P WDR26 NR4A3 CYP2U1-AS1 SPTLC3 GAS1RR RLIG1P2 AGAP12P OR1B1 LINC00494 AC005154.4 AP001605.1 LOC101927948 LOC105375387 DPY19L2P2 ENSG00000289752 AMYP1 E2F7 ACVR2B-AS1 L3MBTL4-AS1 SLC14A2 AC127024.8* |

| | | |
|---|---|---|
| | | ENSG00000291166 PCYT1B AC091304.2 FAM169BP KIR2DL1 LINC01111 FOXO1 CBLN2 LINC02311 PPP1R17 ZNF611 HNRNPA1P30 RPL10P1 STYXL1 AC244517.9 LINC02156 AC098588.1 STK39 PDE11A-AS1 SMURF2 SRL MRO ADAMTS12 DAAM2 AL591501.1 ZFYVE9 FER1L6 TBC1D16 ZNG1A PLXNA4 AL592078.1 RN7SL678P LINC02542 AC008667.4 LINC00504 LOC124900810 CDK18 YBX1 STK24 SCTR ARMT1 DIPK2A CFAP70 DCDC1 FAM20B ENSG00000287410 AC046195.1 FAM3C AMPH SNORA5B ASB4 IDO1 HNRNPH1 MSC-AS1 AC015849.3 AC026786.1 AC015922.1 ARB2A LOC105370489 PRKN ENSG00000286937 U8 NLGN4X ALOX12 AL160272.2 EXOC4 TMBIM1 RMND5A BPIFB1 LINC02044 HSD17B2-AS1 EMB SLC35F5 PRDX3P3 PNPLA3 LINC00320 AC015845.2 AC092447.10 F13A1 ENSG00000288041 XPO6 PIK3C2B AC103876.1 KDM7A SLC44A3 SLC5A7 SLC31A1 GNA14-AS1 MARCHF9 FAM83B IFT172 AC011477.6 TMEM182 AL136317.2 RNPC3-DT RNU6-258P MUC12-AS1 GOLGA8Q CHD7 LIFR-AS1 GPSM1 AL121900.2 LOC121725015 EFL1 CBLL2 AL136372.2 PTBP3 ARL17B ZBTB44-DT AP000561.1 FAM117A RN7SL113P AC112128.1 CDON DEFB130B SLC17A6 MDM2 NLK SDF4 TDRD3 AL133370.1 ITPRID2-DT AL137918.1 THSD7A OVCH1-AS1 GNAI1 IZUMO1 LINC00334 SPATA31D2P MRPL13 AC138932.2 MAPKAPK3 NCAM2 CHML PAPOLG ITPRID2 H3P38 TIAM2 CFAP54 LOC107986178 ZNF10 CFAP210 RORA-AS1 AC015804.1 RMDN1 TRAK2 AL139383.1 ENSG00000293038 SYF2P1 CNMD ENSG00000286229 AP003100.2 SMARCA4 HERC2P3 LRP8 AC018767.3 TAGLN3 LINC00992 CNTN1 AC002451.2 AGL MEOX2 SGTA MAPRE3-AS1 STAT5B ENPEP LINC01242 NUDT19 AKAP3 ADGRG2 SLC22A13 DEFB115 ARHGEF4 LOC102724428 UBE2O MUC17 TNFSF8 LOC105375421 MTUS1-DT AC025884.1 LINC02448 OR4G6P GOLGA8K DUXAP9 C5AR2 AC092100.1 CPEB4 PPP1R36 AC103681.2 SCLT1 LITAFD EIF4ENIF1 HEATR9 TXNRD1 LOC124900848 TNFRSF21 CALCR ENSG00000286479 CHRFAM7A RPL23AP27 ELMO1 PPP1CB ERBIN RN7SL430P CR383658.2 ZNF121 HERC2P9 TRIOBP ENSG00000287881 MTHFD1L MBTPS2 AC092535.1 RGS22 PLS3-AS1 LINC00598 AC006511.5 CD247 ENSG00000290948 ENSG00000287621 MCC RNF111 TRIM52-AS1 IST1 PTH RAB22A GEMIN8P1 ENSG00000287008 ATXN1L ROCR ADAR DNAH10 PDE10A TM9SF2 ENSG00000286250 TOX2 ENSG00000287725 PDHX CLCN3 RHOJ ESYT2 GNG7 CDC37 PGR FGF10 ZFYVE16 JAZF1-AS1 MECOM LINC03104 POTEM FAM182A AC009878.1 L3HYPDH RSPH10B COL4A6 CATSPERB TSPAN18 NLRP8 ENSG00000286800 STK32B PLG NEURL4 POLR2J3 HERC5 LEKR1 CNTNAP5 LINC02505 LOC124900957 AC008109.1 CDH7 LOC101929457 AC008914.1 AC018618.2 LOC122455341 CHIC1 ENSG00000290585 PGPEP1L UBE2E2 SCEL-AS1 ILDR2 AC034229.1 C13orf42 MGC27382 AC145543.1 NCK1 MTERF3 AC074132.1 ENSG00000287058 CD84 ENSG00000287744 FAM168A OR4G4P NONO RARRES1 AC138512.1 KLHL42 MBP NREP AP000820.2 AF064860.1 TANGO6 EXO1 GABRA5 CCDC71L AL591684.2 ARHGAP23P1 RPL9P12 DOK5 TET1 AL512380.2 NDUFA12 SNORD114-9 ZNF737 DYNLRB2-AS1 NEXMIF ENSG00000286274 LOC401913 ELMOD3 AC093227.2 ENSG00000288755 MTCP1 BLZF1 SH3BGR TRAJ17 MROH6 SUZ12P1 AC112242.1 AC104248.1 TMEM135 MED26 AC004492.1 AC231532.1 KDM2B CHRM3 IGBP1P1 HEY2-AS1 PREX1 RGPD3 PRIM2BP AL133500.1 RFFL LINC02177 AIG1 ERICH1 AC006330.1 IGLV1-41 ZNF404 PMS1 MIR2052HG SCOC-AS1 WRNIP1 ST3GAL5 ABCC13 TYW1 ANO3 ZNF821 RPS13P2 GOLGA8R RPL5P31 AC009511.1 TMEM132D SUCLG2 COL11A1 AC119677.1 AC112721.2 AC026150.3 SNORA72 AC113391.2 RSF1 C2CD5-AS1 AC022509.1 NCOA7 KCTD3 AC063949.2 RNU2-33P SERTM2 RPL9P8 ZSWIM3 SEPTIN10 AC097532.2 CIDEB MARCHF1 BPI OR4N3P ZNF734P AL353612.1 ENSG00000286376 SLC45A1 ENSG00000286922 TTC33 MCPH1 AL773545.1 ENSG00000289143 LBH ENSG00000287045 MYO1D LINC01707 SETD4 SESTD1 ENSG00000291338 LINC01661 ENSG00000288553 ZNF214 BRAF TWIST1 HORMAD1 COL1A2 DTNA ENSG00000290095 ENSG00000286020 TMOD3 LOC340512 UBR4 KTN1 MRTFB ENSG00000286147 XPO7 GSTA10P KCNS3 JHY AC011447.3 ENSG00000288902 VWA3B RPL34P11 FREM2-AS1 ENSG00000287608 ENSG00000290114 STEAP2-AS1 RN7SL418P ABLIM1 ENSG00000287763 AC009226.1 AC110792.1 RPL26P26 RBMS1 UGT2B28 ALPK2 CHRNA7 ARL6 HECW1 HMGB1 AC125613.1 ZNF304 ENSG00000286328 DYNLT2B AC021733.1 CDIP1 RAP1GDS1 PALS1 IGHVII-22-1 LINC02388 CHPT1 RFLNA AC091965.3 ACTR3 AL158198.1 AC062039.1 HIVEP1 ATRX ENSG00000293389 STK24-AS1 UBTFL5 TGFB2 LOC105371956 ENSG00000290674 MDFIC LAMA2 ADD3 LINC02226 TRIO ATXN7L1 ZNF385D FBXO16 ZNF85 C8A LINC00269 ENSG00000291054 ATP5PBP5 AC245060.6 LINC00366 HOXC13 IGHV3-72 AL049875.1 SMARCC1 AC245427.1 AMBRA1 REXO1L10P DSP RAD51B NDST1 UBE2FP3 LINC00355 MUSK DERA ENSG00000287722 KRT223P ZNF73P KCNJ18 ECPAS LINC01755 JAM3 ENSG00000287741 KCNIP3 AC027338.2 PNPLA4 AC004485.1 MAP3K5 ALKBH1 AL132656.3 SNORA5A ARPP21 AF130359.1 ABCG1 RGS3 ZNF299P DUXAP8 SCOC AP001464.1 LDHAL6DP NUP93 AC108673.3 ZCCHC4 MRPS9-AS2 LINC02858 ZNF407-AS1 RERG G2E3-AS1 LINC02445 ESRRAP2 OR5AU1 PRKAR2A RBM33 FILNC1 AL604028.1 CAMSAP3 ENSG00000286622 TBC1D5 CFAP57 NOVA1-DT ENSG00000286902 MYO6 FADS2B CALCRL-AS1 ATP11A AC023389.1 MRPL33 RBM23 AC008695.1 ZNF154 KIFAP3 ENSG00000289748 LINC02303 LINC01340 CSTF3 BORA SLC15A4 HIGD1AP9 PRTFDC1 ENSG00000288087 ENSG00000288031 AL024474.2 Z73965.1 LOC105369165 ENSG00000286218 GSTA9P AC109361.2 ATP6V1G1P2 AC244517.11 AC013652.1 ENSG00000293012 CERS6-AS1 CDH12P4 PCDHB8 GOLPH3 AC015908.7 AC090679.1 SLCO3A1 MBTPS1 NCF2 SLC26A5-AS1 PIK3C3 RN7SKP94 SNORA40 GRK3 RNU6-1216P PIGV TRAF3 SND1 USP10 PTPN2P2 EPIC1 CHD3 AC073575.1 GLUD1P4 ENSG00000288692 CD40 EFCAB7 RN7SKP141 RALBP1P1 GSAP SPICE1 IGFBP7 PRMT2 ATP6V1G3 EPHA5 NMNAT1P1 MACROD2-IT1 TMCC2 RABGAP1L-DT LINC03076 PTPN20 AL353626.1 ENSG00000290849 AC087501.3 POLR2CP1 LRRK2-DT SKOR2 AC130650.1 CBR4 |

| | | |
|---|---|---|
| | | *CRLF3P2 ENSG00000289694 GRIK4 ATP13A4 AL162718.1 RUNX1 AC009055.2 ENAH ZNF765 ATP2C1 SNX10 RNU6-785P TMEM230 AL354718.2 RASEF PPTC7 PTGFR GRHL2 AHI1-DT IGHV1OR21-1 AL138895.2 RNA5SP114 AC009403.2 FAM171A1 STRADA PCLO LINC01725 HUWE1 ZNF595 POLA1 ASPH AC087477.2 RRAS2 SH3BGRL AC012462.2 ZDHHC4 AP000894.1 AC114781.3 ST8SIA5-DT AC096558.1 SLCO1B7 AL035706.1 AC105031.2 GTF2F2 SIM1 ADAMTS19 KIAA1217 ABTB2 SOX30 LINC02227 AC244131.2 LOC338694 TMEM132C ANKS1B SCHLAP1 TRAJ16 EEF1B2P5 SPOCK3 AL669831.1 AC005832.4 GSN SMARCA5 PLS1 AC007991.3 TCP11L1 AC095032.2 SPON1 MAPRE3 MICB-DT ITGB6 ANO2 TLR8 HPS3 SMIM7 AL391361.1 AC011447.2 KIF21A LOC124900600 SLIT3 ENSG00000290427 ARMH3 BAGE2 FMO8P CACUL1 NLRP7 NR1H2 PHC2 MITA1 ROBO1 AC142086.6 ZNF578 AC068726.1 ENSG00000290721 ADAM9 MAD1L1 ENSG00000291002 FAR2P1 ENSG00000287021 IDI1P1 ANKRD11 AL162254.1 ITPR1 FAR2P3 PKP4 AL160286.3 CPM AC105052.4 RN7SL495P DNAAF11 ENSG00000288069 LINC01924 LINC01033 GARNL3 PWRN1 CDC42 NDUFAF7 AC007846.2 ABI3BP RNU1-83P LOC124900504 AXDND1 AC011824.3 TMED5 RNFT2 AC025423.4 IPMK DCAF8L2 LINC03042 AC073575.2 LINC01376 CYP2A13 MTREX RPL23AP51 TMEM183A CASP7 AC126763.1 ENSG00000289956 CAMK1G SLC26A11 AC015819.4 PRUNE2 DOK6 AC105924.1 PSMA2P1 SERPINB9P1 LINC01122 RPL5P5 TTC7A GOLGA8S RNA5SP96 ENSG00000288694 AC073130.2 PRKAG2 LINC02965 ENSG00000290490 AC114316.2 LINC00158 MLLT3 UBE2B CCNT2 LINC01912 BTG4 HAVCR1 DRG1 RSPH10B2 ENSG00000286272 TRAPPC10 MGMT LINC02942 CPQ AC034206.1 ENSG00000288714 POLQ CNOT10 MR1 F8 LOC102724710 KRTAP21-2 ARHGAP23 LOC101927026 OASL ENSG00000289723 AC007326.4 SHMT1 LINC00237 FGF7P5 POU5F1P3 CPT2 MIR325HG AP000320.1 TAS2R14 VPS8 RN7SL759P AC010329.5 MTX3 LINC00703 AC092121.1 ENSG00000286206 RN7SL536P ENSG00000286878 GAPT C4orf50 NXT1-AS1 ITGB3BP AC020743.2 LINC01732 ISY1 ENSG00000287211 CACNA2D3 ZNF831 AC242426.2 CXCR5 VN1R31P NCKAP1 WDR47 PKN2 OR4M2-OT1 PRPF40A CLDN12 LINC02211 KRTAP13-6P LINC00433 ITGAM DTD1 AL137230.2 AC019183.1 CNTN3 CPA1 MGAM PRDM11 GOLGA8M AC016382.1 ENSG00000289085 LINC02427 LINC01060 ELOVL2-AS1 CILK1 LINC01497 HOXC13-AS FOSL2 PACRG IL1RAPL2 IGHVIII-22-2 HNRNPCL3 AC020912.1 CHCHD3 MUC19 AC087477.5 FUT1 ZNF337-AS1 ENSG00000289131 LATS2 LINC02141 TYW3 MYH14 AC008581.2 CC2D2A APCDD1L-DT SPDYE2B TPRG1 MRPL32 ENSG00000286168 KLHL33 ERP29 ARHGAP18 LRRTM4 AC013652.2 UVRAG PTPRN2 SMIM10L2B-AS1 MYOM2 AC113386.1 LYN AC093908.1 USP6 LOC100131779 TCP10L DKKL1P1 MIR4636 LINC00390 MYRF SUMO2 LOC102723883 GRIK1 MTA3 ORC2 CTSLP4 LOC100506869 OTX2 HSD11B1-AS1 LINC02203 UBL3 DIP2B LINC01189 LOC107985126 NME7 LINC02146 SEC24C AC068234.1 AC073869.5 LOC102724849 MIR9-1HG LINC02335 LARGE1 AC109583.3 PRAMEF9 ENSG00000286686 ATP6V1H AC009135.1 NUP58 FAM177B FIGNL1 TGM6 SETP1 AL590399.2 COX18 SLC12A1 LINC01538 AC068547.1 TMCO4 CCDC80 PDZD8 NEMF PAMR1 PRODH CCDC33 MYO3B-AS1 ENSG00000286414 AC003006.1 CA10 DIAPH1 LINC01878 RRM2 U6 CRYBB1 ADGRL3 SIL1 LINC02160 LDB2 MYRFL AC024610.2 UNC93A SPTB DEFB126 AP002075.1 C21orf62 MIR3159 PCM1 LOC84214 ALDH1A1 NMNAT3 FMO5 HELLS RNASEL AC010082.1 LINC02008 NAV1 CMKLR2-AS PAQR5 DBF4B DSCAS ZNF355P CORIN TMCC1 TM2D2 LRRC7 IL6ST PCDHB16 TEX41 LINC00458 AC060765.1 AC027288.1 DCP2 CDH12P1 AC092326.2 RBPMSLP PPM1L TMEM177 ENSG00000286806 PAK5 PDZRN4 FAM151B-DT STX17-DT ENSG00000288055 SLC41A2 NOS3 FER1L6-AS2 AL355306.2 LIPE-AS1 LOC105378402 HMGB3P30 KCNIP4 VSX1 LOC101927745 SEC61GP1 EIF3L ANAPC5 NOMO2 NALCN-AS1 TNRC6C ZNF90P3 PRAMEF27 LINC01208 ARHGAP44 RAD51L3-RFFL MLF2 PGAM1P5 MLLT10 BPHL ERCC6 EIF2AK4 PAUPAR LINC00840 RN7SL738P IGKV2OR2-2 GFAP AC016027.4 NIHCOLE AC027031.1 MSRA AL031599.1 LOC100419786 SYNPR DISP2 RAB12 SIMC1P1 LINC02235 LINC01505 IMPG1 LINC00501 ARHGEF33 HMGCLL1 AL139022.2 LINC01911 DSCR4 XKR6 PKN2-AS1 CAP2 LOC101928335 CSNK1G3 ENSG00000287682 MAGEL2 AF165147.1 MATN2 FAR2P2 TGFBR2 SGO1-AS1 AC141257.2 ITGA11 CMKLR2 CFAP77 WWTR1 DDX60L MARK4 COBL MDN1 AC092131.1 RASAL2 LINC01901 SYNJ1 TTC6 TCF7L2 LOC102724421 LINC02192 ZNF800 AL359232.1 AC114781.2 CHAF1B AC022017.1 ENSG00000290067 NRCAM KIAA1328 ENSG00000286326 HIPK2 ZNF462 ANKIB1 MTRF1 ECHDC2 NUTM2A-AS1 RPGR AC090386.2 OVCH1 CRTAC1 E2F6 PCSK5 EGLN3 ENSG00000291336 CUX1 UNC119 AL133319.1 GPM6B RRAGD MTCO3P12 CLEC4A ULK3 C2orf50 ZNF28 AC083902.1 LOC124904710 LINC02484 LOC339166 SPDYE2 AC124290.1 GPR156 ZMAT4 RGPD8 ENSG00000291239 CNOT10-AS1 ENSG00000289949 AP000529.1 AL445224.1 GOLGA8T MIR497HG TUBBP3 WNT4 ACTBP1 GKAP1 AC120036.1 CACNA2D4 RGPD1 PPP2R5E AL161751.1 AC011632.1 ENSG00000288954 RPL39P33 AC134980.2 KPNB1 INHBA-AS1 ENSG00000290385 FGF7P6 AC010332.2 ENSG00000293191 URAHP TTN-AS1 GSDMD LPAR1 ADGRV1 ST7 SERBP1 AL450423.1 DNM1P33 YTHDC1 AC013401.1 QTRT2 ZKSCAN7 EVA1C AOAH AL161716.1 PHF2P2 RASA3 EPB41L5 GAD1 SCG5 ENSG00000288187 AL035078.2 NUDCD1 AC068254.1 MIR603 LINC00581 AL050327.1 ENSG00000288565 WAC WHRN AC093668.1 OCA2 TDP2 LANCL1-AS1 ULK4P2 ALDH9A1 LOC100419045 RAB6C-AS1 LINC01362 ZNF678 ACSM2B UBBP4 IL7 PRUNE1 TUBB8P6 TTC7B INSYN2A BTG3 APP GATA4 ATF7IP2 SMAD5 IGF2BP1 AC116634.1 C2CD4A PNKD ENSG00000287476 MAMLD1 SLC25A53 OR4F6 RPL9P14 RNLS RNU6-387P AC009093.4 ENSG00000291100 ANKRD20A7P AL163932.1 TFIP11 GRAP2 RTKN2 AL136985.3 ENSG00000289862 ENSG00000291260 NEPRO-AS1 RNF38 DMP1 ADAM12 LOC107986837 HSD17B2 LINC02745 UBE2G1 AC138969.2 NBPF12 ATP9B HMGCS2 TLN1 MIR1273F BTG3-AS1* |

| | | |
|---|---|---|
| | | CACNA2D1 DHX35 ENSG00000287347 SP140L CRACDL ENSG00000286288 SLC4A8 CHRM5 ZNF181 SLC13A1 PI4KB MTMR1 AC129926.2 FRY LINC00670 RHOT1 EPS8 ELOVL5 ARHGAP21 LOC105378976 HERC2 AC010809.1 AL354810.1 HOOK3 USE1 ENSG00000289253 AL513323.1 CAND1 PIK3R5 ENSG00000287231 LYZL1 PALLD ENSG00000290523 GZMAP1 LINC02767 TAS2R30 TMCO3 CASC16 IPO7P2 ENSG00000286132 AL132656.2 RPL36P14 RFESDP1 ZNF529 GSTA11P BMT2 AC233699.1 OR52P2P RIPK4 LINC01876 AVPR1B DMTN LMAN1L ENSG00000286406 AC091073.1 RASGRF1 GOLGA6L10 QDPR KATNIP FRMD5 FRRS1 AC092944.1 ADGRE4P IQCM AP002373.1 PTK2 AC006482.1 ENSG00000293467 AC079414.1 NOP9 IGLV3-2 USP7 LOC101928107 MCU TICAM2 ENSG00000293472 RNA5SP404 SCP2 NPEPPSP1 ALCAM SH2B2 LINC02518 ATP1B4 MPP4 AL031963.1 ZNF41 AC019270.1 AC006153.1 AC006019.3 LINC02250 AC046185.1 GATD3 ENSG00000289444 CELF1 HYCC1 ABRAXAS2 DNAH12 EDNRA POTEG POGZ EFHC1 PTMS PIP4K2B HHLA2 PRRC2B RYR1 SYNM-AS1 SLAMF1 UQCC1 ENSG00000288996 HIGD1AP15 LRRK2 NKAIN3 INO80D AF279873.3 ZNF395 ZNF728 IKZF3 GET1-SH3BGR SLC38A4 SLC25A21 AC006043.1 AC244517.4 FRY-AS1 MIR548XHG AC012038.1 CLSTN2 ARIH1 SAMD12-AS1 AC018731.1 RGPD5 CFAP298 AL359757.2 AC015908.2 VTCN1 ITFG2 PKHD1 ENSG00000287039 MEG8 KIF23-AS1 UBXN7 GDNF-AS1 LINC02435 AC104169.1 ENSG00000290429 PJA2 SNORD114-7 TCF4 AC079742.1 ENSG00000287334 ABCC11 MYH2 AL356585.3 MGST1 RBM44 ARHGEF38 PAEP KLF2P2 ENSG00000290410 COP1 AAMDC TIAM1 CSRP3 PCA3 GOLGA8N GAPDHP70 DDX24 MLIP IQSEC2 EML5 PRIM2 ACVR2A AC243830.3 AC034232.2 AL033530.1 ENSG00000288044 LINC01445 GOLGA8DP TMEM25 AC066613.2 LINC02309 PPL ENSG00000286192 C1QTNF7-AS1 TIPIN VIT ANKRD20A1 ENSG00000293363 LINC01324 AC091078.1 BAALC-AS1 AL606760.2 TMEM212 RASSF4 C6orf58 AC006963.1 LOC124902439 ENSG00000288635 RPL13P7 MIR6882 SNTG2 SNORA5C AC092979.1 POC1A AC060834.2 AC091230.1 NTM AC091133.4 LMCD1-AS1 ATP8B4 MEIS1 AMMECR1 AC004805.1 FP236315.2 LINC00639 CYP1D1P TMED11P ENSG00000289368 BRCC3 CDH12P3 ERCC1 ENSG00000288683 AC093010.2 FAM3B LINC00636 FECHP1 ZNF254 BUB1B-PAK6 DUSP14 LINC02888 ENSG00000290416 COL5A2 PRMT8 LINC01237 SNX13 AC090365.1 AC093893.1 RABGEF1 P2RY8 AC068724.3 CYB5R4 KIT TBC1D8 ENSG00000273937 LINC02294 EPHA7 ENSG00000291067 SPAG9 OSBPL3 AC104574.2 MIR548AG1 ANTXR1 ENSG00000293304 RMEL3 TEX9 RAD54B LOC100652967 AC131254.2 AC069257.3 MCFD2 GRIA1 LINC02256 IL13RA1 C19orf47 AC068299.1 AC007326.2 SPDL1 FAM81B TRABD2B WIPI2 PEX26 AC069277.1 ARFGEF2 TEX2 AL671862.1 CTNNB1 ANKRD17 EFCAB5 REPS2 POTEKP ENSG00000286811 AL096701.3 COL19A1 ZNF468 RUNX1T1 CTSK LOC102723341 PRKDC UQCRB-AS1 ARMH4 ATXN2 AC097626.1 LINC00507 IPO5 AC008517.1 AC026124.1 LOC124900584 H2AC1 TNFRSF19 ZNF479 AC091045.1 DENND2A LUZP2 RPL3P1 TRDN-AS1 PTPN14 LINC02240 SCFD1 NFATC3 ITGB8 KCNMB3 ERC2 POFUT2 LINC02973 DMGDH GOLGA6L5P TRANK1 GXYLT2 BCAR3 TPD52L1 LINC01038 PPM1E ZXDC ALG11 KCNMB3P1 AD000090.1 VPS53 LOC100129404 FRMD4A AC099788.1 CTSLP6 LMX1A MEP1A DCAF8L1 MIR3670-3 WWOX OR4D2 ABCA12 LINC00391 LOC128462377 TPST2 CIMAP2 RPL7P52 CD86 PARS2 MIATNB AC023813.4 AC103740.2 HUNK TRAJ19 AC126335.1 NEK10 LINC00824 LINC01448 SNX29 WWP1 FILIP1 LERFS TMEM62 POLR2M COL8A1 MIR4527HG ANAPC10 FBXO15 EEDP1 PTGER4 AC018467.1 GREB1L SMAP1 ZNF215 AC015574.1 PDE7B ALS2 ECT2 AC096711.2 USP40 ENSG00000286481 RASSF8-AS1 PPP1R15B-AS1 VWC2L BEST3 LINC01681 MTARC2 GSG1L LYPD6B BBOX1-AS1 HERC1 DOCK1 HDDC2 CRADD ENSG00000287180 DIAPH3 AC131902.1 LOC105372316 AC027644.4 CCDC107 OR4X2 TARDBPP2 AC005580.1 AL390195.1 GPC4 ZC4H2 BPGM TTC19 AC034213.1 SLC27A6 REXO1L9P PKIG ENSG00000286614 GAREM1 LINC00276 ANKUB1 RPL21P10 LINC00536 NXN IGF2BP2 AC073316.1 AL035420.1 TSPAN12 AL391869.1 ACTR3B NEK5 AC018638.8 IL17RD ZNF722 CDC14B SYNE3 ENSG00000287684 PLCH1 LINC02808 OR1M1 NAMPT HECTD1 VKORC1L1 LINC01182 ELOA PSG11-AS1 NGEF FGF7P4 TRPC5 AC025278.1 NEUROD2 ENSG00000291261 TTC36 FNIP2 ZIM2-AS1 AC097478.1 KLF2P3 AASS JAML TNRC6A KAT14 LOC613266 LINC00290 UGGT2 CCNJ CR383656.6 STIM2 UBE3B ADORA2A-AS1 TNFSF11 PTPRF ENSG00000288843 LINC01473 AC021351.1 IKBKB AC010280.1 DUX4L51 FXR2 SNHG33 SH3BP5 AC010809.2 MIR3670-4 AC068413.1 LOC101927690 ENSG00000293257 TRBV10-2 AC103409.1 USF3 AC002074.1 LYVE1 AC004870.4 APOH ARHGEF28 OR8B8 ENSG00000290928 CLCN1 STEAP1B TASOR LINC02547 CCPG1 AC104781.2 GPRIN1 LINC00571 EML6 TTC8 POU6F2 GCOM1 OPCML LINGO2 ENSG00000290596 CEMIP XIST AC074286.1 ATF2 LOC101927950 LINC00877 AC140481.3 AC091151.1 RNU6-986P ZNF426 KCNJ12 HDAC8 ZNF589 CHRNB3 ACVR1 HLA-DQA2 PID1 COL6A5 HYAL4 EBF2 CYP3A43 CCDC91 GRID2 ZNF423 XPNPEP3 ZCCHC17 KCNJ3 AC008632.1 AP000487.2 ENSG00000293062 ZNF616 PLA2G7 LINC01926 SNORA63D SLC17A6-DT RB1 RPS29P27 LINC00466 FKBP15 NSMCE1 AC078923.1 MPV17 LOC100131635 IFNLR1 PPIAP56 NTNG1 RNU6-826P AC092447.8 GOLGA6L4 OSBPL9 PDE4B AL365214.3 DDX10 MYHAS LINC02994 RAB11FIP1 PKD1L3 KIF13A NLRP4 ADIPOR1 DPEP1 ENSG00000286655 UGT2B24P SLC23A4P LOC124900205 GABRB3 DSCR9 ZNF106 COL4A1 MYZAP LOC100419851 AC093865.1 SMG7 AC091489.1 PLPP3 ENSG00000290421 AL512444.1 UNC80 HNF1B AL160004.1 RNU6-1311P IMMP2L AP001599.1 CHURC1-FNTB LAMP2 CCDC54 SSPN SPSB1 KIRREL3 AC024075.3 GABRG2 F5 UBE2D4 CFAP95 SCG5-AS1 AK5 AP001207.2 CPS1 TMEM120A BNIP3L AC104984.2 AC023300.1 ENSG00000287614 PRKCH AC021088.1 AGPAT5 FAM66B OR2A14 AC015923.1 FSBP RABGAP1L HSBP1L1 CD47 LINC01948 ENSG00000286476 GRAP LOC101927141 DENND2B LINC00603 XRCC5 TBRG4 LOC107984536 LINC01500 LINC01965 HMGB3P2 AC009055.1 GFRA1 SHLD2 SPHKAP |

| | | |
|---|---|---|
| | | *KCND2 RNU6-705P BMX INPP4A OR5W1P MIR663AHG TRAJ18 SBF2-AS1 ZNF418 LARP4 AC012593.1 SLC24A3 RN7SL563P EGLN3-AS1 RAD54L2 MPRIP LINC02351 TAF15 TPST1 GRIA4 AC023813.3 RNA5SP489 POLD3 RAB18 AP000526.1 TPH2 AL079305.1 POU2F1 AL445648.1 LINC00508 PRPF40B AC004870.3 AC244517.6 ENSG00000286833 SGK3 SPINK8 ENSG00000287534 AL592490.1 LDOC1 NRK ENSG00000287635 ATP6V0E2 MTCH2 RGS7BP MGRN1 LOC339298 LOC101930420 AC010745.2 TEX11 LINC02384 AC091965.1 MID2 ENSG00000286080 AC087683.2 RBM28 MEP1B MIR198 LRTM2 LOC105375972 SIK1 FBXL13 GLOD5 OR10J4 ERCC6L2 ENSG00000291293 SLC17A5 ENSG00000286779 PIPOX TEX15 LINC01644 RPS2P1 AP002336.2 RGPD6 TP53BP2 ZNF850 LINC00592 KAT2B ESPNP PDLIM5 LINC01753 EFCAB11 LSINCT5 GOLPH3L ZNF652 LINC02832 PSMA3-AS1 WLS ACTR3C ANKS1A IGKV1-6 TMPRSS11B PRCP PDE6A AL035446.1 SUPV3L1 RN7SKP86 MEF2D AL512310.1 ZPLD1 MYO1B MGAM2 MYL4 OR2T7 RPL39P31 AC091588.2 PTEN MAST2 LOC107984132 MTX1LP GTF2A1 TC2N SETD2 CA13 REXO1L4P UTP4 SPTLC1 AC015922.2 AL137076.1 AC008758.4 AGR3 AGA-DT CDK5RAP2 FLVCR1 PDC-AS1 ANAPC1P4 AC023830.3 UBE2E3 FSIP2LP SAMD3 RPL15P18 LUCAT1 VEPH1 RFC3 EPN2-AS1 MAB21L3 NAPSB AC093423.2 CROCC FARSB ERICH6B SOX21 CCDC54-AS1 ARHGEF7 MIR1243 ENSG00000286487 LIN54 AL445430.1 ANKRD26P1 LRRC8C SAMD12 DACH1 ENSG00000293462 CU634019.3 RPL21P5 LINC03082 ATF3 TRDN LINC01117 LOC105377043 LINC01934 NPHP1 ENSG00000290597 GASK1A FBN2 ZNF397 AL136146.2 LINC00499 MIS18A AC111152.2 AL592546.1 TEKT4P2 ENSG00000290412 IKZF1 AC005154.5 DAOA-AS1 CHODL-AS1 SLC26A8 SNTG1 CAPZA2 AC004147.4 GGT8P AC011405.1 EEF1A1P2 TRAJ20 AC002066.1 CACHD1 SRI ENSG00000293320 GREB1 AL158154.2 ADAT2 ARNT MARK1 BDNF TFCP2L1 ACBD6 PAK6 PLSCR2 SEMA3E BCLAF3 ENSG00000288768 HAS2-AS1 MBOAT1 SCIN AL163195.3 ATP13A3 ENSG00000293265 AP004609.1 MALRD1 TENT4B MACO1 LOC105376244 AC010601.1 ENSG00000293512 ENSG00000287443 CTBP2P10 NDST4 ADAMTS9 AC004917.1 OR4G3P ADGRF2P STIM1 ITPR2-AS1 TSEN15 TMEM237 TMEM161B ENSG00000288563 RN7SL719P HAUS6 SWT1 RAB11FIP2 PTCD2P1 CDH2 EEFSEC ENSG00000287505 COX7B2 LAG3 DPP10-AS1 AC099753.1 ENSG00000291189 Z95331.1 LOC124902888 TMEM72-AS1 AC016573.1 PAPPA SERPINB7 LOC101927605 IL31RA ZNF407 PPIG SUN1 IRAG1-AS1 ENSG00000290217 ASB3 AC239585.2 MTTP GPR84-AS1 CHRNB4 LOC102724934 CUL4AP1 AC019322.1 AGAP9 CTBP2P9 AL137802.3 AP000282.1 LINC00351 USP9X TGM5 COP1-DT NEK7 AC245128.1 INTS15 RNA5SP472 PTPN20CP ZNF80 OR2T27 PDXDC2P TENM1 IL13RA2 ENSG00000293489 AC138123.1 RORB SLC9A7 AL136441.1 GABRB1 LINC02903 MUC12 MYOF ABCD1P5 AP001116.1 AP005212.4 LINC01754 AC142384.1 LTB4R2 ATG12P1 DLGAP1-AS5 JAKMIP1 LOC101928622 SH3KBP1 NALCN AC116903.2 SMOX AC020718.1 ENSG00000291215 AVIL LPCAT2 ENSG00000286163 ASPA IQCH-AS1 GDAP1 AMY2A DDX60 ATG5 AC023509.6 ENSG00000287083 AC126755.4 MIR922 ENSG00000287801 AC007611.1 ENSG00000289870 TFAP2D ENSG00000289972 ISY1-RAB43 LINC00398 EIF2AP4 TTC39C SPATA22 MIR3670-1 MYLK4 BEND7 DIO2-AS1 Z98043.1 ZC2HC1C USP25 ARMC7 KIF2C AA06 MYO5A SIPA1L3 CTSLP1 NAP1L4 SLC6A13 ZNF286A-TBC1D26 AL731684.2 VSTM2B-DT LINC01667 AC090241.3 LRFN2 WDFY4 PAPPA2 AC068138.1 AC092862.1 SFRP4 BLTP1 SPDYE16 SLC12A9 ENSG00000291036 AL109840.2 MTERF4 CRYBG3 LINC01239 OR4Q2 PNRC1 AC008591.1 ULK4P3 AC015922.3 GOLGA8IP ENSG00000286662 AL772307.1 JCAD ENSG00000286003 NELFB VAV2 COL25A1 DANT1 VPS13A LRRC9 TES OSBPL6 SH3TC1 GRIA3 PNPLA8 SPDYE6 AC087564.1 Z83844.1 AC108474.1 FBN1 NIPAL2 HAPSTR1 GARIN2 SERPINA7P1 AC135983.1 RAB31 MX1-AS1 EXO5-DT ENSG00000291283 DIPK2B ZFP82 MAP3K1 CPPED1 DDX4 IQCB1 COA1 CLEC6A ZNF292 AC010653.2 TBX15 BMPER AC109454.4 FUT10 AC008897.2 AC109779.1 LINC00922 RNU6-984P MYO5BP3 EFHC2 CLTC CLASP2 LINC01931 SMYD2 RPRD2 ZNF761 ENSG00000293497 AC024230.1 DUXAP10 VIPR2 SEMA6A ZNF567-DT PARAIL TMPRSS15 AL133372.2 EEF1A1P11 RN7SL106P FAM186A PLUT LINC00251 B3GAT2 AC091885.2 ATP6V0A4 ACSM5 ENSG00000287516 ZNF618 ITGA4 LIX1-AS1 SEC23A DNAJC6 NSUN2 CXXC4-AS1 ENSG00000290620 ROCK1 SCIRT AL390860.1 NCAPH M1AP PTPRK ZC3H13 AC093515.1 PHACTR4 ACYP1 ENSG00000286072 TAX1BP1 LETM2 AC008739.5 OR7A15P RGPD4 AL445430.2 TBC1D4 OR2M5 AC093843.1 APIP AC012456.1 MMP28 SYNDIG1 FAM13C ASXL3 ZNF701 LINC02971 NECTIN1-DT KCNT2 CRYBB2P1 NUP98 LINC02699 CLEC3A PML BX571673.1 MIR3663HG FOXO3B AC012616.1 AFF2 LAYN MGLL SCFD2 AC011891.2 CYP4X1 TRAPPC12 REXO1L2P EBF4 SCARA5 ENSG00000286740 LONRF3 SMURF1 SLC5A12 AP001021.2 PLEK2 TNFSF4 ENSG00000293021 DGKG LINC00923 SP100 TRIM51JP ENSG00000287616 RNA5SP260 BPTF ENSG00000289040 GRAPL PRKG1 PLXDC2 H2BC1 ELAVL4 RPS27P6 CDH9 RF01880 AL731556.2 FOXRED2 AL359736.1 CFAP298-TCP10L NDUFAF2 UQCC2 LINC01524 AC022335.1 UMODL1 LIFR LNCBRM RFPL4B LINC02360 KCNAB2 AC048382.2 GOLGA8H CLCN5 TSEN2 MAGI2 GALK2 LOC101929174 CYP4F62P ZNF844 ENSG00000287783 LINC02060 NIN SRPK2 SPG21 ZNF286A GARS1-DT AL691482.2 ST7-OT4 ANKFY1 AC008764.1 ZNF69 CCDC144CP PRSS50 ASIC2 MIR4645 ENSA ENSG00000286259 LOC124900778 LOC100506274 PPP4R1L MIR9-2HG FNDC3A MOB1AP2 KHDRBS3 SLC30A10 AIMP1 AP4S1 FOXG1-AS1 MYO5BP1 TPK1 E2F6P2 AC092902.5 TNFAIP8 ENSG00000287998 ANKRD50 CALCRL CASC17 USP34 AC091053.1 STK4 AC138305.1 AC023300.3 ENSG00000289174 TNNI3K AL049833.3 AL646090.2 ENSG00000287184 DNAAF4-CCPG1 TNN RAB6D TMEM131 SPC25 AC112493.1 ENSG00000287378 OXR1-AS1 ST6GAL2 RNASE11 SOX21-AS1 AC018742.1 AC244205.1 SCAMP1 TXNL4AP1 PTCHD4 BANP RPL27A ANPEP AC021979.1 LINC01821 PRLR ZNF569 GOLGA8O LINC01697 PTPRA PADI2 AC011287.1 ARL2BPP10 LNX2 ENSG00000289397 AP005436.1 ZNF180 AC007656.1 AC008758.3* |

| | | |
|---|---|---|
| | | *SYCP2 NMI RAB6C PCCA AF241726.2 TMEM163 CLVS1 YLPM1 EYS IL16 LINC00972 ENSG00000291276 AC012485.1 DIPK1A LINC02814 SULT1C2P2 ZSWIM7 RAVER2 ZNF470 POU2F3 ENSG00000286932 AC107373.1 LINC02339 POTEH EDARADD LINC00558 LINC02511 AC120036.4 CR2 OXNAD1 GNA14 CSN1S1 CR392039.3 ENSG00000286152 LPAR6 ZNF56P CTBP2 RGS6 LINC01721 PCNX4-DT ZNF525 AC093895.1 NCALD RPS10P13 HHIP LINC00367 GXYLT1 DYRK1A LOC105377209 PPFIA1 TMEM30A LINC01705 LDHBP3 MYRIP ERICH2-DT LINC00343 PPP1R1C ZNF286B AL355838.1 KCNE2 UNC13C CUL4A NDUFAF4P1 RIMS2 TMEM258 PLD3 AC105052.3 ENSG00000286085 SLC5A1 AC015687.1 AL670729.1 LINC02241 SERINC1 LINC00265 KCTD16 LOC105370802 CDCP1 CHSY3 TXNRD3 AC026358.1 TRAM2-AS1 AC023590.1 EYA4 ZNF888 AL391361.2 SIK3 LINC01748 AC069287.3 DLGAP2 AL163953.1 ARL15 IGHV3OR16-12 CHRNA9 SPATA13 LINC01288 TOPAZ1 ZNF736 AL031432.1 MAP7D3 SEMA5A LRRC28 ENSG00000288035 MORN2 5S_rRNA AL354861.2 LINC02762 PDZD4 NAA25 ENSG00000290805 EIF3FP1 ELL2 LINC00886 FKBP9 LAMA4 LINC01811 UBE3C ATE1 PLCH1-AS1 CCDC92 ENSG00000293330 AC110772.1 ETNK1 TEX30 TTPA MYO5BP2 DUX4L33 PEX5L CYP4F30P SLC2A1-DT PVT1 GCNT1P3 ARL17A GATAD2B METTL3 KRT85 RANBP2 AC019117.2 AC068112.1 ZKSCAN7-AS1 UAP1 PARD3 CCDC73 FAM110B IGLVV-66 LINC00691 KCNN4 LINC01104 TRDC CLDN18 ZNF33A RNF128 AL353132.1 AL049869.2 SLC25A48 CP ENSG00000293385 FUNDC1 RNU6-849P COL6A6 BMAL2 LRRC3B PGPEP1 SATB1-AS1 AC026462.4 LINC02885 BZW1-AS1 NDRG3 AC019226.2 RNF6 SEMA3C VAV1 SMYD1 FANCB RUBCN PEPD ENSG00000289871 DPY19L1 GRIA2 GRM3-AS1 VDAC2 KIAA0825 AC002127.2 C12orf75-AS1 MIR3670-2 ENSG00000276197 GALNTL5 HCCS-DT AL731661.2 SPART AC018697.1 MIR1304 AL138759.1 LOC102723684 IGLV4-3 LINC02315 RN7SL545P* |

**Table S5.** GO associations with biological processes (BP), molecular functions (MF), and cellular components (CC) of 188 rDNA-contacting genes detected in Mel Z cells grown on plastic. Related to Figure 1D.

| GO.ID | Description | padj | Genes |
|---|---|---|---|
| MF | | | |
| GO:0140110 | transcription regulator activity | 0.0006184125350523696 | *MED13L,NFIA,THRB,ZNF536,ARID1B,TBL1XR1,AFF3,ZMYND8,KLF12,ZNF429,KLF7,ZNF280B,ZNF607,ZFHX3,RARB,ESR1,BBX,RERE,NR3C2,ZSCAN23,ZNF615,NCOA2,ZNF860,ETV1,ZNF197,ZNF665,PPARG,ZNF234,TRERF1,ZNF521,IKZF2,NFIB,BCL11A,NPAS3* |
| GO:0043167 | ion binding | 0.0015377009170145755 | *CDK14,PDE1C,MAOA,THRB,MAPK10,ZNF536,ACSS3,CACNA1C,FBLN2,MBTD1,NUDT13,LPP,MPPED2,PTK2B,ATP4A,PRKG2,HMCN1,ZMYND8,RSKR,UBR5,ARHGEF39,PAK3,KLF12,GDPD1,PRICKLE2,ZNF429,EPHA3,KLF7,KIF18A,ZNF280B,KMT2A,ZNF607,ZFHX3,RASGRF2,DAPK1,HCN1,RARB,GNAO1,ASAP2,AGAP4,ESR1,L3MBTL4,FIGN,RERE,NR3C2,RAB3GAP2,ZSCAN23,MX1,ZNF615,ACACB,ZNF860,ZCWPW2,EYA2,ZNF197,TRPV6,ZNF665,CAMK2D,KIF16B,PPARG,CDK6,STK32A,ZNF234,TRERF1,RAPGEF4,ZNF521,PHLPP1,IKZF2,SBF2,PRKD1,PDE11A,TTN,BCL11A,ATP8B1* |
| GO:0036094 | small molecule binding | 0.0024529513965331996 | *CDK14,PDE1C,MAOA,THRB,MAPK10,ZNF536,ACSS3,CACNA1C,FBLN2,MBTD1,NUDT13,LPP,MPPED2,PTK2B,ATP4A,PRKG2,HMCN1,ZMYND8,RSKR,ALDH1A2,UBR5,ARHGEF39,PAK3,KLF12,GDPD1,PRICKLE2,ZNF429,EPHA3,KLF7,KIF18A,ZNF280B,KMT2A,ZNF607,ZFHX3,RASGRF2,DAPK1,HCN1,RARB,GNAO1,ASAP2,AGAP4,ESR1,L3MBTL4,FIGN,RERE,NR3C2,RAB3GAP2,ZSCAN23,MX1,ZNF615,ACACB,ZNF860,ZCWPW2,EYA2,ZNF197,TRPV6,ZNF665,CAMK2D,KIF16B,PPARG,CDK6,STK32A,ZNF234,TRERF1,RAPGEF4,ZNF521,PHLPP1,IKZF2,SBF2,PRKD1,PDE11A,TTN,BCL11A,ATP8B1* |
| GO:0000978 | RNA polymerase II cis-regulatory region sequence-specific DNA binding | 0.0026186895179163747 | *NFIA,THRB,ZNF536,KLF12,ZNF429,KLF7,ZNF280B,ZNF607,ZFHX3,RARB,ESR1,NR3C2,ZSCAN23,ZNF615,NCOA2,ZNF860,ETV1,ZNF197,ZNF665,PPARG,ZNF521,IKZF2,NFIB,BCL11A* |
| GO:00037 | DNA-binding | 0.0029127 | *NFIA,THRB,ZNF536,AFF3,KLF12,ZNF429,KLF7,ZNF280B,ZNF6* |

| | | | |
|---|---|---|---|
| 00 | transcription factor activity | 54726228716 | *07,ZFHX3,RARB,ESR1,BBX,NR3C2,ZSCAN23,ZNF615,ZNF860,ETV1,ZNF197,ZNF665,PPARG,ZNF234,ZNF521,IKZF2,NFIB,BCL11A,NPAS3* |
| GO:0000987 | cis-regulatory region sequence-specific DNA binding | 0.0036332133449603573 | *NFIA,THRB,ZNF536,KLF12,ZNF429,KLF7,ZNF280B,ZNF607,ZFHX3,RARB,ESR1,NR3C2,ZSCAN23,ZNF615,NCOA2,ZNF860,ETV1,ZNF197,ZNF665,PPARG,ZNF521,IKZF2,NFIB,BCL11A* |
| GO:0000977 | RNA polymerase II transcription regulatory region sequence-specific DNA binding | 0.004107430457269752 | *NFIA,THRB,ZNF536,KLF12,ZNF429,KLF7,ZNF280B,ZNF607,ZFHX3,RARB,ESR1,BBX,NR3C2,ZSCAN23,ZNF615,NCOA2,ZNF860,ETV1,ZNF197,ZNF665,PPARG,ZNF521,IKZF2,NFIB,BCL11A,NPAS3* |
| GO:0043565 | sequence-specific DNA binding | 0.004176167758919623 | *NFIA,THRB,ZNF536,TBL1XR1,KLF12,ZNF429,KLF7,ZNF280B,KMT2A,ZNF607,ZFHX3,RARB,ESR1,BBX,RERE,NR3C2,ZSCAN23,ZNF615,NCOA2,ZNF860,ETV1,ZNF197,ZNF665,PPARG,ZNF521,IKZF2,NFIB,BCL11A,NPAS3* |
| GO:0000976 | transcription cis-regulatory region binding | 0.004636845526895301 | *NFIA,THRB,ZNF536,TBL1XR1,KLF12,ZNF429,KLF7,ZNF280B,ZNF607,ZFHX3,RARB,ESR1,BBX,NR3C2,ZSCAN23,ZNF615,NCOA2,ZNF860,ETV1,ZNF197,ZNF665,PPARG,ZNF521,IKZF2,NFIB,BCL11A,NPAS3* |
| GO:0001067 | transcription regulatory region nucleic acid binding | 0.004694330738262828 | *NFIA,THRB,ZNF536,TBL1XR1,KLF12,ZNF429,KLF7,ZNF280B,ZNF607,ZFHX3,RARB,ESR1,BBX,NR3C2,ZSCAN23,ZNF615,NCOA2,ZNF860,ETV1,ZNF197,ZNF665,PPARG,ZNF521,IKZF2,NFIB,BCL11A,NPAS3* |
| GO:1990837 | sequence-specific double-stranded DNA binding | 0.009724616382750486 | *NFIA,THRB,ZNF536,TBL1XR1,KLF12,ZNF429,KLF7,ZNF280B,ZNF607,ZFHX3,RARB,ESR1,BBX,NR3C2,ZSCAN23,ZNF615,NCOA2,ZNF860,ETV1,ZNF197,ZNF665,PPARG,ZNF521,IKZF2,NFIB,BCL11A,NPAS3* |
| GO:0003690 | double-stranded DNA binding | 0.010296766804114654 | *NFIA,THRB,ZNF536,TBL1XR1,AFF3,KLF12,ZNF429,KLF7,ZNF280B,ZNF607,ZFHX3,RARB,ESR1,BBX,NR3C2,ZSCAN23,ZNF615,NCOA2,ZNF860,ETV1,ZNF197,ZNF665,PPARG,ZNF521,IKZF2,NFIB,BCL11A,NPAS3* |
| GO:0005516 | calmodulin binding | 0.013532634848994116 | *PDE1C,CACNA1C,KCNQ3,RASGRF2,DAPK1,ESR1,TRPV6,CAMK2D,TTN* |
| GO:0000981 | DNA-binding transcription factor activity, RNA polymerase II-specific | 0.026209255352545986 | *NFIA,THRB,ZNF536,KLF12,ZNF429,KLF7,ZNF280B,ZNF607,ZFHX3,RARB,ESR1,BBX,NR3C2,ZSCAN23,ZNF615,ZNF860,ETV1,ZNF197,ZNF665,PPARG,ZNF521,NFIB,BCL11A,NPAS3* |
| GO:0106310 | protein serine kinase activity | 0.04089239371178638 | *CDK14,MAPK10,PRKG2,RSKR,PAK3,DAPK1,CAMK2D,CDK6,STK32A,PRKD1,TTN* |
| GO:0004879 | nuclear receptor activity | 0.04383662891326459 | *THRB,RARB,ESR1,NR3C2,PPARG* |

| | | | |
|---|---|---|---|
| GO:0098531 | ligand-modulated transcription factor activity | 0.04754612849417191 | *THRB,RARB,ESR1,NR3C2,PPARG* |
| | | *BP* | |
| GO:0048699 | generation of neurons | 0.000040160883766705496 | *APBB2,NFIA,THRB,GRIP1,ZNF536,KCNQ3,PTK2B,ZMYND8,ALDH1A2,CHL1,PAK3,EPHA3,ADGRB3,KLF7,ZFHX3,HCN1,CECR2,NEDD4L,WNT5A,RERE,EFNA5,TSHR,ETV1,CDK6,ZNF521,LAMB1,NFIB,PRKD1,BCL11A,ATP8B1,VPS13B* |
| GO:0030182 | neuron differentiation | 0.0000414510704757256 | *APBB2,NFIA,THRB,GRIP1,ZNF536,KCNQ3,PTK2B,ZMYND8,ALDH1A2,CHL1,PAK3,EPHA3,ADGRB3,KLF7,ZFHX3,HCN1,CECR2,NEDD4L,WNT5A,RERE,EFNA5,TSHR,ETV1,ZNF521,LAMB1,NFIB,PRKD1,BCL11A,ATP8B1,VPS13B* |
| GO:0022008 | neurogenesis | 0.00008734834287884851 | *APBB2,NFIA,THRB,GRIP1,ZNF536,KCNQ3,PTK2B,ZMYND8,ALDH1A2,CHL1,PAK3,EPHA3,ADGRB3,KLF7,ZFHX3,HCN1,CECR2,RARB,NEDD4L,WNT5A,RERE,EFNA5,TSHR,ETV1,PPARG,CDK6,ZNF521,LAMB1,NFIB,PRKD1,BCL11A,ATP8B1,VPS13B* |
| GO:0065007 | biological regulation | 0.00010944746200296945 | *APBB2,CDK14,PDE1C,MED13L,NFIA,MAOA,THRB,MAPK10,CACNB1,SORBS2,GRIP1,DLGAP1,ZNF536,ARID1B,CACNA1C,TBL1XR1,FBLN2,MBTD1,TBC1D32,KCNQ3,AFF3,PTK2B,CTIF,ATP4A,PRKG2,ZMYND8,ALDH1A2,UBR5,CHL1,ARHGEF39,PAK3,KLF12,PRICKLE2,ZNF429,ADGRD1,EPHA3,ADGRB3,KLF7,SHROOM3,KIF18A,ZNF280B,KMT2A,KIAA1614,ZNF607,ZFHX3,RASGRF2,DAPK1,PELI2,HCN1,TLR1,PLD1,RARB,MEGF10,GNAO1,ASAP2,GABBR1,NEDD4L,WNT5A,ESR1,SLC7A11,L3MBTL4,BNIPL,BBX,RERE,NR3C2,EFNA5,DMXL2,AKAP6,RAB3GAP2,TENT5B,SHROOM4,ZSCAN23,MX1,ZNF615,NCOA2,ACACB,DLC1,RGS17,CLDN1,ZNF860,TSHR,ETV1,EYA2,ZNF197,TRPV6,LDLRAD4,ZNF665,TLR6,CAMK2D,KIF16B,PPARG,TANC1,CDK6,ST8SIA1,STK32A,ZNF234,IL1RAP,TRERF1,RAPGEF4,ZNF521,ANGPT1,PHLPP1,IKZF2,AFAP1L2,LAMB1,NFIB,TTC28,PRKD1,PDE11A,TTN,SRGAP3,BCL11A,FMNL2,NPAS3,ATP8B1* |
| GO:0007275 | multicellular organism development | 0.0001341199229246485 | *APBB2,NFIA,THRB,SORBS2,GRIP1,ZNF536,ARID1B,CACNA1C,TBL1XR1,MBTD1,TBC1D32,KCNQ3,COL12A1,AFF3,PTK2B,PRKG2,ZMYND8,ALDH1A2,CHL1,PAK3,EPHA3,ADGRB3,KLF7,SHROOM3,KIF18A,KMT2A,ZFHX3,HCN1,CECR2,RARB,NEDD4L,WNT5A,ESR1,SLC7A11,BBX,RERE,EFNA5,AKAP6,SHROOM4,ACACB,DLC1,CLDN1,TSHR,ETV1,EYA2,KIF16B,PPARG,CDK6,IL1RAP,ZNF521,ANGPT1,PHLPP1,LAMB1,SBF2,NFIB,PRKD1,TTN,BCL11A,ATP8B1,VPS13B* |
| GO:0007399 | nervous system development | 0.00015648792224342493 | *APBB2,NFIA,THRB,GRIP1,ZNF536,ARID1B,TBC1D32,KCNQ3,PTK2B,ZMYND8,ALDH1A2,CHL1,PAK3,EPHA3,ADGRB3,KLF7,ZFHX3,HCN1,CECR2,RARB,NEDD4L,WNT5A,SLC7A11,RERE,EFNA5,SHROOM4,DLC1,CLDN1,TSHR,ETV1,PPARG,CDK6,IL1RAP,ZNF521,LAMB1,SBF2,NFIB,PRKD1,BCL11A,ATP8B1,VPS13B* |
| GO:0048731 | system development | 0.0001850474234014489 | *APBB2,NFIA,THRB,SORBS2,GRIP1,ZNF536,ARID1B,CACNA1C,MBTD1,TBC1D32,KCNQ3,PTK2B,PRKG2,ZMYND8,ALDH1A2,CHL1,PAK3,EPHA3,ADGRB3,KLF7,KIF18A,KMT2A,ZFHX3,HCN1,CECR2,RARB,NEDD4L,WNT5A,ESR1,SLC7A11,BBX,RERE,EFNA5,AKAP6,SHROOM4,ACACB,DLC1,CLDN1,TSHR,ETV1,PPARG,CDK6,IL1RAP,ZNF521,ANGPT1,PHLPP1,LAMB1,SBF2,NFIB,PRKD1,TTN,BCL11A,ATP8B1,VPS13B* |
| GO:0050789 | regulation of biological process | 0.00022225206417157003 | *APBB2,CDK14,PDE1C,MED13L,NFIA,MAOA,THRB,MAPK10,CACNB1,SORBS2,GRIP1,DLGAP1,ZNF536,ARID1B,CACNA1C,TBL1XR1,FBLN2,MBTD1,TBC1D32,KCNQ3,AFF3,PTK2B,CTIF,ATP4A,PRKG2,ZMYND8,ALDH1A2,UBR5,CHL1,ARHGEF39,PAK3,KLF12,PRICKLE2,ZNF429,ADGRD1,EPHA3,ADGRB3,KLF7,SHROOM3,KIF18A,ZNF280B,KMT2A,KIAA1614,ZNF607,ZFHX3,RASGRF2,DAPK1,PELI2,HCN1,TLR1,PLD1,RARB,MEGF10,GNAO1,GABBR1,NEDD4L,WNT5A,ESR1,SLC7A11,L3MBTL4,BNIPL,BBX,RERE,NR3C2,EFNA5,AKAP6,RAB3GAP2,TENT5B,ZSCAN23,MX1,ZNF615,NCOA2,ACACB,DLC1,RGS17,CLDN1,ZNF860,TSHR,ETV1,EYA2,ZNF197,TRPV6,LDLRAD4,ZNF665,TLR6,CAMK2D,KIF16B,PPARG,TANC1,CDK6,ST8SIA1,STK32A,ZNF234,IL1RAP,TRERF1,RAPGEF4,ZNF521,ANGPT1,PHLPP1,IKZF2,AFAP1L2,LAMB1,NFIB,TTC28,PRKD1,PDE11A,TTN,SRGAP3,BCL11A,FMNL2,NPAS3,ATP8B1* |

| | | | |
|---|---|---|---|
| GO:0048666 | neuron development | 0.000449433115996208 | *APBB2,THRB,GRIP1,KCNQ3,PTK2B,ZMYND8,CHL1,PAK3,EPHA3,ADGRB3,KLF7,HCN1,CECR2,NEDD4L,WNT5A,RERE,EFNA5,TSHR,ETV1,LAMB1,NFIB,PRKD1,BCL11A,ATP8B1,VPS13B* |
| GO:0050794 | regulation of cellular process | 0.00045256869149219085 | *APBB2,CDK14,PDE1C,MED13L,NFIA,MAOA,THRB,MAPK10,CACNB1,SORBS2,GRIP1,ZNF536,ARID1B,CACNA1C,TBL1XR1,FBLN2,MBTD1,TBC1D32,KCNQ3,AFF3,PTK2B,CTIF,ATP4A,PRKG2,ZMYND8,ALDH1A2,UBR5,CHL1,ARHGEF39,PAK3,KLF12,PRICKLE2,ZNF429,ADGRD1,EPHA3,ADGRB3,KLF7,KIF18A,ZNF280B,KMT2A,KIAA1614,ZNF607,ZFHX3,RASGRF2,DAPK1,PELI2,HCN1,TLR1,PLD1,RARB,MEGF10,GNAO1,GABBR1,NEDD4L,WNT5A,ESR1,SLC7A11,L3MBTL4,BNIPL,BBX,RERE,NR3C2,EFNA5,AKAP6,RAB3GAP2,TENT5B,ZSCAN23,MX1,ZNF615,NCOA2,ACACB,DLC1,RGS17,CLDN1,ZNF860,TSHR,ETV1,EYA2,ZNF197,TRPV6,LDLRAD4,ZNF665,TLR6,CAMK2D,KIF16B,PPARG,TANC1,CDK6,ST8SIA1,STK32A,ZNF234,IL1RAP,TRERF1,RAPGEF4,ZNF521,ANGPT1,PHLPP1,IKZF2,AFAP1L2,LAMB1,NFIB,TTC28,PRKD1,PDE11A,TTN,SRGAP3,BCL11A,NPAS3,ATP8B1* |
| GO:0141193 | nuclear receptor-mediated signaling pathway | 0.0005510212557834107 | *THRB,ZNF536,ALDH1A2,UBR5,RARB,ESR1,NR3C2,NCOA2,PPARG,TRERF1* |
| GO:0009891 | positive regulation of biosynthetic process | 0.0011500879467210114 | *APBB2,NFIA,THRB,ARID1B,TBL1XR1,MBTD1,PTK2B,CTIF,ZMYND8,ALDH1A2,UBR5,KLF12,KLF7,KMT2A,ZFHX3,TLR1,PLD1,RARB,WNT5A,ESR1,RERE,RAB3GAP2,TENT5B,ZNF615,NCOA2,ETV1,ZNF197,TLR6,PPARG,CDK6,IL1RAP,TRERF1,ZNF521,ANGPT1,AFAP1L2,NFIB,PRKD1,TTN,BCL11A,NPAS3* |
| GO:0010557 | positive regulation of macromolecule biosynthetic process | 0.0011817250251716948 | *APBB2,NFIA,THRB,ARID1B,TBL1XR1,MBTD1,PTK2B,CTIF,ZMYND8,ALDH1A2,UBR5,KLF12,KLF7,KMT2A,ZFHX3,TLR1,PLD1,RARB,WNT5A,ESR1,RERE,TENT5B,ZNF615,NCOA2,ETV1,ZNF197,TLR6,PPARG,CDK6,IL1RAP,TRERF1,ZNF521,ANGPT1,AFAP1L2,NFIB,PRKD1,TTN,BCL11A,NPAS3* |
| GO:0009653 | anatomical structure morphogenesis | 0.0013539601354727304 | *APBB2,NFIA,THRB,GRIP1,CACNA1C,TBC1D32,COL12A1,AFF3,PTK2B,ALDH1A2,CHL1,PAK3,EPHA3,ADGRB3,KLF7,SHROOM3,HCN1,CECR2,RARB,NEDD4L,WNT5A,ESR1,RERE,EFNA5,DLC1,TSHR,ETV1,EYA2,KIF16B,PPARG,TANC1,ANGPT1,LAMB1,NFIB,PRKD1,TTN,BCL11A,FMNL2,ATP8B1,VPS13B* |
| GO:0010604 | positive regulation of macromolecule metabolic process | 0.004475475551914102 | *APBB2,NFIA,THRB,ARID1B,TBL1XR1,MBTD1,PTK2B,CTIF,ZMYND8,ALDH1A2,UBR5,KLF12,KLF7,KMT2A,ZFHX3,PELI2,TLR1,PLD1,RARB,NEDD4L,WNT5A,ESR1,RERE,EFNA5,RAB3GAP2,TENT5B,ZNF615,NCOA2,ETV1,EYA2,ZNF197,TLR6,PPARG,CDK6,IL1RAP,TRERF1,ZNF521,ANGPT1,AFAP1L2,NFIB,PRKD1,TTN,BCL11A,NPAS3* |
| GO:0009755 | hormone-mediated signaling pathway | 0.004890193681173468 | *THRB,ZNF536,ALDH1A2,UBR5,RARB,ESR1,NR3C2,TSHR,PPARG,TRERF1* |
| GO:0048856 | anatomical structure development | 0.004901637230433587 | *APBB2,NFIA,THRB,SORBS2,GRIP1,ZNF536,ARID1B,CACNA1C,TBL1XR1,MBTD1,TBC1D32,KCNQ3,COL12A1,AFF3,PTK2B,PRKG2,ZMYND8,ALDH1A2,CHL1,PAK3,EPHA3,ADGRB3,KLF7,SHROOM3,KIF18A,KMT2A,ZFHX3,HCN1,CECR2,RARB,MEGF10,NEDD4L,CFAP44,WNT5A,ESR1,SLC7A11,BBX,RERE,EFNA5,AKAP6,SHROOM4,NCOA2,ACACB,DLC1,CLDN1,TSHR,ETV1,EYA2,LDLRAD4,KIF16B,PPARG,TANC1,CDK6,IL1RAP,ZNF521,ANGPT1,PHLPP1,LAMB1,SBF2,NFIB,PRKD1,TTN,BCL11A,FMNL2,ATP8B1,VPS13B* |
| GO:0048518 | positive regulation of biological process | 0.004990272644995997 | *APBB2,NFIA,MAOA,THRB,CACNB1,GRIP1,ARID1B,CACNA1C,TBL1XR1,FBLN2,MBTD1,PTK2B,CTIF,PRKG2,ZMYND8,ALDH1A2,UBR5,ARHGEF39,PAK3,KLF12,EPHA3,ADGRB3,KLF7,KMT2A,ZFHX3,RASGRF2,DAPK1,PELI2,TLR1,PLD1,RARB,MEGF10,GABBR1,NEDD4L,WNT5A,ESR1,RERE,NR3C2,EFNA5,AKAP6,RAB3GAP2,TENT5B,ZNF615,NCOA2,ACACB,DLC1,CLDN1,TSHR,E* |

| | | | |
|---|---|---|---|
| | | | *TV1,EYA2,ZNF197,TLR6,CAMK2D,PPARG,CDK6,ST8SIA1,IL1RAP,TRERF1,RAPGEF4,ZNF521,ANGPT1,AFAP1L2,LAMB1,NFIB,PRKD1,TTN,BCL11A,NPAS3* |
| GO:0048384 | retinoic acid receptor signaling pathway | 0.005811785713708113 | *THRB,ZNF536,ALDH1A2,RARB,PPARG* |
| GO:1901701 | cellular response to oxygen-containing compound | 0.006353034960970903 | *CACNB1,ARID1B,PTK2B,ALDH1A2,EPHA3,KLF7,KIF18A,DAPK1,HCN1,RARB,GNAO1,WNT5A,ESR1,EFNA5,AKAP6,NCOA2,CLDN1,TSHR,TLR6,PPARG,TRERF1,PRKD1,BCL11A* |
| GO:0048522 | positive regulation of cellular process | 0.006859598878757246 | *APBB2,NFIA,MAOA,THRB,GRIP1,ARID1B,TBL1XR1,FBLN2,MBTD1,PTK2B,CTIF,PRKG2,ZMYND8,ALDH1A2,UBR5,ARHGEF39,KLF12,EPHA3,ADGRB3,KLF7,KMT2A,ZFHX3,RASGRF2,DAPK1,PELI2,TLR1,PLD1,RARB,MEGF10,GABBR1,NEDD4L,WNT5A,ESR1,RERE,NR3C2,EFNA5,AKAP6,RAB3GAP2,TENT5B,ZNF615,NCOA2,ACACB,DLC1,CLDN1,TSHR,ETV1,EYA2,ZNF197,TLR6,CAMK2D,PPARG,CDK6,ST8SIA1,IL1RAP,TRERF1,RAPGEF4,ZNF521,ANGPT1,AFAP1L2,LAMB1,NFIB,PRKD1,TTN,BCL11A,NPAS3* |
| GO:0006357 | regulation of transcription by RNA polymerase II | 0.0076412197940895115 | *APBB2,MED13L,NFIA,THRB,ZNF536,ARID1B,TBL1XR1,ZMYND8,KLF12,ZNF429,KLF7,ZNF280B,KMT2A,ZNF607,ZFHX3,RARB,WNT5A,ESR1,BBX,NR3C2,ZSCAN23,ZNF615,NCOA2,ZNF860,ETV1,ZNF197,ZNF665,CAMK2D,PPARG,CDK6,TRERF1,ZNF521,IKZF2,NFIB,PRKD1,BCL11A,NPAS3* |
| GO:0031175 | neuron projection development | 0.007804701747525311 | *APBB2,GRIP1,PTK2B,ZMYND8,CHL1,PAK3,EPHA3,ADGRB3,KLF7,CECR2,NEDD4L,WNT5A,RERE,EFNA5,TSHR,ETV1,LAMB1,NFIB,PRKD1,BCL11A,VPS13B* |
| GO:0009893 | positive regulation of metabolic process | 0.0084001621862824 7 | *APBB2,NFIA,THRB,ARID1B,TBL1XR1,MBTD1,PTK2B,CTIF,ZMYND8,ALDH1A2,UBR5,KLF12,KLF7,KMT2A,ZFHX3,DAPK1,PELI2,TLR1,PLD1,RARB,NEDD4L,WNT5A,ESR1,RERE,EFNA5,RAB3GAP2,TENT5B,ZNF615,NCOA2,TSHR,ETV1,EYA2,ZNF197,TLR6,PPARG,CDK6,IL1RAP,TRERF1,ZNF521,ANGPT1,AFAP1L2,NFIB,PRKD1,TTN,BCL11A,NPAS3* |
| GO:0007154 | cell communication | 0.011655659498694779 | *APBB2,CDK14,PDE1C,NFIA,MAOA,THRB,MAPK10,CACNB1,SORBS2,GRIP1,DLGAP1,ZNF536,CACNA1C,TBL1XR1,TBC1D32,KCNQ3,PTK2B,PRKG2,ZMYND8,ALDH1A2,UBR5,CHL1,PAK3,PRICKLE2,ADGRD1,EPHA3,ADGRB3,KLF7,KMT2A,RASGRF2,DAPK1,PELI2,HCN1,TLR1,PLD1,RARB,GNAO1,GABBR1,WNT5A,ESR1,SLC7A11,NR3C2,EFNA5,AKAP6,MX1,NCOA2,DLC1,RGS17,TSHR,EYA2,TRPV6,LDLRAD4,TLR6,CAMK2D,KIF16B,PPARG,CDK6,STK32A,IL1RAP,TRERF1,RAPGEF4,ANGPT1,PHLPP1,AFAP1L2,LAMB1,PRKD1,PDE11A,TTN,SRGAP3* |
| GO:0006355 | regulation of DNA-templated transcription | 0.011717169275009875 | *APBB2,MED13L,NFIA,THRB,ZNF536,ARID1B,TBL1XR1,MBTD1,AFF3,ZMYND8,KLF12,ZNF429,KLF7,ZNF280B,KMT2A,ZNF607,ZFHX3,RARB,WNT5A,ESR1,L3MBTL4,BBX,RERE,NR3C2,ZSCAN23,ZNF615,NCOA2,ZNF860,ETV1,ZNF197,ZNF665,CAMK2D,PPARG,CDK6,ZNF234,TRERF1,ZNF521,IKZF2,AFAP1L2,NFIB,PRKD1,BCL11A,NPAS3,ATP8B1* |
| GO:2001141 | regulation of RNA biosynthetic process | 0.0135536611050203 5 | *APBB2,MED13L,NFIA,THRB,ZNF536,ARID1B,TBL1XR1,MBTD1,AFF3,ZMYND8,KLF12,ZNF429,KLF7,ZNF280B,KMT2A,ZNF607,ZFHX3,RARB,WNT5A,ESR1,L3MBTL4,BBX,RERE,NR3C2,ZSCAN23,ZNF615,NCOA2,ZNF860,ETV1,ZNF197,ZNF665,CAMK2D,PPARG,CDK6,ZNF234,TRERF1,ZNF521,IKZF2,AFAP1L2,NFIB,PRKD1,BCL11A,NPAS3,ATP8B1* |
| GO:0048468 | cell development | 0.015205785308266514 | *APBB2,THRB,SORBS2,GRIP1,ARID1B,KCNQ3,PTK2B,ZMYND8,ALDH1A2,CHL1,PAK3,EPHA3,ADGRB3,KLF7,KMT2A,HCN1,CECR2,RARB,MEGF10,NEDD4L,CFAP44,WNT5A,ESR1,RERE,EFNA5,AKAP6,CLDN1,TSHR,ETV1,PPARG,CDK6,ANGPT1,LAMB1,NFIB,PRKD1,TTN,BCL11A,ATP8B1,VPS13B* |
| GO:0030522 | intracellular receptor signaling | 0.015445172579293457 | *THRB,ZNF536,ALDH1A2,UBR5,RARB,ESR1,NR3C2,NCOA2,TLR6,PPARG,TRERF1,PRKD1* |

| | | | |
|---|---|---|---|
| | pathway | | |
| GO:0030154 | cell differentiation | 0.019964522686734546 | *APBB2,NFIA,THRB,SORBS2,GRIP1,ZNF536,ARID1B,TBL1XR1,KCNQ3,COL12A1,PTK2B,PRKG2,ZMYND8,ALDH1A2,CHL1,PAK3,EPHA3,ADGRB3,KLF7,KMT2A,ZFHX3,HCN1,CECR2,RARB,MEGF10,GABBR1,NEDD4L,CFAP44,WNT5A,ESR1,SLC7A11,RERE,EFNA5,AKAP6,CLDN1,TSHR,ETV1,EYA2,LDLRAD4,PPARG,TANC1,CDK6,ZNF521,ANGPT1,SPATA25,LAMB1,NFIB,PRKD1,TTN,BCL11A,ATP8B1,VPS13B* |
| GO:0048869 | cellular developmental process | 0.020097176364210167 | *APBB2,NFIA,THRB,SORBS2,GRIP1,ZNF536,ARID1B,TBL1XR1,KCNQ3,COL12A1,PTK2B,PRKG2,ZMYND8,ALDH1A2,CHL1,PAK3,EPHA3,ADGRB3,KLF7,KMT2A,ZFHX3,HCN1,CECR2,RARB,MEGF10,GABBR1,NEDD4L,CFAP44,WNT5A,ESR1,SLC7A11,RERE,EFNA5,AKAP6,CLDN1,TSHR,ETV1,EYA2,LDLRAD4,PPARG,TANC1,CDK6,ZNF521,ANGPT1,SPATA25,LAMB1,NFIB,PRKD1,TTN,BCL11A,ATP8B1,VPS13B* |
| GO:0032501 | multicellular organismal process | 0.020207157133872403 | *APBB2,NFIA,THRB,CACNB1,SORBS2,GRIP1,ZNF536,ARID1B,CACNA1C,TBL1XR1,MBTD1,TBC1D32,KCNQ3,COL12A1,AFF3,PTK2B,PRKG2,HMCN1,ZMYND8,ALDH1A2,CHL1,PAK3,EPHA3,ADGRB3,KLF7,SHROOM3,KIF18A,KMT2A,ZFHX3,LHFPL3,HCN1,TLR1,CECR2,RARB,MEGF10,GNAO1,GABBR1,NEDD4L,WNT5A,ESR1,SLC7A11,BBX,RERE,EFNA5,AKAP6,SHROOM4,NCOA2,ACACB,DLC1,CLDN1,TSHR,ETV1,EYA2,TRPV6,LDLRAD4,TLR6,CAMK2D,KIF16B,PPARG,TANC1,CDK6,IL1RAP,ZNF521,ANGPT1,PHLPP1,AFAP1L2,LAMB1,SBF2,NFIB,PRKD1,TTN,BCL11A,ATP8B1,VPS13B* |
| GO:0023052 | signaling | 0.02182390569989231 | *APBB2,CDK14,PDE1C,NFIA,MAOA,THRB,MAPK10,CACNB1,SORBS2,GRIP1,DLGAP1,ZNF536,CACNA1C,TBL1XR1,TBC1D32,KCNQ3,PTK2B,PRKG2,ZMYND8,ALDH1A2,UBR5,CHL1,PAK3,PRICKLE2,ADGRD1,EPHA3,ADGRB3,KLF7,KMT2A,RASGRF2,DAPK1,PELI2,TLR1,PLD1,RARB,GNAO1,GABBR1,WNT5A,ESR1,SLC7A11,NR3C2,EFNA5,AKAP6,MX1,NCOA2,DLC1,RGS17,TSHR,EYA2,TRPV6,LDLRAD4,TLR6,CAMK2D,KIF16B,PPARG,CDK6,STK32A,IL1RAP,TRERF1,RAPGEF4,ANGPT1,PHLPP1,AFAP1L2,LAMB1,PRKD1,PDE11A,TTN,SRGAP3* |
| GO:0006366 | transcription by RNA polymerase II | 0.024238441323831832 | *APBB2,MED13L,NFIA,THRB,ZNF536,ARID1B,TBL1XR1,ZMYND8,KLF12,ZNF429,KLF7,ZNF280B,KMT2A,ZNF607,ZFHX3,RARB,WNT5A,ESR1,BBX,NR3C2,ZSCAN23,ZNF615,NCOA2,ZNF860,ETV1,ZNF197,ZNF665,CAMK2D,PPARG,CDK6,TRERF1,ZNF521,IKZF2,NFIB,PRKD1,BCL11A,NPAS3* |
| GO:0032502 | developmental process | 0.02699622499494841 | *APBB2,NFIA,THRB,SORBS2,GRIP1,ZNF536,ARID1B,CACNA1C,TBL1XR1,MBTD1,TBC1D32,KCNQ3,COL12A1,AFF3,PTK2B,PRKG2,ZMYND8,ALDH1A2,CHL1,PAK3,EPHA3,ADGRB3,KLF7,SHROOM3,KIF18A,KMT2A,ZFHX3,HCN1,CECR2,RARB,MEGF10,GABBR1,NEDD4L,CFAP44,WNT5A,ESR1,SLC7A11,BBX,RERE,EFNA5,AKAP6,SHROOM4,NCOA2,ACACB,DLC1,CLDN1,TSHR,ETV1,EYA2,LDLRAD4,KIF16B,PPARG,TANC1,CDK6,IL1RAP,ZNF521,ANGPT1,PHLPP1,SPATA25,LAMB1,SBF2,NFIB,PRKD1,TTN,BCL11A,FMNL2,ATP8B1,VPS13B* |
| GO:0120036 | plasma membrane bounded cell projection organization | 0.03043841416508366 | *APBB2,GRIP1,TBC1D32,PTK2B,ZMYND8,CHL1,PAK3,EPHA3,ADGRB3,KLF7,CECR2,PLD1,NEDD4L,CFAP44,WNT5A,RERE,EFNA5,TSHR,ETV1,TANC1,LAMB1,NFIB,PRKD1,BCL11A,ATP8B1,VPS13B* |
| GO:0048513 | animal organ development | 0.03279289342748768 | *NFIA,THRB,SORBS2,CACNA1C,TBL1XR1,TBC1D32,PTK2B,PRKG2,ALDH1A2,EPHA3,KLF7,KIF18A,KMT2A,ZFHX3,HCN1,CECR2,RARB,MEGF10,WNT5A,ESR1,SLC7A11,BBX,RERE,AKAP6,SHROOM4,NCOA2,ACACB,DLC1,CLDN1,TSHR,ETV1,LDLRAD4,PPARG,CDK6,ANGPT1,LAMB1,NFIB,TTN,ATP8B1,VPS13B* |
| GO:0006351 | DNA-templated transcription | 0.03312782020143578 | *APBB2,MED13L,NFIA,THRB,ZNF536,ARID1B,TBL1XR1,MBTD1,AFF3,ZMYND8,KLF12,ZNF429,KLF7,ZNF280B,KMT2A,ZNF607,ZFHX3,RARB,WNT5A,ESR1,L3MBTL4,BBX,RERE,NR3C2,ZSCAN23,ZNF615,NCOA2,ZNF860,ETV1,ZNF197,ZNF665,CAMK2D,PPARG,CDK6,ZNF234,TRERF1,ZNF521,IKZF2,AFAP1L2,NFIB,PRKD1,BCL11A,NPAS3,ATP8B1* |
| GO:0003300 | cardiac muscle | 0.0336450329516993 | *SORBS2,AKAP6,ACACB,CAMK2D,PPARG,TTN* |

| | | | |
|---|---|---|---|
| | hypertrophy | 9 | |
| GO:0045893 | positive regulation of DNA-templated transcription | 0.03403235058129449 | *APBB2,NFIA,THRB,ARID1B,TBL1XR1,MBTD1,ZMYND8,KLF12,KLF7,KMT2A,ZFHX3,RARB,WNT5A,ESR1,RERE,ZNF615,NCOA2,ETV1,ZNF197,PPARG,TRERF1,ZNF521,AFAP1L2,NFIB,PRKD1,BCL11A,NPAS3* |
| GO:1902680 | positive regulation of RNA biosynthetic process | 0.03475061107451531 | *APBB2,NFIA,THRB,ARID1B,TBL1XR1,MBTD1,ZMYND8,KLF12,KLF7,KMT2A,ZFHX3,RARB,WNT5A,ESR1,RERE,ZNF615,NCOA2,ETV1,ZNF197,PPARG,TRERF1,ZNF521,AFAP1L2,NFIB,PRKD1,BCL11A,NPAS3* |
| GO:0051252 | regulation of RNA metabolic process | 0.03814813026013689 | *APBB2,MED13L,NFIA,THRB,ZNF536,ARID1B,TBL1XR1,MBTD1,AFF3,ZMYND8,KLF12,ZNF429,KLF7,ZNF280B,KMT2A,ZNF607,ZFHX3,RARB,WNT5A,ESR1,L3MBTL4,BBX,RERE,NR3C2,TENT5B,ZSCAN23,ZNF615,NCOA2,ZNF860,ETV1,ZNF197,ZNF665,CAMK2D,PPARG,CDK6,ZNF234,TRERF1,ZNF521,IKZF2,AFAP1L2,NFIB,PRKD1,BCL11A,NPAS3,ATP8B1* |
| GO:0014897 | striated muscle hypertrophy | 0.04122478606595102 | *SORBS2,AKAP6,ACACB,CAMK2D,PPARG,TTN* |
| GO:0009888 | tissue development | 0.045517165760548345 | *NFIA,THRB,SORBS2,TBL1XR1,TBC1D32,COL12A1,PTK2B,PRKG2,ALDH1A2,EPHA3,KLF7,CECR2,RARB,MEGF10,WNT5A,ESR1,SLC7A11,AKAP6,NCOA2,DLC1,CLDN1,EYA2,LDLRAD4,KIF16B,PPARG,CDK6,LAMB1,NFIB,TTN,VPS13B* |
| GO:0014896 | muscle hypertrophy | 0.04699995797502981 | *SORBS2,AKAP6,ACACB,CAMK2D,PPARG,TTN* |
| GO:0061448 | connective tissue development | 0.04751489122881557 | *NFIA,TBL1XR1,PRKG2,KLF7,RARB,WNT5A,NCOA2,PPARG,NFIB,VPS13B* |
| GO:0030030 | cell projection organization | 0.04819173063880867 | *APBB2,GRIP1,TBC1D32,PTK2B,ZMYND8,CHL1,PAK3,EPHA3,ADGRB3,KLF7,CECR2,PLD1,NEDD4L,CFAP44,WNT5A,RERE,EFNA5,TSHR,ETV1,TANC1,LAMB1,NFIB,PRKD1,BCL11A,ATP8B1,VPS13B* |
| | | | *CC* |
| GO:0030054 | cell junction | 0.00035808274645883866 | *APBB2,NFIA,CACNB1,SORBS2,GRIP1,DLGAP1,CACNA1C,KCNQ3,LPP,PTK2B,HMCN1,ZMYND8,PAK3,ADGRB3,SHROOM3,RASGRF2,DAPK1,HCN1,PLD1,GNAO1,GABBR1,WNT5A,BTBD8,EFNA5,DMXL2,AKAP6,SHROOM4,DLC1,RGS17,CLDN1,TANC1,IL1RAP,RAPGEF4,PRKD1,SRGAP3,BCL11A* |
| GO:0045202 | synapse | 0.0008756999452024536 | *APBB2,CACNB1,SORBS2,GRIP1,DLGAP1,CACNA1C,KCNQ3,PTK2B,ZMYND8,PAK3,ADGRB3,RASGRF2,DAPK1,HCN1,PLD1,GNAO1,GABBR1,WNT5A,BTBD8,EFNA5,DMXL2,SHROOM4,RGS17,TANC1,IL1RAP,RAPGEF4,SRGAP3,BCL11A* |
| GO:0098794 | postsynapse | 0.01614227984514413 | *GRIP1,DLGAP1,CACNA1C,PTK2B,ZMYND8,PAK3,ADGRB3,DAPK1,HCN1,GNAO1,GABBR1,WNT5A,SHROOM4,TANC1,SRGAP3,BCL11A* |
| GO:0098590 | plasma membrane region | 0.016143332495465276 | *SORBS2,GRIP1,CACNA1C,ATP4A,PRKG2,HMCN1,ADGRB3,SHROOM3,KIF18A,KIAA1614,HCN1,PLD1,GNAO1,GABBR1,SLC7A11,EFNA5,AKAP6,SHROOM4,DLC1,CLDN1,TSHR,ATP8B1* |
| GO:0071944 | cell periphery | 0.02145837362034994 | *CDK14,MAPK10,CACNB1,KCNQ5,SORBS2,GRIP1,DLGAP1,ARID1B,CACNA1C,FBLN2,KCNQ3,COL24A1,C2ORF88,COL12A1,LPP,PTK2B,ATP4A,PRKG2,HMCN1,CHL1,ARHGEF39,PAK3,ADGRD1,EPHA3,ADGRB3,SHROOM3,KIF18A,KIAA1614,LHFPL3,RASGRF2,DAPK1,HCN1,TLR1,PLD1,MEGF10,GNAO1,ASAP2,GABBR1,NEDD4L,WNT5A,ESR1,SLC7A11,BTBD8,EFNA5,AKAP6,RAB3GAP2,SHROOM4,DLC1,RGS17,CLDN1,TSHR,TRPV6,TLR6,CAMK2D,STK32A,IL1RAP,RAPGEF4,ANGPT1,PHLPP1,AFAP1L2,LAMB1,PRKD1,ATP8B1* |
| GO:0005886 | plasma membrane | 0.030390017089701446 | *CDK14,MAPK10,CACNB1,KCNQ5,SORBS2,GRIP1,DLGAP1,ARID1B,CACNA1C,KCNQ3,C2ORF88,LPP,PTK2B,ATP4A,PRKG2,HMCN1,CHL1,ARHGEF39,PAK3,ADGRD1,EPHA3,ADGRB3,SHROOM3,KIF18A,KIAA1614,LHFPL3,RASGRF2,DAPK1,HCN1,TLR* |

| | | | |
|---|---|---|---|
| | | | *1,PLD1,MEGF10,GNAO1,ASAP2,GABBR1,NEDD4L,WNT5A,ESR1,SLC7A11,BTBD8,EFNA5,AKAP6,RAB3GAP2,SHROOM4,DLC1,RGS17,CLDN1,TSHR,TRPV6,TLR6,CAMK2D,STK32A,IL1RAP,RAPGEF4,ANGPT1,PHLPP1,AFAP1L2,PRKD1,ATP8B1* |

**Table S6**. Differential analysis of the genomic interactions of rDNA clusters in Mel Z cells grown on plastic or on Matrigel. Differential 4C analysis was performed as described in the Materials and Methods section. The Excel file is attached separately.

**Table S7**. Venn diagrams show the intersections of rDNA-contacting genes possessing ≥ 30 contacts with nucleoli in Mel Z cells grown either on plastic or on Matrigel with 163 downregulated genes. Related to Figure 6A.

| Names | total | elements |
|---|---|---|
| 163 Mel Z Matrigel Mel Z plastic | 14 | *ENSG00000286540 MEF2C-AS1 EBF1 CPED1 INPP4B IGF1R NLGN1 Y_RNA ZFPM2 BEND5 PLEKHA5 SYNE2 SRGAP2 PLCB1* |
| Mel Z Matrigel Mel Z plastic | 644 | *AC092634.2 PAX7 RALYL LINC01201 FP236241.1 ABCC6 PTPRR DLEU7 EPB41L4B AC012409.2 LRMDA EPN2 PIR MED13L NFIA SUGCT DIP2A PRH1-PRR4 LINC03007 TRPM6 SLC8A1 RNF103-CHMP3 AL356108.1 ANO5 PCDHA13 ZNF385B LOC102724843 PREP AF127577.4 RSRC1 GRIK2 DPYD OTX2-AS1 SLC22A14 LINC03000 KDM4B FGF1 AC090809.1 ZBTB20 ARHGAP15 LOC101929710 LINC00486 LOC107984685 AHR AC022523.1 FSTL5 TTC3 CADPS2 TLK1 ASTN2 FAM9B LRP1B KALRN AC009262.1 MKLN1 USH2A SUMF1 NEGR1 XXYLT1 FGF12 BTBD9 PARP4 FRG1-DT PCDHA12 AC069228.1 MAST4 NRXN1 AC079801.1 AGBL1 MAGI1 GNGT1 AC036214.3 FARS2 ST6GALNAC3 DPP6 AC098588.1 FAM13A RNF217-AS1 DAAM2 STK24 AL365295.1 LMO7 GRM3 U8 EXOC4 LINC02822 AC078828.1 DNAH7 BBS9 ENSG00000290711 NTRK2 ANXA10 ANKRD34C-AS1 CHD7 ARL17B NBEA AL512598.2 RASGEF1B TMX3 LINC00273 MIR99AHG ENSG00000293415 NRG3 PTPRG CALCR ELMO1 ENSG00000287881 NCOA1 IRAG1 LINC01684 AL390957.1 ATP2B4 ROCR TOX2 PHEX SOX6 LINC03104 PDE4DIP TRPM3 FBLN7 TMEM64 DNAH8 CNTNAP5 PRANCR RAPGEF5 FGF13 DRAIC PATJ LINC01798 GPC3 AL512380.2 DMD ADCY2 DYNLRB2-AS1 KCNQ3 LINC02566 CDH19 LINC02966 ADAMTS9-AS2 GNG12-AS1 ATRNL1 CHRM3 ARHGAP24 LINC00907 SLIT2 TPTE2P6 ZNF804A FAM20C ENSG00000291181 AC010235.1 TTC33 LINC00347 AC098588.2 NALF1 KAZN BRAF DNAAF9 BABAM2 ENSG00000289084 KMT2C FKBP9P1 CU638689.2 LINC01320 STEAP2-AS1 ASMER1 B3GNT5 ERBB4 KIF26B GPHN ENSG00000293389 ATXN7L1 ZNF385D RAD51B DOP1B SH2D1A MYO3B ENSG00000287722 SH3RF3 EXT2 DIAPH2 AL138720.1 MAP3K5 STAG2 SPAG16 DSCAML1 HTR2C GALNT13 LOC101927609 CNTN4 TBC1D5 RAP1GAP2 LINC01830 LOC105369165 DMBT1L1 AC013652.1 CCSER1 PIK3C3 HMCN1 ENSG00000286033 LINC01695 LOC105373436 ITGA1 TCF12 SGIP1 ARMC8 ENSG00000289694 RUNX1 MYO16 ESRRG AC058822.1 AL161757.2 PCDHA2 FRG1HP PCDHA11 PCLO PRH1 SLC38A4-AS1 ENSG00000290357 CDH18 FRMD6 ANK2 LOC101927314 MIR100HG ANO2 PCDHA9 SUPT3H ROBO1 LINC01091 ENSG00000289002 NEK1 LINC01924 DGKB PWRN1 CDC42 OBI1-AS1 PRRC2C PCDHAC2 CDH6 AC109830.1 ENSG00000290397 DOK6 WDR64 C8orf34 JAZF1 BNIP3P41 CHODL ZNF267 LINC01692 FGF14 MIR325HG CACNA2D3 LINC03116 SLC8A1-AS1 PCDHA10 MUC19 EDIL3 LRRTM4 PTPRN2 SOX5 DSCAM DGKI LINC01151 COL28A1 DENND1B LARGE1 COLEC10 SDK1 PUS7 CCDC33 LINC02694 EPHA6 LINC02328 SPTB WDR72 TMEM260 IQSEC1 ENSG00000287916 ANK1 PPM1L CASZ1 CNTNAP2 TRIM71 ENSG00000286523 KCNIP4 SOX2-OT CFTR DYNC1I1 NOS1AP OTOGL DOP1A AC027031.1 PCDH9 NKAIN2 PKN2-AS1 RBMS3 OFCC1 AL603840.1 FP700111.1 SGO1-AS1 CFAP20DC CIBAR1-DT ATP6V0D2 PCDHA1 ZNF800 PCDHA8 CU638689.4 ENSG00000290551 TBCK AC108010.1 CDH12 SHC4 LINC02112 AC098650.1 FRMD6-AS2 TESHL CBR3-AS1 RPS3AP27 DDR2 ADAM22 LARS2 HULC GPC6 MCF2L2 TRPS1 ENSG00000293394 CDH4 ENSG00000288187 DPP10 CELF4 ST8SIA6 CNTN6 PARM1 AC005670.2 CU633906.2 FBLN5 SPEF2 ENSG00000286745 WDSUB1 ZNF638 ATP9B MITF CACNA2D1 NSMCE2 PCSK1 MIR4435-2HG PCDHAC1 HERC2 LRFN5 UTRN GPC5 ENSG00000290523 AGBL4 OGDH GHR ULK4 RBFOX1 PCDH7 LRRC4C AC084149.1 PPP1R9A TMEM117 AP001341.1 RNA5-8SN5 SMAD9 NKAIN3 DYNC1H1 CRPPA NEBL AC006148.1 NAALADL2 AC007161.3 OSBPL10 LANCL2 MRPS28 SLC35F1 TRD-AS1 LINC02306 FRMPD4 MEIS1 GALNT17 EDAR PMS2P4 CFAP20DC-AS1 BRINP3 RIMBP2 FHIT AC009093.10 WIPI2 DNMT3A TOX PCDH15 SGCZ ACE2 CHMP3 KCNAB1 PDE4D CNTN5 AL121718.1 ITGB8 PDE3A RIMS1 TRANK1 WWOX EGFEM1P PCDHA7 NEK10 ANAPC10 LSAMP MIPEP LINC01681 PWRN4 AC007100.1 DIAPH3 LINC01440 TMEM178B CPLANE1 NOL4 PCDHA5 PSG11-AS1 PLCB4 RNF216 FTO UGGT2 TNFSF11* |

| | | |
|---|---|---|
| | | *LINC02326 PPFIA2 ENTPD1-AS1 ENOX1 WNT2 ENSG00000292991 OR8B8 STEAP1B UMAD1 LINC00299 POU6F2 SFI1 MGAT4C DIRC3 ANKRD44 CALN1 UXS1 LINC01584 NTNG1 ZNF804B SGCD GRM7 AC007848.1 STS NAV3 AC091489.1 AC092343.1 AL110292.1 AC079943.2 LOC124903324 PTPRM ENSG00000286225 PRKCH NRXN3 DLC1 DENND2B LINC01500 MIR663AHG ATP8A2 CU633906.4 CERS6 AL591463.1 LPIN1 MACROD2 AP005328.1 ENSG00000287635 ADAM7-AS1 AUTS2 AC092042.3 LOC102724701 SYT16 BCAS3 ADARB2 ENSG00000288723 COMT PCDHA6 MCTP1 ENSG00000286110 NBAS ZPLD1 KMT2E LINC03060 DOCK2 SDCCAG8 SSH2 AL035078.4 LMNTD1 CDH13 MDGA2 AC103796.1 BDNF-AS DANT2 LINC01934 FBN2 DAB1 TTLL6 RFTN1 PCDHA4 SNTG1 ALK CACHD1 SEMA3A AC068051.1 YEATS2 CCDC26 SLC8A3 ENSG00000290983 MYO5B CADM2 MALRD1 TENT4B MAD2L1-DT AC010601.1 DCLK1 AC004917.1 FAM135B KIF16B CDH2 TENM2 VPS41 LINC00879 TEX13D HDAC9 KIAA1549L USP9X KCNB2 USP13 NBPF1 CDK6 ADGRL4 AC090376.1 LYST CADPS DDX60 LINC03105 RGS7 ENSG00000293037 LINC01470 TFAP2D AC067956.1 PPP2R2C HEATR4 SPECC1 PTPRT GABPB1 CSMD1 ENSG00000286746 SCEL CTNNA3 FREM2 RAPGEF4 CLTC THSD7B ENSG00000287404 DACH2 AC093515.1 SORCS1 CAMKMT RNF220 ARHGEF37 DLG2 SACS AFF2 PTPRD RORA AL355499.2 NPEPPS PRKG1 BRIP1 MIR924HG LOC107986400 MAGI2 NELL1 MIR4300HG CYP4F62P PLCL1 LINC02215 GALNTL6 PXDNL SCN2A ASIC2 DOCK10 MICAL3 ADK TMEM164 KMT2CP4 RANBP17 GTDC1 RYR2 SLX4IP LDLRAD3 ENSG00000286648 CU633904.3 PTPRA AC011287.1 FAT3 MSH2 PCDHA3 MGAT4A AC078845.1 KLHDC10 RGS9 AF241726.2 MPDZ AC016766.1 EYS SLF1 ROBO2 CR392039.3 ENSG00000288891 LINC02055 SLC39A11 LINC00378 AC026167.1 AC015687.1 PAX3 LOC105379109 KSR2 DLGAP2 BMPR1B PDE8B DCC CTNNA2 PEX5L ARL17A DNER ETV6 CEP112 NRG1 PGM3 DLEU1 SLC25A48 ENSG00000293385 AC092167.1 NPAS3 DNAH5 SATB1-AS1 FANCB LINC02208 NRXN1-DT* |
| 163 Mel Z Matrigel | 115 | *NHLRC3 TNS3 TEAD1 ELAPOR2 TOX3 RALY-AS1 CDC42BPA FAM218A ENSG00000286830 SLC46A2 R3HDM2 CHD1-DT TTC28-AS1 SRGAP2C ZFHX2 NR2F2-AS1 SETBP1 ADCY1 GRID1 ANO4 LINC00589 SRGAP2B DNPEP-AS1 ARHGEF35-AS1 KRT89P PDZD2 WEE2-AS1 ACOXL FNDC3B FHIP1A LINC00663 ZNF519 MACF1 STARD4-AS1 TFDP2 LRRC37A2 ANKRD28 ZNF609 TSHZ2 EOLA2-DT ZNF516 ZEB1-AS1 SFMBT2 FCHSD2 SLC4A7 MTUS1 KLHL32 TMEM100 CACNA1D IVNS1ABP TMEM150C COL4A4 ANKFN1 WNT2B AZIN2 ANKRD27 ANK3 ZNF19 TEX21P ARHGEF10 VAV3 LINC01252 CYP39A1 LRRC37A4P MBD5 STON1 DISC1 FMN1 ZNF827 DSE NAV2 RNF150 GNG2 FRS3 ACKR2 MCTP2 ZFPM2-AS1 SSBP2 ARHGAP32 SPRY4-AS1 PABIR3 BAZ2B CAPS2 CAMK4 PCYOX1L ATXN1 PLEKHG1 COL27A1 ESR2 RASSF3 ZNF221 CNTNAP4 TUBA3D GLI3 ENSG00000287729 GAB2 CREB5 ADGRG6 EPB41 DOCK9 PPARA NCKAP5 EDA CNTNAP3 RBM47 SEMA3D FOXP1 SCN8A ZRANB2-DT AGAP1 RIPOR2 PARD3B HSPG2 FRMD4B GOLGA8B* |
| Mel Z plastic | 2119 | *AL157886.1 FSTL1 CDH12P2 CASC20 C10orf90 APBB2 AC243829.5 PFKP MIR3171HG AC012501.1 ESYT1 STYK1 RPL19 HS3ST3A1 RTN1 RNU7-188P AC100775.1 RGN TRGC2 CDK14 ZSWIM1 MSRB3 PDE1C ENSG00000293331 MREG HNRNPCL4 NOSTRIN ING3 ENSG00000290149 MAP3K3 NINL WSCD1 ENSG00000287515 OSBPL8 SLC12A8 LRRN1 HERC2P2 ENSG00000286309 ENSG00000291144 LINC00698 POTED STX8 AC032019.1 PHF20 FOXK2 CPNE4 TXNDC8 RBM17 AC021517.1 SMYD3 ARHGEF26-AS1 GARS1 RAPGEF6 ARHGEF9 TAFA2 ABR KRT8P32 TLR8-AS1 ISM1 MAOA BLTP3B LINC01163 AC012355.1 LINC02438 HYDIN2 ENSG00000287308 HCCAT5 TRAPPC9 PCDHB1-AS1 SLC16A5 LINC01545 FNDC1 TMEM255A MAP3K13 EIF1AX PPM1A ARL13B RPL15P21 SAMSN1 RN7SL556P C12orf75 AC010291.1 THADA LIMD1 IMMP1L TF SNX10-AS1 CHST11 THRB MYBPC1 USP42 IPP RCSD1 SPTBN1 UBTFL3 AF228730.2 CDC42EP3 ENSG00000286111 AC013472.2 AC021269.2 MIR3117 MYO5C LINC00841 MAPK10 ITPRID1 CACNB1 USP32 AHCYL2 DAPP1 ATF7 CD101-AS1 GNG12 CHRNB2 CDC14C SEZ6 CLDN6 LINC01967 KCNQ5 CDH8 GNB4 AGTPBP1 AL356490.1 ATF7-NPFF FHL1 TRMT6 PPP1R37 RBPMS ATP10A LINC02457 SH3PXD2A SPINK5 MRPS24 OPA3 SKAP2 PRND PIGL ATP7B PPM1H GPBP1L1 RPS6P19 KCNJ6 PCAT1 RPL21P82 BRD4 ZAN DOK4 AC007405.2 LINC00517 AC092378.1 TNRC18 MIR9-3HG LOC102724019 LINC02828 CAMK2B GRIP1 CASC19 ELMO1-AS1 ZNF962P OR4N3BP ARHGAP11A-SCG5 LOC124901321 URGCP-MRPS24 DDI2 AC008764.4 GPATCH8 PGM1 DCT AC073071.1 DLGAP1 FAM193A LNCATV PECR LRP5L SLC38A9 LINC01982 CDRT15P9 LINC00348 ZNF264 QRSL1P2 LINC02133 HBP1 SDR16C5 RGPD2 ABCG8 XPO4 OTUD7A AC060234.3 AL583785.1 AC107909.2 OPN3 MAP4K3 AC092078.2 FAM157A LINC01194 GALNT9 FPGT-TNNI3K SNORD114-10 LOC442028 NOTCH2 CD93 LOC105379362 FKBP5 TMOD2 SLC44A5 SDK2 HMBOX1 SIM1-AS1 AP003900.1 ZNF551 LINC00882 RNU2-49P NPNT AC019211.1 DSC1 EPB41L3 MAP6 AC012363.2 PARVB MORC1 CDH11 RN7SL266P SHANK3 WDR26 NR4A3 CYP2U1-AS1 SPTLC3 GAS1RR AGAP12P LINC00494 AC005154.4 ARID1B LOC101927948 DPY19L2P2 LOC105375387 ENSG00000289752 AMYP1 E2F7 ACVR2B-AS1 L3MBTL4-AS1 SLC14A2 ENSG00000291166 FAM169BP AC091304.2 FOXO1 LINC02311 ZNF611 STYXL1 AC244517.9 LINC02156 STK39 PDE11A-AS1 SMURF2 SRL ADAMTS12 FER1L6 TBC1D16 PLXNA4 AL592078.1 LINC02542 LINC00504 LOC124900810 CDK18 URB2 YBX1 ARMT1 DIPK2A CFAP70 DCDC1 CACNA1C FAM20B ENSG00000287410 AC046195.1 FAM3C SNORA5B ASB4 IDO1 AC026786.1 AC015922.1 ARB2A LOC105370489 PRKN ENSG00000286937 CNN3-DT NLGN4X RMND5A LINC02044 HSD17B2-AS1 PRDX3P3 PNPLA3 LINC00320* |

| | | |
|---|---|---|
| | | AC092447.10 F13A1 ENSG00000288041 TAFA1 PIK3C2B XPO6 AC103876.1 KDM7A SLC44A3 SLC5A7 GNA14-AS1 MARCHF9 FAM83B IFT172 AL136317.2 RNU6-258P GPSM1 AL121900.2 CBLL2 AL136372.2 PTBP3 ZBTB44-DT FAM117A CDON SERPINI1 SLC17A6 NLK SDF4 THSD7A OVCH1-AS1 GNAI1 LINC02405 SPATA31D2P MRPL13 AC138932.2 MAPKAPK3 NCAM2 CHML PAPOLG H3P38 TIAM2 INTS6-AS1 CFAP54 ZNF10 RORA-AS1 AC015804.1 TRAK2 AL139383.1 ENSG00000293038 CNMD ENSG00000286229 HERC2P3 LRP8 TAGLN3 AC018767.3 LINC00992 CNTN1 AC002451.2 AGL MEOX2 MAPRE3-AS1 STAT5B ENPEP LINC01242 NUDT19 ARHGEF4 LOC102724428 UBE2O MUC17 DDIAS AC025884.1 MTUS1-DT DUXAP9 AC092100.1 CPEB4 EIF4ENIF1 HEATR9 LOC124900848 CHRFAM7A ERBIN RN7SL430P CR383658.2 ZNF121 HERC2P9 MBTPS2 RGS22 PLS3-AS1 LINC00598 CD247 ENSG00000290948 ENSG00000287621 CEP192 MCC TRIM52-AS1 PTH GEMIN8P1 ENSG00000287008 ADAR DNAH10 PDE10A TM9SF2 ENSG00000286250 PDHX CLCN3 RHOJ CDC37 FGF10 ZFYVE16 JAZF1-AS1 MECOM AC009878.1 RSPH10B COL4A6 CATSPERB ENSG00000287474 TSPAN18 SAMD15 NLRP8 ENSG00000286800 FRG1JP STK32B PLG NEURL4 POLR2J3 HERC5 LINC02505 LOC124900957 NUDT13 AC008109.1 CDH7 LOC101929457 ENSG00000290585 PGPEP1L UBE2E2 ILDR2 SCEL-AS1 AC034229.1 MGC27382 NCK1 PUDP ENSG00000287744 CD84 FAM168A NONO RARRES1 AC138512.1 KLHL42 NREP AF064860.1 EXO1 CCDC71L GABRA5 AL591684.2 ARHGAP23P1 EBNA1BP2 SNORD114-9 NEXMIF ENSG00000286274 LOC401913 ENSG00000288755 MTCP1 ASTN1 SH3BGR MROH6 TRAJ17 AC104248.1 AC004492.1 AC231532.1 KDM2B PREX1 HEY2-AS1 RGPD3 AL133500.1 PRIM2BP AIG1 ERICH1 IGLV1-41 ZNF404 PMS1 CDIN1 WRNIP1 ABCC13 COL24A1 TYW1 ANO3 GOLGA8R AC009511.1 COL11A1 AC119677.1 AC112721.2 AC113391.2 RSF1 AC022509.1 NCOA7 KCTD3 AC063949.2 C2orf88 RNU2-33P SERTM2 SEPTIN10 CIDEB MARCHF1 OR4N3P ENSG00000286376 ENSG00000286922 SLC45A1 AL773545.1 MCPH1 ENSG00000289143 MYO1D ENSG00000287045 SESTD1 ENSG00000291338 LINC01661 ENSG00000288553 ZNF214 COL1A2 DTNA TMOD3 ENSG00000286020 LOC340512 GEMIN5 EEF2KMT MRTFB ENSG00000286147 XPO7 KCNS3 AC011447.3 ENSG00000288902 VWA3B RPL34P11 FREM2-AS1 ENSG00000287608 ENSG00000290114 COL12A1 ABLIM1 ENSG00000287763 AC009226.1 RBMS1 ALPK2 CHRNA7 HECW1 HMGB1 ZNF304 DYNLT2B AC021733.1 CDIP1 AFF3 RFLNA AL158198.1 AC062039.1 LPP HIVEP1 ATRX TNIK STK24-AS1 UBTFL5 LOC105371956 TGFB2 MDFIC EPB41L4A LAMA2 ADD3 TRIO FBXO16 ZNF85 LINC00269 ENSG00000291054 LINC00366 HOXC13 AL049875.1 SMARCC1 NDST1 LINC00355 MUSK KRT223P ZNF73P KCNJ18 JAM3 ENSG00000287741 KCNIP3 SFPQ AC027338.2 PNPLA4 AC004485.1 ALKBH1 SNORA5A ARPP21 AF130359.1 RGS3 ZNF299P DUXAP8 NUP93 LDHAL6DP AC108673.3 ZCCHC4 MRPS9-AS2 ZNF407-AS1 RERG G2E3-AS1 ESRRAP2 OR5AU1 PRKAR2A FILNC1 RBM33 AL604028.1 CTIF CFAP57 ENSG00000286902 FADS2B CALCRL-AS1 ATP11A AC023389.1 MRPL33 RBM23 AC008695.1 ZNF154 ATP4A CSTF3 SLC15A4 HIGD1AP9 ENSG00000288087 AL024474.2 AC244517.11 CERS6-AS1 CDH12P4 PCDHB8 GOLPH3 AC015908.7 PRKG2 MBTPS1 NCF2 PIGV RNU6-1216P USP10 SND1 EPIC1 ENSG00000288692 RN7SKP141 IGFBP7 RSKR EPHA5 MACROD2-IT1 RABGAP1L-DT LINC03076 PTPN20 ENSG00000290849 AC087501.3 LRRK2-DT SKOR2 AC130650.1 CBR4 CRLF3P2 ATP13A4 AL162718.1 AC009055.2 ENAH SNX10 ATP2C1 TMEM230 ALDH1A2 AL354718.2 PPTC7 STON2 MIR3681HG PTGFR IGHV1OR21-1 AL138895.2 FAM171A1 STRADA LINC01725 HUWE1 ZNF595 ASPH RRAS2 SH3BGRL ZDHHC4 UBR5 AP000894.1 AC114781.3 AC096558.1 GTF2F2 AC105031.2 SIM1 KIAA1217 ABTB2 SOX30 AC244131.2 TMEM132C ANKS1B CHL1 TRAJ16 SPOCK3 GSN PLS1 AC007991.3 TCP11L1 SPON1 MAPRE3 MICB-DT TLR8 HPS3 AL391361.1 AC011447.2 KIF21A LOC124900600 SLIT3 ARMH3 FMO8P CACUL1 NLRP7 ARHGEF39 CD226 PHC2 MITA1 AC142086.6 ZNF578 ENSG00000290721 ENSG00000287021 ANKRD11 ITPR1 AL162254.1 PKP4 AL160286.3 CPM AC105052.4 DNAAF11 ENSG00000288069 PAK3 LINC01033 NDUFAF7 AC007846.2 ABI3BP RNU1-83P KLF12 LOC124900504 AXDND1 AC011824.3 IPMK LINC03042 AC073575.2 LINC01376 CYP2A13 RPL23AP51 TMEM183A RERG-AS1 AC126763.1 ENSG00000289956 CAMK1G PRUNE2 CASC2 SERPINB9P1 LINC01122 RNA5SP96 ENSG00000288694 AC073130.2 PRKAG2 LINC02965 ENSG00000290490 LINC00158 MLLT3 CCNT2 BTG4 HAVCR1 DRG1 RSPH10B2 TRAPPC10 LINC02942 ENSG00000288714 AC034206.1 CPQ CNOT10 CPEB1-AS1 MR1 KRTAP21-2 LOC102724710 F8 TNFRSF12A OASL GDPD1 LINC00237 CPT2 AP000320.1 TAS2R14 VPS8 AC010329.5 MTX3 LINC00703 AC092121.1 ENSG00000286878 C4orf50 NXT1-AS1 LINC01732 AC020743.2 ISY1 ENSG00000287211 ZNF831 ZNF429 AC242426.2 VN1R31P CXCR5 NCKAP1 PRPF40A CLDN12 KRTAP13-6P DTD1 CNTN3 MGAM LINC02427 ENSG00000289085 LINC01060 CILK1 HOXC13-AS FOSL2 PACRG IL1RAPL2 ADGRD1 HNRNPCL3 AC020912.1 EPHA3 CHCHD3 ZNF337-AS1 ENSG00000289131 LATS2 LINC02141 TYW3 MYH14 AC008581.2 APCDD1L-DT SPDYE2B MRPL32 TPRG1 ENSG00000286168 KLHL33 ERP29 CDYL2 ARHGAP18 AC013652.2 UVRAG CACNB4 SMIM10L2B-AS1 MYOM2 AC113386.1 LYN ADGRB3 USP6 LOC100131779 KLF7 LINC00390 MIR4636 LOC102723883 SUMO2 GRIK1 MTA3 CTSLP4 HSD11B1-AS1 LINC02203 UBL3 DIP2B LOC107985126 NME7 SEC24C AC068234.1 AC073869.5 MIR9-1HG AC109583.3 PRAMEF9 ENSG00000286686 ATP6V1H AC009135.1 NUP58 FAM177B FIGNL1 SLC12A1 LINC01538 AC068547.1 PDZD8 PAMR1 AC003006.1 CA10 LINC01878 RRM2 CRYBB1 SIL1 LDB2 MYRFL AC008035.1 AC024610.2 UNC93A MIR3159 C21orf62 LOC84214 ALDH1A1 NMNAT3 FMO5 RNASEL AC010082.1 LINC02008 NAV1 CMKLR2-AS DBF4B PAQR5 DSCAS CORIN TMCC1 LRRC7 PCDHB16 TEX41 LINC00458 AC060765.1 AC027288.1 CDH12P1 DCP2 RBPMSLP |

| | | |
|---|---|---|
| | | *PDZRN4 SGMS2 FAM151B-DT TMED7-TICAM2 STX17-DT ENSG00000288055 SLC41A2 NOS3 FER1L6-AS2 AL355306.2 LIPE-AS1 HMGB3P30 LOC105378402 VSX1 ZNF280B EIF3L NOMO2 TNRC6C NALCN-AS1 ZNF90P3 PRAMEF27 LINC01208 PGAM1P5 MLLT10 BPHL ERCC6 EIF2AK4 LINC00840 RN7SL738P IGKV2OR2-2 KIAA1614 AC016027.4 NIHCOLE MSRA AL031599.1 LOC100419786 SYNPR RAB12 SIMC1P1 LINC02235 C21orf62-AS1 LINC01505 IMPG1 HMGCLL1 LINC01911 DSCR4 XKR6 LOC101928335 ENSG00000287682 MAGEL2 AF165147.1 MATN2 TGFBR2 AC141257.2 CMKLR2 ITGA11 CFAP77 DDX60L MARK4 MDN1 AC092131.1 LINC01901 SYNJ1 TTC6 TCF7L2 LOC102724421 LINC02192 AC114781.2 CHAF1B ENSG00000290067 KIAA1328 ENSG00000286326 ZNF462 MTRF1 ECHDC2 AC090386.2 OVCH1 CRTAC1 E2F6 ZFHX3 ENSG00000291336 CUX1 POLR2C UNC119 SHISA2 GPM6B RRAGD C2orf50 ULK3 AC083902.1 LINC02484 LOC339166 SPDYE2 GPR156 ZMAT4 CNOT10-AS1 ENSG00000289949 AP000529.1 AL445224.1 TUBBP3 LINC01596 GKAP1 CACNA2D4 LHFPL3 RGPD1 PPP2R5E AL161751.1 ENSG00000288954 RPL39P33 KPNB1 INHBA-AS1 ENSG00000290385 AC010332.2 TTN-AS1 GSDMD ADGRV1 RASGRF2 ST7 SERBP1 DNM1P33 AC013401.1 QTRT2 EVA1C AL161716.1 EPB41L5 GAD1 SCG5 AL035078.2 NUDCD1 AC068254.1 MIR603 AL050327.1 WAC WHRN OCA2 AC093668.1 DAPK1 TDP2 ULK4P2 ALDH9A1 LOC100419045 RAB6C-AS1 LINC01362 ZNF678 ACSM2B PRUNE1 TUBB8P6 SAR1B TTC7B INSYN2A BTG3 APP GATA4 ATF7IP2 SMAD5 IGF2BP1 PPIL6 AC116634.1 MAMLD1 LINC02610 RPL9P14 RNLS AC009093.4 ENSG00000291100 ANKRD20A7P AL163932.1 GSKIP AL136985.3 NEPRO-AS1 RNF38 ADAM12 LOC107986837 UBE2G1 NBPF12 AC138969.2 PELI2 MIR1273F BTG3-AS1 DHX35 ENSG00000287347 SP140L HCN1 CRACDL ENSG00000286288 CHRM5 SLC13A1 PI4KB AC129926.2 FRY LINC00670 RHOT1 ELOVL5 AC010809.1 AL354810.1 USE1 HOOK3 AL513323.1 PIK3R5 PALLD LYZL1 TLR1 LINC02767 CECR2 TAS2R30 TMCO3 CASC16 RFESDP1 BMT2 AC233699.1 OR52P2P RIPK4 LINC01876 AVPR1B LMAN1L ENSG00000286406 AC091073.1 GOLGA6L10 QDPR FRRS1 IQCM PTK2 AP002373.1 AC006482.1 ENSG00000293467 AC079414.1 NOP9 USP7 IGLV3-2 TICAM2 ENSG00000293472 SCP2 NPEPPSP1 SH2B2 LINC02518 ATP1B4 AL031963.1 AVEN AC019270.1 AC006153.1 ZNF41 LINC02250 AC046185.1 GATD3 CELF1 HYCC1 DNAH12 POTEG POGZ LINC01090 PIP4K2B PRRC2B SYNM-AS1 UQCC1 ENSG00000288996 LRRK2 INO80D AF279873.3 ZNF395 IKZF3 GET1-SH3BGR SLC38A4 AC006043.1 AC244517.4 MIR548XHG FRY-AS1 CLSTN2 APRG1 ARIH1 SAMD12-AS1 CFAP298 PLD1 AC015908.2 VTCN1 ITFG2 PKHD1 ENSG00000287039 MEG8 TENT5C RARB UBXN7 KIF23-AS1 GDNF-AS1 LINC02435 AC104169.1 ENSG00000290429 PJA2 SNORD114-7 TCF4 AC079742.1 ENSG00000287334 MGST1 RBM44 ARHGEF38 PAEP COP1 TIAM1 PCA3 CSRP3 PHACTR1 MLIP IQSEC2 ACVR2A GNAO1 AC243830.3 AC034232.2 AL033530.1 ASAP2 LINC02309 TIPIN VIT LINC01324 AC091078.1 BAALC-AS1 AL606760.2 RASSF4 C6orf58 LOC124902439 ENSG00000288635 MIR6882 AC092979.1 SNORA5C SNTG2 AC060834.2 NTM AC091230.1 AC091133.4 AMMECR1 AC004805.1 NEDD4L FP236315.2 CYP1D1P LINC00639 TMED11P ENSG00000289368 BRCC3 CDH12P3 ENSG00000288683 AC093010.2 LINC00636 FECHP1 DUSP14 LINC01237 PRMT8 SNX13 AC090365.1 AC093893.1 RABGEF1 AC068724.3 KIT CYB5R4 TBC1D8 ENSG00000273937 EPHA7 ENSG00000291067 OSBPL3 SPAG9 AC104574.2 ANTXR1 ENSG00000293304 RMEL3 LOC100652967 AC131254.2 AC069257.3 LINC02256 C19orf47 AC068299.1 AC007326.2 SPDL1 WNT5A TRABD2B PEX26 TEX2 ARFGEF2 CTNNB1 AL671862.1 ANKRD17 EFCAB5 POTEKP AL096701.3 ENSG00000286811 COL19A1 RUNX1T1 CTSK LOC102723341 PRKDC AGAP4 AC097626.1 LINC00507 IPO5 ESR1 AC026124.1 LOC124900584 TNFRSF19 ZNF479 AC091045.1 LUZP2 RPL3P1 PTPN14 LINC02240 SCFD1 ERC2 POFUT2 LINC02973 DMGDH BCAR3 TPD52L1 L3MBTL4 PPM1E ZXDC AD000090.1 VPS53 LOC100129404 FRMD4A AC099788.1 CTSLP6 LMX1A DCAF8L1 MIR3670-3 LOC128462377 TPST2 FIGN CIMAP2 BTBD8 PARS2 AC103740.2 BNIPL HUNK TRAJ19 LINC01448 LINC00824 SNX29 FILIP1 LERFS TMEM62 SLC44A3-AS1 COPB2-DT FBXO15 EEDP1 PTGER4 AC018467.1 GREB1L AC015574.1 ZNF215 ENSG00000287392 PDE7B BBX AC096711.2 USP40 PPP1R15B-AS1 RASSF8-AS1 VWC2L BEST3 LYPD6B DOCK1 HDDC2 ENSG00000287180 AC131902.1 AC027644.4 CCDC107 TARDBPP2 AC005580.1 GPC4 BPGM PKIG ENSG00000286614 LINC00276 RPL21P10 EFNA5 LINC00536 AC073316.1 IGF2BP2 AL035420.1 AL391869.1 ACTR3B AC018638.8 CDC14B ZNF722 SYNE3 ENSG00000287684 LINC02808 PLCH1 OR1M1 HECTD1 VKORC1L1 NGEF TRPC5 NEUROD2 FNIP2 ZIM2-AS1 AC097478.1 AASS JAML KAT14 CCNJ UBE3B ADORA2A-AS1 PTPRF LINC01473 CASC15 IKBKB AC010280.1 DUX4L51 FXR2 SNHG33 SH3BP5 AC010809.2 AC068413.1 AKAP6 MIR3670-4 LOC101927690 ENSG00000293257 AC103409.1 TRBV10-2 USF3 AC002074.1 LYVE1 APOH AC004870.4 ARHGEF28 RAB3GAP2 CLCN1 TASOR CCPG1 AC104781.2 GPRIN1 EML6 OPCML LINGO2 CEMIP XIST AC074286.1 ATF2 LINC00877 AC140481.3 AC091151.1 RNU6-986P KCNJ12 ZNF426 HDAC8 ACVR1 CHRNB3 HLA-DQA2 PID1 COL6A5 HYAL4 EBF2 CCDC91 GRID2 ZNF423 ZCCHC17 KCNJ3 AC008632.1 AP000487.2 GLDC PLPPR1 SNORA63D SLC17A6-DT RB1 LINC00466 NSMCE1 AC078923.1 MPV17 LOC100131635 IFNLR1 SMIM2-AS1 RNU6-826P GOLGA6L4 PDE4B AL365214.3 MYHAS DDX10 LINC02994 PKD1L3 KIF13A NLRP4 ADIPOR1 ENSG00000286655 SLC23A4P LOC124900205 GABRB3 ZNF143 GOSR2 PET117 SHROOM4 AC093865.1 ZSCAN23 SMG7 PLPP3 UNC80 HNF1B RNU6-1311P IMMP2L ZNF615 AP001599.1 LAMP2 CCDC54 SSPN SPSB1 KIRREL3 GABRG2 F5 SCG5-AS1 CFAP95 AK5 CPS1 NCOA2 TMEM120A AC023300.1 AC104984.2 BNIP3L ENSG00000287614 ACACB AC021088.1 AGPAT5 RABGAP1L LINC01948 ENSG00000286476 LOC101927141 LINC00603 XRCC5 TBRG4* |

| | | |
|---|---|---|
| | | *LOC107984536 RGS17 HMGB3P2 LINC01965 SHLD2 GFRA1 SPHKAP KCND2 INPP4A BMX OR5W1P TRAJ18 ZNF418 SBF2-AS1 AC012593.1 SLC24A3 RN7SL563P RAD54L2 MPRIP LINC02351 TAF15 TPST1 GRIA4 RNA5SP489 RAB18 POLD3 AP000526.1 ZNF860 TPH2 POU2F1 AL079305.1 AL445648.1 LINC00508 PRPF40B AC244517.6 AC004870.3 TMTC1 AL592490.1 ETV1 NRK RGS7BP BACH2 MGRN1 LOC339298 AC010745.2 TEX11 LINC02384 MID2 AC087683.2 RBM28 MIR198 LRTM2 LOC105375972 SIK1 ZCWPW2 FBXL13 OR10J4 GLOD5 ERCC6L2 ENSG00000291293 ENSG00000286779 PIPOX RPS2P1 AP002336.2 ZNF850 TP53BP2 LINC00592 KAT2B ESPNP LSINCT5 GOLPH3L TMTC2 WLS ACTR3C ANKS1A IGKV1-6 RYR3 TMPRSS11B PRCP AL035446.1 SUPV3L1 MEF2D MYO1B MGAM2 MYL4 RPL39P31 AC091588.2 GTF2A1 SPTLC1 AC015922.2 AL137076.1 AC008758.4 AGR3 CDK5RAP2 FLVCR1 LINC02343 AC023830.3 UBE2E3 FSIP2LP SAMD3 RPL15P18 RFC3 EPN2-AS1 FARSB ERICH6B CCDC54-AS1 ARHGEF7 AL445430.1 ANKRD26P1 SAMD12 ENSG00000293462 DACH1 CU634019.3 RPL21P5 LINC03082 ATF3 TRDN LINC01117 LOC105377043 NPHP1 ENSG00000290597 GASK1A AL136146.2 LINC00499 AC111152.2 TEKT4P2 ENSG00000290412 IKZF1 DAOA-AS1 AC005154.5 SLC26A8 CAPZA2 AC004147.4 GGT8P AC011405.1 TRAJ20 AC002066.1 SRI ATP1B1 GREB1 AL158154.2 ADAT2 ARNT BDNF LDLRAD4 TFCP2L1 ACBD6 SEMA3E BCLAF3 HAS2-AS1 SCIN AL163195.3 ENSG00000293265 AP004609.1 MACO1 ZNF665 TLR6 ENSG00000293512 ENSG00000287443 CTBP2P10 NDST4 ADAMTS9 ADGRF2P STIM1 ITPR2-AS1 TSEN15 TMEM237 TMEM161B ENSG00000288563 HAUS6 RAB11FIP2 EEFSEC COX7B2 LAG3 DPP10-AS1 ENSG00000291189 Z95331.1 PPARG LOC124902888 TMEM72-AS1 AC016573.1 TANC1 PAPPA LOC101927605 IRAG1-AS1 ENSG00000290217 ASB3 AC239585.2 MTTP GPR84-AS1 CHRNB4 LOC102724934 AC019322.1 AGAP9 CTBP2P9 AP000282.1 TGM5 NEK7 INTS15 ZBED3-AS1 ZNF80 PDXDC2P TENM1 IL13RA2 ENSG00000293489 LINC01322 RORB AL136441.1 GABRB1 LINC02903 MYOF AP001116.1 LINC01754 AC142384.1 LTB4R2 ATG12P1 JAKMIP1 SH3KBP1 NALCN AC116903.2 SMOX AC020718.1 AVIL LPCAT2 ENSG00000286163 GDAP1 AMY2A AC023509.6 AC126755.4 ENSG00000287801 ENSG00000289870 ISY1-RAB43 LINC00398 EIF2AP4 TTC39C MIR3670-1 MYLK4 BEND7 DIO2-AS1 Z98043.1 TPRXL USP25 KIF2C AA06 MYO5A SIPA1L3 CTSLP1 LINC00393 SLC6A13 ZNF286A-TBC1D26 LRFN2 WDFY4 SPTBN5 AC068138.1 AC092862.1 SFRP4 EIF1B-AS1 BLTP1 SPDYE16 LINC01239 OR4Q2 PNRC1 IL1RAP ULK4P3 SEMA6A-AS1 AC015922.3 ENSG00000286662 AL772307.1 JCAD VAV2 TFRC DANT1 VPS13A TES OSBPL6 SH3TC1 GRIA3 PNPLA8 SPDYE6 AC087564.1 AC108474.1 FBN1 HAPSTR1 SERPINA7P1 RAB31 EXO5-DT ENSG00000291283 DIPK2B MAP3K1 ZFP82 DDX4 IQCB1 COA1 CLEC6A ZNF292 BMPER FUT10 AC109779.1 LINC00922 RNU6-984P CLASP2 ZNF521 RPRD2 ZNF761 PDXDC2P-NPIPB14P AC024230.1 ANGPT1 DUXAP10 VIPR2 SEMA6A ZNF567-DT PARAIL EIF4G1 TMPRSS15 AL133372.2 EEF1A1P11 B3GAT2 ATP6V0A4 ACSM5 PHLPP1 RBKS ZNF618 ITGA4 DNAJC6 CXXC4-AS1 SCIRT ROCK1 AL390860.1 NCAPH M1AP ENSG00000286072 TAX1BP1 AC008739.5 OR7A15P AL445430.2 TBC1D4 AC093843.1 APIP AC012456.1 MMP28 FAM13C ZNF701 LINC02971 NECTIN1-DT KCNT2 CRYBB2P1 NUP98 CLEC3A IKZF2 PML BX571673.1 FOXO3B AC012616.1 LAYN MGLL SCFD2 SCARA5 LONRF3 SLC5A12 PLEK2 TNFSF4 ENSG00000293021 DGKG AFAP1L2 SPATA25 LINC00923 SP100 ENSG00000287616 RNA5SP260 PLXDC2 ELAVL4 SBF2 CDH9 RF01880 AL731556.2 FOXRED2 AL359736.1 CFAP298-TCP10L NDUFAF2 LINC01524 UMODL1 AC022335.1 RFPL4B TTC28 TSEN2 GALK2 PCDH19 ENSG00000287783 LINC02060 NIN SPG21 ZNF286A GARS1-DT ST7-OT4 ANKFY1 ZNF69 CCDC144CP PRSS50 MIR4645 ENSA GMDS-DT ENSG00000286259 PPP4R1L MIR9-2HG FNDC3A KHDRBS3 SLC30A10 AIMP1 AP4S1 FOXG1-AS1 MYO5BP1 TPK1 HSPBP1 PRKD1 CALCRL POT1-AS1 CASC17 SLC20A2 USP34 AC091053.1 STK4 AC138305.1 PCDH10 AC023300.3 ENSG00000289174 TNNI3K AL646090.2 ENSG00000287184 DNAAF4-CCPG1 RAB6D TMEM131 SPC25 AC112493.1 ENSG00000287378 TMEM161B-DT ST6GAL2 RNASE11 AC018742.1 AC244205.1 SCAMP1 TXNL4AP1 PTCHD4 RPL27A LINC01002 ANPEP AC021979.1 PRLR LINC01821 ZNF569 GOLGA8O LINC01697 ARL2BPP10 ENSG00000289397 AP005436.1 ZNF180 AC008758.3 PDE11A NMI RAB6C PCCA CLVS1 YLPM1 IL16 LINC00972 ENSG00000291276 AC012485.1 DIPK1A LINC02814 SULT1C2P2 ZSWIM7 ZNF470 ENSG00000286932 AC107373.1 LINC02339 POTEH EDARADD LINC00558 LINC02511 CR2 OXNAD1 GNA14 ENSG00000286152 LPAR6 ZNF56P CTBP2 TTN LINC01721 NCALD RPS10P13 HHIP DYRK1A LOC105377209 PPFIA1 TMEM30A LINC01705 MYRIP ERICH2-DT LINC00343 PPP1R1C ZNF286B AL355838.1 KCNE2 UNC13C RGS10 LINC02882 CUL4A NDUFAF4P1 AC105052.3 SERINC1 LINC00265 KCTD16 CHSY3 EFCAB13 TXNRD3 TRAM2-AS1 AC026358.1 EYA4 ZNF888 AL391361.2 LINC01748 AC069287.3 AL163953.1 ARL15 SPATA13 LINC01288 ZNF736 AL031432.1 MAP7D3 SEMA5A ENSG00000288035 AL354861.2 LINC02762 ENSG00000290805 EIF3FP1 ELL2 SAMMSON LINC00886 LAMA4 PMEPA1 LINC01811 UBE3C ATE1 ENSG00000293330 ETNK1 AC110772.1 TTPA MYO5BP2 CYP4F30P PVT1 SLC2A1-DT GCNT1P3 CHAC2 GATAD2B FMNL2 METTL3 KRT85 UAP1 ZKSCAN7-AS1 PARD3 LINC00691 KCNN4 LINC01104 TRDC ZNF33A AL353132.1 CP FUNDC1 RNU6-849P COL6A6 BMAL2 ATP8B1 PGPEP1 AC026462.4 BZW1-AS1 RNF6 SEMA3C VAV1 SMYD1 PEPD ENSG00000289871 DPY19L1 GRM3-AS1 GRIA2 VDAC2 KIAA0825 AC002127.2 MIR3670-2 ENSG00000276197 VPS13B SPART AC018697.1 LOC102723684 IGLV4-3 LINC02315* |
| Mel Z Matrigel | 1683 | *SLC18A1 OR2AF1P KLHL13 ABCB7 AL355499.1 PBX4 ACOT12 LOC124903770 ENO1P2 OR11P1P MYO9B LINC00683 COL4A5 LINC01708 ERG ENSG00000293384 ENSG00000291325 PARN AL390816.1 STK16 XK SEMA4D MET MAX PDE4DIPP4 SCYL3 LOC105370954 EIF4G3* |

| | | |
|---|---|---|
| | | *ACSM3 AC231532.2 SNX16 AC116035.1 WWC3-AS1 LINC02476 LINC02487 LINC01915 SPAG11B IFTAP EOLA2 LINC00261 SUMO1P2 ZNG1F LINC01944 LINC02997 SIAH3 DUS3L CETN3 AC116424.1 RASA4B ARL14EP-DT GAS2L1 RPL4P1 AC108025.1 CHMP1B MMP16 OR7E25P ENSG00000286104 METTL25 AC091231.1 FAXC PRORP ZNF534 POLR2J4 LINC02334 DNAH11 SLC9A9 POLR2J POLG AC092957.1 EXOC2 DBIP2 AL358934.1 MYLK-AS1 TACR1 FSIP1 ENSG00000289332 AC068672.2 SNX25 SDC2 LINC02064 AC131571.1 ABHD12 AP002954.1 AC005999.1 COX10 CIITA FANCC AP006219.1 ARAP1 UQCRHL LINC00305 AC073488.11 HEPHL1 RN7SL354P ANKLE2 GACAT3 CEP85 RPRD1B CR381653.1 LINC01828 CIP2A LOC105371855 AHNAK ACTR5 PPP1R3B-DT YBX1P5 EIF4A3 DLEC1 COX10-DT AC004943.3 NFE2L2 AC069335.1 SCHIP1 AC004594.1 SLC46A2-AS1 TFEC NFYC H2BC18 BLOC1S5-TXNDC5 UPP2 CYP19A1 LOC105374367 UBTD1 AC073488.10 LINC00313 DTNB GPR158 VRK2 ITSN1 AC092552.1 ATP2B2 AC073488.2 ESPNL PREX2 SCRN1 AC007314.1 FAM177A1 ELAVL2 AC073488.5 TANC2 MAP9-AS1 PSKH2 ENSG00000286332 AC135507.1 NAV2-AS4 CSMD3 FBF1 NTN4 SERP1 AC090888.1 POLR1A PLEKHM3 ENKUR LOC105375146 CENPBD2P TPTE2 AC017002.6 RAB11FIP4 AC010307.2 CLMAT3 AC116353.4 C10orf53 HSFX4 NPIPB5 SNTA1 PPCDC RPL7AP83 ENSG00000289178 ENSG00000293110 LINC02578 CBX3P10 CCDC144A ASZ1 CU638689.3 ENSG00000289699 NUB1 ENSG00000293482 WIF1 MISFA FLI1 DYM-AS1 LINC02197 PABPC5-AS1 SPATS2L CLIC5 ESRRB ARHGAP26 LINC-PINT RBP7 TRHDE RAB44 PTPN4 AC105180.1 LEMD1-AS1 SLC16A11 ZBTB7C ITGA2 ENSG00000288643 AL442647.1 ENSG00000290070 KARS1P2 MIR5702 AC093459.1 AC026316.5 IL1RAPL1 AC091564.3 KIF5C BLK GPM6A SPATA16 AC068205.2 AC034195.1 WNT16 RERGL KRT86 SETD3 MRPS35P2 ACOX3 RPS6KA2 EFR3B PLPPR5 LINC01790 TRIM24 ZDHHC17 HLCS FIG4 AC106864.2 LINC01621 AP1S3 CFAP47 FOXP2 LOC102724452 RNU6-389P FP325331.1 ENSG00000288016 FBXO34 ENSG00000286069 KMT2D GUSBP5 LPAR3 C3orf49 CKMT1B SHE GIPC2 AC008825.1 GFOD1 ENSG00000288106 TXLNG SP110 SH3BP2 SH3TC2 CEP290 FAM171B CNTNAP3P2 AC239859.1 LRIG3-DT TACC1 SH3PXD2B OCLN AC092484.1 CFAP91 AC024598.1 FASTKD5 NETO1 EGFR PTPRQ TNPO1 GBE1 AC097634.4 SNX32 PCGEM1 DNPEP LOC100506321 AF130417.1 NEO1 GLT8D1 LINC02894 MYT1L STAG1 LINC02428 CLDN14 IQCJ-SCHIP1 AC004702.1 CARM1P1 GUCY1B2 LINC01968 PDE3A-AS1 LINC01829 PLEKHB2 TIMM23 CAPN11 UBOX5 TCERG1 LINC01608 AC024257.1 LINC00869 F10 SLC7A14-AS1 CKMT1A LINC01323 CFAP20DC-DT LINC00970 LINC00370 SCN1A-AS1 LINC03099 FAM184A AC073325.1 CABIN1 PSMD10 FBXO47 CCNO-DT FBXL17 EOLA1 SLC9A4 DHX29 STPG2 AP004833.1 GPAM MSRB3-AS1 IRF2 HEATR6 GET1P1 SPATA6L LRP12 TCF12-DT AC093766.1 LOC100287944 ENSG00000291120 AL353133.2 TBC1D22A LINC01492 ESRP1 HSFX3 NPIPB4 UBE2A BRMS1L RBM41 GOLGA6L1 AC099329.2 AC011477.4 RGS12 LNCOG AC245102.2 LINC02963 AC007529.2 CBX3 LINC02955 ACAP2 SLC39A10 PRKG1-AS1 AC020687.1 ENSG00000289788 ZNF496 ZNF550 CHCHD6 KIZ MPHOSPH9 LOC102724289 RNF10 MGC4859 ENSG00000287051 FAT1 SLC2A2 PIK3R1 AL078621.3 AC139143.1 IL17B LOC101928253 AGMO NOVA1 SARNP SHTN1 ATG12 AC100818.1 AC073488.4 MASP1 AC034114.2 TMEM232 CD200R1 LINC00587 BTD CALD1 CCDC144NL-AS1 SH3GL2 ZNF847P CLBA1 HIBADH ENSG00000288577 LINC02233 CRTC1 VCF1 N4BP2L1 PITPNC1 SLC30A3 AL137009.1 DOCK7 ENSG00000286353 AC106706.1 PDK3 PTCSC2 XKR4 FBXL4 ROR1 SMAD3-DT RAPGEF4-AS1 CCDC178 ADAMTSL1 PAX5 RTL8B AC090023.2 AAK1 LINC02755 CRB1 LINC02436 UTP15 AC116353.5 ARID1A THEMIS LOC124900945 SMAD6 RYBP NYAP2 PSD3 ABCA13 AL355922.4 AC012368.1 MEF2C FZR1 ST8SIA5 TBC1D19 DBNL PPP1R9A-AS1 AC005381.1 ELP4 RYK AP2B1P1 PXT1 DENND11 AC079466.2 TUBGCP3 ITIH5 WEE2 IQCK SLC15A5 MRPS10P2 AC110296.1 NDUFAF4P3 LINC02930 PTPRE MFSD6 SERINC3 AL035401.1 TBC1D31 ZNF347 PTPN12 PTPRZ1 LINC01749 AC105450.1 ACACA AC026786.2 PLN EML1 DDX39BP1 CTDSPL2 SLC66A3 SLC16A7 ALDH7A1P2 NEDD4 OCLNP1 NEK4 CACNA1E AC073488.9 ENSG00000293315 LOC105375297 AC024559.1 FLYWCH1 LRRC38 OVAAL AC078777.1 CYSLTR2 VWA8 PARVA AC106729.1 AC002064.1 SECISBP2 LINC02237 URI1 LOC127903862 MARK3P1 SLC9C1 SCML2 AL031847.2 SNAP25-AS1 CHD6 PDE4DIPP2 PRDX1 NDUFAF6 PACS2 AL121782.1 ATG4B AEBP2 SLC7A14 AL133255.1 DSC2 TRG-AS1 SERPINA1 GLB1L NPSR1-AS1 LOC100132172 LINC00376 OLFM1 RN7SL77P MID1 AC020897.1 POGK LINC00862 SLC12A2 OTX1 EFNB3 TRIM37 LOC101926964 ENSG00000286432 LINC02775 LINC02458 DLG3 LOC105376219 ENSG00000291284 PROSER1 FLOT2 PVRIG SRP68 SV2B LINC01879 ILK LEF1 EYA1 DAAM2-AS1 ZNF816-ZNF321P MAPK14 MYBPC3 WDR17 AC011499.1 ENSG00000290921 ZFHX4 RAPGEF1 ALG13 EFCAB14 AC068633.1 GPRC6A AC009139.2 UNC79 LOC100129616 SLC1A2 HOMER2P1 AC027228.2 EIF2S3B ARHGEF3 GABRB2 BTBD9-AS1 LOC101927293 AC010196.1 GRIN2B UBXN10 SVOP CRISPLD1 NOP16 IFNGR2 ROR1-AS1 DENND4A TBC1D20 AC083939.1 PLD5 OXR1 AC245748.2 LINC00700 SNX2 LOC729732 GRHPR AL929601.1 SCAMP5 IL17RB PARP2 MINDY3 ZSWIM6 MYL1 KLHL4 AC006041.1 TTC3-AS1 LINC03096 EIF3F RDH16 CFAP418-AS1 RNU6-1117P DNAJB6 F10-AS1 LINC02612 LINC02279 RPL23AP7 NPIPB3 ENSG00000289530 ENSG00000290967 LINC02683 ZNF724 PRR4 SPCS1 LEMD3 PLEKHF2 B3GLCT PIEZO1 OR2T11 GGT4P SPTLC1P2 ENSG00000287042 NEB AC103719.1 AL157359.2 AC090888.3 CSRNP3 AC087762.1 KANSL1L AL157944.1 MRGPRX1 AC018618.1 LINC00861 LINC02253 NUP210 PDIA5 LOC127814297 VDR C16orf74 AC097625.1 CCDC149 NEK11 CREB3L2 RTN4 ENSG00000286962 AC096887.1 GNL3L ARHGEF11 PRKAA2 CD163 SGPP2 DPT BBS2 CLSTN1 ZNF155 SERGEF SCYL2 AOPEP SKAP1 AC190387.1 CU634019.5 KRT18P35* |

| | | |
|---|---|---|
| | | *LINC02375 AC022816.1 CHM AC026415.1 SKIC3 MRPL3 SERPINE2 MYO3A ITGA2-AS1 ZNG1E LOC101928866 NUBPL PLAAT3 GGNBP1 IL12RB2 LINC01221 LINC00363 LOC100507336 PIK3CG ANOS1 TPP1 LOC100130691 STIL TRGV5 ATP6V1E2 NBPF10 AL935212.2 NUP160 FGF14-IT1 MCCD1P1 AC132803.1 AC137810.1 CDHR17P AC121757.1 IQGAP1 MYCBP2 WIPI1 MIR6841 LATS1 AC098850.3 NUP153-AS1 ZSCAN25 PHYHIPL ANKRD20A21P LINC01442 AC007881.2 LOC124903099 ENSG00000293441 KLHL12 ENSG00000286215 LOC349160 ENSG00000286097 SNX30 AC007092.1 LINC00942 FANCI THNSL1 OR9Q1 ENSG00000290589 ACAA1 CPEB2-DT ITGA9-AS1 PRSS23 CPHL1P LRRC37A3 ENSG00000287299 SPNS3 EXD3 TNFRSF10B IUR1 MAP2 RNU6-973P AC023078.5 GRM5P1 ENSG00000286982 NPIPB13 ENSG00000287771 FBXW11 MTCL1 HSPH1 AC008268.1 SUGP2 IYD AC108517.1 AC026992.1 SLC29A4P1 HNRNPA1P36 MIR646HG NHSL1 SMG6 HLA-DMA AC093802.1 CEACAM16-AS1 AC010343.3 TCERG1P2 LOC105370500 ENSG00000286041 PEMT ARHGAP6 ENSG00000289503 LINC01845 PPP2R2B CHD5 LINC02196 JAK1 GPD2 IL12A-AS1 ITGA9 KCNB1 RADIL LINC00298 TRIM39-RPP21 AC084200.1 AC105919.1 GNPAT HSD17B12 HS6ST3 GRM4 LOC100887080 AIM2 LINC01283 LINC00911 RF02271 LINC00578 TMEM132B MARK2P11 AL391095.1 AMT ENPP7P10 KITLG ARID4B SATL1 GMDS NDUFS5P5 PLEKHM2 NR1I2 ZNF266 THSD4 PENK-AS1 LINC01471 ENSG00000286371 LINC01414 AC073488.8 AC004852.2 AC068313.1 RFX7 RELN SRP14P2 ENSG00000286556 AF107885.2 LRRC37A ENSG00000286239 STAG3L4 PDHB ADAMTSL3 NXPH1 KMT2CP1 AC012467.2 CRKL LINC00871 TENT5A SYTL3 NDC1 AC009093.2 SULT1C4 LINC02742 LINC02074 SNAP91 ZNF816 AC104452.1 CDKAL1 RPS6KC1 CD40LG VRK1 AC100802.1 ENSG00000287950 PAPOLB URM1 LINC03041 GPAT3 ENSG00000287862 SCN9A ZNF420 DPYSL3 AC073488.3 C1orf146 SPRING1 AL807742.1 SLC16A10 PLCE1-AS2 ATRN NDUFB9 CCDC88A SNORA36C SLC35B4 ARNT2 UGT3A2 SPOCK1 RPS20P32 PITX1-AS1 HPSE2 PLCE1 AC007262.2 TNIP3 FAM106A TRMT2B-AS1 ENSG00000288799 TACC2 AC073488.6 AC009950.1 NT5DC1 AC099511.1 AC006927.5 FRAS1 AC022568.1 DCAF5 LINC01423 GNAL AP004607.6 CSTF1 STK32C MIR7110 AC090114.3 OR7A1P RNU6-10P MSH3 UBE2F-SCLY LINC02664 BAK1 MRPL45 AC034268.2 DIS3L2P1 LAX1 AC013644.1 LINC00705 PTPRG-AS1 STK26 AC002428.1 ENOX2 TENM4 AC068152.1 GOLGA6L25 TTC9-DT LOC102723446 RPS12P20 PPP3CA CHRNA5 CES2 AC133065.1 DUX4 MKNK1 DYSF NKD1 AL157762.1 ENSG00000286856 ENSG00000287849 RBPJ KMT2CP3 AL356807.1 RIN2 PDE6C AL137247.1 AC016629.1 PLEKHG7 DLX6-AS1 ASCC1 CDH10 DENND1A MZT1 LINC02068 ENSG00000290317 PDZRN3 ADAMTS16 TASP1 LINC00381 NBPF19 MICU1 AC008700.1 TAF10 ENSG00000287329 BACE2 KDM5A AC009154.1 ZNF549 AC010328.3 AP002761.2 LINC02026 LOC102724354 LOC101927855 NOMO1 AL133375.1 ARL6IP6 LINC01579 CNOT6 PKIB SPANXA2-OT1 LINC02284 TBX2-AS1 ASAH2 FRYL LINC01145 TBC1D9B PXMP2 ASH1L RSPO2 LINC02266 AC016598.2 AC010636.2 ZNF805 ACSF2 ENSG00000288632 OR5K1 OR13C9 PROX1 MAP3K7CL ZNF891 RALGPS1 CCDC6 PAQR5-DT PIEZO2 AC026474.1 AL133353.1 LINC00613 Z96074.1 VSIG1 CU633906.3 LRRC42 ITGB1 RBM26 AP003108.3 AC022031.2 RNA5SP29 CYB561 AC010632.3 DRC7 AL109837.2 UBA5P1 LINC00240 LINC02346 LINC01823 ENSG00000291175 RHCE CORO1C LHFPL6 SPIDR CCDST C17orf80 AP001180.5 NEFL TMEM185B STK3 CHN2 NBEAP3 FCAR FSIP2-AS1 C1GALT1 HNF4G SMCO4 MSR1 EXOSC3 MED15P7 GABRG3 ENSG00000290790 PSMD7-DT AL138752.2 LINC03095 DPY19L1P1 AC245060.5 SH2D3C APTX TRPC4 RAMP3 ZNF705A PCDH11X CPB1 LIMS1 LINC01456 ENPP2 DOCK4 ARPC1A LINC01900 LINC00382 ENSG00000286458 BCL11B CERS3 GLCE PLS3 AC062021.1 TSPAN2 PCSK2 KCNIP1 PWWP2A LOC105373170 SLC29A4P2 TBC1D10A ENSG00000287188 REEP1 RPH3A CTTNBP2 LINC01088 NME9 CYTH4 NAP1L1 SLC38A6 AC245297.3 AL121594.1 HDAC2-AS2 VPS29 PLPP4 CCDC102B LINC01985 AC132219.1 COPB2 UBR1 AC005972.3 CNN2 COL4A2 SLC1A6 KMT2CP2 DYRK3 IP6K2 AC114501.2 AC069444.2 LNC-LBCS CHRNA6 AP000688.3 AP002765.1 LINC02929 AC002428.2 ENSG00000287478 TLN2 HDAC4 F11-AS1 LINC01276 FYB1 RIOK3P1 AL935212.1 RBM45 SEM1 AC073488.1 GYS2 IQCE LVRN RPGRIP1L MYLK UNC5C CFAP69 MINDY4 CSMD2 TLR7 FLG2 P3H2 AC064859.1 CFL1 LARS2-AS1 PPIAP76 ENSG00000288067 LINC01793 DTYMK ENSG00000290821 WDR7 DIAPH2-AS1 SLAIN2 HMG20A CEP85L TUSC3 COPG2 PLEKHG4B ZFHX2-AS1 RBBP8 AC090517.4 MED13 RNASEH2B PHF2 TAAR2 RAI2 AFDN DEPDC1-AS1 LRP2 CYTIP MTDH ENSG00000290606 ENSG00000287918 ENSG00000288804 GOLGA6L6 LIG1 ZDHHC13 WWC3 LINC02715 SERPINA9 GALNT14 CLPX AC009084.1 VWC2 NCAL1 LINC01170 REPS1 FBXL7 RPA3 TOMM40P2 RNU6-1229P TMEM108 RPL29P26 CC2D2B CLUHP10 CHMP1B-AS1 DOCK7-DT MB21D2 SEZ6L AC005828.4 SLC39A8 AFG2B MANCR AC009242.1 LINC00412 MAP4K3-DT UPRT TMEM59L ADAM17 LINC00968 ENSG00000286185 FAM78B SEMA4F AL133353.2 ELAVL1 LIPJ ZFHX3-AS1 AC004922.1 TRAK1 STXBP1 OR6N1 ENSG00000287923 AC008696.2 IDH1 AL109914.1 LMOD2 AC092329.1 LINC00424 ENSG00000289846 LOC112268173 MIR4713HG TSNAX-DISC1 LINC01958 HDGFL2 MUC13 NUP205 NHLH1 AP005203.1 ELP2 SLCO5A1 RPSAP37 AC021443.1 SNTB1 ORC4 LINC01723 AC135586.2 AC022031.1 AC021231.1 CYP8B1 ENSG00000286500 ZNF350-AS1 APC PEX5L-AS1 LINC00540 AC090371.2 TTLL5 INO80 AL513325.1 NAA11 LOC728554 MOCS2 ERGIC2 PEAR1 CDA LINC01591 BANCR PPARGC1B STON1-GTF2A1L CTRB1 AC093523.1 AC239727.1 AC005225.3 AC124254.1 AL359237.1 AC091551.1 AC108081.1 NOL10 AC092650.1 AC068759.1 LNX1 AC087386.1 LOC105374191 ENSG00000287694 ERC1 SMIM13 AF228730.5 MIR8058 PARM1-AS1 ZNF440 RUBCNL AGO3 UBOX5-AS1 C9 CYYR1-AS1 PVR CASTOR3P CKAP4* |

| | | |
|---|---|---|
| | | *RPL39L ACER1 IQGAP2 CDYL RLF AC087289.4 ENSG00000290507 AC092645.1 TNPO1-DT ANKRA2 C12orf42 TAF5L AP000265.1 JPT1 CTNND2 CU633904.2 DNAH14 LARRPM AC010197.2 DELEC1 UGGT1 PACSIN2 RPL36AP47 MIR181A1HG DPYSL2 NEK2P4 ENSG00000288700 ENSG00000287169 NUP210L AC008574.1 CNTNAP3C AL049775.2 GIMAP8 RAB8B METTL8 NSD2 AL023574.1 ZNF516-DT GRM8 5_8S_rRNA PHACTR3 AL139352.1 AC107023.1 EIF3J-DT AC005261.3 ENSG00000291209 AARS1 KCND1 ENSG00000286499 TMEM267 JADE3 CARD18 PHF3 PSMC2 ENSG00000287831 MAPKAP1 LINC01853 EXOC6B LINC01950 EVC2 GON4L LINC03017 ENSG00000293214 AC007179.2 AC007780.1 ENSG00000286812 DNAH6 MGAT5 IGHV3-42 ACOX1 USP24 HAO1 NUP153 FBXO38-DT LINC01581 HSD17B4 EFHD1 LCN12 AL500522.1 TRMT10B TRAV32 COL21A1 UFL1-AS1 RAI14 ENSG00000292277 LOC285626 GPATCH2L PIGR NRP2 GTF3A SIPA1L2 CAVIN1 MIR548AD GYPE MSL3-DT C1orf21 METTL15 TMEM165 AC016745.2 LINC01229 FOXJ2 RNU4-69P CEP162 HECTD4 ELOVL7 AC092111.3 NR3C1 DLX6 KMT2CP5 AC011466.2 BORCS5 FSTL4 ZC3HAV1 MTOR XACT LINC01797 ENSG00000287907 TRAV5 NBPF20 KSR1 FAF1 LINC02217 SNAP25 AC004691.1 SGSM1 DEFB124 ARL10 MFSD4B-DT CHST4 CCDC144BP AC005050.1 OR11H6 ENSG00000287258 AC083795.2 ENSG00000290565 GOLGA8A CLDND1 CR381670.1 PIP5K1A BTBD2 GH2 MMACHC AC131025.1 AC008539.1 HS6ST2 RAB6B KYNU AC015468.3 LOC105370906 ADAL TBXAS1 ASCC2 AC046136.1 SPRED1 ENSG00000289744 GALC GNG4 NUDT19P4 CDH26 ENSG00000293242 RNF32-DT SNX19P4 PDS5B CRIM1 ENSG00000289487 MEI4 NALF2 RBM46 ABCB5 RNF115 TRIM61 ESD CYYR1 CDHR3 LINC00689 PER2 LINC01076 ANKRD24 IPO5P1 LHPP KAT6A BCOR KCNK12 NTRK3 LINC02932 RXFP1 LINC01493 LINC01807 TADA1 DIS3L2 ENSG00000290702 ENSG00000287291 SNTB2 ENSG00000286954 AC073263.1 RSBN1L PDE1A PDCD10 LOC107985643 PRAMEF11 LMTK2 AC023055.1 AL133268.3 HHAT LINC00632 PSMA3P1 FAM227B CCDC68 COPG2IT1 GOLGA7 AL357153.3 FERMT2 AC245519.1 GCNA GPR82 AC068587.3 AC130710.1 TSPOAP1 AC008056.2 GLUD1 AC016885.1 DNM3 ST3GAL2 SREK1IP1 PHIP DENND5A LINC00309 SS18 LOC124900792 SFRP5 RPL23AP38 ADAM6 GOLGA6L24 AGBL3 ZYG11B AP000311.1 CD244 LINC01643 LINC01933 RN7SKP173 SHISA6 IGLV3-1 NFASC AC122134.1 OR2A1-AS1 HDX RAI1 CPEB2 AC104339.1 LINC00513 ZNF229 GORAB-AS1 NXPE4 PHF6 HMGB4 ENSG00000288075 ENSG00000287533 FGF7 TENT5D MARCHF10 TBCA STXBP6 MGA DENND2B-AS1 AL137782.1 AC021660.2 FOXP4-AS1 RTTN CACNA1A AL596087.2 RN7SKP284 LOC100533679 LIMCH1 TNFSF13B Z93403.1 AC006288.1 ENSG00000291170 MTMR7 PTH2R AC005909.1 LOC100420587 IAH1 AC073488.7 COL14A1 RNU2-38P AC005828.3 EIF2A ENSG00000286209 JARID2 CYP1B1-AS1 CYTOR HDAC2 FIRRM TPO TGOLN2 FGD1 AC008415.1 INSYN2B TRIM39 TRRAP AC009090.6 AC105213.1 ENSG00000286134 LINC01422 NPSR1 GLIDR EIF4E3 USP48 KCNH5 RRM1 MAGEA1 CADM1 INPP5D KLHL29 CFAP161 THUMPD1 AL356309.1 CLYBL OR4C6 RN7SL797P ATF6 PRKAR1B GBF1 IPO11 LINC02805 CCDC88C SERPINE1 ABHD15-AS1 BACH1 CUL9 ENSG00000289228 PRAMEF4 EWSR1 INMT-MINDY4 TM7SF3 SLC26A5 AKAP13 AC091047.1 PKM GPAT4-AS1 GCG MRPL45P2 ATP12A CCDC150 LINC01735 TKFC ENSG00000289293 LINC02218 SMARCAD1-DT PTPN3 ENSG00000292979 SEPHS1P1 STAC AC010547.2 AF241728.1 RTL4 NNT SSX2IP ST8SIA2 TTC17 ELP1 IRF8 HIVEP3 AC074386.1 PHF5AP1 AC005999.2 AC018629.1 AL008638.3 HEPACAM2 KAT7P1 AC023824.1 RFX4 CCDC198 OR6K6 ZNF30 REM1 LINC01482 FREM1 AC091564.7 NDUFA10 CBR1-AS1 CD69 FUNDC2 DBR1 EPS15L1 CYB561A3 AC021660.3 ENSG00000291188 XIRP2 Z99571.1 AC079950.1 LINC01248 KDM3A AL356272.1 ENSG00000293483 MZT2A CAST AC005394.1 KBTBD11-OT1 GPR39 ADGRA3 CASK SLCO4C1 RPS29P9 GCNT1 BPIFB3 AF121898.1 SLC35F4 GGA2 AC008277.1 USH1C AC118758.3 LOC101927468 LINC02307 AC136428.4 NLRP14 DNAJC13 LOC345471 PSME3IP1 LINC01075 TRDV3 LINC01478 ENSG00000287469* |
| 163 | 34 | *BRCA1 FBXL20 PPIEL FOXN3 KC6 SRGAP1 SYNE1 RUNX2 NBPF14 KTN1-AS1 RBMS2 EDNRB SETD5 SMPX LINC00342 ENSG00000286248 GTF2IRD1 HIP1 FBXO32 PTPN13 APBA1 ZNF320 CSGALNACT1 GLI2 TGFBR3 S100PBP PHLDB2 MOB3B COL15A1 CBLB PLEKHH2 MAP2K5 CNTRL GNAO1-DT* |

**Table S8.** Venn diagrams show the intersections of rDNA-contacting genes possessing ≥ 30 contacts with nucleoli in Mel Z cells grown either on plastic or on Matrigel with 188 downregulated genes. Related to Figure 6B.

| Names | total | elements |
|---|---|---|
| 188 Mel Z Matrigel Mel Z plastic | 13 | *MED13L NFIA KCNQ3 DNAAF9 HMCN1 OFCC1 Y_RNA EGFEM1P DLC1 KIF16B CDK6 RAPGEF4 NPAS3* |
| Mel Z Matrigel Mel | 645 | *AC092634.2 PAX7 RALYL LINC01201 FP236241.1 ABCC6 PTPRR DLEU7 EPB41L4B ENSG00000286540 AC012409.2 LRMDA EPN2 PIR SUGCT DIP2A PRH1-PRR4 LINC03007 TRPM6 SLC8A1 RNF103-CHMP3 AL356108.1 ANO5 PCDHA13 ZNF385B LOC102724843 PREP* |

| | | |
|---|---|---|
| Z plastic | | *AF127577.4 RSRC1 GRIK2 DPYD OTX2-AS1 SLC22A14 LINC03000 KDM4B FGF1 AC090809.1 ZBTB20 ARHGAP15 LOC101929710 LINC00486 LOC107984685 AHR AC022523.1 FSTL5 TTC3 CADPS2 TLK1 ASTN2 FAM9B LRP1B KALRN AC009262.1 MKLN1 USH2A SUMF1 NEGR1 XXYLT1 FGF12 BTBD9 PARP4 FRG1-DT PCDHA12 AC069228.1 MAST4 NRXN1 AC079801.1 AGBL1 MAGI1 GNGT1 AC036214.3 FARS2 ST6GALNAC3 DPP6 AC098588.1 FAM13A RNF217-AS1 DAAM2 STK24 AL365295.1 LMO7 GRM3 U8 EXOC4 LINC02822 AC078828.1 DNAH7 BBS9 ENSG00000290711 NTRK2 ANXA10 ANKRD34C-AS1 CHD7 ARL17B NBEA AL512598.2 RASGEF1B TMX3 LINC00273 MIR99AHG ENSG00000293415 NRG3 PTPRG CALCR ELMO1 ENSG00000287881 NCOA1 IRAG1 LINC01684 AL390957.1 ATP2B4 ROCR TOX2 PHEX SOX6 LINC03104 PDE4DIP TRPM3 FBLN7 TMEM64 DNAH8 CNTNAP5 PRANCR RAPGEF5 FGF13 DRAIC PATJ LINC01798 GPC3 AL512380.2 DMD ADCY2 DYNLRB2-AS1 LINC02566 CDH19 LINC02966 ADAMTS9-AS2 GNG12-AS1 ATRNL1 CHRM3 ARHGAP24 LINC00907 SLIT2 TPTE2P6 ZNF804A FAM20C ENSG00000291181 AC010235.1 TTC33 LINC00347 AC098588.2 NALF1 KAZN BRAF BABAM2 ENSG00000289084 KMT2C FKBP9P1 CU638689.2 LINC01320 STEAP2-AS1 ASMER1 B3GNT5 ERBB4 KIF26B GPHN ENSG00000293389 MEF2C-AS1 ATXN7L1 ZNF385D RAD51B DOP1B SH2D1A MYO3B ENSG00000287722 SH3RF3 EXT2 DIAPH2 AL138720.1 MAP3K5 STAG2 SPAG16 DSCAML1 HTR2C GALNT13 LOC101927609 CNTN4 TBC1D5 RAP1GAP2 LINC01830 LOC105369165 DMBT1L1 AC013652.1 CCSER1 PIK3C3 ENSG00000286033 LINC01695 LOC105373436 ITGA1 TCF12 SGIP1 ARMC8 ENSG00000289694 RUNX1 MYO16 ESRRG AC058822.1 AL161757.2 PCDHA2 FRG1HP PCDHA11 PCLO PRH1 SLC38A4-AS1 ENSG00000290357 CDH18 FRMD6 ANK2 LOC101927314 MIR100HG ANO2 PCDHA9 SUPT3H ROBO1 LINC01091 ENSG00000289002 NEK1 LINC01924 DGKB PWRN1 CDC42 EBF1 OBI1-AS1 PRRC2C PCDHAC2 CDH6 AC109830.1 ENSG00000290397 DOK6 WDR64 C8orf34 JAZF1 BNIP3P41 CHODL ZNF267 LINC01692 CPED1 FGF14 MIR325HG CACNA2D3 LINC03116 SLC8A1-AS1 PCDHA10 MUC19 EDIL3 LRRTM4 PTPRN2 INPP4B SOX5 DSCAM DGKI LINC01151 COL28A1 DENND1B LARGE1 COLEC10 SDK1 PUS7 CCDC33 LINC02694 EPHA6 LINC02328 IGF1R SPTB WDR72 NLGN1 TMEM260 IQSEC1 ENSG00000287916 ANK1 PPM1L CASZ1 CNTNAP2 TRIM71 ENSG00000286523 KCNIP4 SOX2-OT CFTR DYNC1I1 NOS1AP OTOGL DOP1A AC027031.1 PCDH9 NKAIN2 PKN2-AS1 RBMS3 AL603840.1 FP700111.1 SGO1-AS1 CFAP20DC CIBAR1-DT ATP6V0D2 PCDHA1 ZNF800 PCDHA8 CU638689.4 ENSG00000290551 TBCK AC108010.1 CDH12 SHC4 LINC02112 AC098650.1 FRMD6-AS2 TESHL CBR3-AS1 RPS3AP27 DDR2 ADAM22 LARS2 HULC GPC6 MCF2L2 TRPS1 ENSG00000293394 CDH4 ENSG00000288187 DPP10 CELF4 ST8SIA6 CNTN6 PARM1 AC005670.2 CU633906.2 FBLN5 SPEF2 ENSG00000286745 WDSUB1 ZNF638 ATP9B MITF CACNA2D1 NSMCE2 PCSK1 MIR4435-2HG PCDHAC1 HERC2 LRFN5 UTRN GPC5 ENSG00000290523 AGBL4 OGDH GHR ULK4 RBFOX1 PCDH7 LRRC4C AC084149.1 PPP1R9A TMEM117 AP001341.1 RNA5-8SN5 SMAD9 NKAIN3 DYNC1H1 CRPPA NEBL AC006148.1 NAALADL2 AC007161.3 OSBPL10 LANCL2 ZFPM2 MRPS28 SLC35F1 TRD-AS1 LINC02306 FRMPD4 MEIS1 GALNT17 EDAR PMS2P4 CFAP20DC-AS1 BRINP3 RIMBP2 FHIT AC009093.10 WIPI2 DNMT3A TOX PCDH15 SGCZ ACE2 CHMP3 KCNAB1 PDE4D CNTN5 AL121718.1 ITGB8 PDE3A RIMS1 TRANK1 WWOX PCDHA7 NEK10 BEND5 ANAPC10 LSAMP MIPEP LINC01681 PWRN4 AC007100.1 DIAPH3 LINC01440 TMEM178B CPLANE1 NOL4 PCDHA5 PSG11-AS1 PLCB4 RNF216 FTO UGGT2 TNFSF11 LINC02326 PPFIA2 ENTPD1-AS1 ENOX1 WNT2 ENSG00000292991 OR8B8 STEAP1B UMAD1 LINC00299 POU6F2 SFI1 MGAT4C DIRC3 ANKRD44 CALN1 UXS1 LINC01584 NTNG1 ZNF804B SGCD GRM7 AC007848.1 STS NAV3 AC091489.1 AC092343.1 AL110292.1 AC079943.2 LOC124903324 PTPRM ENSG00000286225 PRKCH NRXN3 DENND2B LINC01500 MIR663AHG ATP8A2 CU633906.4 CERS6 AL591463.1 LPIN1 PLEKHA5 MACROD2 AP005328.1 ENSG00000287635 ADAM7-AS1 AUTS2 AC092042.3 LOC102724701 SYT16 BCAS3 ADARB2 ENSG00000288723 COMT PCDHA6 MCTP1 ENSG00000286110 NBAS ZPLD1 KMT2E LINC03060 SYNE2 DOCK2 SDCCAG8 SSH2 AL035078.4 LMNTD1 CDH13 MDGA2 AC103796.1 BDNF-AS DANT2 LINC01934 FBN2 DAB1 TTLL6 RFTN1 PCDHA4 SNTG1 ALK CACHD1 SEMA3A AC068051.1 YEATS2 CCDC26 SLC8A3 ENSG00000290983 MYO5B CADM2 MALRD1 TENT4B MAD2L1-DT AC010601.1 DCLK1 AC004917.1 FAM135B CDH2 TENM2 VPS41 LINC00879 TEX13D HDAC9 KIAA1549L USP9X KCNB2 USP13 NBPF1 ADGRL4 AC090376.1 LYST CADPS DDX60 LINC03105 RGS7 ENSG00000293037 LINC01470 SRGAP2 TFAP2D AC067956.1 PPP2R2C HEATR4 SPECC1 PTPRT GABPB1 CSMD1 ENSG00000286746 SCEL CTNNA3 FREM2 CLTC THSD7B ENSG00000287404 DACH2 AC093515.1 SORCS1 CAMKMT RNF220 ARHGEF37 DLG2 SACS AFF2 PTPRD RORA AL355499.2 PLCB1 NPEPPS PRKG1 BRIP1 MIR924HG LOC107986400 MAGI2 NELL1 MIR4300HG CYP4F62P PLCL1 LINC02215 GALNTL6 PXDNL SCN2A ASIC2 DOCK10 MICAL3 ADK TMEM164 KMT2CP4 RANBP17 GTDC1 RYR2 SLX4IP LDLRAD3 ENSG00000286648 CU633904.3 PTPRA AC011287.1 FAT3 MSH2 PCDHA3 MGAT4A AC078845.1 KLHDC10 RGS9 AF241726.2 MPDZ AC016766.1 EYS SLF1 ROBO2 CR392039.3 ENSG00000288891 LINC02055 SLC39A11 LINC00378 AC026167.1 AC015687.1 PAX3 LOC105379109 KSR2 DLGAP2 BMPR1B PDE8B DCC CTNNA2 PEX5L ARL17A DNER ETV6 CEP112 NRG1 PGM3 DLEU1 SLC25A48 ENSG00000293385 AC092167.1 DNAH5 SATB1-AS1 FANCB LINC02208 NRXN1-DT* |
| 188 Mel Z | 122 | *APBB2 CDK14 PDE1C ARHGEF26-AS1 MAOA THRB MAPK10 CACNB1 KCNQ5 PCAT1 GRIP1 DLGAP1 ARID1B CACNA1C CNN3-DT LINC02405 INTS6-AS1 CEP192 FRG1JP NUDT13* |

| | | |
|---|---|---|
| plastic | | *COL24A1 C2orf88 COL12A1 AFF3 LPP EPB41L4A CTIF ATP4A PRKG2 RSKR ALDH1A2 MIR3681HG UBR5 CHL1 ARHGEF39 PAK3 KLF12 CASC2 CPEB1-AS1 GDPD1 ZNF429 ADGRD1 EPHA3 CACNB4 ADGRB3 KLF7 ZNF280B KIAA1614 ZFHX3 LHFPL3 RASGRF2 DAPK1 PPIL6 LINC02610 PELI2 HCN1 TLR1 CECR2 APRG1 PLD1 RARB GNAO1 ASAP2 NEDD4L WNT5A AGAP4 ESR1 L3MBTL4 FIGN BTBD8 BNIPL SLC44A3-AS1 COPB2-DT BBX EFNA5 CASC15 AKAP6 RAB3GAP2 SMIM2-AS1 SHROOM4 ZSCAN23 ZNF615 NCOA2 ACACB RGS17 ZNF860 TMTC1 ETV1 ZCWPW2 TMTC2 LDLRAD4 ZNF665 TLR6 PPARG TANC1 LINC01322 TPRXL LINC00393 EIF1B-AS1 IL1RAP SEMA6A-AS1 ZNF521 PDXDC2P-NPIPB14P ANGPT1 PHLPP1 IKZF2 AFAP1L2 SPATA25 SBF2 TTC28 GMDS-DT PRKD1 TMEM161B-DT LINC01002 PDE11A TTN LINC02882 EFCAB13 SAMMSON FMNL2 ATP8B1 VPS13B* |
| Mel Z plastic | 1997 | *AL157886.1 FSTL1 CDH12P2 CASC20 C10orf90 AC243829.5 PFKP MIR3171HG AC012501.1 ESYT1 STYK1 RPL19 HS3ST3A1 RTN1 RNU7-188P AC100775.1 RGN TRGC2 ZSWIM1 MSRB3 ENSG00000293331 MREG HNRNPCL4 NOSTRIN ING3 ENSG00000290149 MAP3K3 NINL WSCD1 ENSG00000287515 OSBPL8 SLC12A8 LRRN1 HERC2P2 ENSG00000286309 ENSG00000291144 LINC00698 POTED STX8 AC032019.1 PHF20 FOXK2 CPNE4 TXNDC8 RBM17 AC021517.1 SMYD3 GARS1 RAPGEF6 ARHGEF9 TAFA2 ABR KRT8P32 TLR8-AS1 ISM1 BLTP3B LINC01163 AC012355.1 LINC02438 HYDIN2 ENSG00000287308 HCCAT5 TRAPPC9 PCDHB1-AS1 SLC16A5 LINC01545 FNDC1 TMEM255A MAP3K13 EIF1AX PPM1A ARL13B RPL15P21 SAMSN1 RN7SL556P C12orf75 AC010291.1 THADA LIMD1 IMMP1L TF SNX10-AS1 CHST11 MYBPC1 USP42 IPP RCSD1 SPTBN1 UBTFL3 AF228730.2 CDC42EP3 ENSG00000286111 AC013472.2 AC021269.2 MIR3117 MYO5C LINC00841 ITPRID1 USP32 AHCYL2 DAPP1 ATF7 CD101-AS1 GNG12 CHRNB2 CDC14C SEZ6 CLDN6 LINC01967 CDH8 GNB4 AGTPBP1 AL356490.1 ATF7-NPFF FHL1 TRMT6 PPP1R37 RBPMS ATP10A LINC02457 SH3PXD2A SPINK5 MRPS24 OPA3 SKAP2 PRND PIGL ATP7B PPM1H GPBP1L1 RPS6P19 KCNJ6 RPL21P82 BRD4 ZAN DOK4 AC007405.2 LINC00517 AC092378.1 TNRC18 MIR9-3HG LOC102724019 LINC02828 CAMK2B CASC19 ELMO1-AS1 ZNF962P OR4N3BP ARHGAP11A-SCG5 LOC124901321 URGCP-MRPS24 DDI2 AC008764.4 GPATCH8 PGM1 DCT AC073071.1 FAM193A LNCATV PECR LRP5L SLC38A9 LINC01982 CDRT15P9 LINC00348 ZNF264 QRSL1P2 LINC02133 HBP1 SDR16C5 RGPD2 ABCG8 XPO4 OTUD7A AC060234.3 AL583785.1 AC107909.2 OPN3 MAP4K3 AC092078.2 FAM157A LINC01194 GALNT9 FPGT-TNNI3K SNORD114-10 LOC442028 NOTCH2 CD93 LOC105379362 FKBP5 TMOD2 SLC44A5 SDK2 HMBOX1 SIM1-AS1 AP003900.1 ZNF551 LINC00882 RNU2-49P NPNT AC019211.1 DSC1 EPB41L3 MAP6 AC012363.2 PARVB MORC1 CDH11 RN7SL266P SHANK3 WDR26 NR4A3 CYP2U1-AS1 SPTLC3 GAS1RR AGAP12P LINC00494 AC005154.4 LOC101927948 DPY19L2P2 LOC105375387 ENSG00000289752 AMYP1 E2F7 ACVR2B-AS1 L3MBTL4-AS1 SLC14A2 ENSG00000291166 FAM169BP AC091304.2 FOXO1 LINC02311 ZNF611 STYXL1 AC244517.9 LINC02156 STK39 PDE11A-AS1 SMURF2 SRL ADAMTS12 FER1L6 TBC1D16 PLXNA4 AL592078.1 LINC02542 LINC00504 LOC124900810 CDK18 URB2 YBX1 ARMT1 DIPK2A CFAP70 DCDC1 FAM20B ENSG00000287410 AC046195.1 FAM3C SNORA5B ASB4 IDO1 AC026786.1 AC015922.1 ARB2A LOC105370489 PRKN ENSG00000286937 NLGN4X RMND5A LINC02044 HSD17B2-AS1 PRDX3P3 PNPLA3 LINC00320 AC092447.10 F13A1 ENSG00000288041 TAFA1 PIK3C2B XPO6 AC103876.1 KDM7A SLC44A3 SLC5A7 GNA14-AS1 MARCHF9 FAM83B IFT172 AL136317.2 RNU6-258P GPSM1 AL121900.2 CBLL2 AL136372.2 PTBP3 ZBTB44-DT FAM117A CDON SERPINI1 SLC17A6 NLK SDF4 THSD7A OVCH1-AS1 GNAI1 SPATA31D2P MRPL13 AC138932.2 MAPKAPK3 NCAM2 CHML PAPOLG H3P38 TIAM2 CFAP54 ZNF10 RORA-AS1 AC015804.1 TRAK2 AL139383.1 ENSG00000293038 CNMD ENSG00000286229 HERC2P3 LRP8 TAGLN3 AC018767.3 LINC00992 CNTN1 AC002451.2 AGL MEOX2 MAPRE3-AS1 STAT5B ENPEP LINC01242 NUDT19 ARHGEF4 LOC102724428 UBE2O MUC17 DDIAS AC025884.1 MTUS1-DT DUXAP9 AC092100.1 CPEB4 EIF4ENIF1 HEATR9 LOC124900848 CHRFAM7A ERBIN RN7SL430P CR383658.2 ZNF121 HERC2P9 MBTPS2 RGS22 PLS3-AS1 LINC00598 CD247 ENSG00000290948 ENSG00000287621 MCC TRIM52-AS1 PTH GEMIN8P1 ENSG00000287008 ADAR DNAH10 PDE10A TM9SF2 ENSG00000286250 PDHX CLCN3 RHOJ CDC37 FGF10 ZFYVE16 JAZF1-AS1 MECOM AC009878.1 RSPH10B COL4A6 CATSPERB ENSG00000287474 TSPAN18 SAMD15 NLRP8 ENSG00000286800 STK32B PLG NEURL4 POLR2J3 HERC5 LINC02505 LOC124900957 AC008109.1 CDH7 LOC101929457 ENSG00000290585 PGPEP1L UBE2E2 ILDR2 SCEL-AS1 AC034229.1 MGC27382 NCK1 PUDP ENSG00000287744 CD84 FAM168A NONO RARRES1 AC138512.1 KLHL42 NREP AF064860.1 EXO1 CCDC71L GABRA5 AL591684.2 ARHGAP23P1 EBNA1BP2 SNORD114-9 NEXMIF ENSG00000286274 LOC401913 ENSG00000288755 MTCP1 ASTN1 SH3BGR MROH6 TRAJ17 AC104248.1 AC004492.1 AC231532.1 KDM2B PREX1 HEY2-AS1 RGPD3 AL133500.1 PRIM2BP AIG1 ERICH1 IGLV1-41 ZNF404 PMS1 CDIN1 WRNIP1 ABCC13 TYW1 ANO3 GOLGA8R AC009511.1 COL11A1 AC119677.1 AC112721.2 AC113391.2 RSF1 AC022509.1 NCOA7 KCTD3 AC063949.2 RNU2-33P SERTM2 SEPTIN10 CIDEB MARCHF1 OR4N3P ENSG00000286376 ENSG00000286922 SLC45A1 AL773545.1 MCPH1 ENSG00000289143 MYO1D ENSG00000287045 SESTD1 ENSG00000291338 LINC01661 ENSG00000288553 ZNF214 COL1A2 DTNA TMOD3 ENSG00000286020 LOC340512 GEMIN5 EEF2KMT MRTFB ENSG00000286147 XPO7 KCNS3 AC011447.3 ENSG00000288902 VWA3B RPL34P11 FREM2-AS1 ENSG00000287608 ENSG00000290114 ABLIM1 ENSG00000287763 AC009226.1 RBMS1 ALPK2 CHRNA7 HECW1 HMGB1 ZNF304 DYNLT2B AC021733.1 CDIP1 RFLNA AL158198.1 AC062039.1 HIVEP1 ATRX TNIK STK24-AS1 UBTFL5* |

| | | |
|---|---|---|
| | | *LOC105371956 TGFB2 MDFIC LAMA2 ADD3 TRIO FBXO16 ZNF85 LINC00269 ENSG00000291054 LINC00366 HOXC13 AL049875.1 SMARCC1 NDST1 LINC00355 MUSK KRT223P ZNF73P KCNJ18 JAM3 ENSG00000287741 KCNIP3 SFPQ AC027338.2 PNPLA4 AC004485.1 ALKBH1 SNORA5A ARPP21 AF130359.1 RGS3 ZNF299P DUXAP8 NUP93 LDHAL6DP AC108673.3 ZCCHC4 MRPS9-AS2 ZNF407-AS1 RERG G2E3-AS1 ESRRAP2 OR5AU1 PRKAR2A FILNC1 RBM33 AL604028.1 CFAP57 ENSG00000286902 FADS2B CALCRL-AS1 ATP11A AC023389.1 MRPL33 RBM23 AC008695.1 ZNF154 CSTF3 SLC15A4 HIGD1AP9 ENSG00000288087 AL024474.2 AC244517.11 CERS6-AS1 CDH12P4 PCDHB8 GOLPH3 AC015908.7 MBTPS1 NCF2 PIGV RNU6-1216P USP10 SND1 EPIC1 ENSG00000288692 RN7SKP141 IGFBP7 EPHA5 MACROD2-IT1 RABGAP1L-DT LINC03076 PTPN20 ENSG00000290849 AC087501.3 LRRK2-DT SKOR2 AC130650.1 CBR4 CRLF3P2 ATP13A4 AL162718.1 AC009055.2 ENAH SNX10 ATP2C1 TMEM230 AL354718.2 PPTC7 STON2 PTGFR IGHV1OR21-1 AL138895.2 FAM171A1 STRADA LINC01725 HUWE1 ZNF595 ASPH RRAS2 SH3BGRL ZDHHC4 AP000894.1 AC114781.3 AC096558.1 GTF2F2 AC105031.2 SIM1 KIAA1217 ABTB2 SOX30 AC244131.2 TMEM132C ANKS1B TRAJ16 SPOCK3 GSN PLS1 AC007991.3 TCP11L1 SPON1 MAPRE3 MICB-DT TLR8 HPS3 AL391361.1 AC011447.2 KIF21A LOC124900600 SLIT3 ARMH3 FMO8P CACUL1 NLRP7 CD226 PHC2 MITA1 AC142086.6 ZNF578 ENSG00000290721 ENSG00000287021 ANKRD11 ITPR1 AL162254.1 PKP4 AL160286.3 CPM AC105052.4 DNAAF11 ENSG00000288069 LINC01033 NDUFAF7 AC007846.2 ABI3BP RNU1-83P LOC124900504 AXDND1 AC011824.3 IPMK LINC03042 AC073575.2 LINC01376 CYP2A13 RPL23AP51 TMEM183A RERG-AS1 AC126763.1 ENSG00000289956 CAMK1G PRUNE2 SERPINB9P1 LINC01122 RNA5SP96 ENSG00000288694 AC073130.2 PRKAG2 LINC02965 ENSG00000290490 LINC00158 MLLT3 CCNT2 BTG4 HAVCR1 DRG1 RSPH10B2 TRAPPC10 LINC02942 ENSG00000288714 AC034206.1 CPQ CNOT10 MR1 KRTAP21-2 LOC102724710 F8 TNFRSF12A OASL LINC00237 CPT2 AP000320.1 TAS2R14 VPS8 AC010329.5 MTX3 LINC00703 AC092121.1 ENSG00000286878 C4orf50 NXT1-AS1 LINC01732 AC020743.2 ISY1 ENSG00000287211 ZNF831 AC242426.2 VN1R31P CXCR5 NCKAP1 PRPF40A CLDN12 KRTAP13-6P DTD1 CNTN3 MGAM LINC02427 ENSG00000289085 LINC01060 CILK1 HOXC13-AS FOSL2 PACRG IL1RAPL2 HNRNPCL3 AC020912.1 CHCHD3 ZNF337-AS1 ENSG00000289131 LATS2 LINC02141 TYW3 MYH14 AC008581.2 APCDD1L-DT SPDYE2B MRPL32 TPRG1 ENSG00000286168 KLHL33 ERP29 CDYL2 ARHGAP18 AC013652.2 UVRAG SMIM10L2B-AS1 MYOM2 AC113386.1 LYN USP6 LOC100131779 LINC00390 MIR4636 LOC102723883 SUMO2 GRIK1 MTA3 CTSLP4 HSD11B1-AS1 LINC02203 UBL3 DIP2B LOC107985126 NME7 SEC24C AC068234.1 AC073869.5 MIR9-1HG AC109583.3 PRAMEF9 ENSG00000286686 ATP6V1H AC009135.1 NUP58 FAM177B FIGNL1 SLC12A1 LINC01538 AC068547.1 PDZD8 PAMR1 AC003006.1 CA10 LINC01878 RRM2 CRYBB1 SIL1 LDB2 MYRFL AC008035.1 AC024610.2 UNC93A MIR3159 C21orf62 LOC84214 ALDH1A1 NMNAT3 FMO5 RNASEL AC010082.1 LINC02008 NAV1 CMKLR2-AS DBF4B PAQR5 DSCAS CORIN TMCC1 LRRC7 PCDHB16 TEX41 LINC00458 AC060765.1 AC027288.1 CDH12P1 DCP2 RBPMSLP PDZRN4 SGMS2 FAM151B-DT TMED7-TICAM2 STX17-DT ENSG00000288055 SLC41A2 NOS3 FER1L6-AS2 AL355306.2 LIPE-AS1 HMGB3P30 LOC105378402 VSX1 EIF3L NOMO2 TNRC6C NALCN-AS1 ZNF90P3 PRAMEF27 LINC01208 PGAM1P5 MLLT10 BPHL ERCC6 EIF2AK4 LINC00840 RN7SL738P IGKV2OR2-2 AC016027.4 NIHCOLE MSRA AL031599.1 LOC100419786 SYNPR RAB12 SIMC1P1 LINC02235 C21orf62-AS1 LINC01505 IMPG1 HMGCLL1 LINC01911 DSCR4 XKR6 LOC101928335 ENSG00000287682 MAGEL2 AF165147.1 MATN2 TGFBR2 AC141257.2 CMKLR2 ITGA11 CFAP77 DDX60L MARK4 MDN1 AC092131.1 LINC01901 SYNJ1 TTC6 TCF7L2 LOC102724421 LINC02192 AC114781.2 CHAF1B ENSG00000290067 KIAA1328 ENSG00000286326 ZNF462 MTRF1 ECHDC2 AC090386.2 OVCH1 CRTAC1 E2F6 ENSG00000291336 CUX1 POLR2C UNC119 SHISA2 GPM6B RRAGD C2orf50 ULK3 AC083902.1 LINC02484 LOC339166 SPDYE2 GPR156 ZMAT4 CNOT10-AS1 ENSG00000289949 AP000529.1 AL445224.1 TUBBP3 LINC01596 GKAP1 CACNA2D4 RGPD1 PPP2R5E AL161751.1 ENSG00000288954 RPL39P33 KPNB1 INHBA-AS1 ENSG00000290385 AC010332.2 TTN-AS1 GSDMD ADGRV1 ST7 SERBP1 DNM1P33 AC013401.1 QTRT2 EVA1C AL161716.1 EPB41L5 GAD1 SCG5 AL035078.2 NUDCD1 AC068254.1 MIR603 AL050327.1 WAC WHRN OCA2 AC093668.1 TDP2 ULK4P2 ALDH9A1 LOC100419045 RAB6C-AS1 LINC01362 ZNF678 ACSM2B PRUNE1 TUBB8P6 SAR1B TTC7B INSYN2A BTG3 APP GATA4 ATF7IP2 SMAD5 IGF2BP1 AC116634.1 MAMLD1 RPL9P14 RNLS AC009093.4 ENSG00000291100 ANKRD20A7P AL163932.1 GSKIP AL136985.3 NEPRO-AS1 RNF38 ADAM12 LOC107986837 UBE2G1 NBPF12 AC138969.2 MIR1273F BTG3-AS1 DHX35 ENSG00000287347 SP140L CRACDL ENSG00000286288 CHRM5 SLC13A1 PI4KB AC129926.2 FRY LINC00670 RHOT1 ELOVL5 AC010809.1 AL354810.1 USE1 HOOK3 AL513323.1 PIK3R5 PALLD LYZL1 LINC02767 TAS2R30 TMCO3 CASC16 RFESDP1 BMT2 AC233699.1 OR52P2P RIPK4 LINC01876 AVPR1B LMAN1L ENSG00000286406 AC091073.1 GOLGA6L10 QDPR FRRS1 IQCM PTK2 AP002373.1 AC006482.1 ENSG00000293467 AC079414.1 NOP9 USP7 IGLV3-2 TICAM2 ENSG00000293472 SCP2 NPEPPSP1 SH2B2 LINC02518 ATP1B4 AL031963.1 AVEN AC019270.1 AC006153.1 ZNF41 LINC02250 AC046185.1 GATD3 CELF1 HYCC1 DNAH12 POTEG POGZ LINC01090 PIP4K2B PRRC2B SYNM-AS1 UQCC1 ENSG00000288996 LRRK2 INO80D AF279873.3 ZNF395 IKZF3 GET1-SH3BGR SLC38A4 AC006043.1 AC244517.4 MIR548XHG FRY-AS1 CLSTN2 ARIH1 SAMD12-AS1 CFAP298 AC015908.2 VTCN1 ITFG2 PKHD1 ENSG00000287039 MEG8 TENT5C UBXN7 KIF23-AS1* |

| | | |
|---|---|---|
| | | *GDNF-AS1 LINC02435 AC104169.1 ENSG00000290429 PJA2 SNORD114-7 TCF4 AC079742.1 ENSG00000287334 MGST1 RBM44 ARHGEF38 PAEP COP1 TIAM1 PCA3 CSRP3 PHACTR1 MLIP IQSEC2 ACVR2A AC243830.3 AC034232.2 AL033530.1 LINC02309 TIPIN VIT LINC01324 AC091078.1 BAALC-AS1 AL606760.2 RASSF4 C6orf58 LOC124902439 ENSG00000288635 MIR6882 AC092979.1 SNORA5C SNTG2 AC060834.2 NTM AC091230.1 AC091133.4 AMMECR1 AC004805.1 FP236315.2 CYP1D1P LINC00639 TMED11P ENSG00000289368 BRCC3 CDH12P3 ENSG00000288683 AC093010.2 LINC00636 FECHP1 DUSP14 LINC01237 PRMT8 SNX13 AC090365.1 AC093893.1 RABGEF1 AC068724.3 KIT CYB5R4 TBC1D8 ENSG00000273937 EPHA7 ENSG00000291067 OSBPL3 SPAG9 AC104574.2 ANTXR1 ENSG00000293304 RMEL3 LOC100652967 AC131254.2 AC069257.3 LINC02256 C19orf47 AC068299.1 AC007326.2 SPDL1 TRABD2B PEX26 TEX2 ARFGEF2 CTNNB1 AL671862.1 ANKRD17 EFCAB5 POTEKP AL096701.3 ENSG00000286811 COL19A1 RUNX1T1 CTSK LOC102723341 PRKDC AC097626.1 LINC00507 IPO5 AC026124.1 LOC124900584 TNFRSF19 ZNF479 AC091045.1 LUZP2 RPL3P1 PTPN14 LINC02240 SCFD1 ERC2 POFUT2 LINC02973 DMGDH BCAR3 TPD52L1 PPM1E ZXDC AD000090.1 VPS53 LOC100129404 FRMD4A AC099788.1 CTSLP6 LMX1A DCAF8L1 MIR3670-3 LOC128462377 TPST2 CIMAP2 PARS2 AC103740.2 HUNK TRAJ19 LINC01448 LINC00824 SNX29 FILIP1 LERFS TMEM62 FBXO15 EEDP1 PTGER4 AC018467.1 GREB1L AC015574.1 ZNF215 ENSG00000287392 PDE7B AC096711.2 USP40 PPP1R15B-AS1 RASSF8-AS1 VWC2L BEST3 LYPD6B DOCK1 HDDC2 ENSG00000287180 AC131902.1 AC027644.4 CCDC107 TARDBPP2 AC005580.1 GPC4 BPGM PKIG ENSG00000286614 LINC00276 RPL21P10 LINC00536 AC073316.1 IGF2BP2 AL035420.1 AL391869.1 ACTR3B AC018638.8 CDC14B ZNF722 SYNE3 ENSG00000287684 LINC02808 PLCH1 OR1M1 HECTD1 VKORC1L1 NGEF TRPC5 NEUROD2 FNIP2 ZIM2-AS1 AC097478.1 AASS JAML KAT14 CCNJ UBE3B ADORA2A-AS1 PTPRF LINC01473 IKBKB AC010280.1 DUX4L51 FXR2 SNHG33 SH3BP5 AC010809.2 AC068413.1 MIR3670-4 LOC101927690 ENSG00000293257 AC103409.1 TRBV10-2 USF3 AC002074.1 LYVE1 APOH AC004870.4 ARHGEF28 CLCN1 TASOR CCPG1 AC104781.2 GPRIN1 EML6 OPCML LINGO2 CEMIP XIST AC074286.1 ATF2 LINC00877 AC140481.3 AC091151.1 RNU6-986P KCNJ12 ZNF426 HDAC8 ACVR1 CHRNB3 HLA-DQA2 PID1 COL6A5 HYAL4 EBF2 CCDC91 GRID2 ZNF423 ZCCHC17 KCNJ3 AC008632.1 AP000487.2 GLDC PLPPR1 SNORA63D SLC17A6-DT RB1 LINC00466 NSMCE1 AC078923.1 MPV17 LOC100131635 IFNLR1 RNU6-826P GOLGA6L4 PDE4B AL365214.3 MYHAS DDX10 LINC02994 PKD1L3 KIF13A NLRP4 ADIPOR1 ENSG00000286655 SLC23A4P LOC124900205 GABRB3 ZNF143 GOSR2 PET117 AC093865.1 SMG7 PLPP3 UNC80 HNF1B RNU6-1311P IMMP2L AP001599.1 LAMP2 CCDC54 SSPN SPSB1 KIRREL3 GABRG2 F5 SCG5-AS1 CFAP95 AK5 CPS1 TMEM120A AC023300.1 AC104984.2 BNIP3L ENSG00000287614 AC021088.1 AGPAT5 RABGAP1L LINC01948 ENSG00000286476 LOC101927141 LINC00603 XRCC5 TBRG4 LOC107984536 HMGB3P2 LINC01965 SHLD2 GFRA1 SPHKAP KCND2 INPP4A BMX OR5W1P TRAJ18 ZNF418 SBF2-AS1 AC012593.1 SLC24A3 RN7SL563P RAD54L2 MPRIP LINC02351 TAF15 TPST1 GRIA4 RNA5SP489 RAB18 POLD3 AP000526.1 TPH2 POU2F1 AL079305.1 AL445648.1 LINC00508 PRPF40B AC244517.6 AC004870.3 AL592490.1 NRK RGS7BP BACH2 MGRN1 LOC339298 AC010745.2 TEX11 LINC02384 MID2 AC087683.2 RBM28 MIR198 LRTM2 LOC105375972 SIK1 FBXL13 OR10J4 GLOD5 ERCC6L2 ENSG00000291293 ENSG00000286779 PIPOX RPS2P1 AP002336.2 ZNF850 TP53BP2 LINC00592 KAT2B ESPNP LSINCT5 GOLPH3L WLS ACTR3C ANKS1A IGKV1-6 RYR3 TMPRSS11B PRCP AL035446.1 SUPV3L1 MEF2D MYO1B MGAM2 MYL4 RPL39P31 AC091588.2 GTF2A1 SPTLC1 AC015922.2 AL137076.1 AC008758.4 AGR3 CDK5RAP2 FLVCR1 LINC02343 AC023830.3 UBE2E3 FSIP2LP SAMD3 RPL15P18 RFC3 EPN2-AS1 FARSB ERICH6B CCDC54-AS1 ARHGEF7 AL445430.1 ANKRD26P1 SAMD12 ENSG00000293462 DACH1 CU634019.3 RPL21P5 LINC03082 ATF3 TRDN LINC01117 LOC105377043 NPHP1 ENSG00000290597 GASK1A AL136146.2 LINC00499 AC111152.2 TEKT4P2 ENSG00000290412 IKZF1 DAOA-AS1 AC005154.5 SLC26A8 CAPZA2 AC004147.4 GGT8P AC011405.1 TRAJ20 AC002066.1 SRI ATP1B1 GREB1 AL158154.2 ADAT2 ARNT BDNF TFCP2L1 ACBD6 SEMA3E BCLAF3 HAS2-AS1 SCIN AL163195.3 ENSG00000293265 AP004609.1 MACO1 ENSG00000293512 ENSG00000287443 CTBP2P10 NDST4 ADAMTS9 ADGRF2P STIM1 ITPR2-AS1 TSEN15 TMEM237 TMEM161B ENSG00000288563 HAUS6 RAB11FIP2 EEFSEC COX7B2 LAG3 DPP10-AS1 ENSG00000291189 Z95331.1 LOC124902888 TMEM72-AS1 AC016573.1 PAPPA LOC101927605 IRAG1-AS1 ENSG00000290217 ASB3 AC239585.2 MTTP GPR84-AS1 CHRNB4 LOC102724934 AC019322.1 AGAP9 CTBP2P9 AP000282.1 TGM5 NEK7 INTS15 ZBED3-AS1 ZNF80 PDXDC2P TENM1 IL13RA2 ENSG00000293489 RORB AL136441.1 GABRB1 LINC02903 MYOF AP001116.1 LINC01754 AC142384.1 LTB4R2 ATG12P1 JAKMIP1 SH3KBP1 NALCN AC116903.2 SMOX AC020718.1 AVIL LPCAT2 ENSG00000286163 GDAP1 AMY2A AC023509.6 AC126755.4 ENSG00000287801 ENSG00000289870 ISY1-RAB43 LINC00398 EIF2AP4 TTC39C MIR3670-1 MYLK4 BEND7 DIO2-AS1 Z98043.1 USP25 KIF2C AA06 MYO5A SIPA1L3 CTSLP1 SLC6A13 ZNF286A-TBC1D26 LRFN2 WDFY4 SPTBN5 AC068138.1 AC092862.1 SFRP4 BLTP1 SPDYE16 LINC01239 OR4Q2 PNRC1 ULK4P3 AC015922.3 ENSG00000286662 AL772307.1 JCAD VAV2 TFRC DANT1 VPS13A TES OSBPL6 SH3TC1 GRIA3 PNPLA8 SPDYE6 AC087564.1 AC108474.1 FBN1 HAPSTR1 SERPINA7P1 RAB31 EXO5-DT ENSG00000291283 DIPK2B MAP3K1 ZFP82 DDX4 IQCB1 COA1 CLEC6A ZNF292 BMPER FUT10 AC109779.1 LINC00922 RNU6-984P CLASP2 RPRD2 ZNF761 AC024230.1 DUXAP10 VIPR2 SEMA6A ZNF567-DT PARAIL EIF4G1 TMPRSS15 AL133372.2 EEF1A1P11 B3GAT2 ATP6V0A4 ACSM5 RBKS ZNF618 ITGA4 DNAJC6* |

| | | |
|---|---|---|
| | | *CXXC4-AS1 SCIRT ROCK1 AL390860.1 NCAPH M1AP ENSG00000286072 TAX1BP1 AC008739.5 OR7A15P AL445430.2 TBC1D4 AC093843.1 APIP AC012456.1 MMP28 FAM13C ZNF701 LINC02971 NECTIN1-DT KCNT2 CRYBB2P1 NUP98 CLEC3A PML BX571673.1 FOXO3B AC012616.1 LAYN MGLL SCFD2 SCARA5 LONRF3 SLC5A12 PLEK2 TNFSF4 ENSG00000293021 DGKG LINC00923 SP100 ENSG00000287616 RNA5SP260 PLXDC2 ELAVL4 CDH9 RF01880 AL731556.2 FOXRED2 AL359736.1 CFAP298-TCP10L NDUFAF2 LINC01524 UMODL1 AC022335.1 RFPL4B TSEN2 GALK2 PCDH19 ENSG00000287783 LINC02060 NIN SPG21 ZNF286A GARS1-DT ST7-OT4 ANKFY1 ZNF69 CCDC144CP PRSS50 MIR4645 ENSA ENSG00000286259 PPP4R1L MIR9-2HG FNDC3A KHDRBS3 SLC30A10 AIMP1 AP4S1 FOXG1-AS1 MYO5BP1 TPK1 HSPBP1 CALCRL POT1-AS1 CASC17 SLC20A2 USP34 AC091053.1 STK4 AC138305.1 PCDH10 AC023300.3 ENSG00000289174 TNNI3K AL646090.2 ENSG00000287184 DNAAF4-CCPG1 RAB6D TMEM131 SPC25 AC112493.1 ENSG00000287378 ST6GAL2 RNASE11 AC018742.1 AC244205.1 SCAMP1 TXNL4AP1 PTCHD4 RPL27A ANPEP AC021979.1 PRLR LINC01821 ZNF569 GOLGA8O LINC01697 ARL2BPP10 ENSG00000289397 AP005436.1 ZNF180 AC008758.3 NMI RAB6C PCCA CLVS1 YLPM1 IL16 LINC00972 ENSG00000291276 AC012485.1 DIPK1A LINC02814 SULT1C2P2 ZSWIM7 ZNF470 ENSG00000286932 AC107373.1 LINC02339 POTEH EDARADD LINC00558 LINC02511 CR2 OXNAD1 GNA14 ENSG00000286152 LPAR6 ZNF56P CTBP2 LINC01721 NCALD RPS10P13 HHIP DYRK1A LOC105377209 PPFIA1 TMEM30A LINC01705 MYRIP ERICH2-DT LINC00343 PPP1R1C ZNF286B AL355838.1 KCNE2 UNC13C RGS10 CUL4A NDUFAF4P1 AC105052.3 SERINC1 LINC00265 KCTD16 CHSY3 TXNRD3 TRAM2-AS1 AC026358.1 EYA4 ZNF888 AL391361.2 LINC01748 AC069287.3 AL163953.1 ARL15 SPATA13 LINC01288 ZNF736 AL031432.1 MAP7D3 SEMA5A ENSG00000288035 AL354861.2 LINC02762 ENSG00000290805 EIF3FP1 ELL2 LINC00886 LAMA4 PMEPA1 LINC01811 UBE3C ATE1 ENSG00000293330 ETNK1 AC110772.1 TTPA MYO5BP2 CYP4F30P PVT1 SLC2A1-DT GCNT1P3 CHAC2 GATAD2B METTL3 KRT85 UAP1 ZKSCAN7-AS1 PARD3 LINC00691 KCNN4 LINC01104 TRDC ZNF33A AL353132.1 CP FUNDC1 RNU6-849P COL6A6 BMAL2 PGPEP1 AC026462.4 BZW1-AS1 RNF6 SEMA3C VAV1 SMYD1 PEPD ENSG00000289871 DPY19L1 GRM3-AS1 GRIA2 VDAC2 KIAA0825 AC002127.2 MIR3670-2 ENSG00000276197 SPART AC018697.1 LOC102723684 IGLV4-3 LINC02315* |
| Mel Z Matrigel | 1798 | *SLC18A1 OR2AF1P KLHL13 ABCB7 AL355499.1 PBX4 ACOT12 NHLRC3 LOC124903770 ENO1P2 OR11P1P MYO9B LINC00683 COL4A5 LINC01708 ERG ENSG00000293384 ENSG00000291325 PARN AL390816.1 STK16 XK SEMA4D MET MAX TNS3 PDE4DIPP4 SCYL3 TEAD1 LOC105370954 EIF4G3 ACSM3 AC231532.2 SNX16 AC116035.1 WWC3-AS1 LINC02476 LINC02487 LINC01915 SPAG11B IFTAP EOLA2 LINC00261 SUMO1P2 ZNG1F LINC01944 LINC02997 SIAH3 DUS3L CETN3 AC116424.1 RASA4B ARL14EP-DT GAS2L1 RPL4P1 AC108025.1 CHMP1B MMP16 OR7E25P ENSG00000286104 METTL25 AC091231.1 ELAPOR2 FAXC PRORP ZNF534 TOX3 POLR2J4 LINC02334 DNAH11 RALY-AS1 SLC9A9 CDC42BPA POLR2J POLG AC092957.1 EXOC2 DBIP2 AL358934.1 MYLK-AS1 TACR1 FSIP1 ENSG00000289332 AC068672.2 SNX25 SDC2 LINC02064 AC131571.1 ABHD12 AP002954.1 AC005999.1 COX10 CIITA FANCC AP006219.1 ARAP1 UQCRHL LINC00305 AC073488.11 HEPHL1 RN7SL354P ANKLE2 GACAT3 FAM218A CEP85 RPRD1B CR381653.1 LINC01828 CIP2A LOC105371855 AHNAK ACTR5 PPP1R3B-DT YBX1P5 EIF4A3 DLEC1 COX10-DT AC004943.3 NFE2L2 AC069335.1 SCHIP1 AC004594.1 SLC46A2-AS1 TFEC ENSG00000286830 NFYC H2BC18 BLOC1S5-TXNDC5 UPP2 CYP19A1 LOC105374367 SLC46A2 UBTD1 AC073488.10 LINC00313 DTNB GPR158 VRK2 ITSN1 AC092552.1 R3HDM2 ATP2B2 AC073488.2 ESPNL PREX2 SCRN1 AC007314.1 FAM177A1 ELAVL2 AC073488.5 TANC2 MAP9-AS1 PSKH2 ENSG00000286332 AC135507.1 NAV2-AS4 CSMD3 FBF1 NTN4 SERP1 AC090888.1 POLR1A PLEKHM3 ENKUR LOC105375146 CENPBD2P TPTE2 AC017002.6 RAB11FIP4 AC010307.2 CLMAT3 AC116353.4 C10orf53 HSFX4 NPIPB5 SNTA1 PPCDC CHD1-DT RPL7AP83 ENSG00000289178 ENSG00000293110 LINC02578 TTC28-AS1 CBX3P10 CCDC144A ASZ1 CU638689.3 ENSG00000289699 NUB1 SRGAP2C ENSG00000293482 WIF1 MISFA FLI1 DYM-AS1 LINC02197 PABPC5-AS1 SPATS2L CLIC5 ESRRB ZFHX2 ARHGAP26 LINC-PINT RBP7 NR2F2-AS1 TRHDE RAB44 PTPN4 AC105180.1 LEMD1-AS1 SLC16A11 SETBP1 ZBTB7C ITGA2 ENSG00000288643 AL442647.1 ENSG00000290070 KARS1P2 MIR5702 AC093459.1 AC026316.5 IL1RAPL1 AC091564.3 KIF5C BLK GPM6A SPATA16 AC068205.2 AC034195.1 WNT16 RERGL KRT86 SETD3 MRPS35P2 ACOX3 ADCY1 GRID1 RPS6KA2 EFR3B PLPPR5 LINC01790 TRIM24 ZDHHC17 HLCS FIG4 AC106864.2 LINC01621 AP1S3 CFAP47 FOXP2 LOC102724452 RNU6-389P FP325331.1 ENSG00000288016 FBXO34 ENSG00000286069 KMT2D GUSBP5 LPAR3 C3orf49 CKMT1B SHE ANO4 GIPC2 AC008825.1 GFOD1 ENSG00000288106 TXLNG SP110 SH3BP2 SH3TC2 CEP290 FAM171B CNTNAP3P2 AC239859.1 LRIG3-DT TACC1 SH3PXD2B OCLN AC092484.1 CFAP91 AC024598.1 FASTKD5 NETO1 EGFR PTPRQ TNPO1 GBE1 AC097634.4 SNX32 PCGEM1 DNPEP LOC100506321 LINC00589 AF130417.1 NEO1 GLT8D1 LINC02894 MYT1L SRGAP2B STAG1 LINC02428 CLDN14 IQCJ-SCHIP1 AC004702.1 CARM1P1 GUCY1B2 LINC01968 PDE3A-AS1 LINC01829 PLEKHB2 TIMM23 CAPN11 UBOX5 TCERG1 LINC01608 AC024257.1 LINC00869 F10 SLC7A14-AS1 CKMT1A LINC01323 CFAP20DC-DT LINC00970 DNPEP-AS1 LINC00370 SCN1A-AS1 LINC03099 FAM184A ARHGEF35-AS1 AC073325.1 CABIN1 PSMD10 FBXO47 KRT89P CCNO-DT FBXL17 PDZD2 EOLA1 SLC9A4 DHX29 STPG2 AP004833.1 GPAM MSRB3-AS1 IRF2 HEATR6 GET1P1 SPATA6L LRP12 TCF12-DT AC093766.1 WEE2-AS1 LOC100287944 ENSG00000291120 AL353133.2 TBC1D22A* |

| | | |
|---|---|---|
| | | *LINC01492 ESRP1 HSFX3 NPIPB4 UBE2A BRMS1L RBM41 GOLGA6L1 AC099329.2 AC011477.4 RGS12 LNCOG AC245102.2 LINC02963 ACOXL AC007529.2 CBX3 LINC02955 ACAP2 SLC39A10 PRKG1-AS1 AC020687.1 ENSG00000289788 ZNF496 ZNF550 CHCHD6 KIZ MPHOSPH9 LOC102724289 RNF10 MGC4859 ENSG00000287051 FAT1 SLC2A2 PIK3R1 AL078621.3 AC139143.1 IL17B LOC101928253 AGMO NOVA1 SARNP SHTN1 ATG12 AC100818.1 FNDC3B AC073488.4 MASP1 AC034114.2 TMEM232 CD200R1 LINC00587 BTD CALD1 CCDC144NL-AS1 SH3GL2 ZNF847P CLBA1 HIBADH ENSG00000288577 LINC02233 CRTC1 VCF1 FHIP1A N4BP2L1 PITPNC1 SLC30A3 LINC00663 AL137009.1 DOCK7 ENSG00000286353 AC106706.1 PDK3 PTCSC2 XKR4 FBXL4 ROR1 SMAD3-DT RAPGEF4-AS1 CCDC178 ADAMTSL1 PAX5 RTL8B AC090023.2 AAK1 LINC02755 CRB1 LINC02436 UTP15 AC116353.5 ARID1A THEMIS LOC124900945 SMAD6 RYBP NYAP2 PSD3 ABCA13 AL355922.4 AC012368.1 MEF2C ZNF519 FZR1 ST8SIA5 TBC1D19 DBNL PPP1R9A-AS1 AC005381.1 ELP4 RYK MACF1 AP2B1P1 PXT1 STARD4-AS1 DENND11 AC079466.2 TUBGCP3 ITIH5 WEE2 IQCK SLC15A5 TFDP2 MRPS10P2 AC110296.1 NDUFAF4P3 LINC02930 PTPRE MFSD6 SERINC3 AL035401.1 TBC1D31 ZNF347 PTPN12 PTPRZ1 LINC01749 AC105450.1 ACACA AC026786.2 PLN EML1 DDX39BP1 CTDSPL2 SLC66A3 SLC16A7 ALDH7A1P2 NEDD4 OCLNP1 NEK4 CACNA1E AC073488.9 ENSG00000293315 LOC105375297 AC024559.1 FLYWCH1 LRRC38 OVAAL AC078777.1 CYSLTR2 VWA8 PARVA LRRC37A2 AC106729.1 AC002064.1 SECISBP2 LINC02237 URI1 LOC127903862 MARK3P1 SLC9C1 SCML2 ANKRD28 AL031847.2 SNAP25-AS1 CHD6 PDE4DIPP2 ZNF609 PRDX1 NDUFAF6 PACS2 AL121782.1 ATG4B AEBP2 SLC7A14 AL133255.1 DSC2 TRG-AS1 SERPINA1 GLB1L NPSR1-AS1 LOC100132172 LINC00376 OLFM1 RN7SL77P MID1 AC020897.1 POGK TSHZ2 LINC00862 SLC12A2 OTX1 EFNB3 TRIM37 LOC101926964 ENSG00000286432 LINC02775 LINC02458 DLG3 LOC105376219 ENSG00000291284 PROSER1 FLOT2 PVRIG SRP68 SV2B LINC01879 ILK LEF1 EYA1 DAAM2-AS1 ZNF816-ZNF321P MAPK14 MYBPC3 WDR17 AC011499.1 ENSG00000290921 ZFHX4 RAPGEF1 ALG13 EFCAB14 AC068633.1 GPRC6A AC009139.2 EOLA2-DT UNC79 LOC100129616 ZNF516 SLC1A2 HOMER2P1 ZEB1-AS1 AC027228.2 EIF2S3B ARHGEF3 GABRB2 BTBD9-AS1 LOC101927293 AC010196.1 GRIN2B UBXN10 SVOP CRISPLD1 NOP16 IFNGR2 ROR1-AS1 DENND4A TBC1D20 AC083939.1 PLD5 OXR1 AC245748.2 LINC00700 SFMBT2 SNX2 LOC729732 GRHPR AL929601.1 SCAMP5 IL17RB PARP2 MINDY3 ZSWIM6 MYL1 KLHL4 AC006041.1 TTC3-AS1 LINC03096 EIF3F RDH16 FCHSD2 SLC4A7 CFAP418-AS1 RNU6-1117P DNAJB6 F10-AS1 LINC02612 LINC02279 RPL23AP7 NPIPB3 ENSG00000289530 ENSG00000290967 LINC02683 ZNF724 PRR4 SPCS1 MTUS1 LEMD3 PLEKHF2 B3GLCT PIEZO1 OR2T11 GGT4P SPTLC1P2 ENSG00000287042 NEB AC103719.1 AL157359.2 AC090888.3 CSRNP3 AC087762.1 KANSL1L AL157944.1 MRGPRX1 KLHL32 AC018618.1 TMEM100 LINC00861 LINC02253 NUP210 PDIA5 LOC127814297 VDR C16orf74 AC097625.1 CCDC149 NEK11 CREB3L2 RTN4 ENSG00000286962 AC096887.1 GNL3L CACNA1D ARHGEF11 PRKAA2 IVNS1ABP CD163 SGPP2 DPT BBS2 CLSTN1 TMEM150C ZNF155 SERGEF SCYL2 AOPEP SKAP1 AC190387.1 CU634019.5 KRT18P35 LINC02375 AC022816.1 CHM AC026415.1 SKIC3 MRPL3 SERPINE2 MYO3A ITGA2-AS1 ZNG1E LOC101928866 NUBPL PLAAT3 GGNBP1 IL12RB2 LINC01221 LINC00363 LOC100507336 PIK3CG ANOS1 COL4A4 TPP1 LOC100130691 ANKFN1 STIL TRGV5 ATP6V1E2 NBPF10 AL935212.2 NUP160 FGF14-IT1 MCCD1P1 AC132803.1 AC137810.1 CDHR17P AC121757.1 IQGAP1 MYCBP2 WIPI1 MIR6841 LATS1 AC098850.3 NUP153-AS1 ZSCAN25 PHYHIPL ANKRD20A21P LINC01442 AC007881.2 LOC124903099 ENSG00000293441 KLHL12 ENSG00000286215 LOC349160 ENSG00000286097 SNX30 AC007092.1 LINC00942 FANCI THNSL1 OR9Q1 ENSG00000290589 ACAA1 CPEB2-DT ITGA9-AS1 PRSS23 WNT2B CPHL1P LRRC37A3 ENSG00000287299 SPNS3 EXD3 TNFRSF10B IUR1 MAP2 RNU6-973P AC023078.5 GRM5P1 ENSG00000286982 NPIPB13 ENSG00000287771 FBXW11 MTCL1 HSPH1 AC008268.1 SUGP2 AZIN2 IYD AC108517.1 AC026992.1 SLC29A4P1 HNRNPA1P36 MIR646HG NHSL1 SMG6 HLA-DMA AC093802.1 CEACAM16-AS1 AC010343.3 TCERG1P2 LOC105370500 ENSG00000286041 PEMT ARHGAP6 ENSG00000289503 LINC01845 PPP2R2B CHD5 LINC02196 JAK1 GPD2 IL12A-AS1 ITGA9 KCNB1 RADIL LINC00298 TRIM39-RPP21 AC084200.1 AC105919.1 GNPAT HSD17B12 HS6ST3 GRM4 ANKRD27 LOC100887080 AIM2 ANK3 LINC01283 LINC00911 RF02271 LINC00578 ZNF19 TEX21P TMEM132B MARK2P11 AL391095.1 AMT ENPP7P10 KITLG ARID4B SATL1 GMDS NDUFS5P5 PLEKHM2 NR1I2 ZNF266 THSD4 PENK-AS1 LINC01471 ENSG00000286371 LINC01414 AC073488.8 AC004852.2 AC068313.1 RFX7 RELN SRP14P2 ENSG00000286556 AF107885.2 LRRC37A ENSG00000286239 STAG3L4 PDHB ADAMTSL3 NXPH1 KMT2CP1 AC012467.2 CRKL LINC00871 TENT5A ARHGEF10 SYTL3 NDC1 AC009093.2 SULT1C4 LINC02742 LINC02074 SNAP91 ZNF816 AC104452.1 CDKAL1 VAV3 RPS6KC1 CD40LG VRK1 AC100802.1 ENSG00000287950 PAPOLB URM1 LINC03041 GPAT3 ENSG00000287862 SCN9A ZNF420 DPYSL3 AC073488.3 C1orf146 SPRING1 AL807742.1 SLC16A10 PLCE1-AS2 LINC01252 ATRN NDUFB9 CCDC88A SNORA36C SLC35B4 ARNT2 UGT3A2 SPOCK1 RPS20P32 PITX1-AS1 HPSE2 PLCE1 AC007262.2 TNIP3 FAM106A TRMT2B-AS1 ENSG00000288799 TACC2 AC073488.6 AC009950.1 NT5DC1 CYP39A1 AC099511.1 AC006927.5 FRAS1 AC022568.1 DCAF5 LINC01423 GNAL AP004607.6 CSTF1 STK32C MIR7110 AC090114.3 OR7A1P RNU6-10P MSH3 UBE2F-SCLY LINC02664 BAK1 MRPL45 LRRC37A4P AC034268.2 DIS3L2P1 LAX1 AC013644.1 LINC00705 PTPRG-AS1 STK26 AC002428.1 ENOX2 TENM4 AC068152.1 GOLGA6L25 TTC9-DT LOC102723446 RPS12P20 PPP3CA CHRNA5 CES2 AC133065.1 DUX4 MKNK1 DYSF NKD1 AL157762.1 ENSG00000286856 ENSG00000287849 RBPJ* |

| | | |
|---|---|---|
| | | *KMT2CP3 AL356807.1 RIN2 PDE6C AL137247.1 AC016629.1 PLEKHG7 DLX6-AS1 ASCC1 CDH10 DENND1A MZT1 LINC02068 ENSG00000290317 PDZRN3 ADAMTS16 TASP1 LINC00381 NBPF19 MICU1 AC008700.1 TAF10 ENSG00000287329 MBD5 BACE2 KDM5A AC009154.1 ZNF549 AC010328.3 AP002761.2 LINC02026 LOC102724354 LOC101927855 NOMO1 AL133375.1 ARL6IP6 LINC01579 CNOT6 PKIB SPANXA2-OT1 LINC02284 TBX2-AS1 ASAH2 FRYL LINC01145 TBC1D9B STON1 PXMP2 ASH1L RSPO2 LINC02266 AC016598.2 AC010636.2 ZNF805 ACSF2 ENSG00000288632 OR5K1 OR13C9 DISC1 PROX1 MAP3K7CL FMN1 ZNF891 RALGPS1 CCDC6 PAQR5-DT PIEZO2 AC026474.1 AL133353.1 LINC00613 Z96074.1 VSIG1 CU633906.3 LRRC42 ITGB1 RBM26 AP003108.3 AC022031.2 RNA5SP29 CYB561 AC010632.3 DRC7 ZNF827 AL109837.2 UBA5P1 LINC00240 DSE LINC02346 LINC01823 ENSG00000291175 RHCE CORO1C LHFPL6 SPIDR CCDST C17orf80 AP001180.5 NEFL NAV2 TMEM185B STK3 CHN2 RNF150 NBEAP3 FCAR FSIP2-AS1 C1GALT1 HNF4G SMCO4 MSR1 EXOSC3 MED15P7 GABRG3 ENSG00000290790 PSMD7-DT AL138752.2 LINC03095 DPY19L1P1 AC245060.5 SH2D3C APTX TRPC4 RAMP3 GNG2 ZNF705A FRS3 PCDH11X CPB1 LIMS1 LINC01456 ENPP2 DOCK4 ACKR2 ARPC1A LINC01900 LINC00382 MCTP2 ENSG00000286458 BCL11B CERS3 GLCE PLS3 AC062021.1 TSPAN2 PCSK2 KCNIP1 PWWP2A LOC105373170 SLC29A4P2 ZFPM2-AS1 TBC1D10A ENSG00000287188 SSBP2 REEP1 RPH3A CTTNBP2 LINC01088 NME9 CYTH4 NAP1L1 SLC38A6 AC245297.3 AL121594.1 HDAC2-AS2 VPS29 PLPP4 CCDC102B LINC01985 AC132219.1 COPB2 UBR1 AC005972.3 CNN2 COL4A2 SLC1A6 KMT2CP2 ARHGAP32 DYRK3 IP6K2 AC114501.2 AC069444.2 LNC-LBCS CHRNA6 AP000688.3 AP002765.1 LINC02929 AC002428.2 ENSG00000287478 TLN2 HDAC4 F11-AS1 LINC01276 FYB1 RIOK3P1 AL935212.1 RBM45 SEM1 AC073488.1 GYS2 IQCE LVRN RPGRIP1L MYLK SPRY4-AS1 UNC5C CFAP69 MINDY4 CSMD2 TLR7 FLG2 PABIR3 P3H2 AC064859.1 CFL1 BAZ2B LARS2-AS1 PPIAP76 ENSG00000288067 LINC01793 DTYMK ENSG00000290821 WDR7 DIAPH2-AS1 SLAIN2 HMG20A CEP85L TUSC3 COPG2 PLEKHG4B ZFHX2-AS1 RBBP8 AC090517.4 MED13 RNASEH2B PHF2 TAAR2 RAI2 AFDN CAPS2 DEPDC1-AS1 LRP2 CYTIP MTDH ENSG00000290606 ENSG00000287918 ENSG00000288804 GOLGA6L6 LIG1 ZDHHC13 WWC3 LINC02715 CAMK4 SERPINA9 GALNT14 CLPX AC009084.1 VWC2 NCAL1 LINC01170 REPS1 FBXL7 RPA3 TOMM40P2 RNU6-1229P TMEM108 RPL29P26 CC2D2B CLUHP10 CHMP1B-AS1 DOCK7-DT MB21D2 SEZ6L AC005828.4 PCYOX1L SLC39A8 AFG2B MANCR AC009242.1 LINC00412 MAP4K3-DT UPRT ATXN1 TMEM59L ADAM17 LINC00968 ENSG00000286185 FAM78B SEMA4F AL133353.2 ELAVL1 LIPJ ZFHX3-AS1 AC004922.1 TRAK1 STXBP1 OR6N1 ENSG00000287923 AC008696.2 IDH1 AL109914.1 LMOD2 AC092329.1 LINC00424 ENSG00000289846 LOC112268173 MIR4713HG TSNAX-DISC1 LINC01958 HDGFL2 MUC13 NUP205 PLEKHG1 NHLH1 AP005203.1 ELP2 SLCO5A1 RPSAP37 AC021443.1 SNTB1 ORC4 LINC01723 AC135586.2 AC022031.1 AC021231.1 CYP8B1 ENSG00000286500 ZNF350-AS1 APC PEX5L-AS1 LINC00540 AC090371.2 TTLL5 INO80 AL513325.1 NAA11 LOC728554 MOCS2 ERGIC2 PEAR1 CDA LINC01591 BANCR PPARGC1B STON1-GTF2A1L CTRB1 COL27A1 AC093523.1 ESR2 AC239727.1 AC005225.3 AC124254.1 AL359237.1 AC091551.1 AC108081.1 NOL10 AC092650.1 AC068759.1 LNX1 AC087386.1 LOC105374191 ENSG00000287694 ERC1 SMIM13 AF228730.5 MIR8058 PARM1-AS1 ZNF440 RUBCNL AGO3 UBOX5-AS1 C9 CYYR1-AS1 PVR CASTOR3P CKAP4 RPL39L ACER1 IQGAP2 CDYL RLF AC087289.4 ENSG00000290507 AC092645.1 TNPO1-DT ANKRA2 C12orf42 TAF5L AP000265.1 JPT1 CTNND2 CU633904.2 DNAH14 LARRPM AC010197.2 DELEC1 RASSF3 UGGT1 PACSIN2 RPL36AP47 MIR181A1HG DPYSL2 NEK2P4 ENSG00000288700 ENSG00000287169 NUP210L AC008574.1 CNTNAP3C AL049775.2 GIMAP8 RAB8B METTL8 NSD2 AL023574.1 ZNF516-DT GRM8 5_8S_rRNA PHACTR3 AL139352.1 AC107023.1 EIF3J-DT AC005261.3 ENSG00000291209 AARS1 KCND1 ENSG00000286499 TMEM267 JADE3 CARD18 PHF3 PSMC2 ENSG00000287831 MAPKAP1 LINC01853 EXOC6B LINC01950 EVC2 GON4L LINC03017 ENSG00000293214 AC007179.2 AC007780.1 ENSG00000286812 DNAH6 MGAT5 IGHV3-42 ACOX1 USP24 HAO1 NUP153 FBXO38-DT LINC01581 HSD17B4 EFHD1 LCN12 AL500522.1 TRMT10B TRAV32 COL21A1 UFL1-AS1 RAI14 ENSG00000292277 LOC285626 GPATCH2L PIGR NRP2 GTF3A SIPA1L2 CAVIN1 MIR548AD GYPE MSL3-DT C1orf21 METTL15 TMEM165 AC016745.2 LINC01229 FOXJ2 RNU4-69P CEP162 HECTD4 ELOVL7 AC092111.3 NR3C1 DLX6 KMT2CP5 AC011466.2 BORCS5 FSTL4 ZC3HAV1 MTOR XACT LINC01797 ENSG00000287907 TRAV5 NBPF20 KSR1 FAF1 LINC02217 SNAP25 AC004691.1 SGSM1 DEFB124 ARL10 MFSD4B-DT CHST4 CCDC144BP AC005050.1 OR11H6 ENSG00000287258 AC083795.2 ENSG00000290565 GOLGA8A CLDND1 ZNF221 CR381670.1 PIP5K1A BTBD2 GH2 MMACHC AC131025.1 AC008539.1 HS6ST2 RAB6B KYNU AC015468.3 LOC105370906 ADAL TBXAS1 ASCC2 AC046136.1 SPRED1 ENSG00000289744 GALC CNTNAP4 GNG4 NUDT19P4 TUBA3D CDH26 ENSG00000293242 RNF32-DT SNX19P4 PDS5B CRIM1 ENSG00000289487 MEI4 NALF2 RBM46 ABCB5 RNF115 TRIM61 ESD CYYR1 CDHR3 LINC00689 PER2 LINC01076 ANKRD24 GLI3 IPO5P1 LHPP KAT6A BCOR KCNK12 NTRK3 LINC02932 RXFP1 LINC01493 ENSG00000287729 LINC01807 TADA1 GAB2 DIS3L2 ENSG00000290702 ENSG00000287291 SNTB2 ENSG00000286954 AC073263.1 RSBN1L PDE1A PDCD10 LOC107985643 PRAMEF11 LMTK2 AC023055.1 AL133268.3 HHAT LINC00632 CREB5 PSMA3P1 FAM227B CCDC68 ADGRG6 COPG2IT1 GOLGA7 AL357153.3 FERMT2 AC245519.1 GCNA GPR82 AC068587.3 AC130710.1 TSPOAP1 AC008056.2 GLUD1 AC016885.1 DNM3 ST3GAL2 SREK1IP1 PHIP EPB41 DENND5A LINC00309 SS18 LOC124900792 SFRP5 DOCK9 RPL23AP38 ADAM6 GOLGA6L24 AGBL3 ZYG11B PPARA NCKAP5 AP000311.1 CD244* |

| | | |
|---|---|---|
| | | *LINC01643 LINC01933 RN7SKP173 SHISA6 IGLV3-1 NFASC AC122134.1 OR2A1-AS1 HDX RAI1 CPEB2 AC104339.1 LINC00513 ZNF229 GORAB-AS1 NXPE4 PHF6 HMGB4 ENSG00000288075 ENSG00000287533 FGF7 TENT5D MARCHF10 TBCA STXBP6 MGA DENND2B-AS1 AL137782.1 AC021660.2 FOXP4-AS1 RTTN CACNA1A AL596087.2 RN7SKP284 EDA LOC100533679 LIMCH1 TNFSF13B Z93403.1 AC006288.1 ENSG00000291170 MTMR7 PTH2R CNTNAP3 AC005909.1 LOC100420587 IAH1 AC073488.7 COL14A1 RNU2-38P RBM47 AC005828.3 EIF2A ENSG00000286209 JARID2 CYP1B1-AS1 CYTOR HDAC2 FIRRM TPO TGOLN2 FGD1 AC008415.1 INSYN2B TRIM39 TRRAP AC009090.6 SEMA3D AC105213.1 ENSG00000286134 LINC01422 NPSR1 GLIDR EIF4E3 USP48 KCNH5 RRM1 MAGEA1 CADM1 INPP5D KLHL29 CFAP161 THUMPD1 AL356309.1 CLYBL OR4C6 RN7SL797P ATF6 PRKAR1B GBF1 IPO11 LINC02805 CCDC88C SERPINE1 ABHD15-AS1 BACH1 CUL9 ENSG00000289228 PRAMEF4 EWSR1 INMT-MINDY4 TM7SF3 SLC26A5 AKAP13 AC091047.1 PKM GPAT4-AS1 GCG MRPL45P2 ATP12A CCDC150 LINC01735 TKFC ENSG00000289293 LINC02218 SMARCAD1-DT PTPN3 ENSG00000292979 SEPHS1P1 FOXP1 STAC SCN8A AC010547.2 AF241728.1 RTL4 NNT SSX2IP ST8SIA2 TTC17 ELP1 IRF8 HIVEP3 AC074386.1 PHF5AP1 AC005999.2 AC018629.1 AL008638.3 HEPACAM2 KAT7P1 AC023824.1 RFX4 CCDC198 OR6K6 ZNF30 ZRANB2-DT REM1 LINC01482 AGAP1 FREM1 RIPOR2 AC091564.7 NDUFA10 CBR1-AS1 CD69 FUNDC2 DBR1 EPS15L1 CYB561A3 PARD3B AC021660.3 ENSG00000291188 XIRP2 Z99571.1 AC079950.1 LINC01248 KDM3A AL356272.1 ENSG00000293483 MZT2A CAST AC005394.1 KBTBD11-OT1 GPR39 ADGRA3 CASK HSPG2 SLCO4C1 RPS29P9 GCNT1 BPIFB3 AF121898.1 SLC35F4 GGA2 AC008277.1 USH1C AC118758.3 LOC101927468 LINC02307 AC136428.4 NLRP14 DNAJC13 FRMD4B LOC345471 PSME3IP1 GOLGA8B LINC01075 TRDV3 LINC01478 ENSG00000287469* |
| 188 | 53 | *CROCCP3 SORBS2 ZNF536 ACSS3 TBL1XR1 FBLN2 MBTD1 TBC1D32 GULP1 MPPED2 PTK2B ZMYND8 ATP6V0E2-AS1 FLJ43315 PRICKLE2 SHROOM3 KIF18A KMT2A ZNF607 ANKRD55 MEGF10 GGTA2P GABBR1 CFAP44 SLC7A11 MIS18A-AS1 STK32A-AS1 RERE NR3C2 DMXL2 TENT5B MX1 CLDN1 TSHR EYA2 LINC02293 ZNF197 LINC00906 TRPV6 IQCH CAMK2D ST8SIA1 STK32A ZNF234 KBTBD11 TRERF1 ENSG00000286207 LAMB1 NFIB SRGAP3 BCL11A NECTIN3-AS1 ELOA-AS1* |

**Table S9.** The 163 downregulated genes are simultaneously regulated in different combinations by 543 different transcription factors. The search was performed in TF-Gene Co-occurrence (Enrichr Submissions TF-Gene Co-occurrence, https://maayanlab.cloud/Enrichr/ ). Related to Figure 6C.

| Term | Overlap | Adjusted P-value | Genes |
|---|---|---|---|
| ZNF407 | 29/299 | 8.550545640156517E-20 | *MACF1;DOCK9;AGAP1;CBLB;FMN1;BAZ2B;SYNE2;GLI3;IGF1R;SYNE1;GLI2;ATXN1;DISC1;TNS3;NCKAP5;SRGAP1;TEAD1;MBD5;FNDC3B;ANK3;FOXN3;NAV2;CDC42BPA;FOXP1;PARD3B;SETBP1;ZNF516;PLCB1;CREB5* |
| SCAPER | 25/299 | 1.2614735579368733E-15 | *MACF1;DOCK9;AGAP1;CBLB;BAZ2B;SYNE2;IGF1R;SYNE1;ATXN1;TNS3;FNDC3B;MTUS1;PLEKHA5;ANK3;FOXN3;NAV2;CDC42BPA;FOXP1;TGFBR3;SLC4A7;INPP4B;FCHSD2;SETBP1;PLCB1;SSBP2* |
| SOX6 | 25/299 | 1.2614735579368733E-15 | *NLGN1;SEMA3D;TSHZ2;SYNE2;GLI3;IGF1R;SYNE1;EDNRB;PDZD2;ATXN1;TNS3;NCKAP5;TEAD1;VAV3;EBF1;MTUS1;ANK3;FRMD4B;NAV2;FOXP1;PARD3B;TGFBR3;SETBP1;PLCB1;CREB5* |
| NPAS3 | 24/299 | 8.103927312947807E-15 | *VAV3;NLGN1;TSHZ2;EBF1;AGAP1;ANK3;FOXN3;NAV2;GLI3;IGF1R;FOXP1;SYNE1;GLI2;PARD3B;TGFBR3;EDNRB;ATXN1;SETBP1;ZFPM2;PLCB1;TNS3;NCKAP5;TEAD1;CREB5* |
| SOX5 | 24/299 | 8.103927312947807E-15 | *NLGN1;TSHZ2;EBF1;FMN1;ANK3;NAV2;GLI3;IGF1R;FOXP1;SYNE1;PARD3B;TGFBR3;EDNRB;PDZD2;ATXN1;GNG2;SETBP1;ZFPM2;PLCB1;TNS3;NCKAP5;SRGAP1;TEAD1;CREB5* |
| ZNF462 | 24/299 | 8.103927312947807E-15 | *VAV3;MACF1;FNDC3B;MTUS1;CBLB;ANK3;NAV2;FBXO32;PTPN13;HSPG2;SYNE2;GLI3;IGF1R;FOXP1;SYNE1;GLI2;TGFBR3;PDZD2;SETBP1;RNF150;TNS3;PHLDB2;SRGAP1;TEAD1* |
| PRDM16 | 23/299 | 8.47829776242948E-14 | *PLEKHG1;GRID1;EBF1;AGAP1;ANK3;FOXN3;NAV2;HSPG2;GLI3;IGF1R;FOXP1;SYNE1;GLI2;PARD3B;TGFBR3;PDZD2;ATXN1;ZFPM2;PLCB1;TNS3;NCKAP5;TEAD1;CREB5* |
| FOXN3 | 22/299 | 6.870269763826198E-13 | *VAV3;MACF1;DOCK9;FNDC3B;EBF1;MTUS1;AGAP1;CBLB;ANK3;BAZ2B;NAV2;FBXO32;SYNE2;IGF1R;FOXP1;SYNE1;TGFBR3;ATXN1;SETBP1;SSBP2;TNS3;TEAD1* |
| ZNF236 | 22/299 | 6.870269763826198E-13 | *MACF1;EPB41;DOCK9;SFMBT2;FNDC3B;AGAP1;BAZ2B;FOXN3;NAV2;SYNE2;IGF1R;FOXP1;SYNE1;SLC4A7;ZNF609;ATXN1;FCHSD2;SETBP1;ZNF516;PLCB1;TNS3;TEAD1* |
| ZNF827 | 22/299 | 6.870269763826198E-13 | *VAV3;MACF1;FNDC3B;MTUS1;CBLB;ANK3;BAZ2B;FOXN3;NAV2;FBXO32;IGF1R;FOXP1;SYNE1;TGFBR3;INPP4B;ARHGAP32;ATXN1;SETBP1;RNF150;TNS3;PHLDB2;SRGAP1* |
| ADAMTS | 21/299 | 4.42925115192 | *PLEKHG1;COL27A1;TSHZ2;AGAP1;ANK3;NAV2;RUNX2;IGF1R;FOX* |

| | | | |
|---|---|---|---|
| 17 | | 3132E-12 | *P1;SYNE1;GLI2;PARD3B;TGFBR3;PDZD2;ATXN1;SETBP1;PLCB1;TNS3;NCKAP5;TEAD1;CREB5* |
| BACH2 | 21/299 | 4.42925115192 3132E-12 | *RIPOR2;PLEKHG1;DOCK9;FNDC3B;EBF1;CBLB;ANK3;FRMD4B;FOXN3;NAV2;FBXO32;IGF1R;FOXP1;SYNE1;TGFBR3;ATXN1;SETBP1;PLCB1;TNS3;TEAD1;CREB5* |
| RFX3 | 21/299 | 4.42925115192 3132E-12 | *VAV3;TSHZ2;CBLB;ANK3;BAZ2B;FOXN3;NAV2;PTPN13;SYNE2;IGF1R;FOXP1;SYNE1;TGFBR3;SLC4A7;ATXN1;GNG2;SETBP1;PLCB1;SSBP2;TNS3;TEAD1* |
| RORA | 21/299 | 4.42925115192 3132E-12 | *VAV3;FNDC3B;EBF1;AGAP1;ANK3;FOXN3;NAV2;FBXO32;RUNX2;SYNE2;IGF1R;FOXP1;SYNE1;TGFBR3;INPP4B;PDZD2;ATXN1;SETBP1;PLCB1;TEAD1;CREB5* |
| ZNF233 | 21/299 | 4.42925115192 3132E-12 | *CSGALNACT1;VAV3;ZNF221;RIPOR2;TSHZ2;CACNA1D;FMN1;ANK3;BAZ2B;FBXO32;PTPN13;SYNE2;SYNE1;SETBP1;ZNF519;SCN8A;RNF150;ZFPM2;PLCB1;SSBP2;NCKAP5* |
| ZNF608 | 21/299 | 4.42925115192 3132E-12 | *MACF1;EBF1;MTUS1;CBLB;ANK3;FOXN3;NAV2;FBXO32;GLI3;IGF1R;FOXP1;SYNE1;GLI2;TGFBR3;ATXN1;SETBP1;SSBP2;TNS3;PHLDB2;TEAD1;CREB5* |
| ZSCAN20 | 21/299 | 4.42925115192 3132E-12 | *MACF1;EPB41;DOCK9;FNDC3B;AGAP1;AZIN2;ANK3;FOXN3;NAV2;CDC42BPA;HSPG2;SYNE2;IGF1R;FOXP1;SYNE1;GLI2;FCHSD2;SETBP1;TNS3;SRGAP1;CREB5* |
| KIAA1549 | 20/299 | 3.54453857478 1262E-11 | *RIPOR2;MACF1;HIP1;MTUS1;ANK3;NAV2;ADCY1;HSPG2;SYNE2;GLI3;IGF1R;SYNE1;ARHGAP32;PDZD2;ATXN1;SETBP1;TNS3;SRGAP1;TEAD1;CREB5* |
| TRPS1 | 20/299 | 3.54453857478 1262E-11 | *VAV3;FNDC3B;EBF1;MTUS1;CBLB;ANK3;NAV2;RUNX2;GLI3;IGF1R;FOXP1;SYNE1;TGFBR3;INPP4B;EDNRB;ATXN1;SETBP1;TNS3;TEAD1;CREB5* |
| ZFAT | 20/299 | 3.54453857478 1262E-11 | *VAV3;MACF1;FNDC3B;AGAP1;CBLB;ANK3;FOXN3;NAV2;RUNX2;SYNE2;IGF1R;FOXP1;PARD3B;INPP4B;ATXN1;SETBP1;PLCB1;TNS3;TEAD1;CREB5* |
| ZFHX3 | 20/299 | 3.54453857478 1262E-11 | *MACF1;FNDC3B;EBF1;MTUS1;AGAP1;ANK3;BAZ2B;FOXN3;NAV2;HSPG2;SYNE2;GLI3;IGF1R;FOXP1;SYNE1;TGFBR3;ATXN1;SETBP1;TNS3;TEAD1* |
| ZNF532 | 20/299 | 3.54453857478 1262E-11 | *MACF1;HIP1;FNDC3B;ANK3;FOXN3;NAV2;FBXO32;PTPN13;SYNE2;GLI3;IGF1R;FOXP1;SYNE1;ATXN1;ZNF827;SETBP1;PLCB1;TNS3;PHLDB2;TEAD1* |
| AFF3 | 19/299 | 2.23965956154 9418E-10 | *VAV3;RIPOR2;EBF1;ANK3;NAV2;IGF1R;FOXP1;SYNE1;GLI2;TGFBR3;INPP4B;PDZD2;ATXN1;SETBP1;RNF150;PLCB1;TNS3;NCKAP5;CREB5* |
| BAZ2B | 19/299 | 2.23965956154 9418E-10 | *IVNS1ABP;MACF1;DOCK9;FNDC3B;MTUS1;CBLB;ANK3;FRMD4B;FOXN3;NAV2;CDC42BPA;PTPN13;SYNE2;IGF1R;FOXP1;SYNE1;SLC4A7;ATXN1;SETBP1* |
| ESRRG | 19/299 | 2.23965956154 9418E-10 | *VAV3;NLGN1;TSHZ2;EBF1;MTUS1;ANK3;NAV2;ADCY1;IGF1R;FOXP1;SYNE1;TGFBR3;EDNRB;ATXN1;ZFPM2;PLCB1;NCKAP5;TEAD1;CREB5* |
| FOXP2 | 19/299 | 2.23965956154 9418E-10 | *NLGN1;SEMA3D;TSHZ2;EBF1;MTUS1;ANK3;NAV2;FBXO32;RUNX2;GLI3;IGF1R;FOXP1;SYNE1;TGFBR3;SETBP1;ZFPM2;PLCB1;NCKAP5;TEAD1* |
| IKZF2 | 19/299 | 2.23965956154 9418E-10 | *VAV3;RIPOR2;CBLB;ANK3;FRMD4B;NAV2;FBXO32;PTPN13;RUNX2;SYNE2;IGF1R;FOXP1;SYNE1;TGFBR3;INPP4B;ATXN1;SETBP1;SSBP2;TNS3* |
| KLF12 | 19/299 | 2.23965956154 9418E-10 | *DOCK9;EBF1;CBLB;ANK3;FOXN3;NAV2;IGF1R;FOXP1;SYNE1;GLI2;TGFBR3;INPP4B;ATXN1;GNG2;SETBP1;PLCB1;TNS3;TEAD1;CREB5* |
| NR3C2 | 19/299 | 2.23965956154 9418E-10 | *VAV3;NLGN1;DOCK9;MTUS1;ANK3;NAV2;FBXO32;IGF1R;FOXP1;SYNE1;TGFBR3;INPP4B;PDZD2;ATXN1;SETBP1;RNF150;ZFPM2;PLCB1;TNS3* |
| SETBP1 | 19/299 | 2.23965956154 9418E-10 | *TSHZ2;FNDC3B;EBF1;CBLB;ANK3;NAV2;SYNE2;GLI3;IGF1R;FOXP1;SYNE1;TGFBR3;INPP4B;PDZD2;ATXN1;RNF150;PLCB1;TNS3;CREB5* |
| ZBTB20 | 19/299 | 2.23965956154 9418E-10 | *MACF1;FNDC3B;EBF1;MTUS1;ANK3;BAZ2B;FOXN3;NAV2;FBXO32;SYNE2;IGF1R;FOXP1;SYNE1;TGFBR3;ATXN1;SETBP1;PLCB1;TNS3;TEAD1* |
| ZNF516 | 19/299 | 2.23965956154 9418E-10 | *VAV3;RBM47;SFMBT2;FNDC3B;FOXN3;NAV2;GAB2;GLI3;IGF1R;FOXP1;SYNE1;GLI2;TGFBR3;ATXN1;SETBP1;MCTP2;TNS3;TEAD1;CREB5* |
| ZNF621 | 19/299 | 2.23965956154 9418E-10 | *ANKRD28;MACF1;HIP1;FNDC3B;MTUS1;CBLB;BAZ2B;NAV2;FBXO32;CDC42BPA;RUNX2;IGF1R;TGFBR3;ATXN1;TFDP2;PPARA;SRGAP1;TEAD1;CREB5* |
| ZNF704 | 19/299 | 2.23965956154 9418E-10 | *VAV3;PLEKHG1;MTUS1;ANK3;FRMD4B;FOXN3;NAV2;FBXO32;IGF1R;SYNE1;TGFBR3;INPP4B;ATXN1;RNF150;SSBP2;TNS3;SRGAP1;TEAD1;CREB5* |
| BCL11B | 18/299 | 1.45732956534 54052E-9 | *VAV3;RIPOR2;DOCK9;EBF1;ANK3;NAV2;SYNE2;IGF1R;FOXP1;SYNE1;TGFBR3;INPP4B;ATXN1;GNG2;SETBP1;CAMK4;PLCB1;TNS3* |
| CHD6 | 18/299 | 1.45732956534 54052E-9 | *MACF1;DOCK9;AGAP1;ANK3;BAZ2B;FOXN3;NAV2;ADCY1;CDC42BPA;SYNE2;IGF1R;FOXP1;SYNE1;ARHGAP32;PDZD2;ATXN1;PLCB1;TNS3* |
| CHD7 | 18/299 | 1.45732956534 | *IVNS1ABP;MACF1;FNDC3B;MTUS1;ANK3;FRMD4B;BAZ2B;FOXN3;NAV2;SYNE2;IGF1R;FOXP1;SYNE1;SLC4A7;ATXN1;SETBP1;TNS3;* |

| | | | |
|---|---|---|---|
| | | 54052E-9 | *TEAD1* |
| GLIS3 | 18/299 | 1.45732956534 54052E-9 | *FNDC3B;EBF1;NAV2;FBXO32;RUNX2;GLI3;IGF1R;FOXP1;SYNE1;GLI2;PARD3B;TGFBR3;ATXN1;TNS3;PHLDB2;SRGAP1;TEAD1;CREB5* |
| HMBOX1 | 18/299 | 1.45732956534 54052E-9 | *MACF1;DOCK9;FNDC3B;MTUS1;CBLB;ANK3;BAZ2B;FOXN3;NAV2;SYNE2;IGF1R;FOXP1;SYNE1;TGFBR3;ATXN1;TNS3;TEAD1;CREB5* |
| PHF21A | 18/299 | 1.45732956534 54052E-9 | *MACF1;SETD5;MBD5;FNDC3B;MTUS1;CBLB;ANK3;BAZ2B;FOXN3;NAV2;SYNE2;IGF1R;FOXP1;ATXN1;FCHSD2;SETBP1;SSBP2;TNS3* |
| PRDM10 | 18/299 | 1.45732956534 54052E-9 | *MACF1;DOCK9;MTUS1;AGAP1;ANK3;BAZ2B;FOXN3;NAV2;RUNX2;SYNE2;IGF1R;FOXP1;SYNE1;ARHGAP32;ATXN1;TNS3;SRGAP1;TEAD1* |
| PRDM5 | 18/299 | 1.45732956534 54052E-9 | *SEMA3D;EBF1;ANK3;FRMD4B;NAV2;GLI3;FOXP1;SYNE1;GLI2;PARD3B;TGFBR3;INPP4B;EDNRB;ZFPM2;TNS3;PHLDB2;TEAD1;CREB5* |
| PROX1 | 18/299 | 1.45732956534 54052E-9 | *TSHZ2;EBF1;ANK3;FRMD4B;NAV2;FBXO32;IGF1R;FOXP1;SYNE1;TGFBR3;EDNRB;SETBP1;ZFPM2;PLCB1;TNS3;NCKAP5;TEAD1;CREB5* |
| SATB1 | 18/299 | 1.45732956534 54052E-9 | *VAV3;RIPOR2;MACF1;CBLB;ANK3;NAV2;FBXO32;SYNE2;IGF1R;FOXP1;SYNE1;TGFBR3;ATXN1;GNG2;SETBP1;PLCB1;SSBP2;TNS3* |
| THRB | 18/299 | 1.45732956534 54052E-9 | *VAV3;EBF1;ANK3;NAV2;ADCY1;FBXO32;RUNX2;IGF1R;FOXP1;SYNE1;TGFBR3;INPP4B;EDNRB;PDZD2;ATXN1;PPARA;PLCB1;TNS3* |
| ZHX3 | 18/299 | 1.45732956534 54052E-9 | *MACF1;HIP1;DOCK9;FNDC3B;MTUS1;AGAP1;ANK3;FOXN3;NAV2;IGF1R;FOXP1;SYNE1;TGFBR3;ATXN1;SETBP1;TNS3;SRGAP1;TEAD1* |
| ZNF618 | 18/299 | 1.45732956534 54052E-9 | *VAV3;HIP1;MTUS1;ANK3;NAV2;RUNX2;SYNE2;IGF1R;FOXP1;SYNE1;TGFBR3;ATXN1;ZNF827;SETBP1;TNS3;PHLDB2;TEAD1;CREB5* |
| ZNF717 | 18/299 | 1.45732956534 54052E-9 | *NLGN1;SEMA3D;MTUS1;AGAP1;FMN1;ANK3;FRMD4B;FBXO32;SYNE2;SYNE1;PDZD2;SETBP1;ZNF519;ZFPM2;SRGAP2;MCTP2;TNS3;NCKAP5* |
| CAMTA1 | 17/299 | 1.00489800691 62705E-8 | *MACF1;DOCK9;AGAP1;ANK3;FOXN3;NAV2;IGF1R;FOXP1;SYNE1;TGFBR3;ATXN1;SETBP1;PLCB1;TNS3;NCKAP5;TEAD1;CREB5* |
| FOXP1 | 17/299 | 1.00489800691 62705E-8 | *VAV3;MACF1;SETD5;FNDC3B;EBF1;MTUS1;ANK3;FOXN3;NAV2;RUNX2;IGF1R;SYNE1;TGFBR3;ATXN1;SETBP1;TNS3;TEAD1* |
| GLI3 | 17/299 | 1.00489800691 62705E-8 | *EBF1;ANK3;NAV2;PTPN13;HSPG2;RUNX2;IGF1R;FOXP1;GLI2;TGFBR3;ATXN1;SETBP1;ZFPM2;PLCB1;TNS3;TEAD1;CREB5* |
| NCOA1 | 17/299 | 1.00489800691 62705E-8 | *MACF1;DOCK9;FNDC3B;CBLB;ANK3;BAZ2B;FOXN3;NAV2;SYNE2;IGF1R;FOXP1;SYNE1;TGFBR3;ATXN1;SETBP1;SSBP2;TNS3* |
| NFIA | 17/299 | 1.00489800691 62705E-8 | *MACF1;FNDC3B;EBF1;MTUS1;ANK3;FOXN3;NAV2;SYNE2;IGF1R;FOXP1;SYNE1;TGFBR3;ATXN1;SETBP1;TNS3;TEAD1;CREB5* |
| PBX1 | 17/299 | 1.00489800691 62705E-8 | *VAV3;EBF1;MTUS1;ANK3;NAV2;FBXO32;SYNE2;IGF1R;FOXP1;SYNE1;TGFBR3;ATXN1;SETBP1;PLCB1;SSBP2;TNS3;TEAD1* |
| PLXNA4 | 17/299 | 1.00489800691 62705E-8 | *RIPOR2;EBF1;ANK3;NAV2;ADCY1;FBXO32;IGF1R;SYNE1;TGFBR3;PDZD2;SETBP1;RNF150;ZFPM2;PLCB1;TNS3;NCKAP5;CREB5* |
| POU6F2 | 17/299 | 1.00489800691 62705E-8 | *NLGN1;GRID1;TSHZ2;EBF1;ANK3;NAV2;ADCY1;GLI3;IGF1R;FOXP1;SYNE1;PDZD2;SETBP1;ZFPM2;PLCB1;NCKAP5;CREB5* |
| PRDM15 | 17/299 | 1.00489800691 62705E-8 | *MACF1;HIP1;FNDC3B;AGAP1;ANK3;BAZ2B;FOXN3;NAV2;SYNE2;IGF1R;FOXP1;SYNE1;ATXN1;SETBP1;TNS3;TEAD1;CREB5* |
| SSH2 | 17/299 | 1.00489800691 62705E-8 | *IVNS1ABP;RIPOR2;MACF1;DOCK9;FNDC3B;CBLB;ANK3;BAZ2B;FOXN3;SYNE2;IGF1R;FOXP1;SYNE1;TGFBR3;ATXN1;FCHSD2;TNS3* |
| ZIM2 | 17/299 | 1.00489800691 62705E-8 | *VAV3;NLGN1;EBF1;MTUS1;ANK3;NAV2;ADCY1;SYNE2;SYNE1;TGFBR3;EDNRB;SETBP1;ZFPM2;PLCB1;NCKAP5;CNTNAP4;CREB5* |
| ZNF774 | 17/299 | 1.00489800691 62705E-8 | *PLEKHH2;HIP1;MTUS1;AGAP1;CBLB;FMN1;ANK3;NAV2;GAB2;FBXO32;IGF1R;SYNE1;TGFBR3;ATXN1;SETBP1;TFDP2;CREB5* |
| AFF1 | 16/299 | 6.31458549882 3992E-8 | *MACF1;RBM47;FNDC3B;MTUS1;CBLB;ANK3;BAZ2B;FOXN3;NAV2;SYNE2;IGF1R;FOXP1;TGFBR3;ATXN1;TNS3;TEAD1* |
| CASZ1 | 16/299 | 6.31458549882 3992E-8 | *VAV3;MACF1;RBM47;EBF1;AGAP1;ANK3;NAV2;FBXO32;HSPG2;SYNE2;IGF1R;FOXP1;TGFBR3;PDZD2;SETBP1;TNS3* |
| DEPDC5 | 16/299 | 6.31458549882 3992E-8 | *MACF1;SETD5;DOCK9;MTUS1;AGAP1;ANK3;BAZ2B;BRCA1;NAV2;SYNE2;IGF1R;FOXP1;SYNE1;ATXN1;PLCB1;TNS3* |
| MLLT3 | 16/299 | 6.31458549882 3992E-8 | *VAV3;MTUS1;CBLB;ANK3;FRMD4B;NAV2;IGF1R;FOXP1;SYNE1;TGFBR3;INPP4B;ATXN1;SETBP1;SSBP2;TNS3;TEAD1* |
| NCOA2 | 16/299 | 6.31458549882 3992E-8 | *MACF1;DOCK9;FNDC3B;CBLB;ANK3;BAZ2B;FOXN3;NAV2;SYNE2;IGF1R;FOXP1;SYNE1;TGFBR3;ATXN1;TNS3;TEAD1* |
| NR6A1 | 16/299 | 6.31458549882 | *VAV3;RBM47;EBF1;PLEKHA5;ANK3;FOXN3;NAV2;ADCY1;RUNX2;I* |

| | | | |
|---|---|---|---|
| | | 3992E-8 | *GF1R;FOXP1;GLI2;TGFBR3;ATXN1;PHLDB2;TEAD1* |
| PRDM2 | 16/299 | 6.31458549882 3992E-8 | *MACF1;FNDC3B;AGAP1;CBLB;ANK3;BAZ2B;FOXN3;NAV2;SYNE2;IGF1R;FOXP1;SYNE1;ATXN1;SETBP1;TNS3;TEAD1* |
| RAPGEF 5 | 16/299 | 6.31458549882 3992E-8 | *VAV3;PLEKHG1;HIP1;DOCK9;MTUS1;ANK3;NAV2;SYNE2;SYNE1;TGFBR3;INPP4B;EDNRB;PDZD2;SETBP1;PLCB1;TNS3* |
| RERE | 16/299 | 6.31458549882 3992E-8 | *MACF1;FNDC3B;AGAP1;ANK3;BAZ2B;FOXN3;NAV2;SYNE2;IGF1R;FOXP1;SYNE1;TGFBR3;ATXN1;SETBP1;TNS3;TEAD1* |
| TFDP2 | 16/299 | 6.31458549882 3992E-8 | *IVNS1ABP;MTUS1;CBLB;ANK3;BAZ2B;FOXN3;BRCA1;NAV2;FBXO32;SYNE2;IGF1R;FOXP1;SYNE1;TGFBR3;ATXN1;SSBP2* |
| TRERF1 | 16/299 | 6.31458549882 3992E-8 | *VAV3;MACF1;HIP1;ANK3;FOXN3;NAV2;FBXO32;RUNX2;SYNE2;IGF1R;FOXP1;SYNE1;TGFBR3;ATXN1;SETBP1;TNS3* |
| ZBTB38 | 16/299 | 6.31458549882 3992E-8 | *MACF1;FNDC3B;MTUS1;ANK3;BAZ2B;FOXN3;NAV2;FBXO32;SYNE2;IGF1R;FOXP1;SYNE1;ATXN1;TNS3;PHLDB2;TEAD1* |
| ZHX2 | 16/299 | 6.31458549882 3992E-8 | *VAV3;FNDC3B;MTUS1;CBLB;ANK3;FOXN3;NAV2;FBXO32;IGF1R;FOXP1;TGFBR3;ATXN1;SETBP1;TNS3;TEAD1;CREB5* |
| ZNF33B | 16/299 | 6.31458549882 3992E-8 | *IVNS1ABP;VAV3;MACF1;RBM47;PLEKHA5;ANK3;BAZ2B;FOXN3;FBXO32;SYNE2;IGF1R;FOXP1;TGFBR3;ADGRG6;SSBP2;TNS3* |
| ZNF391 | 16/299 | 6.31458549882 3992E-8 | *PLEKHG1;PLEKHH2;HIP1;TSHZ2;MTUS1;FMN1;ANK3;BAZ2B;FBXO32;PTPN13;IGF1R;SYNE1;TGFBR3;SCN8A;RNF150;CREB5* |
| ZNF605 | 16/299 | 6.31458549882 3992E-8 | *VAV3;MTUS1;PLEKHA5;FMN1;ANK3;BAZ2B;NAV2;PTPN13;SYNE2;IGF1R;SYNE1;TGFBR3;INPP4B;SCN8A;PLCB1;SRGAP1* |
| ZNF713 | 16/299 | 6.31458549882 3992E-8 | *PLEKHG1;ANK3;BAZ2B;NAV2;FBXO32;FOXP1;SYNE1;ZNF827;SETBP1;TFDP2;PLCB1;SSBP2;TNS3;NCKAP5;SRGAP1;CREB5* |
| ATF7 | 15/299 | 3.73135554171 35327E-7 | *MACF1;FNDC3B;ANK3;FOXN3;NAV2;HSPG2;RUNX2;SYNE2;IGF1R;FOXP1;ATXN1;TNS3;SRGAP1;TEAD1;CREB5* |
| DPF3 | 15/299 | 3.73135554171 35327E-7 | *EBF1;AGAP1;ANK3;FRMD4B;NAV2;FBXO32;SYNE1;TGFBR3;INPP4B;SETBP1;ZFPM2;TNS3;NCKAP5;PHLDB2;CREB5* |
| GTF2IR D2 | 15/299 | 3.73135554171 35327E-7 | *MTUS1;AGAP1;FRMD4B;BAZ2B;FBXO32;HSPG2;IGF1R;FOXP1;SYNE1;TGFBR3;INPP4B;ATXN1;SSBP2;TNS3;SRGAP1* |
| HELZ | 15/299 | 3.73135554171 35327E-7 | *MACF1;FNDC3B;ANK3;BAZ2B;FOXN3;NAV2;CDC42BPA;SYNE2;IGF1R;FOXP1;SYNE1;SLC4A7;ATXN1;TNS3;TEAD1* |
| JAZF1 | 15/299 | 3.73135554171 35327E-7 | *FNDC3B;EBF1;FOXN3;NAV2;IGF1R;FOXP1;SYNE1;TGFBR3;PDZD2;ATXN1;GNG2;SETBP1;PLCB1;TNS3;CREB5* |
| LCORL | 15/299 | 3.73135554171 35327E-7 | *IVNS1ABP;ANK3;BAZ2B;FOXN3;NAV2;SYNE2;IGF1R;FOXP1;SLC4A7;ATXN1;SETBP1;PLCB1;SSBP2;TNS3;TEAD1* |
| MITF | 15/299 | 3.73135554171 35327E-7 | *EBF1;MTUS1;FRMD4B;NAV2;FBXO32;RUNX2;IGF1R;FOXP1;TGFBR3;INPP4B;EDNRB;ATXN1;TNS3;TEAD1;CREB5* |
| NFIB | 15/299 | 3.73135554171 35327E-7 | *MACF1;FNDC3B;EBF1;MTUS1;ANK3;NAV2;SYNE2;IGF1R;FOXP1;TGFBR3;EDNRB;ATXN1;SETBP1;TNS3;TEAD1* |
| PKHD1 | 15/299 | 3.73135554171 35327E-7 | *VAV3;GRID1;AGAP1;FMN1;ANK3;NAV2;SYNE2;GLI3;SYNE1;PARD3B;PDZD2;SETBP1;ZFPM2;NCKAP5;CREB5* |
| POU2F1 | 15/299 | 3.73135554171 35327E-7 | *MACF1;FNDC3B;EBF1;AGAP1;ANK3;BAZ2B;FOXN3;BRCA1;NAV2;CDC42BPA;SYNE2;IGF1R;FOXP1;ATXN1;TEAD1* |
| RREB1 | 15/299 | 3.73135554171 35327E-7 | *MACF1;RBM47;FNDC3B;MTUS1;ANK3;BAZ2B;FOXN3;NAV2;SYNE2;IGF1R;FOXP1;TGFBR3;ATXN1;TNS3;TEAD1* |
| TCF12 | 15/299 | 3.73135554171 35327E-7 | *IVNS1ABP;MACF1;FNDC3B;ANK3;BAZ2B;FOXN3;NAV2;GLI3;IGF1R;FOXP1;SLC4A7;ATXN1;SSBP2;TNS3;TEAD1* |
| TEAD1 | 15/299 | 3.73135554171 35327E-7 | *MACF1;FNDC3B;ANK3;NAV2;CDC42BPA;HSPG2;GLI3;IGF1R;FOXP1;SYNE1;TGFBR3;ATXN1;TNS3;PHLDB2;SRGAP1* |
| TSHZ2 | 15/299 | 3.73135554171 35327E-7 | *PLEKHG1;COL15A1;EBF1;ANK3;NAV2;FBXO32;FOXP1;SYNE1;TGFBR3;EDNRB;PDZD2;ATXN1;SETBP1;TNS3;CREB5* |
| ZBTB44 | 15/299 | 3.73135554171 35327E-7 | *MACF1;FNDC3B;MTUS1;ANK3;BAZ2B;FOXN3;SYNE2;IGF1R;FOXP1;TGFBR3;SLC4A7;ATXN1;TFDP2;TNS3;TEAD1* |
| ZBTB7C | 15/299 | 3.73135554171 35327E-7 | *VAV3;COL15A1;RBM47;TSHZ2;EBF1;FMN1;ANK3;NAV2;ADCY1;GLI2;TGFBR3;PDZD2;ATXN1;TNS3;NCKAP5* |
| ZMAT1 | 15/299 | 3.73135554171 35327E-7 | *VAV3;RIPOR2;MTUS1;ANK3;BAZ2B;NAV2;FBXO32;SYNE2;SYNE1;TGFBR3;INPP4B;EDNRB;PLCB1;SSBP2;TNS3* |
| ZNF135 | 15/299 | 3.73135554171 35327E-7 | *CSGALNACT1;RIPOR2;EBF1;AGAP1;ANK3;NAV2;FBXO32;IGF1R;PDZD2;SETBP1;RNF150;ZFPM2;PLCB1;TNS3;CREB5* |

| | | | |
|---|---|---|---|
| ZNF280D | 15/299 | 3.7313555417135327E-7 | *ANKRD28;MACF1;FNDC3B;MTUS1;PLEKHA5;ANK3;BAZ2B;SYNE2;IGF1R;FOXP1;SYNE1;SLC4A7;ATXN1;CNTRL;SSBP2* |
| ZNF385D | 15/299 | 3.7313555417135327E-7 | *VAV3;NLGN1;SEMA3D;TSHZ2;EBF1;ANK3;NAV2;SYNE1;TGFBR3;INPP4B;SETBP1;RNF150;ZFPM2;PLCB1;NCKAP5* |
| ZSCAN23 | 15/299 | 3.7313555417135327E-7 | *NLGN1;TSHZ2;ANK3;NAV2;PTPN13;FOXP1;SYNE1;PARD3B;CPED1;EDNRB;SETBP1;SCN8A;RNF150;NCKAP5;CREB5* |
| AEBP2 | 14/299 | 1.981029492324671E-6 | *IVNS1ABP;MACF1;FNDC3B;PLEKHA5;ANK3;BAZ2B;FOXN3;NAV2;IGF1R;FOXP1;SLC4A7;ATXN1;TNS3;TEAD1* |
| ARID5B | 14/299 | 1.981029492324671E-6 | *VAV3;FNDC3B;CBLB;ANK3;FOXN3;NAV2;FBXO32;SYNE2;IGF1R;FOXP1;SYNE1;TGFBR3;ATXN1;TNS3* |
| ATXN7 | 14/299 | 1.981029492324671E-6 | *RIPOR2;MACF1;SETD5;FNDC3B;MTUS1;BAZ2B;FOXN3;NAV2;SYNE2;IGF1R;FOXP1;SYNE1;ATXN1;TEAD1* |
| BNC2 | 14/299 | 1.981029492324671E-6 | *COL15A1;EBF1;ANK3;NAV2;RUNX2;GLI3;FOXP1;SYNE1;GLI2;TGFBR3;ZFPM2;TNS3;PHLDB2;CREB5* |
| CHD9 | 14/299 | 1.981029492324671E-6 | *MACF1;FNDC3B;CBLB;ANK3;BAZ2B;FOXN3;NAV2;SYNE2;IGF1R;FOXP1;SYNE1;ATXN1;TNS3;TEAD1* |
| CUX1 | 14/299 | 1.981029492324671E-6 | *MACF1;FNDC3B;AGAP1;ANK3;FOXN3;NAV2;SYNE2;IGF1R;FOXP1;SYNE1;ATXN1;SSBP2;TNS3;TEAD1* |
| ESR1 | 14/299 | 1.981029492324671E-6 | *VAV3;EBF1;ANK3;BRCA1;RUNX2;ESR2;IGF1R;FOXP1;SYNE1;GLI2;TGFBR3;INPP4B;ATXN1;PPARA* |
| FMNL2 | 14/299 | 1.981029492324671E-6 | *MACF1;DOCK9;FNDC3B;MTUS1;ANK3;FRMD4B;NAV2;IGF1R;FOXP1;SYNE1;TGFBR3;TNS3;PHLDB2;TEAD1* |
| HIVEP3 | 14/299 | 1.981029492324671E-6 | *MACF1;FNDC3B;AGAP1;ANK3;NAV2;RUNX2;IGF1R;FOXP1;SYNE1;TGFBR3;ATXN1;SETBP1;TNS3;CREB5* |
| IKZF4 | 14/299 | 1.981029492324671E-6 | *RIPOR2;SFMBT2;TSHZ2;CACNA1D;ANK3;NAV2;IGF1R;FOXP1;SYNE1;TGFBR3;ATXN1;SETBP1;SRGAP1;TEAD1* |
| MBNL2 | 14/299 | 1.981029492324671E-6 | *IVNS1ABP;MACF1;FNDC3B;MTUS1;ANK3;NAV2;FBXO32;IGF1R;FOXP1;SYNE1;TGFBR3;ATXN1;TNS3;TEAD1* |
| NFATC2 | 14/299 | 1.981029492324671E-6 | *RIPOR2;EBF1;ANK3;NAV2;FBXO32;RUNX2;IGF1R;FOXP1;SYNE1;TGFBR3;INPP4B;ATXN1;TNS3;CREB5* |
| PLAG1 | 14/299 | 1.981029492324671E-6 | *VAV3;EBF1;MTUS1;NAV2;FBXO32;RUNX2;IGF1R;FOXP1;TGFBR3;ATXN1;SETBP1;PLCB1;SSBP2;CREB5* |
| PLXNA2 | 14/299 | 1.981029492324671E-6 | *ANK3;NAV2;HSPG2;IGF1R;FOXP1;SYNE1;TGFBR3;PDZD2;ATXN1;SETBP1;PLCB1;TNS3;PHLDB2;TEAD1* |
| POU6F1 | 14/299 | 1.981029492324671E-6 | *EBF1;MTUS1;ANK3;NAV2;ADCY1;FBXO32;HSPG2;FOXP1;SYNE1;TGFBR3;PDZD2;ATXN1;SETBP1;TNS3* |
| RARB | 14/299 | 1.981029492324671E-6 | *EBF1;ANK3;NAV2;FBXO32;RUNX2;GLI3;FOXP1;SYNE1;TGFBR3;EDNRB;PDZD2;PLCB1;TNS3;CREB5* |
| RGS6 | 14/299 | 1.981029492324671E-6 | *VAV3;NLGN1;GRID1;TSHZ2;EBF1;ANK3;NAV2;ADCY1;SYNE1;EDNRB;PDZD2;ZFPM2;PLCB1;NCKAP5* |
| SORBS2 | 14/299 | 1.981029492324671E-6 | *MACF1;MTUS1;ANK3;NAV2;FBXO32;SYNE2;IGF1R;FOXP1;SYNE1;TGFBR3;PDZD2;PLCB1;TNS3;PHLDB2* |
| SP4 | 14/299 | 1.981029492324671E-6 | *IVNS1ABP;EBF1;ANK3;BAZ2B;FOXN3;SYNE2;IGF1R;FOXP1;SYNE1;TGFBR3;SLC4A7;ATXN1;SSBP2;TEAD1* |
| SSH1 | 14/299 | 1.981029492324671E-6 | *MACF1;HIP1;FNDC3B;CBLB;NAV2;FBXO32;HSPG2;SYNE2;IGF1R;FOXP1;TGFBR3;ATXN1;TNS3;TEAD1* |
| ZC3H6 | 14/299 | 1.981029492324671E-6 | *RIPOR2;MTUS1;CBLB;ANK3;BAZ2B;FBXO32;SYNE2;IGF1R;SYNE1;TGFBR3;FBXL20;ATXN1;SETBP1;SSBP2* |
| ZKSCAN2 | 14/299 | 1.981029492324671E-6 | *MACF1;ANK3;NAV2;ADCY1;SYNE2;GLI3;IGF1R;FOXP1;TGFBR3;ATXN1;SETBP1;SCN8A;SRGAP1;CREB5* |
| ZNF208 | 14/299 | 1.981029492324671E-6 | *RIPOR2;NLGN1;SFMBT2;TSHZ2;MTUS1;FMN1;ANK3;SYNE2;SYNE1;TGFBR3;EDNRB;RNF150;ZFPM2;NCKAP5* |
| ZNF221 | 14/299 | 1.981029492324671E-6 | *ZNF320;SEMA3D;MTUS1;FMN1;ANK3;BAZ2B;SYNE2;IGF1R;SYNE1;SETBP1;PLCB1;NCKAP5;SRGAP1;CREB5* |
| ZNF385B | 14/299 | 1.981029492324671E-6 | *VAV3;NLGN1;EBF1;ANK3;NAV2;ADCY1;SYNE1;TMEM100;INPP4B;PDZD2;ZFPM2;PLCB1;TNS3;CREB5* |
| ZNF418 | 14/299 | 1.981029492324671E-6 | *ZNF320;VAV3;RIPOR2;ANK3;NAV2;SYNE2;IGF1R;FOXP1;SYNE1;INPP4B;EDNRB;PDZD2;SETBP1;TNS3* |
| ZNF429 | 14/299 | 1.98102949232 | *ZNF320;VAV3;RIPOR2;HIP1;TSHZ2;FMN1;ANK3;BAZ2B;FOXN3;IGF1R;TGFBR3;SETBP1;ZNF519;PLCB1* |

| | | | |
|---|---|---|---|
| | | 4671E-6 | |
| ZNF506 | 14/299 | 1.98102949232 4671E-6 | *ZNF320;VAV3;RIPOR2;FMN1;ANK3;BAZ2B;SYNE2;FOXP1;SYNE1;TGFBR3;SETBP1;PLCB1;SSBP2;CREB5* |
| ZNF556 | 14/299 | 1.98102949232 4671E-6 | *VAV3;RIPOR2;DOCK9;MTUS1;NAV2;IGF1R;GLI2;TGFBR3;FBXL20;SETBP1;COL4A4;CAMK4;ZFPM2;TNS3* |
| ZNF660 | 14/299 | 1.98102949232 4671E-6 | *VAV3;RIPOR2;HIP1;EBF1;ANK3;RUNX2;FOXP1;GLI2;PDZD2;GNG2;SETBP1;CAMK4;RNF150;CREB5* |
| ZNF98 | 14/299 | 1.98102949232 4671E-6 | *VAV3;ZNF221;NLGN1;SEMA3D;MTUS1;ANK3;SYNE1;PARD3B;TGFBR3;EDNRB;RNF150;PLCB1;TNS3;TEAD1* |
| BBX | 13/299 | 9.83839134079 0593E-6 | *MACF1;FNDC3B;CBLB;ANK3;BAZ2B;NAV2;SYNE2;IGF1R;FOXP1;SLC4A7;ATXN1;TNS3;TEAD1* |
| DACH1 | 13/299 | 9.83839134079 0593E-6 | *VAV3;EBF1;ANK3;IGF1R;FOXP1;TGFBR3;INPP4B;TOX3;EDNRB;ATXN1;SETBP1;PLCB1;NCKAP5* |
| EBF1 | 13/299 | 9.83839134079 0593E-6 | *COL15A1;TSHZ2;NAV2;GLI3;FOXP1;CPED1;TGFBR3;EDNRB;PDZD2;GNG2;SETBP1;TNS3;CREB5* |
| ERG | 13/299 | 9.83839134079 0593E-6 | *CSGALNACT1;COL15A1;EBF1;HSPG2;RUNX2;GLI3;IGF1R;FOXP1;SYNE1;TGFBR3;EDNRB;TNS3;CREB5* |
| ETV6 | 13/299 | 9.83839134079 0593E-6 | *IVNS1ABP;MACF1;FNDC3B;CBLB;FOXN3;NAV2;RUNX2;IGF1R;FOXP1;ATXN1;TNS3;TEAD1;CREB5* |
| GRM6 | 13/299 | 9.83839134079 0593E-6 | *HIP1;EBF1;CACNA1D;FMN1;ANK3;NAV2;ADCY1;PTPN13;HSPG2;IGF1R;SYNE1;GLI2;EDNRB* |
| HIVEP1 | 13/299 | 9.83839134079 0593E-6 | *IVNS1ABP;MACF1;FNDC3B;ANK3;BAZ2B;NAV2;SYNE2;IGF1R;FOXP1;SYNE1;ATXN1;TNS3;TEAD1* |
| HKR1 | 13/299 | 9.83839134079 0593E-6 | *VAV3;MACF1;HIP1;DOCK9;AGAP1;ANK3;BAZ2B;NAV2;IGF1R;FOXP1;INPP4B;SETBP1;PLCB1* |
| HMG20A | 13/299 | 9.83839134079 0593E-6 | *IVNS1ABP;MACF1;FNDC3B;AGAP1;FOXN3;NAV2;IGF1R;FOXP1;TGFBR3;ATXN1;SSBP2;TNS3;TEAD1* |
| MEF2C | 13/299 | 9.83839134079 0593E-6 | *RIPOR2;EBF1;ANK3;IGF1R;FOXP1;SYNE1;TGFBR3;INPP4B;EDNRB;ATXN1;GNG2;PLCB1;TNS3* |
| MEIS1 | 13/299 | 9.83839134079 0593E-6 | *EBF1;NAV2;RUNX2;GLI3;IGF1R;FOXP1;TGFBR3;EDNRB;SETBP1;ZFPM2;SSBP2;TNS3;CREB5* |
| MYT1L | 13/299 | 9.83839134079 0593E-6 | *SETD5;NLGN1;MBD5;CACNA1D;ANK3;NAV2;ADCY1;FOXP1;SYNE1;ATXN1;CAMK4;PLCB1;NCKAP5* |
| NFAT5 | 13/299 | 9.83839134079 0593E-6 | *MACF1;FNDC3B;ANK3;BAZ2B;NAV2;CDC42BPA;SYNE2;IGF1R;FOXP1;SYNE1;TGFBR3;ATXN1;TEAD1* |
| PHF20 | 13/299 | 9.83839134079 0593E-6 | *IVNS1ABP;MACF1;FNDC3B;ANK3;BAZ2B;FOXN3;IGF1R;FOXP1;SLC4A7;ATXN1;SSBP2;TNS3;TEAD1* |
| PLXNC1 | 13/299 | 9.83839134079 0593E-6 | *VAV3;RIPOR2;EBF1;NAV2;RUNX2;SYNE1;TGFBR3;EDNRB;ATXN1;GNG2;SETBP1;TNS3;CREB5* |
| RFX4 | 13/299 | 9.83839134079 0593E-6 | *GRID1;EBF1;ANK3;NAV2;GLI3;GLI2;PARD3B;TGFBR3;TOX3;EDNRB;NCKAP5;TEAD1;CREB5* |
| RUNX2 | 13/299 | 9.83839134079 0593E-6 | *VAV3;FNDC3B;EBF1;NAV2;FBXO32;IGF1R;FOXP1;GLI2;TGFBR3;ATXN1;TNS3;PHLDB2;CREB5* |
| SATB2 | 13/299 | 9.83839134079 0593E-6 | *VAV3;EBF1;ANK3;NAV2;ADCY1;RUNX2;IGF1R;FOXP1;SYNE1;ATXN1;PLCB1;TNS3;SRGAP1* |
| TCF20 | 13/299 | 9.83839134079 0593E-6 | *MACF1;SETD5;DOCK9;AGAP1;ANK3;FOXN3;NAV2;SYNE2;IGF1R;FOXP1;SYNE1;ATXN1;TNS3* |
| TIGD6 | 13/299 | 9.83839134079 0593E-6 | *RBM47;FNDC3B;FRMD4B;SYNE2;IGF1R;FOXP1;TGFBR3;SETBP1;ZNF516;SRGAP2;TNS3;SRGAP1;CREB5* |
| TOX | 13/299 | 9.83839134079 0593E-6 | *VAV3;RIPOR2;EBF1;ANK3;NAV2;FBXO32;FOXP1;TGFBR3;ATXN1;GNG2;SETBP1;PLCB1;TNS3* |
| ZBTB47 | 13/299 | 9.83839134079 0593E-6 | *HIP1;FOXN3;NAV2;FBXO32;HSPG2;RUNX2;IGF1R;FOXP1;SYNE1;TGFBR3;ATXN1;TNS3;CREB5* |
| ZEB1 | 13/299 | 9.83839134079 0593E-6 | *FNDC3B;EBF1;NAV2;RUNX2;IGF1R;FOXP1;SYNE1;TGFBR3;ATXN1;ZFPM2;TNS3;PHLDB2;TEAD1* |
| ZFHX4 | 13/299 | 9.83839134079 0593E-6 | *TSHZ2;EBF1;ANK3;NAV2;GLI3;FOXP1;SYNE1;GLI2;TGFBR3;SETBP1;ZFPM2;PHLDB2;TEAD1* |
| ZNF154 | 13/299 | 9.83839134079 0593E-6 | *CSGALNACT1;VAV3;RIPOR2;TSHZ2;FMN1;ANK3;RUNX2;FOXP1;SYNE1;CPED1;TGFBR3;SETBP1;RNF150* |

| | | | |
|---|---|---|---|
| ZNF229 | 13/299 | 9.83839134079 0593E-6 | *CSGALNACT1;ZNF221;MTUS1;FMN1;ANK3;NAV2;GLI3;SYNE1;EDNRB;PDZD2;TNS3;NCKAP5;CREB5* |
| ZNF423 | 13/299 | 9.83839134079 0593E-6 | *COL15A1;EBF1;ANK3;NAV2;GLI3;FOXP1;GLI2;TGFBR3;EDNRB;SETBP1;ZFPM2;TNS3;CREB5* |
| ZNF438 | 13/299 | 9.83839134079 0593E-6 | *VAV3;RBM47;FNDC3B;BAZ2B;NAV2;GAB2;GLI3;TGFBR3;ATXN1;SETBP1;TNS3;SRGAP1;CREB5* |
| ZNF471 | 13/299 | 9.83839134079 0593E-6 | *TSHZ2;EBF1;ANK3;IGF1R;SYNE1;CPED1;TGFBR3;PDZD2;RNF150;ZFPM2;PLCB1;SSBP2;CREB5* |
| ZNF490 | 13/299 | 9.83839134079 0593E-6 | *VAV3;SETD5;MBD5;CACNA1D;BAZ2B;FOXN3;BRCA1;NAV2;IGF1R;FOXP1;FBXL20;TFDP2;SRGAP1* |
| ZNF493 | 13/299 | 9.83839134079 0593E-6 | *ZNF320;RIPOR2;MACF1;MTUS1;ANK3;BAZ2B;FOXN3;PTPN13;SYNE2;IGF1R;SYNE1;ZNF519;PLCB1* |
| ZNF546 | 13/299 | 9.83839134079 0593E-6 | *ZNF221;TSHZ2;MTUS1;FMN1;BAZ2B;SYNE2;IGF1R;SYNE1;TGFBR3;ATXN1;ZNF827;SETBP1;SRGAP1* |
| ZNF568 | 13/299 | 9.83839134079 0593E-6 | *ZNF320;ZNF221;MTUS1;AGAP1;NAV2;SYNE2;FOXP1;SYNE1;SETBP1;PLCB1;SSBP2;TNS3;NCKAP5* |
| ZNF573 | 13/299 | 9.83839134079 0593E-6 | *ZNF320;VAV3;ZNF221;MACF1;MTUS1;BAZ2B;NAV2;SYNE2;IGF1R;TGFBR3;ZNF827;TNS3;SRGAP1* |
| ZNF609 | 13/299 | 9.83839134079 0593E-6 | *MACF1;HIP1;FNDC3B;ANK3;FOXN3;NAV2;SYNE2;IGF1R;FOXP1;SLC4A7;ATXN1;ZNF827;TNS3* |
| ZNF611 | 13/299 | 9.83839134079 0593E-6 | *ZNF320;MACF1;HIP1;PLEKHA5;BAZ2B;FOXN3;NAV2;SYNE2;IGF1R;FOXP1;TGFBR3;TFDP2;TNS3* |
| ZNF626 | 13/299 | 9.83839134079 0593E-6 | *ZNF320;VAV3;RIPOR2;RBM47;ANK3;FOXP1;CPED1;TGFBR3;INPP4B;ATXN1;ZNF519;PLCB1;CREB5* |
| ZNF709 | 13/299 | 9.83839134079 0593E-6 | *ZNF320;AGAP1;FMN1;ANK3;BAZ2B;NAV2;SYNE2;IGF1R;FOXP1;GNG2;SETBP1;ZNF519;CREB5* |
| ZNF782 | 13/299 | 9.83839134079 0593E-6 | *VAV3;MTUS1;BAZ2B;FBXO32;PTPN13;SYNE2;SYNE1;TGFBR3;ARHGAP32;SETBP1;CNTRL;SSBP2;TNS3* |
| DZIP1 | 12/299 | 4.68947712077 5006E-5 | *TGFBR3;EDNRB;GNG2;SETBP1;DOCK9;EBF1;ANK3;NAV2;PLCB1;PTPN13;SSBP2;SYNE1* |
| EZH1 | 12/299 | 4.68947712077 5006E-5 | *TGFBR3;FBXL20;ATXN1;DOCK9;MTUS1;ANK3;BAZ2B;FBXO32;SSBP2;IGF1R;FOXP1;SYNE1* |
| GLIS1 | 12/299 | 4.68947712077 5006E-5 | *TGFBR3;COL27A1;GRID1;EBF1;NAV2;ZFPM2;PLCB1;NCKAP5;RUNX2;GLI3;FOXP1;GLI2* |
| HIVEP2 | 12/299 | 4.68947712077 5006E-5 | *MACF1;ATXN1;DOCK9;FNDC3B;CBLB;ANK3;NAV2;PLCB1;TNS3;IGF1R;FOXP1;SYNE1* |
| LCOR | 12/299 | 4.68947712077 5006E-5 | *SLC4A7;MACF1;ATXN1;FNDC3B;ANK3;BAZ2B;FOXN3;NAV2;TNS3;TEAD1;IGF1R;FOXP1* |
| MACF1 | 12/299 | 4.68947712077 5006E-5 | *ATXN1;DOCK9;FNDC3B;ANK3;NAV2;CDC42BPA;TNS3;HSPG2;SYNE2;IGF1R;FOXP1;SYNE1* |
| MEF2A | 12/299 | 4.68947712077 5006E-5 | *MACF1;ATXN1;FNDC3B;MTUS1;ANK3;FOXN3;NAV2;TNS3;TEAD1;IGF1R;FOXP1;SYNE1* |
| MTA3 | 12/299 | 4.68947712077 5006E-5 | *IVNS1ABP;RIPOR2;MACF1;RBM47;DOCK9;ANK3;FOXN3;NAV2;TNS3;SYNE2;IGF1R;FOXP1* |
| NR5A2 | 12/299 | 4.68947712077 5006E-5 | *VAV3;TGFBR3;EDNRB;EBF1;ANK3;NAV2;TNS3;TEAD1;RUNX2;FOXP1;GLI2;CREB5* |
| PAX6 | 12/299 | 4.68947712077 5006E-5 | *VAV3;TGFBR3;EDNRB;EBF1;ANK3;NAV2;TEAD1;GLI3;IGF1R;FOXP1;GLI2;CREB5* |
| RAPGEF4 | 12/299 | 4.68947712077 5006E-5 | *VAV3;TGFBR3;RIPOR2;EDNRB;PDZD2;MTUS1;ANK3;NAV2;ADCY1;PLCB1;FOXP1;SYNE1* |
| RFX7 | 12/299 | 4.68947712077 5006E-5 | *IVNS1ABP;SLC4A7;MACF1;SETD5;MBD5;MTUS1;ANK3;BAZ2B;NAV2;TEAD1;IGF1R;FOXP1* |
| TCF4 | 12/299 | 4.68947712077 5006E-5 | *TGFBR3;MACF1;ATXN1;SETBP1;EBF1;ANK3;NAV2;TNS3;RUNX2;IGF1R;FOXP1;SYNE1* |
| TCF7L2 | 12/299 | 4.68947712077 5006E-5 | *TGFBR3;ATXN1;FNDC3B;EBF1;MTUS1;ANK3;NAV2;TNS3;TEAD1;RUNX2;IGF1R;FOXP1* |
| TSHZ1 | 12/299 | 4.68947712077 5006E-5 | *TGFBR3;ATXN1;SETBP1;TSHZ2;EBF1;ANK3;FOXN3;NAV2;FBXO32;TNS3;IGF1R;FOXP1* |
| TUB | 12/299 | 4.68947712077 | *VAV3;NLGN1;GNG2;SCN8A;ANK3;RNF150;NAV2;ADCY1;PLCB1;IGF1R;SYNE1;GLI2* |

| | | | |
|---|---|---|---|
| | | 5006E-5 | |
| ZBTB10 | 12/299 | 4.689477120775006E-5 | IVNS1ABP;TGFBR3;ATXN1;FNDC3B;EBF1;CBLB;NAV2;FBXO32;TNS3;SYNE2;IGF1R;FOXP1 |
| ZEB2 | 12/299 | 4.689477120775006E-5 | TGFBR3;EDNRB;ATXN1;GNG2;FNDC3B;EBF1;NAV2;TNS3;RUNX2;FOXP1;SYNE1;CREB5 |
| ZFHX2 | 12/299 | 4.689477120775006E-5 | ANK3;NAV2;ADCY1;FBXO32;TNS3;HSPG2;SRGAP1;TEAD1;IGF1R;FOXP1;SYNE1;GLI2 |
| ZFP14 | 12/299 | 4.689477120775006E-5 | TGFBR3;RIPOR2;TSHZ2;ANK3;BAZ2B;NAV2;FBXO32;PPARA;PTPN13;SSBP2;SRGAP1;IGF1R |
| ZFP64 | 12/299 | 4.689477120775006E-5 | IVNS1ABP;VAV3;TSHZ2;FNDC3B;AGAP1;ANK3;NAV2;TNS3;IGF1R;FOXP1;GLI2;CREB5 |
| ZFPM2 | 12/299 | 4.689477120775006E-5 | TGFBR3;COL15A1;EDNRB;PDZD2;EBF1;ANK3;RNF150;NAV2;PLCB1;GLI3;SYNE1;CREB5 |
| ZFYVE26 | 12/299 | 4.689477120775006E-5 | MACF1;ATXN1;FNDC3B;MTUS1;CBLB;BAZ2B;FOXN3;NAV2;FBXO32;TNS3;SYNE2;IGF1R |
| ZNF214 | 12/299 | 4.689477120775006E-5 | TGFBR3;ZNF221;PLEKHG1;SCN8A;ZNF516;ANK3;NAV2;PTPN13;TNS3;SYNE2;GLI3;SYNE1 |
| ZNF264 | 12/299 | 4.689477120775006E-5 | VAV3;TGFBR3;FBXL20;MACF1;ARHGAP32;ZNF609;ATXN1;HIP1;FOXN3;IGF1R;FOXP1;CREB5 |
| ZNF343 | 12/299 | 4.689477120775006E-5 | TGFBR3;MACF1;PLEKHG1;HIP1;ZNF516;ANK3;NAV2;TNS3;HSPG2;RUNX2;SYNE2;FOXP1 |
| ZNF362 | 12/299 | 4.689477120775006E-5 | IVNS1ABP;TGFBR3;ATXN1;ZNF827;FNDC3B;FOXN3;NAV2;FBXO32;SSBP2;TNS3;HSPG2;IGF1R |
| ZNF445 | 12/299 | 4.689477120775006E-5 | MACF1;SETD5;ZNF609;HIP1;ZNF827;TFDP2;EPB41;FOXN3;PPARA;CDC42BPA;TEAD1;IGF1R |
| ZNF483 | 12/299 | 4.689477120775006E-5 | TGFBR3;NLGN1;SFMBT2;CAMK4;MTUS1;FMN1;ANK3;ADCY1;FBXO32;PLCB1;SYNE2;SYNE1 |
| ZNF491 | 12/299 | 4.689477120775006E-5 | ZNF320;TGFBR3;FBXL20;RIPOR2;SETBP1;TSHZ2;APBA1;RNF150;PTPN13;NCKAP5;SYNE1;CREB5 |
| ZNF536 | 12/299 | 4.689477120775006E-5 | NLGN1;EDNRB;SETBP1;GRID1;TSHZ2;EBF1;ANK3;ADCY1;ZFPM2;NCKAP5;GLI2;CREB5 |
| ZNF577 | 12/299 | 4.689477120775006E-5 | ZNF320;TGFBR3;RIPOR2;GOLGA8B;TOX3;BAZ2B;FOXN3;FBXO32;SSBP2;IGF1R;FOXP1;SYNE1 |
| ZNF619 | 12/299 | 4.689477120775006E-5 | TGFBR3;MACF1;HIP1;SETBP1;MTUS1;FMN1;BAZ2B;BRCA1;PPARA;TNS3;SYNE2;CREB5 |
| ZNF676 | 12/299 | 4.689477120775006E-5 | CYP39A1;TGFBR3;NLGN1;EDNRB;SEMA3D;TSHZ2;EBF1;MTUS1;FMN1;RNF150;ZFPM2;SYNE1 |
| ZNF678 | 12/299 | 4.689477120775006E-5 | IVNS1ABP;TGFBR3;SLC4A7;MACF1;ZNF519;EPB41;BAZ2B;NAV2;PLCB1;CDC42BPA;SYNE2;IGF1R |
| ZNF718 | 12/299 | 4.689477120775006E-5 | INPP4B;ZNF519;SCN8A;PLEKHA5;FMN1;FOXN3;PLCB1;SSBP2;TNS3;RUNX2;SYNE2;IGF1R |
| ZNF75D | 12/299 | 4.689477120775006E-5 | VAV3;MACF1;ATXN1;SETBP1;ANK3;BAZ2B;FOXN3;FBXO32;PTPN13;SYNE2;SYNE1;CREB5 |
| ZNF781 | 12/299 | 4.689477120775006E-5 | CSGALNACT1;CPED1;TGFBR3;PLEKHH2;EDNRB;GNG2;SETBP1;TSHZ2;EBF1;MTUS1;RNF150;ZFPM2 |
| ZNF783 | 12/299 | 4.689477120775006E-5 | MACF1;RBM47;COL27A1;ATXN1;SETBP1;FNDC3B;AGAP1;CBLB;PLCB1;IGF1R;FOXP1;CREB5 |
| ZNF804B | 12/299 | 4.689477120775006E-5 | INPP4B;NLGN1;PDZD2;SETBP1;GRID1;ANK3;NAV2;ADCY1;ZFPM2;PLCB1;NCKAP5;SYNE1 |
| ZSCAN5A | 12/299 | 4.689477120775006E-5 | VAV3;TGFBR3;RIPOR2;MACF1;FNDC3B;AGAP1;FOXN3;NAV2;PLCB1;TNS3;IGF1R;CREB5 |
| ZXDA | 12/299 | 4.689477120775006E-5 | TGFBR3;ATXN1;SETBP1;MTUS1;CBLB;BAZ2B;FOXN3;RNF150;ZFPM2;FBXO32;SYNE1;CREB5 |
| ARID2 | 11/299 | 2.1506722820361165E-4 | IVNS1ABP;SLC4A7;MACF1;ATXN1;FNDC3B;ANK3;BAZ2B;NAV2;SYNE2;IGF1R;FOXP1 |
| ASH1L | 11/299 | 2.1506722820361165E-4 | MACF1;SETD5;MBD5;ATXN1;ANK3;BAZ2B;CDC42BPA;SYNE2;IGF1R;FOXP1;SYNE1 |
| CLOCK | 11/299 | 2.1506722820361165E-4 | SLC4A7;MACF1;ATXN1;FNDC3B;BAZ2B;PPARA;CDC42BPA;TEAD1;IGF1R;FOXP1;SYNE1 |

| | | | |
|---|---|---|---|
| CUX2 | 11/299 | 2.1506722820361165E-4 | *TOX3;PDZD2;SETBP1;DOCK9;EBF1;ANK3;NAV2;ADCY1;PLCB1;IGF1R;FOXP1* |
| DHX57 | 11/299 | 2.1506722820361165E-4 | *MACF1;ATXN1;DOCK9;FNDC3B;MTUS1;ANK3;BAZ2B;TNS3;SYNE2;FOXP1;SYNE1* |
| EXOC2 | 11/299 | 2.1506722820361165E-4 | *MACF1;ATXN1;DOCK9;FNDC3B;AGAP1;CBLB;NAV2;TNS3;IGF1R;FOXP1;SYNE1* |
| FAM171B | 11/299 | 2.1506722820361165E-4 | *TGFBR3;EDNRB;GNG2;MTUS1;ANK3;RNF150;ADCY1;FBXO32;PLCB1;PTPN13;SYNE1* |
| FOXJ3 | 11/299 | 2.1506722820361165E-4 | *VAV3;MACF1;SETD5;ATXN1;CBLB;FOXN3;SSBP2;TEAD1;SYNE2;IGF1R;FOXP1* |
| FOXK1 | 11/299 | 2.1506722820361165E-4 | *MACF1;ATXN1;FNDC3B;AGAP1;FOXN3;NAV2;TNS3;HSPG2;TEAD1;IGF1R;FOXP1* |
| HLF | 11/299 | 2.1506722820361165E-4 | *TGFBR3;EDNRB;PDZD2;EBF1;ANK3;RNF150;NAV2;ADCY1;FBXO32;PLCB1;SYNE1* |
| KLF3 | 11/299 | 2.1506722820361165E-4 | *TGFBR3;ATXN1;FNDC3B;MTUS1;FRMD4B;FOXN3;NAV2;TNS3;TEAD1;IGF1R;FOXP1* |
| LGR4 | 11/299 | 2.1506722820361165E-4 | *TGFBR3;EDNRB;FNDC3B;MTUS1;ANK3;NAV2;PTPN13;TNS3;SRGAP1;TEAD1;IGF1R* |
| MLXIP | 11/299 | 2.1506722820361165E-4 | *TGFBR3;MACF1;ATXN1;FNDC3B;NAV2;FBXO32;TNS3;HSPG2;TEAD1;IGF1R;FOXP1* |
| NCOR2 | 11/299 | 2.1506722820361165E-4 | *MACF1;ATXN1;AGAP1;ANK3;NAV2;TNS3;HSPG2;TEAD1;IGF1R;FOXP1;SYNE1* |
| NFIX | 11/299 | 2.1506722820361165E-4 | *TGFBR3;ATXN1;FNDC3B;EBF1;ANK3;NAV2;TNS3;HSPG2;TEAD1;IGF1R;FOXP1* |
| NR2C1 | 11/299 | 2.1506722820361165E-4 | *IVNS1ABP;MACF1;ATXN1;CBLB;ANK3;BRCA1;NAV2;TEAD1;SYNE2;IGF1R;FOXP1* |
| PBX3 | 11/299 | 2.1506722820361165E-4 | *IVNS1ABP;TGFBR3;ATXN1;GNG2;EBF1;NAV2;SSBP2;TNS3;GLI3;IGF1R;FOXP1* |
| PGR | 11/299 | 2.1506722820361165E-4 | *VAV3;INPP4B;EDNRB;EBF1;BRCA1;NAV2;ADCY1;PLCB1;RUNX2;IGF1R;SYNE1* |
| PKNOX1 | 11/299 | 2.1506722820361165E-4 | *IVNS1ABP;TGFBR3;ATXN1;FNDC3B;FOXN3;BRCA1;NAV2;TNS3;TEAD1;IGF1R;FOXP1* |
| PROX2 | 11/299 | 2.1506722820361165E-4 | *RIPOR2;PDZD2;ATXN1;MTUS1;FMN1;ANK3;FOXN3;NAV2;SRGAP1;SYNE2;SYNE1* |
| SALL1 | 11/299 | 2.1506722820361165E-4 | *EDNRB;EBF1;NAV2;ZFPM2;TNS3;TEAD1;GLI3;IGF1R;FOXP1;GLI2;CREB5* |
| SCML4 | 11/299 | 2.1506722820361165E-4 | *VAV3;RIPOR2;INPP4B;GNG2;CAMK4;EBF1;ANK3;FBXO32;SYNE2;FOXP1;SYNE1* |
| ST18 | 11/299 | 2.1506722820361165E-4 | *GNG2;EBF1;MTUS1;ANK3;FRMD4B;NAV2;ADCY1;PLCB1;NCKAP5;SYNE1;CREB5* |
| TP73 | 11/299 | 2.1506722820361165E-4 | *VAV3;BRCA1;NAV2;PPARA;HSPG2;TEAD1;RUNX2;GLI3;IGF1R;FOXP1;GLI2* |
| TSHZ3 | 11/299 | 2.1506722820361165E-4 | *TGFBR3;ATXN1;SETBP1;TSHZ2;EBF1;NAV2;ZFPM2;TNS3;GLI3;FOXP1;SYNE1* |
| ZBED3 | 11/299 | 2.1506722820361165E-4 | *VAV3;TGFBR3;ATXN1;HIP1;MTUS1;BRCA1;NAV2;FBXO32;TNS3;IGF1R;CREB5* |
| ZBTB24 | 11/299 | 2.1506722820361165E-4 | *IVNS1ABP;TGFBR3;SLC4A7;RIPOR2;FNDC3B;ANK3;BAZ2B;FOXN3;IGF1R;FOXP1;SYNE1* |
| ZBTB37 | 11/299 | 2.1506722820361165E-4 | *IVNS1ABP;MACF1;ATXN1;HIP1;BAZ2B;FOXN3;NAV2;CDC42BPA;TEAD1;SYNE2;IGF1R* |
| ZNF334 | 11/299 | 2.1506722820361165E-4 | *TGFBR3;RIPOR2;TOX3;TSHZ2;ANK3;BAZ2B;RNF150;NAV2;ADCY1;SYNE2;CREB5* |
| ZNF555 | 11/299 | 2.1506722820361165E-4 | *ZNF320;VAV3;FBXL20;ATXN1;GAB2;FBXO32;SSBP2;IGF1R;FOXP1;SYNE1;CREB5* |
| ZNF585B | 11/299 | 2.1506722820361165E-4 | *ZNF320;VAV3;TGFBR3;ATXN1;ZNF827;MTUS1;BAZ2B;FBXO32;RUNX2;FOXP1;SYNE1* |
| ZNF587 | 11/299 | 2.1506722820361165E-4 | *ZNF320;TGFBR3;MACF1;SETD5;TFDP2;FNDC3B;BAZ2B;FOXN3;NAV2;SYNE2;IGF1R* |
| ZNF596 | 11/299 | 2.15067228203 | *TGFBR3;PLEKHG1;MBD5;SETBP1;MTUS1;CBLB;BAZ2B;FBXO32;PLCB1;PTPN13;SYNE1* |

| | | | |
|---|---|---|---|
| | | 61165E-4 | |
| ZNF599 | 11/299 | 2.15067228203 61165E-4 | *FBXL20;GNG2;SETBP1;FRMD4B;BAZ2B;FOXN3;NAV2;RUNX2;BEND 5;IGF1R;SYNE1* |
| ZNF652 | 11/299 | 2.15067228203 61165E-4 | *VAV3;TGFBR3;ATXN1;FNDC3B;MTUS1;ANK3;BAZ2B;FOXN3;SYNE2 ;IGF1R;FOXP1* |
| ZNF710 | 11/299 | 2.15067228203 61165E-4 | *MACF1;RBM47;ATXN1;ZNF827;ZNF516;FNDC3B;FRMD4B;NAV2;TN S3;IGF1R;FOXP1* |
| ZNF716 | 11/299 | 2.15067228203 61165E-4 | *ZNF320;RBM47;TSHZ2;FNDC3B;CBLB;FMN1;MCTP2;NCKAP5;FOXP 1;CNTNAP4;CREB5* |
| ZNF778 | 11/299 | 2.15067228203 61165E-4 | *MACF1;ATXN1;EPB41;ZNF516;FNDC3B;MTUS1;AGAP1;NAV2;TNS3 ;IGF1R;GLI2* |
| ZNF780 B | 11/299 | 2.15067228203 61165E-4 | *ZNF320;ZNF221;SLC4A7;MACF1;ZNF609;ATXN1;ANK3;BAZ2B;PP ARA;TEAD1;IGF1R* |
| ZNF841 | 11/299 | 2.15067228203 61165E-4 | *ZNF320;ARHGAP32;PLEKHH2;ATXN1;FNDC3B;CBLB;PTPN13;PHLD B2;SYNE2;FOXP1;SYNE1* |
| AHDC1 | 10/299 | 8.79471064698 1942E-4 | *MACF1;SETD5;ATXN1;AGAP1;ANK3;NAV2;TNS3;HSPG2;IGF1R;FO XP1* |
| BCL11A | 10/299 | 8.79471064698 1942E-4 | *VAV3;RIPOR2;SETBP1;EBF1;ANK3;NAV2;PLCB1;TNS3;IGF1R;FO XP1* |
| CHD2 | 10/299 | 8.79471064698 1942E-4 | *MACF1;SETD5;MBD5;ATXN1;FNDC3B;BAZ2B;NAV2;IGF1R;FOXP1; SYNE1* |
| CREB5 | 10/299 | 8.79471064698 1942E-4 | *TGFBR3;RBM47;ATXN1;FNDC3B;EBF1;NAV2;TNS3;RUNX2;IGF1R; FOXP1* |
| DACH2 | 10/299 | 8.79471064698 1942E-4 | *VAV3;RIPOR2;TOX3;NLGN1;GNG2;TSHZ2;EBF1;ADCY1;PLCB1;NC KAP5* |
| ELF2 | 10/299 | 8.79471064698 1942E-4 | *MACF1;ATXN1;FNDC3B;CBLB;BAZ2B;FOXN3;TNS3;IGF1R;FOXP1; CREB5* |
| ETV1 | 10/299 | 8.79471064698 1942E-4 | *VAV3;EDNRB;GNG2;EBF1;ANK3;NAV2;PLCB1;FOXP1;GLI2;CREB5* |
| FOXO3 | 10/299 | 8.79471064698 1942E-4 | *TGFBR3;ATXN1;FNDC3B;CBLB;ANK3;FOXN3;NAV2;TNS3;IGF1R;F OXP1* |
| GLI2 | 10/299 | 8.79471064698 1942E-4 | *TGFBR3;MACF1;EBF1;NAV2;TNS3;HSPG2;TEAD1;RUNX2;GLI3;IG F1R* |
| GON4L | 10/299 | 8.79471064698 1942E-4 | *MACF1;SETD5;DOCK9;ANK3;BAZ2B;NAV2;CDC42BPA;SYNE2;IGF1 R;SYNE1* |
| GTF2IR D2B | 10/299 | 8.79471064698 1942E-4 | *GOLGA8B;MTUS1;STON1;CBLB;ANK3;BAZ2B;FBXO32;PLCB1;SYNE 2;CREB5* |
| HNF4G | 10/299 | 8.79471064698 1942E-4 | *INPP4B;TOX3;RBM47;ANK3;PPARA;NCKAP5;RUNX2;GLI3;GLI2;C REB5* |
| MEIS2 | 10/299 | 8.79471064698 1942E-4 | *TGFBR3;EDNRB;TSHZ2;EBF1;ANK3;NAV2;ZFPM2;TEAD1;GLI3;FO XP1* |
| MINK1 | 10/299 | 8.79471064698 1942E-4 | *IVNS1ABP;MACF1;ATXN1;DOCK9;AGAP1;ANK3;NAV2;TNS3;IGF1R ;SYNE1* |
| NFIC | 10/299 | 8.79471064698 1942E-4 | *TGFBR3;ATXN1;AGAP1;ANK3;NAV2;TNS3;HSPG2;TEAD1;IGF1R;F OXP1* |
| NFXL1 | 10/299 | 8.79471064698 1942E-4 | *SLC4A7;RBM47;FNDC3B;MTUS1;CBLB;BAZ2B;BRCA1;TNS3;FOXP1 ;CREB5* |
| NR2E3 | 10/299 | 8.79471064698 1942E-4 | *EBF1;NAV2;ADCY1;PPARA;TNS3;TEAD1;RUNX2;ESR2;FOXP1;GLI 2* |
| ONECUT 2 | 10/299 | 8.79471064698 1942E-4 | *VAV3;TOX3;ATXN1;EBF1;NAV2;ADCY1;SRGAP1;TEAD1;IGF1R;CR EB5* |
| PKNOX2 | 10/299 | 8.79471064698 1942E-4 | *TGFBR3;EDNRB;SETBP1;EBF1;ANK3;NAV2;PLCB1;TNS3;SYNE1;G LI2* |
| PRDM6 | 10/299 | 8.79471064698 1942E-4 | *COL15A1;EDNRB;TSHZ2;EBF1;NAV2;ZFPM2;NCKAP5;GLI3;GLI2; CREB5* |
| PRDM8 | 10/299 | 8.79471064698 1942E-4 | *TGFBR3;PDZD2;GNG2;EBF1;NAV2;ZFPM2;PLCB1;NCKAP5;RUNX2; CREB5* |
| RAG1 | 10/299 | 8.79471064698 1942E-4 | *VAV3;RIPOR2;INPP4B;CAMK4;EBF1;MCTP2;PHLDB2;IGF1R;SYNE 1;CREB5* |

| | | | |
|---|---|---|---|
| RBM20 | 10/299 | 8.79471064698 1942E-4 | *TGFBR3;EDNRB;MTUS1;ANK3;NAV2;FBXO32;PTPN13;TNS3;NCKAP5;IGF1R* |
| RGS9 | 10/299 | 8.79471064698 1942E-4 | *TGFBR3;EDNRB;PDZD2;GNG2;CAMK4;ANK3;NAV2;FBXO32;PLCB1;SYNE1* |
| RORB | 10/299 | 8.79471064698 1942E-4 | *NLGN1;EDNRB;PDZD2;SCN8A;EBF1;ANK3;ADCY1;ZFPM2;PLCB1;GLI3* |
| SALL2 | 10/299 | 8.79471064698 1942E-4 | *VAV3;TGFBR3;TOX3;EDNRB;ANK3;NAV2;FBXO32;PTPN13;GLI3;GLI2* |
| SMAD3 | 10/299 | 8.79471064698 1942E-4 | *TGFBR3;MACF1;ATXN1;FNDC3B;BRCA1;NAV2;TNS3;RUNX2;IGF1R;FOXP1* |
| TP63 | 10/299 | 8.79471064698 1942E-4 | *VAV3;TGFBR3;INPP4B;EBF1;BRCA1;NAV2;FBXO32;RUNX2;IGF1R;FOXP1* |
| YEATS2 | 10/299 | 8.79471064698 1942E-4 | *IVNS1ABP;SLC4A7;MACF1;SETD5;FNDC3B;AGAP1;ANK3;NAV2;TNS3;SYNE2* |
| ZBTB4 | 10/299 | 8.79471064698 1942E-4 | *TGFBR3;ATXN1;SETBP1;FNDC3B;FOXN3;NAV2;FBXO32;TNS3;FOXP1;SYNE1* |
| ZBTB40 | 10/299 | 8.79471064698 1942E-4 | *MACF1;ATXN1;DOCK9;NAV2;TNS3;SYNE2;IGF1R;FOXP1;SYNE1;CREB5* |
| ZC3H7B | 10/299 | 8.79471064698 1942E-4 | *MACF1;AGAP1;ANK3;NAV2;CDC42BPA;TNS3;HSPG2;IGF1R;FOXP1;SYNE1* |
| ZCCHC6 | 10/299 | 8.79471064698 1942E-4 | *IVNS1ABP;SLC4A7;MACF1;ATXN1;FNDC3B;CBLB;BAZ2B;TNS3;SYNE2;FOXP1* |
| ZFP41 | 10/299 | 8.79471064698 1942E-4 | *COL27A1;ATXN1;HIP1;AGAP1;BRCA1;NAV2;FBXO32;IGF1R;FOXP1;GLI2* |
| ZHX1 | 10/299 | 8.79471064698 1942E-4 | *IVNS1ABP;TGFBR3;SLC4A7;MACF1;ATXN1;MTUS1;FBXO32;TEAD1;IGF1R;FOXP1* |
| ZKSCAN 3 | 10/299 | 8.79471064698 1942E-4 | *IVNS1ABP;MACF1;TFDP2;MTUS1;ANK3;BAZ2B;SSBP2;SYNE2;IGF1R;SYNE1* |
| ZNF117 | 10/299 | 8.79471064698 1942E-4 | *VAV3;TGFBR3;HIP1;MTUS1;ANK3;BAZ2B;FBXO32;SYNE2;IGF1R;CREB5* |
| ZNF133 | 10/299 | 8.79471064698 1942E-4 | *IVNS1ABP;TGFBR3;ATXN1;ZNF516;CBLB;GTF2IRD1;BAZ2B;TNS3;IGF1R;CREB5* |
| ZNF141 | 10/299 | 8.79471064698 1942E-4 | *ZNF320;VAV3;INPP4B;SETBP1;ZNF519;PLEKHA5;ANK3;BAZ2B;MCTP2;SYNE2* |
| ZNF254 | 10/299 | 8.79471064698 1942E-4 | *IVNS1ABP;SLC4A7;MACF1;ANK3;BAZ2B;FOXN3;NAV2;SSBP2;IGF1R;FOXP1* |
| ZNF292 | 10/299 | 8.79471064698 1942E-4 | *IVNS1ABP;SLC4A7;MACF1;FNDC3B;ANK3;BAZ2B;NAV2;CDC42BPA;SYNE2;IGF1R* |
| ZNF341 | 10/299 | 8.79471064698 1942E-4 | *TGFBR3;FCHSD2;FNDC3B;NAV2;FBXO32;TNS3;SRGAP1;IGF1R;FOXP1;CREB5* |
| ZNF365 | 10/299 | 8.79471064698 1942E-4 | *VAV3;TGFBR3;INPP4B;ATXN1;ANK3;NAV2;ADCY1;PLCB1;SYNE1;CREB5* |
| ZNF37A | 10/299 | 8.79471064698 1942E-4 | *TGFBR3;ATXN1;MTUS1;ANK3;BAZ2B;CDC42BPA;TEAD1;SYNE2;IGF1R;FOXP1* |
| ZNF431 | 10/299 | 8.79471064698 1942E-4 | *ZNF320;TGFBR3;SLC4A7;MACF1;ZNF519;TFDP2;PLEKHA5;BAZ2B;SYNE2;IGF1R* |
| ZNF441 | 10/299 | 8.79471064698 1942E-4 | *TGFBR3;RIPOR2;INPP4B;GNG2;SETBP1;TSHZ2;MTUS1;FBXO32;SYNE1;CREB5* |
| ZNF484 | 10/299 | 8.79471064698 1942E-4 | *ZNF221;SLC4A7;RIPOR2;ZNF827;CNTRL;CBLB;BAZ2B;IGF1R;SYNE1;CREB5* |
| ZNF514 | 10/299 | 8.79471064698 1942E-4 | *TGFBR3;INPP4B;GOLGA8B;HIP1;SETBP1;ANK3;BAZ2B;NAV2;IGF1R;FOXP1* |
| ZNF528 | 10/299 | 8.79471064698 1942E-4 | *ZNF320;CSGALNACT1;ADGRG6;ANK3;NAV2;ADCY1;FBXO32;PTPN13;SRGAP1;SYNE1* |
| ZNF677 | 10/299 | 8.79471064698 1942E-4 | *ZNF320;CPED1;TGFBR3;RIPOR2;EDNRB;PDZD2;SETBP1;ZFPM2;SYNE1;CREB5* |
| ZNF699 | 10/299 | 8.79471064698 1942E-4 | *TGFBR3;SETBP1;FNDC3B;CBLB;ANK3;FRMD4B;NAV2;RUNX2;SYNE1;CREB5* |
| ZNF804 | 10/299 | 8.79471064698 | *CPED1;NLGN1;EDNRB;SETBP1;CAMK4;ANK3;ZFPM2;PLCB1;NCKAP5;SYNE1* |

| | | | |
|---|---|---|---|
| A | | 1942E-4 | |
| ZNF839 | 10/299 | 8.794710646981942E-4 | *IVNS1ABP;MACF1;SETBP1;FNDC3B;BAZ2B;FOXN3;TNS3;SRGAP1;SYNE2;FOXP1* |
| ZNF91 | 10/299 | 8.794710646981942E-4 | *TGFBR3;SETBP1;FNDC3B;MTUS1;ANK3;BAZ2B;SSBP2;TNS3;SYNE2;SYNE1* |
| ARNT | 9/299 | 0.0032494027861537833 | *MACF1;ATXN1;CBLB;BRCA1;PPARA;TEAD1;RUNX2;IGF1R;FOXP1* |
| ARNT2 | 9/299 | 0.0032494027861537833 | *EDNRB;ANK3;NAV2;ADCY1;PLCB1;TNS3;GLI3;IGF1R;GLI2* |
| CREBL2 | 9/299 | 0.0032494027861537833 | *TGFBR3;ATXN1;DOCK9;MTUS1;FOXN3;FBXO32;TNS3;IGF1R;FOXP1* |
| CRX | 9/299 | 0.0032494027861537833 | *EBF1;BRCA1;PPARA;HSPG2;TEAD1;RUNX2;ESR2;GLI3;GLI2* |
| DMTF1 | 9/299 | 0.0032494027861537833 | *IVNS1ABP;VAV3;SLC4A7;MACF1;CBLB;BAZ2B;NAV2;SSBP2;SYNE2* |
| DZIP1L | 9/299 | 0.0032494027861537833 | *TGFBR3;ANK3;NAV2;HSPG2;PHLDB2;TEAD1;GLI3;SYNE1;GLI2* |
| ELK4 | 9/299 | 0.0032494027861537833 | *IVNS1ABP;MACF1;ATXN1;FNDC3B;FOXN3;CDC42BPA;TEAD1;IGF1R;FOXP1* |
| FOXJ2 | 9/299 | 0.0032494027861537833 | *IVNS1ABP;ATXN1;HIP1;FNDC3B;CBLB;FOXN3;NAV2;TNS3;FOXP1* |
| FOXK2 | 9/299 | 0.0032494027861537833 | *MACF1;ATXN1;FNDC3B;AGAP1;BRCA1;TNS3;TEAD1;IGF1R;FOXP1* |
| FOXO1 | 9/299 | 0.0032494027861537833 | *TGFBR3;ATXN1;EBF1;FOXN3;FBXO32;TNS3;TEAD1;IGF1R;FOXP1* |
| FOXP4 | 9/299 | 0.0032494027861537833 | *ATXN1;AGAP1;NAV2;TNS3;HSPG2;TEAD1;IGF1R;FOXP1;GLI2* |
| GPR155 | 9/299 | 0.0032494027861537833 | *TGFBR3;RIPOR2;ATXN1;GNG2;DOCK9;ANK3;FBXO32;PLCB1;SYNE1* |
| HBP1 | 9/299 | 0.0032494027861537833 | *IVNS1ABP;ATXN1;FNDC3B;CBLB;BAZ2B;FOXN3;FBXO32;SSBP2;IGF1R* |
| HIF3A | 9/299 | 0.0032494027861537833 | *TGFBR3;EDNRB;PDZD2;DOCK9;NAV2;TNS3;GLI3;GLI2;CREB5* |
| LMX1B | 9/299 | 0.0032494027861537833 | *GRID1;EBF1;ANK3;NAV2;ADCY1;TEAD1;GLI3;IGF1R;GLI2* |
| NFATC1 | 9/299 | 0.0032494027861537833 | *TGFBR3;RIPOR2;ATXN1;EBF1;NAV2;TNS3;RUNX2;IGF1R;FOXP1* |
| NFATC3 | 9/299 | 0.0032494027861537833 | *MACF1;ATXN1;FNDC3B;CBLB;FOXN3;SSBP2;SYNE2;IGF1R;FOXP1* |
| NFRKB | 9/299 | 0.0032494027861537833 | *IVNS1ABP;MACF1;SETD5;FNDC3B;BAZ2B;BRCA1;NAV2;SYNE2;FOXP1* |
| NR2C2 | 9/299 | 0.0032494027861537833 | *MACF1;SETD5;ATXN1;ANK3;BAZ2B;FOXN3;TEAD1;IGF1R;FOXP1* |
| NR3C1 | 9/299 | 0.0032494027861537833 | *MACF1;ATXN1;FNDC3B;CBLB;TNS3;SYNE2;IGF1R;FOXP1;SYNE1* |
| PKHD1L1 | 9/299 | 0.0032494027861537833 | *EDNRB;PDZD2;SEMA3D;EBF1;MTUS1;ANK3;ZFPM2;SYNE2;SYNE1* |
| PLXNB1 | 9/299 | 0.0032494027861537833 | *TGFBR3;DOCK9;ANK3;NAV2;PTPN13;TNS3;HSPG2;SYNE2;IGF1R* |
| PRDM9 | 9/299 | 0.0032494027861537833 | *VAV3;INPP4B;COL15A1;PDZD2;CBLB;NAV2;TNS3;SYNE2;CREB5* |
| REST | 9/299 | 0.0032494027861537833 | *MACF1;ATXN1;FNDC3B;BAZ2B;FOXN3;BRCA1;TEAD1;IGF1R;FOXP1* |
| RFX2 | 9/299 | 0.0032494027861537833 | *TGFBR3;NAV2;TNS3;HSPG2;TEAD1;GLI3;FOXP1;GLI2;CREB5* |
| SALL3 | 9/299 | 0.0032494027861537833 | *TOX3;EDNRB;EBF1;ANK3;NAV2;TNS3;GLI3;FOXP1;GLI2* |
| SIM1 | 9/299 | 0.0032494027861537833 | *TGFBR3;EDNRB;SFMBT2;EBF1;ANK3;ZFPM2;TNS3;IGF1R;GLI2* |

| | | | |
|---|---|---|---|
| SLC4A10 | 9/299 | 0.0032494027861537833 | *NLGN1;SCN8A;CAMK4;FMN1;ANK3;ADCY1;PLCB1;NCKAP5;SYNE1* |
| SMAD5 | 9/299 | 0.0032494027861537833 | *TGFBR3;SLC4A7;ATXN1;FNDC3B;BAZ2B;FOXN3;TEAD1;IGF1R;FOXP1* |
| TBX18 | 9/299 | 0.0032494027861537833 | *CPED1;TGFBR3;SEMA3D;EBF1;ZFPM2;TNS3;PHLDB2;RUNX2;GLI2* |
| THRA | 9/299 | 0.0032494027861537833 | *TGFBR3;ATXN1;ANK3;NAV2;PPARA;TNS3;IGF1R;FOXP1;SYNE1* |
| XPA | 9/299 | 0.0032494027861537833 | *TGFBR3;ATXN1;SETBP1;TFDP2;MTUS1;ANK3;BRCA1;IGF1R;SYNE1* |
| ZBTB34 | 9/299 | 0.0032494027861537833 | *ARHGAP32;ATXN1;FNDC3B;BAZ2B;FOXN3;TEAD1;IGF1R;FOXP1;CREB5* |
| ZCCHC11 | 9/299 | 0.0032494027861537833 | *IVNS1ABP;SLC4A7;MACF1;CNTRL;CBLB;BAZ2B;CDC42BPA;SYNE2;FOXP1* |
| ZKSCAN1 | 9/299 | 0.0032494027861537833 | *MACF1;ATXN1;FNDC3B;ANK3;BAZ2B;FOXN3;TEAD1;IGF1R;FOXP1* |
| ZNF132 | 9/299 | 0.0032494027861537833 | *CPED1;RIPOR2;PLEKHG1;SCN8A;ANK3;RNF150;PPARA;SYNE1;CREB5* |
| ZNF160 | 9/299 | 0.0032494027861537833 | *ZNF320;MACF1;MTUS1;CBLB;ANK3;BAZ2B;NAV2;IGF1R;FOXP1* |
| ZNF19 | 9/299 | 0.0032494027861537833 | *ZNF221;ATXN1;MTUS1;NAV2;FBXO32;TNS3;NCKAP5;FOXP1;SYNE1* |
| ZNF211 | 9/299 | 0.0032494027861537833 | *ZNF320;TGFBR3;MACF1;CBLB;NAV2;GAB2;SSBP2;SYNE2;CREB5* |
| ZNF248 | 9/299 | 0.0032494027861537833 | *TGFBR3;MACF1;ATXN1;SETBP1;ANK3;BAZ2B;FOXN3;SSBP2;SYNE2* |
| ZNF284 | 9/299 | 0.0032494027861537833 | *ZNF320;VAV3;TGFBR3;ZNF221;SETBP1;FBXO32;SSBP2;SRGAP1;CREB5* |
| ZNF3 | 9/299 | 0.0032494027861537833 | *IVNS1ABP;TFDP2;CBLB;BAZ2B;FOXN3;NAV2;FBXO32;TEAD1;FOXP1* |
| ZNF300 | 9/299 | 0.0032494027861537833 | *NLGN1;PLEKHH2;ADGRG6;EBF1;PLCB1;PTPN13;TNS3;PHLDB2;CREB5* |
| ZNF318 | 9/299 | 0.0032494027861537833 | *MACF1;ATXN1;CBLB;ANK3;BAZ2B;NAV2;SYNE2;IGF1R;SYNE1* |
| ZNF333 | 9/299 | 0.0032494027861537833 | *FBXL20;ATXN1;BAZ2B;FOXN3;NAV2;TNS3;RUNX2;FOXP1;SYNE1* |
| ZNF345 | 9/299 | 0.0032494027861537833 | *ZNF320;RIPOR2;ZNF519;PLEKHA5;BAZ2B;FBXO32;SSBP2;SYNE2;IGF1R* |
| ZNF366 | 9/299 | 0.0032494027861537833 | *CSGALNACT1;CPED1;TGFBR3;COL15A1;RBM47;EDNRB;TSHZ2;EBF1;TNS3* |
| ZNF382 | 9/299 | 0.0032494027861537833 | *ZNF320;ZNF221;RIPOR2;DOCK9;CAMK4;ANK3;NAV2;SSBP2;CREB5* |
| ZNF398 | 9/299 | 0.0032494027861537833 | *RBM47;ATXN1;TFDP2;EPB41;FOXN3;ADCY1;SYNE2;IGF1R;FOXP1* |
| ZNF41 | 9/299 | 0.0032494027861537833 | *SLC4A7;ATXN1;ZNF827;DOCK9;MTUS1;PPARA;TNS3;RUNX2;SYNE1* |
| ZNF415 | 9/299 | 0.0032494027861537833 | *CSGALNACT1;TGFBR3;EDNRB;MTUS1;ANK3;ZFPM2;PLCB1;SSBP2;CREB5* |
| ZNF43 | 9/299 | 0.0032494027861537833 | *ZNF320;VAV3;MTUS1;ANK3;BAZ2B;PTPN13;SSBP2;FOXP1;SYNE1* |
| ZNF518B | 9/299 | 0.0032494027861537833 | *SLC4A7;RBM47;GNG2;SETBP1;BAZ2B;PTPN13;PHLDB2;TEAD1;SYNE1* |
| ZNF548 | 9/299 | 0.0032494027861537833 | *ZNF320;TGFBR3;FBXL20;DOCK9;PLEKHA5;CBLB;FOXN3;IGF1R;SYNE1* |
| ZNF563 | 9/299 | 0.0032494027861537833 | *VAV3;TGFBR3;RIPOR2;INPP4B;ZNF827;MTUS1;ANK3;RNF150;NAV2* |
| ZNF585A | 9/299 | 0.0032494027861537833 | *ZNF320;VAV3;RIPOR2;ATXN1;MTUS1;CBLB;BAZ2B;FBXO32;IGF1R* |
| ZNF648 | 9/299 | 0.00324940278 | *GRID1;EBF1;AGAP1;ANK3;NAV2;TNS3;NCKAP5;GLI2;CREB5* |

| | | | |
|---|---|---|---|
| | | 61537833 | |
| ZNF700 | 9/299 | 0.00324940278 61537833 | *ZNF320;IVNS1ABP;SLC4A7;GOLGA8B;MACF1;CNTRL;FNDC3B;BAZ2B;FOXP1* |
| ZNF740 | 9/299 | 0.00324940278 61537833 | *IVNS1ABP;FBXL20;MACF1;NAV2;PPARA;TNS3;TEAD1;IGF1R;FOXP1* |
| ZNF8 | 9/299 | 0.00324940278 61537833 | *FBXL20;ATXN1;HIP1;SETBP1;AGAP1;NAV2;IGF1R;FOXP1;SYNE1* |
| ZNF805 | 9/299 | 0.00324940278 61537833 | *IVNS1ABP;FBXL20;MACF1;ATXN1;FOXN3;FBXO32;SYNE2;IGF1R;SYNE1* |
| ZNF808 | 9/299 | 0.00324940278 61537833 | *ZNF320;TGFBR3;DOCK9;AGAP1;NAV2;TNS3;SRGAP1;IGF1R;FOXP1* |
| ZNF83 | 9/299 | 0.00324940278 61537833 | *ZNF320;IVNS1ABP;GOLGA8B;MTUS1;CBLB;BAZ2B;SSBP2;SYNE2;CREB5* |
| ZNF99 | 9/299 | 0.00324940278 61537833 | *TGFBR3;RIPOR2;PDZD2;MTUS1;FMN1;ZFPM2;TNS3;NCKAP5;SYNE1* |
| ZSCAN2 | 9/299 | 0.00324940278 61537833 | *FBXL20;ATXN1;ANK3;BAZ2B;FOXN3;NAV2;FBXO32;TNS3;FOXP1* |
| ZXDC | 9/299 | 0.00324940278 61537833 | *MACF1;ATXN1;FNDC3B;AGAP1;CBLB;BAZ2B;NAV2;IGF1R;FOXP1* |
| AFF4 | 8/299 | 0.01084737792 5196319 | *SLC4A7;MACF1;ATXN1;FNDC3B;BAZ2B;TEAD1;IGF1R;FOXP1* |
| ALX4 | 8/299 | 0.01084737792 5196319 | *TGFBR3;COL15A1;EBF1;NAV2;TNS3;RUNX2;GLI3;GLI2* |
| AR | 8/299 | 0.01084737792 5196319 | *TGFBR3;EDNRB;EBF1;ANK3;BRCA1;PPARA;RUNX2;IGF1R* |
| ATF6 | 8/299 | 0.01084737792 5196319 | *IVNS1ABP;VAV3;MACF1;ATXN1;FNDC3B;CBLB;IGF1R;FOXP1* |
| BAZ2A | 8/299 | 0.01084737792 5196319 | *MACF1;SETD5;ATXN1;BAZ2B;NAV2;SYNE2;IGF1R;FOXP1* |
| CREB3L2 | 8/299 | 0.01084737792 5196319 | *TGFBR3;ATXN1;HIP1;FNDC3B;NAV2;TNS3;TEAD1;IGF1R* |
| EBF3 | 8/299 | 0.01084737792 5196319 | *TGFBR3;COL15A1;EDNRB;GNG2;TSHZ2;EBF1;NAV2;ZFPM2* |
| EEA1 | 8/299 | 0.01084737792 5196319 | *SLC4A7;MACF1;ATXN1;FNDC3B;BAZ2B;CDC42BPA;TEAD1;IGF1R* |
| EP400 | 8/299 | 0.01084737792 5196319 | *MACF1;SETD5;ANK3;NAV2;SYNE2;IGF1R;FOXP1;SYNE1* |
| ESRRB | 8/299 | 0.01084737792 5196319 | *EBF1;ANK3;NAV2;PPARA;TNS3;TEAD1;FOXP1;GLI2* |
| ETS1 | 8/299 | 0.01084737792 5196319 | *TGFBR3;FNDC3B;EBF1;TNS3;PHLDB2;RUNX2;IGF1R;FOXP1* |
| FOXN2 | 8/299 | 0.01084737792 5196319 | *IVNS1ABP;VAV3;SLC4A7;ATXN1;FNDC3B;FOXN3;TEAD1;FOXP1* |
| GATA4 | 8/299 | 0.01084737792 5196319 | *RBM47;EBF1;NAV2;ZFPM2;RUNX2;IGF1R;FOXP1;GLI2* |
| GPATCH8 | 8/299 | 0.01084737792 5196319 | *MACF1;SETD5;ATXN1;MTUS1;BAZ2B;SYNE2;IGF1R;FOXP1* |
| GRHL2 | 8/299 | 0.01084737792 5196319 | *VAV3;TOX3;RBM47;ANK3;NAV2;TNS3;IGF1R;FOXP1* |
| HAND2 | 8/299 | 0.01084737792 5196319 | *TMEM100;TGFBR3;COL15A1;EDNRB;EBF1;ZFPM2;RUNX2;GLI2* |
| HIC2 | 8/299 | 0.01084737792 5196319 | *MACF1;ATXN1;NAV2;TNS3;TEAD1;IGF1R;FOXP1;CREB5* |
| HOXA3 | 8/299 | 0.01084737792 5196319 | *TGFBR3;EBF1;NAV2;PHLDB2;RUNX2;FOXP1;GLI2;CREB5* |
| HSF5 | 8/299 | 0.01084737792 5196319 | *RIPOR2;ATXN1;ADGRG6;EBF1;IGF1R;FOXP1;SYNE1;CREB5* |
| KLF13 | 8/299 | 0.01084737792 5196319 | *IVNS1ABP;ATXN1;FNDC3B;FOXN3;NAV2;TNS3;IGF1R;FOXP1* |

| | | | |
|---|---|---|---|
| KLF7 | 8/299 | 0.01084737792 5196319 | *ATXN1;FNDC3B;NAV2;TNS3;IGF1R;FOXP1;SYNE1;CREB5* |
| LEF1 | 8/299 | 0.01084737792 5196319 | *TGFBR3;RIPOR2;EDNRB;CAMK4;EBF1;RUNX2;IGF1R;FOXP1* |
| LHX4 | 8/299 | 0.01084737792 5196319 | *TGFBR3;GRID1;SCN8A;EBF1;AGAP1;ANK3;NAV2;GLI2* |
| MYT1 | 8/299 | 0.01084737792 5196319 | *TOX3;SETBP1;EBF1;CACNA1D;ANK3;NAV2;ADCY1;NCKAP5* |
| NCOR1 | 8/299 | 0.01084737792 5196319 | *MACF1;SETD5;ANK3;BAZ2B;BRCA1;IGF1R;FOXP1;SYNE1* |
| NPAS2 | 8/299 | 0.01084737792 5196319 | *PDZD2;ATXN1;ANK3;NAV2;FBXO32;PLCB1;FOXP1;SYNE1* |
| NR2F2 | 8/299 | 0.01084737792 5196319 | *TGFBR3;EDNRB;EBF1;NAV2;TNS3;TEAD1;IGF1R;FOXP1* |
| NRF1 | 8/299 | 0.01084737792 5196319 | *IVNS1ABP;ATXN1;FOXN3;BRCA1;NAV2;TEAD1;IGF1R;FOXP1* |
| PAX2 | 8/299 | 0.01084737792 5196319 | *GRID1;EBF1;NAV2;ADCY1;TEAD1;RUNX2;GLI3;GLI2* |
| PAX5 | 8/299 | 0.01084737792 5196319 | *RIPOR2;SETBP1;EBF1;TNS3;RUNX2;IGF1R;FOXP1;GLI2* |
| PAX8 | 8/299 | 0.01084737792 5196319 | *VAV3;EBF1;NAV2;RUNX2;IGF1R;FOXP1;GLI2;CREB5* |
| PLXNA1 | 8/299 | 0.01084737792 5196319 | *MACF1;HIP1;FNDC3B;AGAP1;NAV2;TNS3;HSPG2;IGF1R* |
| POU3F4 | 8/299 | 0.01084737792 5196319 | *TOX3;EDNRB;EBF1;ZFPM2;NCKAP5;TEAD1;GLI3;GLI2* |
| RBPJ | 8/299 | 0.01084737792 5196319 | *IVNS1ABP;MACF1;ATXN1;FNDC3B;TNS3;TEAD1;IGF1R;FOXP1* |
| RC3H1 | 8/299 | 0.01084737792 5196319 | *IVNS1ABP;SLC4A7;MACF1;ATXN1;FNDC3B;BAZ2B;CDC42BPA;FOX P1* |
| RCOR1 | 8/299 | 0.01084737792 5196319 | *IVNS1ABP;ATXN1;FNDC3B;FOXN3;TNS3;TEAD1;IGF1R;FOXP1* |
| SOX11 | 8/299 | 0.01084737792 5196319 | *TOX3;EDNRB;GNG2;EBF1;NAV2;GLI3;IGF1R;GLI2* |
| TBX20 | 8/299 | 0.01084737792 5196319 | *TGFBR3;EDNRB;EBF1;NAV2;ZFPM2;TEAD1;GLI2;CREB5* |
| TBX4 | 8/299 | 0.01084737792 5196319 | *TMEM100;TGFBR3;EDNRB;WNT2B;EBF1;NAV2;TNS3;GLI2* |
| TBX5 | 8/299 | 0.01084737792 5196319 | *EDNRB;EBF1;NAV2;ZFPM2;RUNX2;FOXP1;GLI2;CREB5* |
| TCF7 | 8/299 | 0.01084737792 5196319 | *TGFBR3;RIPOR2;CAMK4;EBF1;NAV2;RUNX2;IGF1R;FOXP1* |
| TCF7L1 | 8/299 | 0.01084737792 5196319 | *TGFBR3;EDNRB;NAV2;TNS3;HSPG2;TEAD1;GLI3;GLI2* |
| TEAD3 | 8/299 | 0.01084737792 5196319 | *NAV2;TNS3;HSPG2;PHLDB2;TEAD1;GLI3;IGF1R;GLI2* |
| TIGD4 | 8/299 | 0.01084737792 5196319 | *TMEM100;INPP4B;ANKFN1;RBM47;EDNRB;ANK3;NAV2;SYNE1* |
| UBR4 | 8/299 | 0.01084737792 5196319 | *MACF1;ANK3;NAV2;TNS3;HSPG2;SYNE2;IGF1R;SYNE1* |
| ZBTB16 | 8/299 | 0.01084737792 5196319 | *TGFBR3;EDNRB;PDZD2;EBF1;NAV2;FBXO32;IGF1R;SYNE1* |
| ZBTB25 | 8/299 | 0.01084737792 5196319 | *IVNS1ABP;TGFBR3;ATXN1;CBLB;BAZ2B;FOXN3;SYNE2;IGF1R* |
| ZBTB46 | 8/299 | 0.01084737792 5196319 | *PDZD2;ATXN1;HIP1;DOCK9;EBF1;NAV2;TNS3;IGF1R* |
| ZNF148 | 8/299 | 0.01084737792 5196319 | *SLC4A7;MACF1;ATXN1;FNDC3B;BAZ2B;TEAD1;IGF1R;FOXP1* |
| ZNF20 | 8/299 | 0.01084737792 | *ATXN1;FNDC3B;CBLB;BAZ2B;NAV2;TNS3;SRGAP1;IGF1R* |

| | | | |
|---|---|---|---|
| | | 5196319 | |
| ZNF224 | 8/299 | 0.01084737792 5196319 | *IVNS1ABP;ZNF221;MACF1;ATXN1;BAZ2B;NAV2;IGF1R;SYNE1* |
| ZNF23 | 8/299 | 0.01084737792 5196319 | *IVNS1ABP;ATXN1;DOCK9;MTUS1;ANK3;NAV2;FBXO32;SYNE1* |
| ZNF235 | 8/299 | 0.01084737792 5196319 | *SETBP1;DOCK9;MTUS1;ANK3;BAZ2B;NAV2;SSBP2;SYNE1* |
| ZNF253 | 8/299 | 0.01084737792 5196319 | *ZNF320;VAV3;TGFBR3;ZNF519;MTUS1;ANK3;BAZ2B;SYNE2* |
| ZNF28 | 8/299 | 0.01084737792 5196319 | *ZNF320;VAV3;DOCK9;MTUS1;FOXN3;NAV2;CDC42BPA;SYNE2* |
| ZNF311 | 8/299 | 0.01084737792 5196319 | *CSGALNACT1;RIPOR2;INPP4B;EBF1;NAV2;TNS3;GLI2;CREB5* |
| ZNF34 | 8/299 | 0.01084737792 5196319 | *TGFBR3;ZNF827;MTUS1;ANK3;NAV2;FBXO32;SSBP2;TNS3* |
| ZNF354 C | 8/299 | 0.01084737792 5196319 | *NLGN1;TSHZ2;EBF1;ANK3;RNF150;TEAD1;GLI3;SYNE1* |
| ZNF443 | 8/299 | 0.01084737792 5196319 | *IVNS1ABP;VAV3;TGFBR3;ZNF519;BAZ2B;BRCA1;NAV2;TNS3* |
| ZNF501 | 8/299 | 0.01084737792 5196319 | *ZNF221;RIPOR2;PLEKHG1;ADCY1;ZFPM2;PHLDB2;SYNE1;CREB5* |
| ZNF507 | 8/299 | 0.01084737792 5196319 | *SLC4A7;SETD5;ZNF827;BAZ2B;CDC42BPA;SYNE2;IGF1R;FOXP1* |
| ZNF540 | 8/299 | 0.01084737792 5196319 | *TGFBR3;TSHZ2;EBF1;APBA1;ANK3;ADCY1;SYNE1;CREB5* |
| ZNF552 | 8/299 | 0.01084737792 5196319 | *ZNF320;VAV3;INPP4B;RBM47;ANK3;NAV2;TNS3;IGF1R* |
| ZNF578 | 8/299 | 0.01084737792 5196319 | *ZNF320;TGFBR3;RIPOR2;PLEKHG1;NLGN1;EDNRB;EBF1;IGF1R* |
| ZNF606 | 8/299 | 0.01084737792 5196319 | *ZNF320;VAV3;SETBP1;BAZ2B;NAV2;PTPN13;SSBP2;SYNE2* |
| ZNF610 | 8/299 | 0.01084737792 5196319 | *TGFBR3;RNF150;NAV2;FBXO32;PLCB1;SSBP2;IGF1R;CREB5* |
| ZNF615 | 8/299 | 0.01084737792 5196319 | *ZNF320;TGFBR3;RBM47;MTUS1;BAZ2B;PTPN13;SSBP2;IGF1R* |
| ZNF625 | 8/299 | 0.01084737792 5196319 | *ZNF320;VAV3;ZNF221;PLEKHA5;BAZ2B;RNF150;NAV2;IGF1R* |
| ZNF641 | 8/299 | 0.01084737792 5196319 | *TGFBR3;RBM47;ATXN1;HIP1;FNDC3B;FBXO32;IGF1R;FOXP1* |
| ZNF667 | 8/299 | 0.01084737792 5196319 | *TGFBR3;RIPOR2;EDNRB;MTUS1;ADCY1;FBXO32;TNS3;IGF1R* |
| ZNF70 | 8/299 | 0.01084737792 5196319 | *SETBP1;FNDC3B;PPARA;TNS3;SRGAP1;GLI3;IGF1R;CREB5* |
| ZNF720 | 8/299 | 0.01084737792 5196319 | *TGFBR3;SLC4A7;SFMBT2;CBLB;BAZ2B;FOXN3;SSBP2;SYNE2* |
| ZNF74 | 8/299 | 0.01084737792 5196319 | *VAV3;RIPOR2;ZNF609;HIP1;TFDP2;AGAP1;IGF1R;GLI2* |
| ZNF81 | 8/299 | 0.01084737792 5196319 | *SLC4A7;MACF1;SETBP1;MTUS1;BAZ2B;PPARA;SYNE2;IGF1R* |
| ZNF836 | 8/299 | 0.01084737792 5196319 | *ZNF320;VAV3;MACF1;CBLB;BAZ2B;FOXN3;FBXO32;SSBP2* |
| ZSCAN1 8 | 8/299 | 0.01084737792 5196319 | *TGFBR3;EDNRB;GNG2;SETBP1;NAV2;FBXO32;SSBP2;SYNE1* |
| ZXDB | 8/299 | 0.01084737792 5196319 | *TGFBR3;ATXN1;MTUS1;CBLB;NAV2;GAB2;FBXO32;IGF1R* |
| ARGFX | 7/299 | 0.03198218497 3890077 | *VAV3;FBXL20;RIPOR2;ZNF609;SFMBT2;ANO4;FOXP1* |
| ATOH8 | 7/299 | 0.03198218497 3890077 | *TGFBR3;EDNRB;EBF1;NAV2;FBXO32;TNS3;PHLDB2* |

| | | | |
|---|---|---|---|
| CAMTA2 | 7/299 | 0.03198218497 3890077 | *ATXN1;ANK3;NAV2;TNS3;HSPG2;FOXP1;SYNE1* |
| CPSF4L | 7/299 | 0.03198218497 3890077 | *PLEKHG1;RBM47;COL27A1;NAV2;TNS3;TEAD1;GLI2* |
| CREB1 | 7/299 | 0.03198218497 3890077 | *MACF1;ATXN1;CBLB;BAZ2B;BRCA1;IGF1R;FOXP1* |
| DBX2 | 7/299 | 0.03198218497 3890077 | *NLGN1;EDNRB;ZFPM2;SRGAP1;TEAD1;GLI3;GLI2* |
| DMRTA1 | 7/299 | 0.03198218497 3890077 | *TOX3;SEMA3D;TSHZ2;EBF1;TNS3;PHLDB2;GLI3* |
| DNAJC1 | 7/299 | 0.03198218497 3890077 | *TGFBR3;MACF1;ATXN1;FNDC3B;TNS3;IGF1R;FOXP1* |
| DUXA | 7/299 | 0.03198218497 3890077 | *ZNF320;FMN1;NCKAP5;TEAD1;GLI3;FOXP1;GLI2* |
| EBF4 | 7/299 | 0.03198218497 3890077 | *COL27A1;EBF1;NAV2;ADCY1;NCKAP5;HSPG2;GLI2* |
| EMX2 | 7/299 | 0.03198218497 3890077 | *TMEM100;TGFBR3;TOX3;EDNRB;EBF1;TEAD1;GLI3* |
| ESR2 | 7/299 | 0.03198218497 3890077 | *EBF1;BRCA1;PPARA;RUNX2;IGF1R;FOXP1;GLI2* |
| FBXO41 | 7/299 | 0.03198218497 3890077 | *SCN8A;AGAP1;ANK3;NAV2;ADCY1;PLCB1;SYNE1* |
| FEZF1 | 7/299 | 0.03198218497 3890077 | *TOX3;NLGN1;EDNRB;EBF1;NAV2;GLI3;GLI2* |
| FGD1 | 7/299 | 0.03198218497 3890077 | *COL27A1;HIP1;ANK3;NAV2;PTPN13;TNS3;HSPG2* |
| FOXF2 | 7/299 | 0.03198218497 3890077 | *TMEM100;TGFBR3;COL15A1;EDNRB;EBF1;RUNX2;GLI2* |
| GLIS2 | 7/299 | 0.03198218497 3890077 | *NAV2;TNS3;HSPG2;PHLDB2;GLI3;IGF1R;GLI2* |
| GRHL1 | 7/299 | 0.03198218497 3890077 | *VAV3;TGFBR3;RBM47;MTUS1;NAV2;FBXO32;IGF1R* |
| HEY2 | 7/299 | 0.03198218497 3890077 | *TMEM100;TGFBR3;EDNRB;EBF1;NAV2;ADCY1;FBXO32* |
| KLF11 | 7/299 | 0.03198218497 3890077 | *TGFBR3;MTUS1;FOXN3;FBXO32;TNS3;IGF1R;CREB5* |
| KLF15 | 7/299 | 0.03198218497 3890077 | *TMEM100;TGFBR3;EDNRB;MTUS1;FBXO32;PPARA;TNS3* |
| KLF8 | 7/299 | 0.03198218497 3890077 | *TGFBR3;RIPOR2;MTUS1;ANK3;NAV2;FBXO32;SYNE1* |
| LEUTX | 7/299 | 0.03198218497 3890077 | *NLGN1;GRID1;FMN1;FOXN3;ANO4;FOXP1;CREB5* |
| MBNL3 | 7/299 | 0.03198218497 3890077 | *VAV3;TGFBR3;RIPOR2;ADGRG6;NAV2;SYNE2;SYNE1* |
| MECP2 | 7/299 | 0.03198218497 3890077 | *MACF1;ATXN1;ANK3;TEAD1;IGF1R;FOXP1;SYNE1* |
| MEF2D | 7/299 | 0.03198218497 3890077 | *ATXN1;FNDC3B;NAV2;TNS3;TEAD1;IGF1R;FOXP1* |
| MEOX2 | 7/299 | 0.03198218497 3890077 | *CPED1;TMEM100;TGFBR3;COL15A1;EDNRB;EBF1;ZFPM2* |
| MGA | 7/299 | 0.03198218497 3890077 | *SLC4A7;MACF1;BAZ2B;CDC42BPA;SYNE2;IGF1R;FOXP1* |
| MZF1 | 7/299 | 0.03198218497 3890077 | *ATXN1;NAV2;PPARA;TEAD1;IGF1R;FOXP1;GLI2* |
| NFX1 | 7/299 | 0.03198218497 3890077 | *IVNS1ABP;MACF1;ATXN1;ANK3;TNS3;IGF1R;FOXP1* |
| NR2E1 | 7/299 | 0.03198218497 3890077 | *TOX3;EDNRB;EBF1;TEAD1;GLI3;GLI2;CREB5* |
| PAX3 | 7/299 | 0.03198218497 | *EDNRB;EBF1;RUNX2;GLI3;IGF1R;FOXP1;GLI2* |

| | | | |
|---|---|---|---|
| | | 3890077 | |
| POGZ | 7/299 | 0.03198218497 3890077 | *MACF1;SETD5;MBD5;ANK3;NAV2;IGF1R;FOXP1* |
| POU3F1 | 7/299 | 0.03198218497 3890077 | *EDNRB;GRID1;EBF1;NAV2;FBXO32;TEAD1;FOXP1* |
| POU3F2 | 7/299 | 0.03198218497 3890077 | *TOX3;NLGN1;EDNRB;EBF1;TEAD1;GLI3;GLI2* |
| POU3F3 | 7/299 | 0.03198218497 3890077 | *EDNRB;EBF1;NCKAP5;TEAD1;GLI3;IGF1R;GLI2* |
| PPARD | 7/299 | 0.03198218497 3890077 | *ATXN1;NAV2;PPARA;HSPG2;TEAD1;IGF1R;FOXP1* |
| RCOR3 | 7/299 | 0.03198218497 3890077 | *IVNS1ABP;MACF1;ATXN1;BAZ2B;CDC42BPA;IGF1R;FOXP1* |
| RGS7 | 7/299 | 0.03198218497 3890077 | *NLGN1;CAMK4;ANK3;ADCY1;PLCB1;NCKAP5;SYNE1* |
| RHOXF1 | 7/299 | 0.03198218497 3890077 | *CNTNAP3;TGFBR3;NLGN1;PLEKHH2;EDNRB;PDZD2;EBF1* |
| RUNX1 | 7/299 | 0.03198218497 3890077 | *ATXN1;FNDC3B;NAV2;TNS3;RUNX2;IGF1R;FOXP1* |
| SIX2 | 7/299 | 0.03198218497 3890077 | *TGFBR3;EBF1;NAV2;FBXO32;RUNX2;FOXP1;GLI2* |
| SMAD2 | 7/299 | 0.03198218497 3890077 | *IVNS1ABP;ATXN1;FOXN3;BRCA1;TEAD1;IGF1R;FOXP1* |
| SMAD6 | 7/299 | 0.03198218497 3890077 | *TGFBR3;EDNRB;NAV2;TNS3;HSPG2;IGF1R;GLI2* |
| SOX13 | 7/299 | 0.03198218497 3890077 | *TGFBR3;ANK3;NAV2;TNS3;HSPG2;SYNE2;GLI2* |
| SOX2 | 7/299 | 0.03198218497 3890077 | *TOX3;EDNRB;NAV2;TEAD1;GLI3;IGF1R;GLI2* |
| SRY | 7/299 | 0.03198218497 3890077 | *EBF1;BRCA1;PPARA;TEAD1;RUNX2;ESR2;FOXP1* |
| TCF21 | 7/299 | 0.03198218497 3890077 | *CPED1;TMEM100;TGFBR3;COL15A1;EDNRB;EBF1;ZFPM2* |
| TFAP2A | 7/299 | 0.03198218497 3890077 | *VAV3;EBF1;NAV2;TEAD1;RUNX2;IGF1R;GLI2* |
| TFCP2L 1 | 7/299 | 0.03198218497 3890077 | *TGFBR3;RBM47;MTUS1;ANK3;NAV2;FBXO32;TNS3* |
| THAP6 | 7/299 | 0.03198218497 3890077 | *RIPOR2;ANK3;BAZ2B;BRCA1;FBXO32;FOXP1;SYNE1* |
| TOX2 | 7/299 | 0.03198218497 3890077 | *TGFBR3;EDNRB;GNG2;SETBP1;EBF1;NAV2;TNS3* |
| TOX3 | 7/299 | 0.03198218497 3890077 | *VAV3;EDNRB;SETBP1;NAV2;ADCY1;NCKAP5;GLI3* |
| TRIM32 | 7/299 | 0.03198218497 3890077 | *IVNS1ABP;ATXN1;CBLB;TNS3;PHLDB2;FOXP1;SYNE1* |
| VEZF1 | 7/299 | 0.03198218497 3890077 | *IVNS1ABP;BAZ2B;FOXN3;TNS3;TEAD1;IGF1R;FOXP1* |
| WT1 | 7/299 | 0.03198218497 3890077 | *TGFBR3;EDNRB;EBF1;BRCA1;ZFPM2;RUNX2;IGF1R* |
| ZFP2 | 7/299 | 0.03198218497 3890077 | *TGFBR3;EBF1;MTUS1;ANK3;FBXO32;SSBP2;SYNE1* |
| ZFP28 | 7/299 | 0.03198218497 3890077 | *EDNRB;DOCK9;EBF1;MTUS1;ANK3;IGF1R;SYNE1* |
| ZFP30 | 7/299 | 0.03198218497 3890077 | *EDNRB;ATXN1;SETBP1;MTUS1;ANK3;SSBP2;IGF1R* |
| ZFP82 | 7/299 | 0.03198218497 3890077 | *TGFBR3;RIPOR2;TFDP2;BAZ2B;FBXO32;FOXP1;CREB5* |
| ZFX | 7/299 | 0.03198218497 3890077 | *SLC4A7;ATXN1;FNDC3B;BAZ2B;TEAD1;IGF1R;FOXP1* |

| | | | |
|---|---|---|---|
| ZKSCAN 4 | 7/299 | 0.031982184973890077 | *IVNS1ABP;FBXL20;ATXN1;MTUS1;FBXO32;TNS3;SYNE1* |
| ZMAT4 | 7/299 | 0.031982184973890077 | *TOX3;NLGN1;ANK3;NAV2;ADCY1;PLCB1;NCKAP5* |
| ZNF138 | 7/299 | 0.031982184973890077 | *ZNF519;TFDP2;MTUS1;PLEKHA5;ANK3;BAZ2B;SYNE2* |
| ZNF189 | 7/299 | 0.031982184973890077 | *IVNS1ABP;TGFBR3;MTUS1;ANK3;BAZ2B;FBXO32;PTPN13* |
| ZNF25 | 7/299 | 0.031982184973890077 | *TGFBR3;ATXN1;GNG2;FBXO32;SSBP2;PHLDB2;SYNE1* |
| ZNF250 | 7/299 | 0.031982184973890077 | *SETBP1;TFDP2;FNDC3B;CBLB;NAV2;FBXO32;SSBP2* |
| ZNF26 | 7/299 | 0.031982184973890077 | *TGFBR3;CBLB;BAZ2B;BRCA1;PLCB1;PHLDB2;FOXP1* |
| ZNF266 | 7/299 | 0.031982184973890077 | *IVNS1ABP;TGFBR3;GOLGA8B;FNDC3B;ANK3;NAV2;CREB5* |
| ZNF277 | 7/299 | 0.031982184973890077 | *IVNS1ABP;RIPOR2;CBLB;BAZ2B;FOXN3;FBXO32;SSBP2* |
| ZNF320 | 7/299 | 0.031982184973890077 | *VAV3;SETBP1;ANK3;FOXN3;NAV2;PLCB1;IGF1R* |
| ZNF350 | 7/299 | 0.031982184973890077 | *TGFBR3;ANK3;BRCA1;FBXO32;PTPN13;IGF1R;CREB5* |
| ZNF395 | 7/299 | 0.031982184973890077 | *TGFBR3;ATXN1;FOXN3;NAV2;SSBP2;TNS3;IGF1R* |
| ZNF396 | 7/299 | 0.031982184973890077 | *SETBP1;STON1;CBLB;BAZ2B;NAV2;FBXO32;SYNE1* |
| ZNF44 | 7/299 | 0.031982184973890077 | *RIPOR2;BAZ2B;NAV2;SYNE2;IGF1R;FOXP1;SYNE1* |
| ZNF454 | 7/299 | 0.031982184973890077 | *TOX3;EDNRB;EBF1;NAV2;ZFPM2;SYNE1;CREB5* |
| ZNF487 | 7/299 | 0.031982184973890077 | *CSGALNACT1;TGFBR3;FBXL20;PLEKHG1;ATXN1;FCHSD2;TNS3* |
| ZNF488 | 7/299 | 0.031982184973890077 | *VAV3;TGFBR3;MTUS1;ANK3;NAV2;FBXO32;CREB5* |
| ZNF502 | 7/299 | 0.031982184973890077 | *CSGALNACT1;TGFBR3;RIPOR2;ANK3;MOB3B;FOXP1;CREB5* |
| ZNF510 | 7/299 | 0.031982184973890077 | *IVNS1ABP;MBD5;TFDP2;CBLB;BAZ2B;SYNE2;IGF1R* |
| ZNF521 | 7/299 | 0.031982184973890077 | *TGFBR3;COL15A1;SETBP1;TSHZ2;EBF1;ZFPM2;GLI3* |
| ZNF527 | 7/299 | 0.031982184973890077 | *ZNF320;TGFBR3;ZNF221;HIP1;SETBP1;ANK3;BAZ2B* |
| ZNF554 | 7/299 | 0.031982184973890077 | *FBXL20;TFDP2;ANK3;GAB2;FBXO32;PPARA;CREB5* |
| ZNF558 | 7/299 | 0.031982184973890077 | *TGFBR3;GOLGA8B;SETBP1;EBF1;FOXN3;FBXO32;PLCB1* |
| ZNF559 | 7/299 | 0.031982184973890077 | *VAV3;TGFBR3;SETD5;MBD5;SETBP1;MTUS1;FOXP1* |
| ZNF562 | 7/299 | 0.031982184973890077 | *IVNS1ABP;SLC4A7;MACF1;ZNF609;FNDC3B;FOXN3;FOXP1* |
| ZNF582 | 7/299 | 0.031982184973890077 | *PDZD2;SETBP1;EBF1;ANK3;ZFPM2;FOXP1;CREB5* |
| ZNF607 | 7/299 | 0.031982184973890077 | *ZNF320;TGFBR3;ZNF519;PLEKHA5;NAV2;IGF1R;SYNE1* |
| ZNF629 | 7/299 | 0.031982184973890077 | *MACF1;ZNF609;HIP1;NAV2;SSBP2;TNS3;HSPG2* |
| ZNF662 | 7/299 | 0.031982184973890077 | *CSGALNACT1;TGFBR3;EDNRB;ZFPM2;TEAD1;IGF1R;CREB5* |
| ZNF681 | 7/299 | 0.03198218497 | *ZNF320;RIPOR2;PDZD2;ZNF519;MTUS1;ANK3;BRCA1* |

| | | | |
|---|---|---|---|
| | | 3890077 | |
| ZNF711 | 7/299 | 0.03198218497 3890077 | *VAV3;GNG2;SETBP1;ANK3;PLCB1;SSBP2;SYNE2* |
| ZNF772 | 7/299 | 0.03198218497 3890077 | *ZNF320;VAV3;ZNF221;PLEKHG1;DOCK9;ANK3;IGF1R* |
| ZNF792 | 7/299 | 0.03198218497 3890077 | *VAV3;ZNF827;CBLB;FBXO32;TNS3;RUNX2;CREB5* |
| ZNF793 | 7/299 | 0.03198218497 3890077 | *ZNF320;VAV3;SETBP1;SCN8A;ANK3;RNF150;FOXP1* |
| ZSCAN29 | 7/299 | 0.03198218497 3890077 | *IVNS1ABP;FBXL20;DOCK9;MTUS1;BAZ2B;FOXN3;IGF1R* |

**Table S10.** The 188 downregulated genes are simultaneously regulated in different combinations by 906 different transcription factors. The search was performed in TF-Gene Co-occurrence (Enrichr Submissions TF-Gene Co-occurrence, https://maayanlab.cloud/Enrichr/ ). Related to Figure 6D.

| Term | Overlap | Adjusted P-value | Genes |
|---|---|---|---|
| SOX5 | 28/299 | 7.836738071980201E-17 | *THRB;NEDD4L;AKAP6;CACNA1C;SLC7A11;EFNA5;AFF3;LPP;NR3C2;NPAS3;TTC28;CHL1;DLGAP1;ZFHX3;KLF12;ANGPT1;BCL11A;WNT5A;ETV1;SORBS2;ESR1;CDK6;NFIA;NFIB;DLC1;RARB;CDK14;EPHA3* |
| ZNF385B | 26/299 | 5.88752472375352E-15 | *THRB;MAOA;NEDD4L;TMTC1;AKAP6;SLC7A11;EFNA5;AFF3;NR3C2;DLGAP1;ANGPT1;ETV1;SORBS2;LHFPL3;ESR1;GNAO1;NFIA;NFIB;ALDH1A2;DLC1;MPPED2;RARB;CDK14;EPHA3;RAPGEF4;HCN1* |
| NPAS3 | 25/299 | 2.6908410588642888E-14 | *AKAP6;CACNA1C;EFNA5;AFF3;LPP;CHL1;DLGAP1;APBB2;SRGAP3;ZFHX3;KLF12;BCL11A;EYA2;ETV1;SORBS2;LHFPL3;ESR1;MAPK10;NFIA;ADGRB3;NFIB;DLC1;RARB;CDK14;EPHA3* |
| POU6F2 | 25/299 | 2.6908410588642888E-14 | *THRB;TMTC1;AKAP6;LDLRAD4;CACNA1C;EFNA5;AFF3;NR3C2;NPAS3;CHL1;DLGAP1;ZFHX3;BCL11A;SORBS2;LHFPL3;ESR1;MAPK10;NFIA;ADGRB3;NFIB;DLC1;RARB;KCNQ5;CDK14;HCN1* |
| RFX3 | 25/299 | 2.6908410588642888E-14 | *RERE;THRB;ZMYND8;NEDD4L;SLC7A11;IKZF2;EFNA5;LPP;BBX;SRGAP3;NCOA2;ZFHX3;KLF12;BCL11A;ETV1;SORBS2;ARID1B;MED13L;KLF7;CDK6;NFIA;TBL1XR1;NFIB;DMXL2;CDK14* |
| ESRRG | 24/299 | 1.6269594547759017E-13 | *ZFHX3;KLF12;THRB;ANGPT1;BCL11A;NEDD4L;ETV1;AKAP6;SORBS2;LHFPL3;CACNA1C;EFNA5;AFF3;ESR1;NR3C2;NPAS3;NFIA;NFIB;DLC1;MPPED2;RARB;KCNQ5;DLGAP1;RAPGEF4* |
| FOXP2 | 24/299 | 1.6269594547759017E-13 | *ZFHX3;KLF12;THRB;BCL11A;COL12A1;NEDD4L;TMTC1;ETV1;FIGN;SORBS2;CACNA1C;EFNA5;AFF3;ESR1;NR3C2;NPAS3;CDK6;NFIA;NFIB;DLC1;MPPED2;RARB;HMCN1;EPHA3* |
| RGS6 | 24/299 | 1.6269594547759017E-13 | *KLF12;THRB;PDE1C;BCL11A;RASGRF2;NEDD4L;TMTC1;AKAP6;SORBS2;LHFPL3;CACNA1C;EFNA5;AFF3;LPP;NPAS3;MAPK10;NFIA;CHL1;NFIB;DLC1;RARB;KCNQ5;DLGAP1;CDK14* |
| THRB | 24/299 | 1.6269594547759017E-13 | *ZFHX3;KLF12;ANGPT1;DAPK1;COL12A1;PRICKLE2;NEDD4L;TMTC1;SORBS2;CACNA1C;SLC7A11;EFNA5;AFF3;ESR1;NR3C2;MAPK10;CDK6;NFIA;NFIB;DLC1;RARB;DLGAP1;PPARG;CDK14* |
| ADAMTS17 | 23/299 | 1.3778487227503808E-12 | *ZFHX3;THRB;BCL11A;COL12A1;NEDD4L;TMTC2;TMTC1;SORBS2;LHFPL3;CACNA1C;EFNA5;AFF3;FBLN2;LPP;ESR1;NPAS3;CDK6;NFIA;NFIB;ALDH1A2;DLC1;HMCN1;SRGAP3* |
| ZNF407 | 23/299 | 1.3778487227503808E-12 | *RERE;NCOA2;ZFHX3;KLF12;KMT2A;BCL11A;ZMYND8;NEDD4L;VPS13B;SORBS2;CACNA1C;LPP;ARID1B;MED13L;TTN;CDK6;NFIA;DLC1;UBR5;BBX;CEP192;SBF2;CDK14* |
| PLXNA4 | 22/299 | 1.0640139162137608E-11 | *ZFHX3;PDE1C;BCL11A;COL12A1;PRICKLE2;NEDD4L;TMTC1;AKAP6;SORBS2;LAMB1;CACNA1C;AFF3;FBLN2;NFIA;CHL1;NFIB;DLC1;DLGAP1;HMCN1;SRGAP3* |

| | | | |
|---|---|---|---|
| | | | *;CDK14;EPHA3* |
| ZNF608 | 22/299 | 1.064013916213 7608E-11 | *ZFHX3;KLF12;DAPK1;BCL11A;ZMYND8;PRICKLE2;NEDD4L;TMTC2;ETV1;SORBS2;SLC7A11;EFNA5;AFF3;LPP;ARID1B;KLF7;CDK6;NFIA;NFIB;DLC1;CDK14;ZNF521* |
| ZNF662 | 22/299 | 1.064013916213 7608E-11 | *ZFHX3;ACSS3;THRB;ANGPT1;BCL11A;PELI2;PRICKLE2;TMTC1;SORBS2;LHFPL3;AFF3;FBLN2;LPP;ESR1;NFIA;CHL1;NFIB;DLC1;KCNQ5;PPARG;ZNF521;RAPGEF4* |
| FOXP1 | 21/299 | 5.550759152858 124E-11 | *RERE;ZFHX3;KLF12;CAMK2D;DAPK1;BCL11A;ZMYND8;NEDD4L;SORBS2;EFNA5;AFF3;LPP;ARID1B;MED13L;KLF7;CDK6;NFIA;TBL1XR1;NFIB;DLC1;APBB2* |
| GLI3 | 21/299 | 5.550759152858 124E-11 | *ZFHX3;BCL11A;COL12A1;WNT5A;ETV1;SORBS2;LAMB1;SLC7A11;EFNA5;AFF3;FBLN2;LPP;CDK6;NFIA;NFIB;DLC1;RARB;HMCN1;PRKD1;CDK14;EPHA3* |
| MYT1L | 21/299 | 5.550759152858 124E-11 | *BCL11A;NEDD4L;AKAP6;SORBS2;LHFPL3;CACNA1C;AFF3;ARID1B;NR3C2;MED13L;GNAO1;MAPK10;NFIA;TBL1XR1;ADGRB3;CHL1;NFIB;KCNQ3;DLGAP1;SRGAP3;RAPGEF4* |
| PRDM9 | 21/299 | 5.550759152858 124E-11 | *ZFHX3;THRB;ANGPT1;BCL11A;EYA2;COL12A1;NEDD4L;ETV1;AKAP6;LHFPL3;SLC7A11;EFNA5;AFF3;ESR1;NR3C2;TTN;NFIA;DLC1;DLGAP1;HMCN1;EPHA3* |
| SETBP1 | 21/299 | 5.550759152858 124E-11 | *RERE;ZFHX3;KLF12;DAPK1;BCL11A;PRICKLE2;NEDD4L;TMTC2;SORBS2;CACNA1C;EFNA5;AFF3;LPP;ARID1B;NR3C2;TTC28;NFIA;NFIB;DLC1;SRGAP3;CDK14* |
| SLC4A10 | 21/299 | 5.550759152858 124E-11 | *RASGRF2;PRICKLE2;AKAP6;SORBS2;LHFPL3;CACNA1C;NR3C2;GNAO1;MAPK10;CACNB4;ADGRB3;CHL1;DLC1;DMXL2;KCNQ3;KCNQ5;DLGAP1;PAK3;CDK14;RAPGEF4;HCN1* |
| SOX6 | 21/299 | 5.550759152858 124E-11 | *ZFHX3;KLF12;THRB;ANGPT1;BCL11A;COL12A1;WNT5A;NEDD4L;ETV1;AKAP6;SORBS2;EFNA5;AFF3;LPP;NPAS3;CDK6;NFIA;NFIB;DLC1;RARB;EPHA3* |
| ZFAT | 21/299 | 5.550759152858 124E-11 | *RERE;NCOA2;ZFHX3;KLF12;CAMK2D;DAPK1;BCL11A;ZMYND8;NEDD4L;EFNA5;AFF3;LPP;ARID1B;CDK6;NFIA;TBL1XR1;NFIB;DLC1;KCNQ5;APBB2;CDK14* |
| ZNF507 | 21/299 | 5.550759152858 124E-11 | *NCOA2;ZFHX3;KLF12;KMT2A;BCL11A;ZMYND8;NEDD4L;SLC7A11;IKZF2;LPP;ARID1B;NR3C2;MED13L;MBTD1;CDK6;NFIA;TBL1XR1;NFIB;DLC1;UBR5;BBX* |
| ZNF804B | 21/299 | 5.550759152858 124E-11 | *ZFHX3;THRB;COL24A1;EYA2;AKAP6;SORBS2;LHFPL3;CACNA1C;AFF3;NR3C2;NPAS3;ADGRB3;CHL1;DLC1;KCNQ3;KCNQ5;DLGAP1;HMCN1;EPHA3;RAPGEF4;HCN1* |
| ZNF827 | 21/299 | 5.550759152858 124E-11 | *RERE;ZFHX3;KLF12;KMT2A;BCL11A;ZMYND8;NEDD4L;SORBS2;SLC7A11;AFF3;LPP;ARID1B;NR3C2;KLF7;CDK6;NFIA;TBL1XR1;NFIB;DLC1;BBX;APBB2* |
| AFF3 | 20/299 | 3.397049917808 4554E-10 | *ZFHX3;KLF12;DAPK1;BCL11A;COL12A1;WNT5A;NEDD4L;TMTC1;SORBS2;CACNA1C;LPP;TTC28;CDK6;FMNL2;NFIA;NFIB;DLC1;KCNQ5;SRGAP3;EPHA3* |
| KLF12 | 20/299 | 3.397049917808 4554E-10 | *ZFHX3;CAMK2D;THRB;BCL11A;NEDD4L;ETV1;SORBS2;SLC7A11;IKZF2;EFNA5;AFF3;LPP;ARID1B;NR3C2;KLF7;CDK6;NFIA;NFIB;DLC1;CDK14* |
| PRDM5 | 20/299 | 3.397049917808 4554E-10 | *CAMK2D;THRB;ANGPT1;COL12A1;WNT5A;NEDD4L;ETV1;SORBS2;LAMB1;SLC7A11;EFNA5;LPP;GULP1;CDK6;NFIA;NFIB;DLC1;PPARG;PRKD1;CDK14* |
| RARB | 20/299 | 3.397049917808 4554E-10 | *ZFHX3;THRB;ANGPT1;WNT5A;ETV1;SORBS2;CACNA1C;EFNA5;AFF3;LPP;ESR1;CDK6;NFIA;NFIB;ALDH1A2;DLC1;MPPED2;PPARG;CDK14;EPHA3* |
| SCAPER | 20/299 | 3.397049917808 4554E-10 | *RERE;ZFHX3;KLF12;CAMK2D;ZMYND8;NEDD4L;VPS13B;SORBS2;LPP;ARID1B;NR3C2;TTC28;KLF7;NFIA;NFIB;DLC1;UBR5;DMXL2;BBX;SBF2* |
| ST18 | 20/299 | 3.397049917808 4554E-10 | *ZFHX3;PDE1C;BCL11A;ETV1;AKAP6;SORBS2;LHFPL3;SLC7A11;AFF3;NPAS3;GNAO1;FMNL2;NFIA;ADGRB3;CHL1;NFIB;DLC1;DLGAP1;EPHA3;RAPGEF4* |
| TBX5 | 20/299 | 3.397049917808 4554E-10 | *ZFHX3;PDE1C;ANGPT1;COL12A1;WNT5A;ETV1;SORBS2;CACNA1C;AFF3;ESR1;TTN;ADGRD1;NFIA;NFIB;ALDH1A2;DLC1;RARB;PPARG;HMCN1;EPHA3* |
| ZNF354C | 20/299 | 3.397049917808 4554E-10 | *ZFHX3;KLF12;THRB;MEGF10;ANGPT1;PELI2;TMTC2;ETV1;LHFPL3;SLC7A11;AFF3;LPP;ESR1;NFIA;NFIB;DLC1;PPARG;PRKD1;ZNF521;EPHA3* |

| | | | |
|---|---|---|---|
| ZNF385D | 20/299 | 3.3970499178084554E-10 | *THRB;ANGPT1;BCL11A;NEDD4L;TMTC1;ETV1;AKAP6;SORBS2;LHFPL3;EFNA5;AFF3;NR3C2;NFIA;ADGRB3;CHL1;NFIB;DLC1;DLGAP1;CDK14;EPHA3* |
| ZNF536 | 20/299 | 3.3970499178084554E-10 | *ZFHX3;PDE1C;MEGF10;BCL11A;TMTC2;ETV1;AKAP6;SORBS2;LHFPL3;CACNA1C;AFF3;NPAS3;GNAO1;NFIA;ADGRB3;CHL1;DLC1;MPPED2;ZNF521;EPHA3* |
| ZNF573 | 20/299 | 3.3970499178084554E-10 | *ZFHX3;KLF12;KMT2A;BCL11A;ZMYND8;NEDD4L;SORBS2;SLC7A11;AFF3;LPP;ACACB;NR3C2;TTC28;ZNF607;NFIA;NFIB;ZNF429;DLC1;ZNF615;ZNF234* |
| CAMTA1 | 19/299 | 2.1195755876685772E-9 | *RERE;ZFHX3;BCL11A;NEDD4L;AKAP6;SORBS2;CACNA1C;AFF3;LPP;ESR1;ARID1B;NPAS3;CDK6;NFIA;NFIB;DLC1;DLGAP1;SRGAP3;CDK14* |
| KIAA1549 | 19/299 | 2.1195755876685772E-9 | *ZFHX3;KLF12;KMT2A;BCL11A;COL12A1;NEDD4L;ETV1;SORBS2;SLC7A11;ASAP2;AFF3;LPP;ARID1B;KLF7;CDK6;NFIA;NFIB;DLC1;SRGAP3* |
| NCOA1 | 19/299 | 2.1195755876685772E-9 | *RERE;NCOA2;ZFHX3;CAMK2D;KMT2A;DAPK1;NEDD4L;LPP;ESR1;ARID1B;MED13L;CDK6;NFIA;TBL1XR1;NFIB;DLC1;UBR5;BBX;PPARG* |
| NCOA2 | 19/299 | 2.1195755876685772E-9 | *RERE;ZFHX3;KLF12;CAMK2D;KMT2A;ZMYND8;NEDD4L;VPS13B;LPP;ESR1;ARID1B;MED13L;CDK6;NFIA;TBL1XR1;NFIB;DLC1;UBR5;BBX* |
| PRDM16 | 19/299 | 2.1195755876685772E-9 | *ZFHX3;THRB;BCL11A;EYA2;NEDD4L;SHROOM3;TMTC1;SORBS2;CACNA1C;EFNA5;AFF3;LPP;ESR1;ARID1B;NPAS3;CDK6;NFIA;NFIB;DLC1* |
| SATB2 | 19/299 | 2.1195755876685772E-9 | *ZFHX3;CAMK2D;THRB;BCL11A;COL12A1;WNT5A;NEDD4L;TMTC1;ETV1;LAMB1;EFNA5;AFF3;KLF7;CDK6;NFIA;NFIB;DLC1;PPARG;CDK14* |
| TOX | 19/299 | 2.1195755876685772E-9 | *ZFHX3;KLF12;ANGPT1;DAPK1;BCL11A;WNT5A;NEDD4L;ETV1;SORBS2;SLC7A11;EFNA5;AFF3;LPP;CDK6;NFIA;NFIB;DLC1;RARB;CDK14* |
| TRERF1 | 19/299 | 2.1195755876685772E-9 | *RERE;ZFHX3;KLF12;DAPK1;BCL11A;ZMYND8;NEDD4L;SORBS2;SLC7A11;IKZF2;EFNA5;AFF3;LPP;ARID1B;CDK6;NFIA;NFIB;DLC1;APBB2* |
| TUB | 19/299 | 2.1195755876685772E-9 | *PRICKLE2;NEDD4L;TMTC1;ETV1;AKAP6;SORBS2;LHFPL3;CACNA1C;EFNA5;AFF3;KBTBD11;GNAO1;MAPK10;CACNB4;NFIA;CHL1;NFIB;DLC1;DLGAP1* |
| ZKSCAN2 | 19/299 | 2.1195755876685772E-9 | *RERE;ZFHX3;KLF12;KMT2A;BCL11A;PRICKLE2;NEDD4L;AKAP6;SORBS2;LHFPL3;SLC7A11;EFNA5;AFF3;ARID1B;NFIA;NFIB;DLC1;HMCN1;SRGAP3* |
| ZNF618 | 19/299 | 2.1195755876685772E-9 | *ZFHX3;KLF12;DAPK1;ZMYND8;COL12A1;MX1;NEDD4L;SORBS2;SLC7A11;AFF3;LPP;ARID1B;KLF7;CDK6;FMNL2;NFIA;NFIB;DLC1;SRGAP3* |
| ZNF660 | 19/299 | 2.1195755876685772E-9 | *KLF12;THRB;PDE1C;PRICKLE2;SORBS2;SLC7A11;AFF3;LPP;NPAS3;MAPK10;KLF7;NFIA;NFIB;ZNF429;DLC1;MPPED2;KCNQ5;SRGAP3;ZNF521* |
| BAZ2B | 18/299 | 1.245536511014006E-8 | *RERE;NCOA2;ZFHX3;CAMK2D;KMT2A;BCL11A;ZMYND8;SORBS2;LPP;ARID1B;MED13L;TANC1;CDK6;NFIA;NFIB;DLC1;DMXL2;BBX* |
| CHD7 | 18/299 | 1.245536511014006E-8 | *RERE;ZFHX3;KMT2A;DAPK1;BCL11A;ZMYND8;NEDD4L;LPP;ARID1B;MED13L;TANC1;CDK6;FMNL2;NFIA;TBL1XR1;NFIB;DLC1;UBR5* |
| HMBOX1 | 18/299 | 1.245536511014006E-8 | *RERE;NCOA2;ZFHX3;KLF12;KMT2A;ZMYND8;NEDD4L;VPS13B;ETV1;LPP;MED13L;KLF7;NFIA;TBL1XR1;NFIB;DLC1;UBR5;BBX* |
| HNF4G | 18/299 | 1.245536511014006E-8 | *KLF12;THRB;MAOA;BCL11A;ATP8B1;COL12A1;ETV1;SORBS2;SLC7A11;EFNA5;ESR1;NR3C2;NFIA;NFIB;DLC1;KCNQ5;PPARG;EPHA3* |
| NR2C2 | 18/299 | 1.245536511014006E-8 | *RERE;NCOA2;ZFHX3;KLF12;KMT2A;ZMYND8;VPS13B;LPP;ARID1B;MED13L;KLF7;CDK6;NFIA;TBL1XR1;NFIB;DLC1;UBR5;BBX* |
| NR3C2 | 18/299 | 1.245536511014006E-8 | *KLF12;THRB;DAPK1;MAOA;BCL11A;PELI2;NEDD4L;TMTC1;ETV1;SORBS2;AFF3;ESR1;NFIA;NFIB;DLC1;PPARG;SRGAP3;RAPGEF4* |
| NR6A1 | 18/299 | 1.245536511014006E-8 | *ZFHX3;THRB;DAPK1;BCL11A;ZMYND8;NEDD4L;FIGN;SLC7A11;EFNA5;LPP;CECR2;KLF7;CDK6;NFIA;NFIB;DLC1;RARB;PPARG* |
| PAX3 | 18/299 | 1.245536511014006E-8 | *ZFHX3;BCL11A;WNT5A;ETV1;AKAP6;LHFPL3;EFNA5;AFF3;ESR1;NPAS3;CDK6;NFIA;NFIB;ALDH1A2;DLC1;RARB;PPARG;EPHA3* |
| POU2F1 | 18/299 | 1.245536511014 | *RERE;NCOA2;ZFHX3;KLF12;KMT2A;BCL11A;ZMYND8* |

| | | | |
|---|---|---|---|
| | | 006E-8 | *;NEDD4L;LPP;ARID1B;MED13L;KLF7;CDK6;NFIA;TBL1XR1;NFIB;DLC1;BBX* |
| ZFHX3 | 18/299 | 1.245536511014006E-8 | *RERE;KLF12;KMT2A;BCL11A;ZMYND8;NEDD4L;EFNA5;AFF3;LPP;ARID1B;MED13L;TTC28;KLF7;CDK6;NFIA;NFIB;DLC1;APBB2* |
| ZNF462 | 18/299 | 1.245536511014006E-8 | *RERE;ZFHX3;DAPK1;BCL11A;ZMYND8;NEDD4L;SHROOM3;ETV1;SORBS2;LAMB1;EFNA5;LPP;TTC28;TANC1;CDK6;NFIA;NFIB;DLC1* |
| ZNF471 | 18/299 | 1.245536511014006E-8 | *KLF12;PDE1C;ANGPT1;BCL11A;COL12A1;NEDD4L;AKAP6;SORBS2;SLC7A11;AFF3;LPP;NR3C2;CHL1;NFIB;ZNF429;DLC1;MPPED2;ZNF521* |
| ZNF532 | 18/299 | 1.245536511014006E-8 | *ZFHX3;BCL11A;ZMYND8;COL12A1;NEDD4L;SORBS2;LAMB1;SLC7A11;LPP;KLF7;CDK6;FMNL2;NFIA;TBL1XR1;NFIB;DLC1;BBX;APBB2* |
| ZNF546 | 18/299 | 1.245536511014006E-8 | *KLF12;GDPD1;SORBS2;SLC7A11;IKZF2;EFNA5;LPP;ESR1;TTC28;CDK6;NFIA;NFIB;ZNF429;DLC1;BBX;APBB2;ZNF234;CFAP44* |
| ZNF577 | 18/299 | 1.245536511014006E-8 | *BCL11A;NEDD4L;SORBS2;SLC7A11;ACACB;SHROOM4;NR3C2;MBTD1;CDK6;NFIA;TBC1D32;NFIB;ZNF429;DLC1;ZNF615;ZNF521;ZNF234;CFAP44* |
| ZNF704 | 18/299 | 1.245536511014006E-8 | *ZFHX3;KLF12;PELI2;NEDD4L;ETV1;SORBS2;SLC7A11;EFNA5;AFF3;LPP;KLF7;CDK6;FMNL2;NFIA;NFIB;DLC1;PPARG;HMCN1* |
| ADAMTS19 | 17/299 | 6.592266488202851E-8 | *ANGPT1;BCL11A;COL12A1;WNT5A;SORBS2;LHFPL3;CACNA1C;EFNA5;AFF3;FBLN2;TTN;ADGRB3;ALDH1A2;DLC1;MPPED2;HMCN1;EPHA3* |
| ATF7 | 17/299 | 6.592266488202851E-8 | *RERE;NCOA2;ZFHX3;KLF12;KMT2A;ZMYND8;NEDD4L;LPP;ARID1B;MED13L;KLF7;CDK6;NFIA;NFIB;DLC1;BBX;APBB2* |
| ATXN7 | 17/299 | 6.592266488202851E-8 | *RERE;NCOA2;ZFHX3;KMT2A;ZMYND8;NEDD4L;VPS13B;LPP;ARID1B;MED13L;KLF7;NFIA;TBL1XR1;NFIB;DLC1;UBR5;BBX* |
| BACH2 | 17/299 | 6.592266488202851E-8 | *ZFHX3;KLF12;BCL11A;PELI2;NEDD4L;ETV1;SLC7A11;EFNA5;AFF3;LPP;TTC28;KLF7;CDK6;NFIA;NFIB;DLC1;CDK14* |
| CHD6 | 17/299 | 6.592266488202851E-8 | *RERE;ZFHX3;KMT2A;BCL11A;ZMYND8;NEDD4L;SORBS2;CACNA1C;AFF3;LPP;ARID1B;MED13L;CDK6;NFIA;NFIB;DLC1;SRGAP3* |
| CHD9 | 17/299 | 6.592266488202851E-8 | *RERE;NCOA2;ZFHX3;KMT2A;ZMYND8;NEDD4L;VPS13B;LPP;ARID1B;MED13L;CDK6;NFIA;TBL1XR1;NFIB;DLC1;UBR5;BBX* |
| DPF3 | 17/299 | 6.592266488202851E-8 | *ZFHX3;THRB;BCL11A;WNT5A;NEDD4L;SORBS2;CACNA1C;EFNA5;AFF3;ESR1;NR3C2;CDK6;NFIA;NFIB;DLC1;DLGAP1;SRGAP3* |
| FOXN3 | 17/299 | 6.592266488202851E-8 | *RERE;ZFHX3;KLF12;ZMYND8;NEDD4L;SORBS2;AFF3;LPP;ARID1B;MED13L;KLF7;CDK6;NFIA;TBL1XR1;NFIB;DLC1;APBB2* |
| HELZ | 17/299 | 6.592266488202851E-8 | *RERE;NCOA2;ZFHX3;KMT2A;ZMYND8;NEDD4L;VPS13B;LPP;ARID1B;MED13L;KLF7;CDK6;NFIA;TBL1XR1;NFIB;UBR5;BBX* |
| LCOR | 17/299 | 6.592266488202851E-8 | *RERE;NCOA2;ZFHX3;KLF12;KMT2A;NEDD4L;VPS13B;SLC7A11;LPP;ARID1B;MED13L;KLF7;CDK6;NFIA;TBL1XR1;NFIB;BBX* |
| LCORL | 17/299 | 6.592266488202851E-8 | *RERE;ZFHX3;KLF12;CAMK2D;BCL11A;NEDD4L;SLC7A11;EFNA5;LPP;ARID1B;MBTD1;CDK6;NFIA;TBL1XR1;NFIB;DLC1;BBX* |
| PKHD1 | 17/299 | 6.592266488202851E-8 | *KLF12;COL12A1;AKAP6;SORBS2;LHFPL3;CACNA1C;SLC7A11;AFF3;LPP;ESR1;NR3C2;TTN;ADGRB3;DLC1;KCNQ5;HMCN1;EPHA3* |
| PRDM10 | 17/299 | 6.592266488202851E-8 | *RERE;NCOA2;ZFHX3;KLF12;KMT2A;ZMYND8;NEDD4L;LPP;ARID1B;MED13L;KLF7;CDK6;NFIA;TBL1XR1;NFIB;DLC1;BBX* |
| PRDM6 | 17/299 | 6.592266488202851E-8 | *ZFHX3;ANGPT1;BCL11A;EYA2;COL12A1;WNT5A;TMTC1;CACNA1C;EFNA5;AFF3;ESR1;NFIA;NFIB;ALDH1A2;DLC1;HMCN1;EPHA3* |
| RAPGEF5 | 17/299 | 6.592266488202851E-8 | *ZFHX3;DAPK1;BCL11A;NEDD4L;TMTC2;TMTC1;ETV1;SORBS2;SLC7A11;LPP;KLF7;CDK6;NFIA;NFIB;DLC1;APBB2;CDK14* |
| RFX7 | 17/299 | 6.592266488202 | *RERE;NCOA2;ZFHX3;KLF12;KMT2A;BCL11A;LPP;ARID1B;MED13L;MBTD1;CDK6;NFIA;TBL1XR1;NFIB;U* |

| | | | |
|---|---|---|---|
| | | 851E-8 | *BR5;BBX;SRGAP3* |
| RORA | 17/299 | 6.592266488202851E-8 | *ZFHX3;KLF12;CAMK2D;THRB;DAPK1;BCL11A;NEDD4L;SLC7A11;EFNA5;AFF3;LPP;NR3C2;KLF7;CDK6;NFIA;NFIB;DLC1* |
| RORB | 17/299 | 6.592266488202851E-8 | *THRB;BCL11A;ETV1;SORBS2;LHFPL3;SLC7A11;EFNA5;NPAS3;GNAO1;NFIA;CHL1;NFIB;DLC1;RARB;KCNQ5;DLGAP1;EPHA3* |
| TCF12 | 17/299 | 6.592266488202851E-8 | *RERE;NCOA2;ZFHX3;KLF12;CAMK2D;KMT2A;BCL11A;ZMYND8;LPP;ARID1B;MED13L;CDK6;NFIA;TBL1XR1;NFIB;DLC1;BBX* |
| ZBTB44 | 17/299 | 6.592266488202851E-8 | *RERE;NCOA2;ZFHX3;KLF12;KMT2A;BCL11A;NEDD4L;VPS13B;LPP;ARID1B;MED13L;MBTD1;CDK6;NFIA;TBL1XR1;NFIB;BBX* |
| ZIM2 | 17/299 | 6.592266488202851E-8 | *ZFHX3;COL12A1;TMTC1;SORBS2;CACNA1C;AFF3;ESR1;NPAS3;TTN;MAPK10;ADGRD1;ADGRB3;CHL1;DLC1;ZNF536;RAPGEF4;HCN1* |
| ZNF311 | 17/299 | 6.592266488202851E-8 | *ZFHX3;GABBR1;THRB;BCL11A;COL12A1;WNT5A;MX1;NEDD4L;SORBS2;LHFPL3;SLC7A11;EFNA5;ESR1;CDK6;NFIA;NFIB;DLC1* |
| ZNF609 | 17/299 | 6.592266488202851E-8 | *RERE;NCOA2;ZFHX3;KMT2A;ZMYND8;NEDD4L;VPS13B;LPP;ARID1B;MED13L;CDK6;NFIA;TBL1XR1;NFIB;UBR5;BBX;APBB2* |
| ZNF793 | 17/299 | 6.592266488202851E-8 | *BCL11A;MX1;GDPD1;TMTC1;SLC7A11;IKZF2;AFF3;LPP;NR3C2;ZNF607;NFIA;NFIB;ZNF429;DLC1;ZNF615;ZNF665;ZNF234* |
| ZSCAN23 | 17/299 | 6.592266488202851E-8 | *ZFHX3;KLF12;GABBR1;DAPK1;TMTC1;FIGN;AKAP6;SORBS2;LDLRAD4;EFNA5;NR3C2;NFIA;NFIB;DLC1;KCNQ5;SRGAP3;ZNF521* |
| ARNT | 16/299 | 3.399130301563878E-7 | *RERE;NCOA2;ZFHX3;CAMK2D;KMT2A;DAPK1;LPP;ESR1;CDK6;NFIA;TBL1XR1;NFIB;DLC1;UBR5;BBX;PPARG* |
| BCL11A | 16/299 | 3.399130301563878E-7 | *ZFHX3;DAPK1;ETV1;EFNA5;AFF3;ARID1B;NR3C2;MED13L;CDK6;NFIA;TBL1XR1;NFIB;DLC1;MPPED2;SRGAP3;CDK14* |
| BNC2 | 16/299 | 3.399130301563878E-7 | *ZFHX3;ANGPT1;COL12A1;WNT5A;ETV1;EFNA5;AFF3;FBLN2;LPP;CDK6;NFIA;NFIB;DLC1;HMCN1;CDK14;EPHA3* |
| DACH1 | 16/299 | 3.399130301563878E-7 | *ZFHX3;KLF12;ANGPT1;BCL11A;WNT5A;NEDD4L;ETV1;SORBS2;SLC7A11;EFNA5;AFF3;NFIA;NFIB;DLC1;RARB;EPHA3* |
| GLIS3 | 16/299 | 3.399130301563878E-7 | *ZFHX3;COL12A1;WNT5A;NEDD4L;SORBS2;LAMB1;SLC7A11;EFNA5;AFF3;LPP;CDK6;NFIA;NFIB;DLC1;PPARG;CDK14* |
| HMG20A | 16/299 | 3.399130301563878E-7 | *RERE;NCOA2;ZFHX3;CAMK2D;BCL11A;ZMYND8;NEDD4L;LPP;ARID1B;MED13L;CDK6;NFIA;TBL1XR1;NFIB;DLC1;BBX* |
| MYT1 | 16/299 | 3.399130301563878E-7 | *BCL11A;EYA2;ETV1;SORBS2;LHFPL3;CACNA1C;GNAO1;MAPK10;NFIA;ADGRB3;CHL1;NFIB;DLC1;DLGAP1;SRGAP3;PAK3* |
| NR5A2 | 16/299 | 3.399130301563878E-7 | *ZFHX3;KLF12;THRB;BCL11A;COL12A1;WNT5A;NEDD4L;ETV1;EFNA5;ESR1;NR3C2;NFIA;NFIB;DLC1;PPARG;HMCN1* |
| PGR | 16/299 | 3.399130301563878E-7 | *THRB;ANGPT1;COL12A1;WNT5A;ETV1;SORBS2;AFF3;ESR1;NR3C2;CDK6;NFIA;CHL1;NFIB;DLC1;PPARG;EPHA3* |
| PHF20 | 16/299 | 3.399130301563878E-7 | *RERE;NCOA2;ZFHX3;CAMK2D;KMT2A;ZMYND8;NEDD4L;LPP;ARID1B;MED13L;CDK6;TBL1XR1;NFIB;DLC1;UBR5;BBX* |
| PKNOX2 | 16/299 | 3.399130301563878E-7 | *ZFHX3;THRB;COL12A1;WNT5A;PRICKLE2;TMTC1;SORBS2;CACNA1C;EFNA5;AFF3;NFIA;NFIB;DLC1;RARB;HMCN1;EPHA3* |
| PRDM15 | 16/299 | 3.399130301563878E-7 | *RERE;ZFHX3;KMT2A;BCL11A;ZMYND8;NEDD4L;SORBS2;AFF3;LPP;ARID1B;KLF7;CDK6;NFIA;NFIB;DLC1;APBB2* |
| RERE | 16/299 | 3.399130301563878E-7 | *NCOA2;ZFHX3;KMT2A;ZMYND8;NEDD4L;SORBS2;AFF3;LPP;ARID1B;MED13L;CDK6;NFIA;TBL1XR1;NFIB;DLC1;UBR5* |
| RREB1 | 16/299 | 3.399130301563878E-7 | *RERE;NCOA2;ZFHX3;KMT2A;ZMYND8;NEDD4L;LPP;ARID1B;MED13L;TANC1;CDK6;NFIA;TBL1XR1;NFIB;DLC1;BBX* |

| | | | |
|---|---|---|---|
| SIM1 | 16/299 | 3.399130301563878E-7 | *ZFHX3;THRB;PDE1C;COL12A1;WNT5A;ETV1;LHFPL3;EFNA5;LPP;ESR1;NPAS3;NFIA;NFIB;ALDH1A2;DLC1;KCNQ5* |
| SP4 | 16/299 | 3.399130301563878E-7 | *NCOA2;ZFHX3;KLF12;THRB;BCL11A;NEDD4L;ETV1;IKZF2;NR3C2;KLF7;CDK6;NFIA;TBL1XR1;NFIB;DLC1;BBX* |
| TCF7L2 | 16/299 | 3.399130301563878E-7 | *RERE;ZFHX3;DAPK1;BCL11A;NEDD4L;EFNA5;LPP;ARID1B;NR3C2;MED13L;CDK6;NFIA;TBL1XR1;NFIB;DLC1;PPARG* |
| TEAD1 | 16/299 | 3.399130301563878E-7 | *ZFHX3;COL12A1;NEDD4L;SORBS2;LAMB1;SLC7A11;EFNA5;LPP;TANC1;CDK6;FMNL2;NFIA;NFIB;DLC1;BBX;APBB2* |
| TFAP2B | 16/299 | 3.399130301563878E-7 | *ZFHX3;BCL11A;EYA2;COL12A1;WNT5A;ETV1;EFNA5;AFF3;ESR1;NFIA;CHL1;NFIB;ALDH1A2;DLC1;RARB;EPHA3* |
| ZBTB20 | 16/299 | 3.399130301563878E-7 | *RERE;ZFHX3;KLF12;CAMK2D;ANGPT1;NEDD4L;SORBS2;LPP;NR3C2;TTC28;KLF7;CDK6;NFIA;NFIB;DLC1;BBX* |
| ZFHX4 | 16/299 | 3.399130301563878E-7 | *ZFHX3;ANGPT1;COL12A1;WNT5A;ETV1;FIGN;SLC7A11;EFNA5;AFF3;LPP;NFIA;NFIB;DLC1;HMCN1;CDK14;EPHA3* |
| ZFPM2 | 16/299 | 3.399130301563878E-7 | *ANGPT1;COL12A1;WNT5A;ETV1;SORBS2;LAMB1;CACNA1C;EFNA5;AFF3;NR3C2;NFIA;NFIB;DLC1;HMCN1;PRKD1;EPHA3* |
| ZIC4 | 16/299 | 3.399130301563878E-7 | *ZFHX3;PDE1C;BCL11A;EYA2;COL12A1;WNT5A;LHFPL3;EFNA5;ESR1;NPAS3;NFIA;NFIB;ALDH1A2;DLC1;RARB;EPHA3* |
| ZNF236 | 16/299 | 3.399130301563878E-7 | *RERE;NCOA2;ZFHX3;KLF12;KMT2A;ZMYND8;NEDD4L;TMTC2;VPS13B;LPP;ARID1B;MED13L;NFIB;UBR5;BBX;CEP192* |
| ZNF516 | 16/299 | 3.399130301563878E-7 | *RERE;ZFHX3;DAPK1;BCL11A;PELI2;NEDD4L;LDLRAD4;AFF3;LPP;ARID1B;KLF7;CDK6;NFIA;NFIB;DLC1;APBB2* |
| ZNF605 | 16/299 | 3.399130301563878E-7 | *ZFHX3;KLF12;DAPK1;BCL11A;ZMYND8;GDPD1;NEDD4L;SLC7A11;IKZF2;CDK6;ZNF607;NFIA;NFIB;ZNF615;BBX;CFAP44* |
| ZNF713 | 16/299 | 3.399130301563878E-7 | *ZFHX3;KLF12;KMT2A;BCL11A;MX1;GDPD1;PRICKLE2;ETV1;SORBS2;SLC7A11;TTC28;NFIA;NFIB;DLC1;HMCN1;RAPGEF4* |
| ZNF718 | 16/299 | 3.399130301563878E-7 | *BCL11A;NEDD4L;TMTC1;SORBS2;LHFPL3;LDLRAD4;SLC7A11;AFF3;TRERF1;TTC28;CECR2;CDK6;NFIA;DLC1;ZNF615;DLGAP1* |
| ZNF8 | 16/299 | 3.399130301563878E-7 | *RERE;ZFHX3;KLF12;KMT2A;DAPK1;BCL11A;ZMYND8;NEDD4L;LPP;ESR1;ARID1B;MED13L;KLF7;CDK6;NFIB;DLC1* |
| ZNF804A | 16/299 | 3.399130301563878E-7 | *ANGPT1;BCL11A;COL12A1;WNT5A;NEDD4L;TMTC1;LHFPL3;SLC7A11;EFNA5;AFF3;NFIA;ADGRB3;DLC1;HMCN1;CDK14;EPHA3* |
| ALX1 | 15/299 | 1.679936341713079E-6 | *ZFHX3;COL12A1;WNT5A;ETV1;FIGN;LHFPL3;SLC7A11;EFNA5;NFIA;NFIB;ALDH1A2;DLC1;RARB;PPARG;HMCN1* |
| AR | 15/299 | 1.679936341713079E-6 | *THRB;ANGPT1;DAPK1;MAOA;COL12A1;WNT5A;ETV1;AFF3;ESR1;NR3C2;CDK6;NFIA;NFIB;DLC1;PPARG* |
| ARNT2 | 15/299 | 1.679936341713079E-6 | *BCL11A;COL12A1;WNT5A;NEDD4L;TMTC1;ETV1;SORBS2;SLC7A11;AFF3;GNAO1;CDK6;NFIA;NFIB;DLC1;SRGAP3* |
| BBX | 15/299 | 1.679936341713079E-6 | *RERE;NCOA2;ZFHX3;CAMK2D;KMT2A;ZMYND8;NEDD4L;LPP;ARID1B;MED13L;KLF7;CDK6;NFIA;TBL1XR1;NFIB* |
| CUX1 | 15/299 | 1.679936341713079E-6 | *RERE;ZFHX3;CAMK2D;BCL11A;ZMYND8;NEDD4L;AFF3;LPP;ARID1B;MED13L;CDK6;NFIA;TBL1XR1;NFIB;DLC1* |
| DZIP1 | 15/299 | 1.679936341713079E-6 | *ANGPT1;DAPK1;ST8SIA1;COL12A1;WNT5A;ETV1;SORBS2;LAMB1;EFNA5;FBLN2;TTC28;NFIA;CHL1;NFIB;DLC1* |
| ELF2 | 15/299 | 1.679936341713079E-6 | *RERE;NCOA2;ZFHX3;CAMK2D;ZMYND8;NEDD4L;LPP;ARID1B;MED13L;CDK6;NFIA;TBL1XR1;NFIB;DLC1;BBX* |
| FOXJ3 | 15/299 | 1.679936341713 | *RERE;ZFHX3;KMT2A;ZMYND8;NEDD4L;LPP;ARID1B;* |

| | | | |
|---|---|---|---|
| | | 079E-6 | *MED13L;CDK6;NFIA;TBL1XR1;NFIB;UBR5;BBX;APBB2* |
| FOXK1 | 15/299 | 1.679936341713079E-6 | *RERE;ZFHX3;CAMK2D;KMT2A;ZMYND8;NEDD4L;LPP;ARID1B;MED13L;TANC1;CDK6;NFIA;TBL1XR1;NFIB;DLC1* |
| GATAD2B | 15/299 | 1.679936341713079E-6 | *RERE;NCOA2;ZFHX3;KLF12;KMT2A;ZMYND8;LPP;ARID1B;MED13L;KLF7;CDK6;NFIA;TBL1XR1;NFIB;BBX* |
| GLIS1 | 15/299 | 1.679936341713079E-6 | *ZFHX3;EYA2;COL12A1;WNT5A;CACNA1C;EFNA5;AFF3;FBLN2;ESR1;NR3C2;NFIA;NFIB;DLC1;PPARG;HMCN1* |
| HDX | 15/299 | 1.679936341713079E-6 | *KLF12;WNT5A;MX1;PRICKLE2;ETV1;SLC7A11;IKZF2;EFNA5;CDK6;NFIA;NFIB;DLC1;BBX;HMCN1;EPHA3* |
| HIVEP3 | 15/299 | 1.679936341713079E-6 | *RERE;ZFHX3;BCL11A;NEDD4L;SORBS2;CACNA1C;EFNA5;AFF3;LPP;KLF7;CDK6;FMNL2;NFIA;NFIB;DLC1* |
| IKZF4 | 15/299 | 1.679936341713079E-6 | *ZFHX3;KLF12;KMT2A;BCL11A;NEDD4L;ETV1;SLC7A11;IKZF2;AFF3;LPP;KLF7;CDK6;NFIA;NFIB;DLC1* |
| MITF | 15/299 | 1.679936341713079E-6 | *ZFHX3;THRB;ANGPT1;DAPK1;NEDD4L;ETV1;SORBS2;SLC7A11;LPP;ESR1;CDK6;NFIA;NFIB;DLC1;PPARG* |
| NFIA | 15/299 | 1.679936341713079E-6 | *RERE;ZFHX3;THRB;BCL11A;NEDD4L;ETV1;SORBS2;EFNA5;AFF3;LPP;ARID1B;CDK6;TBL1XR1;NFIB;DLC1* |
| NR2C1 | 15/299 | 1.679936341713079E-6 | *NCOA2;ZFHX3;KLF12;CAMK2D;DAPK1;BCL11A;ZMYND8;NEDD4L;LPP;ESR1;CDK6;NFIA;TBL1XR1;NFIB;DLC1* |
| NR2E3 | 15/299 | 1.679936341713079E-6 | *ZFHX3;THRB;BCL11A;WNT5A;ETV1;SLC7A11;AFF3;FBLN2;ESR1;NFIA;NFIB;DLC1;RARB;PPARG;EPHA3* |
| PAX6 | 15/299 | 1.679936341713079E-6 | *ZFHX3;BCL11A;WNT5A;ETV1;LHFPL3;SLC7A11;EFNA5;ESR1;NPAS3;MAPK10;CDK6;NFIA;NFIB;DLC1;PPARG* |
| PHF21A | 15/299 | 1.679936341713079E-6 | *RERE;NCOA2;ZFHX3;KMT2A;BCL11A;ZMYND8;NEDD4L;LPP;ARID1B;MED13L;KLF7;NFIA;TBL1XR1;NFIB;BBX* |
| PLAG1 | 15/299 | 1.679936341713079E-6 | *ZFHX3;KLF12;ANGPT1;BCL11A;WNT5A;ETV1;SLC7A11;IKZF2;LPP;NR3C2;CDK6;NFIA;NFIB;DLC1;PPARG* |
| RBM20 | 15/299 | 1.679936341713079E-6 | *AFAP1L2;WNT5A;AKAP6;SORBS2;CACNA1C;EFNA5;TTN;CDK6;NFIA;NFIB;ALDH1A2;DLC1;MPPED2;RARB;HMCN1* |
| SHOX2 | 15/299 | 1.679936341713079E-6 | *ZFHX3;ANGPT1;BCL11A;COL12A1;WNT5A;ETV1;SLC7A11;EFNA5;AFF3;NFIA;NFIB;ALDH1A2;DLC1;PPARG;EPHA3* |
| TFAP2D | 15/299 | 1.679936341713079E-6 | *THRB;BCL11A;WNT5A;LHFPL3;ESR1;NFIA;ADGRB3;CHL1;NFIB;ALDH1A2;DLC1;KCNQ5;PPARG;EPHA3;HCN1* |
| ZBTB37 | 15/299 | 1.679936341713079E-6 | *NCOA2;ZFHX3;KLF12;KMT2A;SLC7A11;IKZF2;LPP;MED13L;KLF7;CDK6;NFIA;TBL1XR1;NFIB;DLC1;BBX* |
| ZFP14 | 15/299 | 1.679936341713079E-6 | *ZFHX3;KLF12;THRB;PELI2;GDPD1;SLC7A11;IKZF2;EFNA5;AFF3;LPP;NR3C2;TTC28;NFIA;NFIB;DLC1* |
| ZNF214 | 15/299 | 1.679936341713079E-6 | *ZFHX3;KLF12;COL12A1;SHROOM3;ETV1;SORBS2;AFF3;ESR1;NR3C2;MAPK10;EPB41L4A;NFIA;DLC1;RARB;HMCN1* |
| ZNF229 | 15/299 | 1.679936341713079E-6 | *ZFHX3;KLF12;RASGRF2;COL12A1;TMTC1;SORBS2;LHFPL3;SLC7A11;AFF3;FBLN2;ZNF607;NFIB;DLC1;HMCN1;ZNF234* |
| ZNF254 | 15/299 | 1.679936341713079E-6 | *ZFHX3;KLF12;PELI2;NEDD4L;VPS13B;SLC7A11;LPP;MBTD1;CDK6;NFIA;TBL1XR1;NFIB;ZNF429;DLC1;BBX* |
| ZNF280D | 15/299 | 1.679936341713079E-6 | *RERE;ZFHX3;CAMK2D;KMT2A;BCL11A;ZMYND8;SLC7A11;LPP;MBTD1;NFIA;TBL1XR1;NFIB;DLC1;UBR5;BBX* |
| ZNF491 | 15/299 | 1.679936341713079E-6 | *ZFHX3;KLF12;LHFPL3;IKZF2;AFF3;LPP;CDK6;ZNF607;NFIA;NFIB;ZNF429;DLC1;ZNF615;SRGAP3;CFAP44* |
| ZNF540 | 15/299 | 1.679936341713 | *ST8SIA1;BCL11A;RASGRF2;AKAP6;SORBS2;LHFPL3* |

| | | | |
|---|---|---|---|
| | | 079E-6 | *;LDLRAD4;AFF3;NR3C2;GNAO1;MAPK10;ZNF607;NFIA;NFIB;DLC1* |
| ZNF568 | 15/299 | 1.679936341713079E-6 | *ZFHX3;KLF12;ZMYND8;LHFPL3;AFF3;NPAS3;KLF7;CDK6;ZNF607;NFIA;NFIB;ZNF429;DLC1;ZNF615;ZNF234* |
| ZNF611 | 15/299 | 1.679936341713079E-6 | *NCOA2;ZFHX3;KLF12;ATP8B1;NEDD4L;VPS13B;LPP;MED13L;CDK6;NFIA;NFIB;DLC1;BBX;APBB2;ZNF665* |
| ZNF709 | 15/299 | 1.679936341713079E-6 | *ZFHX3;KLF12;BCL11A;TMTC1;IKZF2;AFF3;ESR1;EPB41L4A;ZNF607;NFIA;NFIB;ZNF429;DLC1;ZNF615;ZNF665* |
| ZNF781 | 15/299 | 1.679936341713079E-6 | *ZFHX3;ANGPT1;BCL11A;FIGN;AKAP6;SORBS2;LHFPL3;AFF3;ZNF607;NFIB;ZNF429;DLC1;MPPED2;ZNF615;ZNF665* |
| AEBP2 | 14/299 | 7.220256215310226E-6 | *RERE;ZFHX3;CAMK2D;ZMYND8;NEDD4L;LPP;ARID1B;MED13L;CDK6;NFIA;TBL1XR1;NFIB;DLC1;BBX* |
| ALX4 | 14/299 | 7.220256215310226E-6 | *ZFHX3;THRB;EYA2;COL12A1;WNT5A;LHFPL3;EFNA5;AFF3;FBLN2;ESR1;NFIA;NFIB;ALDH1A2;DLC1* |
| ARID2 | 14/299 | 7.220256215310226E-6 | *RERE;NCOA2;ZFHX3;KMT2A;ZMYND8;LPP;ARID1B;MED13L;CDK6;NFIA;TBL1XR1;NFIB;UBR5;BBX* |
| ASH1L | 14/299 | 7.220256215310226E-6 | *RERE;NCOA2;ZFHX3;KMT2A;BCL11A;VPS13B;LPP;ARID1B;MED13L;NFIA;TBL1XR1;NFIB;UBR5;BBX* |
| CUX2 | 14/299 | 7.220256215310226E-6 | *ZFHX3;BCL11A;NEDD4L;SORBS2;LHFPL3;CACNA1C;AFF3;GNAO1;NFIA;NFIB;DLC1;MPPED2;KCNQ5;DLGAP1* |
| DLX1 | 14/299 | 7.220256215310226E-6 | *ZFHX3;THRB;BCL11A;COL12A1;WNT5A;ETV1;SLC7A11;AFF3;NFIA;NFIB;ALDH1A2;DLC1;RARB;KCNQ5* |
| DMRTA1 | 14/299 | 7.220256215310226E-6 | *THRB;BCL11A;EYA2;COL12A1;WNT5A;ETV1;LHFPL3;SLC7A11;EFNA5;AFF3;CLDN1;NFIA;DLC1;EPHA3* |
| EBF1 | 14/299 | 7.220256215310226E-6 | *ZFHX3;ANGPT1;BCL11A;COL12A1;WNT5A;TMTC1;EFNA5;AFF3;FBLN2;NFIA;NFIB;DLC1;PPARG;EPHA3* |
| EBF3 | 14/299 | 7.220256215310226E-6 | *ZFHX3;ANGPT1;BCL11A;COL12A1;WNT5A;EFNA5;AFF3;NFIA;NFIB;ALDH1A2;DLC1;MPPED2;PPARG;EPHA3* |
| EP400 | 14/299 | 7.220256215310226E-6 | *RERE;NCOA2;ZFHX3;KMT2A;ZMYND8;NEDD4L;VPS13B;LPP;ARID1B;MED13L;NFIA;TBL1XR1;NFIB;UBR5* |
| ETV6 | 14/299 | 7.220256215310226E-6 | *RERE;ZFHX3;BCL11A;ZMYND8;NEDD4L;LPP;ARID1B;MED13L;KLF7;CDK6;NFIA;TBL1XR1;NFIB;DLC1* |
| FBXO41 | 14/299 | 7.220256215310226E-6 | *GABBR1;BCL11A;PRICKLE2;LHFPL3;CACNA1C;AFF3;GNAO1;MAPK10;CACNB4;ADGRB3;NFIB;DLGAP1;SRGAP3;RAPGEF4* |
| FOXJ2 | 14/299 | 7.220256215310226E-6 | *RERE;NCOA2;ZFHX3;KMT2A;BCL11A;NEDD4L;LPP;ARID1B;MED13L;KLF7;NFIA;NFIB;DLC1;BBX* |
| GLI2 | 14/299 | 7.220256215310226E-6 | *ZFHX3;COL12A1;WNT5A;ETV1;EFNA5;AFF3;ESR1;CDK6;NFIA;NFIB;DLC1;PPARG;HMCN1;EPHA3* |
| JAZF1 | 14/299 | 7.220256215310226E-6 | *ZFHX3;KLF12;BCL11A;PRICKLE2;NEDD4L;SLC7A11;EFNA5;AFF3;LPP;CDK6;NFIA;NFIB;DLC1;CDK14* |
| LHX9 | 14/299 | 7.220256215310226E-6 | *ZFHX3;BCL11A;COL12A1;WNT5A;ETV1;EFNA5;AFF3;ESR1;NFIA;NFIB;ALDH1A2;DLC1;HMCN1;EPHA3* |
| LIN28B | 14/299 | 7.220256215310226E-6 | *ZFHX3;BCL11A;WNT5A;ETV1;FIGN;LHFPL3;SLC7A11;EFNA5;CECR2;CDK6;NFIA;NFIB;DLC1;EPHA3* |
| LMX1B | 14/299 | 7.220256215310226E-6 | *ZFHX3;BCL11A;EYA2;WNT5A;LHFPL3;CACNA1C;EFNA5;AFF3;ESR1;NPAS3;NFIA;NFIB;ALDH1A2;DLC1* |
| MECP2 | 14/299 | 7.220256215310226E-6 | *RERE;ZFHX3;KMT2A;ZMYND8;NEDD4L;LPP;ESR1;ARID1B;MED13L;KLF7;CDK6;NFIA;NFIB;DLC1* |
| MEIS2 | 14/299 | 7.220256215310226E-6 | *ZFHX3;BCL11A;COL12A1;WNT5A;ETV1;SORBS2;LAMB1;EFNA5;CDK6;NFIA;NFIB;DLC1;MPPED2;RARB* |
| MGA | 14/299 | 7.220256215310226E-6 | *RERE;NCOA2;ZFHX3;KMT2A;ZMYND8;VPS13B;LPP;ARID1B;MED13L;TBL1XR1;NFIB;UBR5;DMXL2;BBX* |
| MKRN3 | 14/299 | 7.220256215310226E-6 | *ZFHX3;BCL11A;EYA2;TMTC1;ETV1;LHFPL3;SLC7A11;CDK6;NFIA;CHL1;NFIB;ALDH1A2;DLC1;EPHA3* |
| MTA3 | 14/299 | 7.220256215310226E-6 | *RERE;ZFHX3;BCL11A;ZMYND8;NEDD4L;AFF3;LPP;ARID1B;TANC1;CDK6;NFIA;TBL1XR1;NFIB;DLC1* |

| | | | |
|---|---|---|---|
| NRF1 | 14/299 | 7.220256215310226E-6 | *RERE;NCOA2;ZFHX3;KMT2A;ZMYND8;NEDD4L;ARID1B;MED13L;KLF7;CDK6;NFIA;TBL1XR1;NFIB;DLC1* |
| ONECUT2 | 14/299 | 7.220256215310226E-6 | *ZFHX3;BCL11A;COL12A1;WNT5A;NEDD4L;ETV1;LHFPL3;SLC7A11;EFNA5;CDK6;NFIA;NFIB;DLC1;RARB* |
| PROX2 | 14/299 | 7.220256215310226E-6 | *ZFHX3;GABBR1;PDE1C;LHFPL3;SLC7A11;EFNA5;ESR1;NR3C2;TTC28;TTN;NFIA;DLC1;RARB;RAPGEF4* |
| RAG1 | 14/299 | 7.220256215310226E-6 | *ZFHX3;KLF12;BCL11A;IL1RAP;IKZF2;AFF3;LPP;ESR1;NR3C2;TTN;MAPK10;CDK6;NFIA;DLC1* |
| RCOR1 | 14/299 | 7.220256215310226E-6 | *NCOA2;ZFHX3;KMT2A;ZMYND8;NEDD4L;LPP;ARID1B;MED13L;CDK6;NFIA;TBL1XR1;NFIB;DLC1;UBR5* |
| RCOR3 | 14/299 | 7.220256215310226E-6 | *RERE;NCOA2;ZFHX3;KLF12;CAMK2D;KMT2A;BCL11A;ZMYND8;NEDD4L;CDK6;NFIA;TBL1XR1;NFIB;BBX* |
| REST | 14/299 | 7.220256215310226E-6 | *NCOA2;ZFHX3;KMT2A;LPP;ESR1;MED13L;KLF7;CDK6;NFIA;TBL1XR1;NFIB;DLC1;UBR5;BBX* |
| RFX4 | 14/299 | 7.220256215310226E-6 | *ZFHX3;MEGF10;BCL11A;LHFPL3;SLC7A11;EFNA5;ESR1;NPAS3;NFIA;NFIB;DLC1;RARB;DLGAP1;SRGAP3* |
| RGS7 | 14/299 | 7.220256215310226E-6 | *RGS17;BCL11A;SORBS2;LHFPL3;CACNA1C;GNAO1;MAPK10;ADGRB3;CHL1;DLC1;KCNQ5;DLGAP1;RAPGEF4;HCN1* |
| SMAD4 | 14/299 | 7.220256215310226E-6 | *RERE;KMT2A;NEDD4L;LPP;ESR1;ARID1B;MED13L;CDK6;NFIA;TBL1XR1;NFIB;DLC1;UBR5;PPARG* |
| SMAD9 | 14/299 | 7.220256215310226E-6 | *ZFHX3;THRB;ANGPT1;COL12A1;WNT5A;TMTC1;SORBS2;SLC7A11;AFF3;NFIA;NFIB;DLC1;PPARG;HMCN1* |
| TBR1 | 14/299 | 7.220256215310226E-6 | *ZFHX3;BCL11A;LHFPL3;ARID1B;NR3C2;MED13L;NFIA;TBL1XR1;NFIB;DLC1;DLGAP1;SRGAP3;RAPGEF4;HCN1* |
| TBX15 | 14/299 | 7.220256215310226E-6 | *ZFHX3;ANGPT1;COL12A1;WNT5A;SORBS2;LAMB1;FBLN2;NFIA;NFIB;ALDH1A2;DLC1;PPARG;HMCN1;EPHA3* |
| TBX20 | 14/299 | 7.220256215310226E-6 | *BCL11A;COL12A1;WNT5A;ETV1;SORBS2;LHFPL3;CACNA1C;EFNA5;TTN;NFIA;DLC1;RARB;PPARG;HMCN1* |
| TCF4 | 14/299 | 7.220256215310226E-6 | *RERE;ZFHX3;BCL11A;NEDD4L;ETV1;LPP;ARID1B;KLF7;CDK6;NFIA;TBL1XR1;NFIB;DLC1;CDK14* |
| TFAP2A | 14/299 | 7.220256215310226E-6 | *ZFHX3;BCL11A;COL12A1;WNT5A;ETV1;SLC7A11;EFNA5;CLDN1;ESR1;CDK6;NFIA;NFIB;DLC1;PPARG* |
| TSHZ3 | 14/299 | 7.220256215310226E-6 | *ZFHX3;BCL11A;COL12A1;WNT5A;PRICKLE2;CACNA1C;EFNA5;AFF3;FBLN2;NFIA;NFIB;DLC1;HMCN1;EPHA3* |
| ZFP64 | 14/299 | 7.220256215310226E-6 | *ZFHX3;KLF12;BCL11A;ZMYND8;NEDD4L;SHROOM3;EFNA5;AFF3;ARID1B;CDK6;NFIA;TBL1XR1;NFIB;DLC1* |
| ZMAT4 | 14/299 | 7.220256215310226E-6 | *BCL11A;ETV1;SORBS2;LHFPL3;EFNA5;ADGRB3;DLC1;MPPED2;KCNQ3;RARB;KCNQ5;DLGAP1;CDK14;EPHA3* |
| ZNF148 | 14/299 | 7.220256215310226E-6 | *RERE;NCOA2;ZFHX3;KMT2A;ZMYND8;LPP;ARID1B;MED13L;CDK6;NFIA;TBL1XR1;NFIB;UBR5;BBX* |
| ZNF154 | 14/299 | 7.220256215310226E-6 | *ZFHX3;KLF12;PDE1C;BCL11A;PRICKLE2;LHFPL3;IKZF2;AFF3;LPP;TTN;MAPK10;NFIA;NFIB;DLC1* |
| ZNF248 | 14/299 | 7.220256215310226E-6 | *ZFHX3;KLF12;KMT2A;BCL11A;NEDD4L;SLC7A11;IKZF2;AFF3;LPP;NR3C2;CDK6;NFIA;NFIB;DLC1* |
| ZNF292 | 14/299 | 7.220256215310226E-6 | *RERE;NCOA2;ZFHX3;KMT2A;ZMYND8;VPS13B;LPP;ARID1B;MED13L;MBTD1;TBL1XR1;NFIB;UBR5;BBX* |
| ZNF318 | 14/299 | 7.220256215310226E-6 | *RERE;NCOA2;ZFHX3;KMT2A;BCL11A;ZMYND8;NEDD4L;VPS13B;LPP;ARID1B;MED13L;NFIA;UBR5;APBB2* |
| ZNF334 | 14/299 | 7.220256215310226E-6 | *ANGPT1;BCL11A;NEDD4L;TMTC1;ETV1;SORBS2;LHFPL3;CLDN1;ACACB;NFIB;DLC1;MPPED2;PPARG;ZNF521* |
| ZNF391 | 14/299 | 7.220256215310226E-6 | *THRB;BCL11A;GDPD1;TMTC2;ETV1;AKAP6;SLC7A11;KLF7;CDK6;NFIA;NFIB;DLC1;KCNQ5;SRGAP3* |
| ZNF423 | 14/299 | 7.220256215310226E-6 | *ZFHX3;BCL11A;SORBS2;LHFPL3;CACNA1C;AFF3;FBLN2;NR3C2;ADGRD1;NFIA;NFIB;DLC1;HMCN1;ZNF521* |

| | | | |
|---|---|---|---|
| ZNF429 | 14/299 | 7.220256215310226E-6 | *KLF12;THRB;BCL11A;NEDD4L;SORBS2;SLC7A11;IKZF2;LPP;MAPK10;ZNF607;NFIA;NFIB;DLC1;ZNF615* |
| ZNF445 | 14/299 | 7.220256215310226E-6 | *RERE;ZFHX3;KLF12;KMT2A;NEDD4L;LPP;ARID1B;MED13L;TTC28;KLF7;CDK6;TBL1XR1;NFIB;DLC1* |
| ZNF555 | 14/299 | 7.220256215310226E-6 | *KLF12;ZMYND8;MX1;GDPD1;SLC7A11;AFF3;KLF7;CDK6;ZNF607;NFIA;ZNF429;DLC1;ZNF615;ZNF234* |
| ZNF594 | 14/299 | 7.220256215310226E-6 | *ZFHX3;KLF12;KMT2A;DAPK1;BCL11A;NEDD4L;SLC7A11;MBTD1;ZNF607;NFIA;ZNF429;DLC1;ZNF615;ZNF234* |
| ZNF676 | 14/299 | 7.220256215310226E-6 | *COL12A1;ETV1;SORBS2;LHFPL3;TTN;NFIA;ADGRB3;CHL1;NFIB;ZNF429;ALDH1A2;DLC1;HMCN1;EPHA3* |
| ZNF677 | 14/299 | 7.220256215310226E-6 | *THRB;PDE1C;DAPK1;BCL11A;TMTC1;LHFPL3;NR3C2;CHL1;NFIB;ZNF429;DLC1;MPPED2;ZNF665;ZNF521* |
| ZNF699 | 14/299 | 7.220256215310226E-6 | *ZFHX3;KLF12;CAMK2D;COL12A1;NEDD4L;LAMB1;IL1RAP;SLC7A11;LPP;KLF7;CDK6;NFIB;DLC1;BBX* |
| ZNF717 | 14/299 | 7.220256215310226E-6 | *ZFHX3;DAPK1;COL12A1;NEDD4L;SORBS2;LHFPL3;EFNA5;AFF3;NR3C2;TTN;DLC1;HMCN1;ZNF521;EPHA3* |
| ZNF808 | 14/299 | 7.220256215310226E-6 | *RERE;ZFHX3;KMT2A;MX1;NEDD4L;VPS13B;TMTC1;LDLRAD4;CDK6;ZNF607;ZNF429;DLC1;ZNF615;ZNF234* |
| ZNF84 | 14/299 | 7.220256215310226E-6 | *KLF12;KMT2A;BCL11A;NEDD4L;SORBS2;LPP;CDK6;NFIA;TBL1XR1;NFIB;DLC1;DMXL2;ZNF615;BBX* |
| ZXDA | 14/299 | 7.220256215310226E-6 | *ZFHX3;KLF12;MAOA;BCL11A;PELI2;NEDD4L;TMTC2;SLC7A11;LPP;NR3C2;NFIA;NFIB;DLC1;BBX* |
| AFF1 | 13/299 | 3.037194265059546E-5 | *RERE;NCOA2;ZFHX3;ZMYND8;NEDD4L;LPP;ARID1B;MED13L;CDK6;NFIA;NFIB;DLC1;BBX* |
| ATOH1 | 13/299 | 3.037194265059546E-5 | *ZFHX3;BCL11A;EYA2;ETV1;LHFPL3;EFNA5;ESR1;NR3C2;NPAS3;NFIA;NFIB;DLC1;PPARG* |
| BARX2 | 13/299 | 3.037194265059546E-5 | *ZFHX3;THRB;BCL11A;EYA2;COL12A1;SLC7A11;EFNA5;ESR1;NFIA;NFIB;ALDH1A2;DLC1;PPARG* |
| BCL11B | 13/299 | 3.037194265059546E-5 | *ZFHX3;KLF12;BCL11A;NEDD4L;SLC7A11;EFNA5;AFF3;KBTBD11;CDK6;NFIA;NFIB;DLC1;MPPED2* |
| CASZ1 | 13/299 | 3.037194265059546E-5 | *RERE;ZFHX3;BCL11A;NEDD4L;SHROOM3;SORBS2;CACNA1C;EFNA5;AFF3;ARID1B;NFIA;NFIB;DLC1* |
| CHD2 | 13/299 | 3.037194265059546E-5 | *RERE;ZFHX3;KMT2A;BCL11A;ZMYND8;LPP;ARID1B;MED13L;NFIA;TBL1XR1;NFIB;UBR5;BBX* |
| CLOCK | 13/299 | 3.037194265059546E-5 | *NCOA2;ZFHX3;KLF12;KMT2A;NEDD4L;LPP;KLF7;CDK6;NFIA;TBL1XR1;NFIB;DLC1;BBX* |
| DACH2 | 13/299 | 3.037194265059546E-5 | *ANGPT1;LHFPL3;CACNA1C;ADGRB3;CHL1;ALDH1A2;DLC1;RARB;KCNQ5;DLGAP1;HMCN1;PAK3;EPHA3* |
| DEPDC5 | 13/299 | 3.037194265059546E-5 | *RERE;NCOA2;ZFHX3;DAPK1;ZMYND8;NEDD4L;VPS13B;SORBS2;ASAP2;LPP;ARID1B;DLC1;APBB2* |
| DOT1L | 13/299 | 3.037194265059546E-5 | *RERE;KMT2A;BCL11A;ZMYND8;NEDD4L;ARID1B;MED13L;CDK6;NFIA;TBL1XR1;NFIB;DLC1;SRGAP3* |
| ELK4 | 13/299 | 3.037194265059546E-5 | *RERE;NCOA2;ZFHX3;KLF12;KMT2A;LPP;MED13L;KLF7;CDK6;NFIA;TBL1XR1;NFIB;BBX* |
| ETV1 | 13/299 | 3.037194265059546E-5 | *BCL11A;WNT5A;SORBS2;SLC7A11;EFNA5;CDK6;NFIA;NFIB;DLC1;PPARG;APBB2;CDK14;EPHA3* |
| FAM171B | 13/299 | 3.037194265059546E-5 | *COL12A1;PRICKLE2;ETV1;SORBS2;SLC7A11;GULP1;NFIA;CHL1;NFIB;DLC1;HMCN1;CDK14;EPHA3* |
| HAND2 | 13/299 | 3.037194265059546E-5 | *ANGPT1;COL12A1;WNT5A;CACNA1C;FBLN2;NFIA;NFIB;ALDH1A2;DLC1;MPPED2;RARB;PPARG;EPHA3* |
| HIVEP1 | 13/299 | 3.037194265059546E-5 | *RERE;NCOA2;ZFHX3;KMT2A;NEDD4L;SLC7A11;LPP;MED13L;KLF7;CDK6;NFIA;NFIB;DLC1* |
| HKR1 | 13/299 | 3.037194265059546E-5 | *ZFHX3;KLF12;NEDD4L;SHROOM3;SORBS2;LPP;ESR1;ARID1B;KLF7;NFIA;NFIB;DLC1;ZNF615* |
| HOXA3 | 13/299 | 3.037194265059546E-5 | *ZFHX3;ANGPT1;BCL11A;COL12A1;WNT5A;LAMB1;CDK6;NFIA;NFIB;ALDH1A2;DLC1;RARB;PPARG* |
| HOXD4 | 13/299 | 3.037194265059 | *ZFHX3;EYA2;COL12A1;WNT5A;ETV1;LHFPL3;NFIA;* |

| | | | |
|---|---|---|---|
| | | 546E-5 | *NFIB;ALDH1A2;DLC1;RARB;PPARG;EPHA3* |
| MACF1 | 13/299 | 3.037194265059 546E-5 | *RERE;ZFHX3;KMT2A;ZMYND8;NEDD4L;SORBS2;LPP;CDK6;NFIA;NFIB;DLC1;UBR5;APBB2* |
| MEF2A | 13/299 | 3.037194265059 546E-5 | *RERE;ZFHX3;CAMK2D;NEDD4L;LPP;ARID1B;MED13L;CDK6;NFIA;TBL1XR1;NFIB;DLC1;CDK14* |
| MEIS1 | 13/299 | 3.037194265059 546E-5 | *ZFHX3;ANGPT1;BCL11A;WNT5A;ETV1;SORBS2;EFNA5;LPP;CDK6;NFIA;NFIB;DLC1;RARB* |
| MLXIP | 13/299 | 3.037194265059 546E-5 | *RERE;ZFHX3;KMT2A;ZMYND8;NEDD4L;LPP;ARID1B;MED13L;KLF7;CDK6;NFIA;NFIB;DLC1* |
| NEUROG2 | 13/299 | 3.037194265059 546E-5 | *ZFHX3;BCL11A;EYA2;WNT5A;LHFPL3;EFNA5;AFF3;NFIA;CHL1;NFIB;ALDH1A2;DLC1;EPHA3* |
| NFAT5 | 13/299 | 3.037194265059 546E-5 | *RERE;NCOA2;ZFHX3;KMT2A;VPS13B;LPP;MED13L;CDK6;NFIA;TBL1XR1;NFIB;DLC1;BBX* |
| NFIB | 13/299 | 3.037194265059 546E-5 | *ZFHX3;BCL11A;COL12A1;NEDD4L;ETV1;SORBS2;EFNA5;LPP;ARID1B;CDK6;NFIA;TBL1XR1;DLC1* |
| NRK | 13/299 | 3.037194265059 546E-5 | *PDE1C;ANGPT1;COL12A1;WNT5A;SLC7A11;EFNA5;CLDN1;FBLN2;NFIA;NFIB;DLC1;HMCN1;EPHA3* |
| PAX9 | 13/299 | 3.037194265059 546E-5 | *THRB;BCL11A;EYA2;COL12A1;WNT5A;SLC7A11;EFNA5;ESR1;NFIA;NFIB;ALDH1A2;DLC1;PPARG* |
| POU3F2 | 13/299 | 3.037194265059 546E-5 | *ZFHX3;BCL11A;WNT5A;ETV1;LHFPL3;EFNA5;NPAS3;CDK6;NFIA;CHL1;NFIB;DLC1;EPHA3* |
| POU3F3 | 13/299 | 3.037194265059 546E-5 | *ZFHX3;BCL11A;WNT5A;ETV1;LHFPL3;EFNA5;AFF3;NPAS3;NFIA;CHL1;NFIB;DLC1;MPPED2* |
| POU3F4 | 13/299 | 3.037194265059 546E-5 | *ZFHX3;WNT5A;ETV1;LHFPL3;NPAS3;GULP1;NFIA;CHL1;NFIB;ALDH1A2;DLC1;RARB;EPHA3* |
| POU4F1 | 13/299 | 3.037194265059 546E-5 | *ZFHX3;KLF12;BCL11A;EYA2;LHFPL3;SLC7A11;EFNA5;AFF3;ESR1;CDK6;NFIA;NFIB;DLC1* |
| PRDM2 | 13/299 | 3.037194265059 546E-5 | *RERE;NCOA2;ZFHX3;KMT2A;ZMYND8;NEDD4L;LPP;ARID1B;MED13L;NFIA;NFIB;DLC1;BBX* |
| RC3H1 | 13/299 | 3.037194265059 546E-5 | *RERE;NCOA2;ZFHX3;KMT2A;LPP;ARID1B;MED13L;NFIA;TBL1XR1;NFIB;DLC1;UBR5;BBX* |
| SHOX | 13/299 | 3.037194265059 546E-5 | *ZFHX3;PDE1C;BCL11A;LHFPL3;CACNA1C;ESR1;NR3C2;NFIA;NFIB;DLC1;RARB;PPARG;HCN1* |
| SHPRH | 13/299 | 3.037194265059 546E-5 | *NCOA2;KLF12;KMT2A;VPS13B;LPP;ARID1B;MED13L;MBTD1;CDK6;TBL1XR1;UBR5;BBX;SBF2* |
| SIM2 | 13/299 | 3.037194265059 546E-5 | *ZFHX3;BCL11A;EYA2;COL12A1;WNT5A;SLC7A11;EFNA5;AFF3;ESR1;NFIA;NFIB;DLC1;PPARG* |
| TCF20 | 13/299 | 3.037194265059 546E-5 | *RERE;NCOA2;ZFHX3;KMT2A;BCL11A;ZMYND8;NEDD4L;ARID1B;MED13L;NFIA;TBL1XR1;NFIB;UBR5* |
| TFDP2 | 13/299 | 3.037194265059 546E-5 | *RERE;ZFHX3;KLF12;BCL11A;ZMYND8;NEDD4L;LPP;CDK6;NFIA;TBL1XR1;NFIB;DLC1;BBX* |
| TIGD6 | 13/299 | 3.037194265059 546E-5 | *ZFHX3;THRB;AKAP6;IL1RAP;SLC7A11;AFF3;LPP;KLF7;EPB41L4A;CDK6;NFIA;NFIB;DLC1* |
| WNT8B | 13/299 | 3.037194265059 546E-5 | *ZFHX3;EYA2;WNT5A;PELI2;SORBS2;LHFPL3;CACNA1C;EFNA5;AFF3;ALDH1A2;DLC1;RARB;EPHA3* |
| ZBTB10 | 13/299 | 3.037194265059 546E-5 | *ZFHX3;KLF12;PELI2;NEDD4L;SLC7A11;LPP;MED13L;KLF7;CDK6;NFIA;TBL1XR1;NFIB;DLC1* |
| ZBTB38 | 13/299 | 3.037194265059 546E-5 | *ZFHX3;KLF12;ZMYND8;NEDD4L;SLC7A11;LPP;KLF7;CDK6;NFIA;TBL1XR1;NFIB;DLC1;BBX* |
| ZBTB40 | 13/299 | 3.037194265059 546E-5 | *RERE;ZFHX3;KLF12;KMT2A;ZMYND8;NEDD4L;LPP;ARID1B;MED13L;CTIF;CDK6;NFIA;NFIB* |
| ZBTB7C | 13/299 | 3.037194265059 546E-5 | *ZFHX3;ATP8B1;EYA2;COL12A1;WNT5A;NEDD4L;SHROOM3;EFNA5;FBLN2;NR3C2;NFIA;NFIB;DLC1* |
| ZFP30 | 13/299 | 3.037194265059 546E-5 | *ZFHX3;KLF12;BCL11A;WNT5A;GDPD1;SLC7A11;IKZF2;KBTBD11;KLF7;CDK6;NFIA;NFIB;DLC1* |
| ZHX2 | 13/299 | 3.037194265059 546E-5 | *RERE;ZFHX3;DAPK1;BCL11A;ZMYND8;NEDD4L;SLC7A11;EFNA5;LPP;CDK6;NFIA;NFIB;DLC1* |
| ZHX3 | 13/299 | 3.037194265059 546E-5 | *RERE;ZFHX3;KLF12;ZMYND8;NEDD4L;LPP;ACACB;TANC1;CDK6;NFIA;NFIB;DLC1;APBB2* |

| | | | |
|---|---|---|---|
| ZIC1 | 13/299 | 3.037194265059546E-5 | *ZFHX3;BCL11A;COL12A1;WNT5A;ETV1;LHFPL3;SLC7A11;EFNA5;NFIA;NFIB;ALDH1A2;DLC1;EPHA3* |
| ZIC5 | 13/299 | 3.037194265059546E-5 | *ZFHX3;BCL11A;COL12A1;WNT5A;ETV1;LHFPL3;SLC7A11;EFNA5;CECR2;CDK6;NFIA;NFIB;DLC1* |
| ZNF132 | 13/299 | 3.037194265059546E-5 | *THRB;BCL11A;WNT5A;MX1;SORBS2;LHFPL3;AFF3;NR3C2;ZNF607;DLC1;ZNF615;ZNF665;ZNF234* |
| ZNF233 | 13/299 | 3.037194265059546E-5 | *THRB;PRICKLE2;NEDD4L;SLC7A11;AFF3;TTN;EPB41L4A;NFIA;NFIB;ZNF429;DLC1;ZNF615;ZNF234* |
| ZNF235 | 13/299 | 3.037194265059546E-5 | *ZFHX3;KLF12;DAPK1;ZMYND8;ETV1;SLC7A11;IKZF2;AFF3;CDK6;NFIA;NFIB;DLC1;ZNF234* |
| ZNF345 | 13/299 | 3.037194265059546E-5 | *KLF12;KMT2A;BCL11A;IKZF2;MBTD1;KLF7;ZNF607;NFIA;ZNF429;DLC1;ZNF615;ZNF234;CFAP44* |
| ZNF440 | 13/299 | 3.037194265059546E-5 | *KLF12;THRB;ATP8B1;IKZF2;LPP;KLF7;CDK6;ZNF607;NFIA;ZNF429;DLC1;ZNF615;ZNF234* |
| ZNF483 | 13/299 | 3.037194265059546E-5 | *KLF12;BCL11A;PRICKLE2;TMTC1;ETV1;LHFPL3;NR3C2;GULP1;GNAO1;CHL1;NFIB;DLC1;PAK3* |
| ZNF518B | 13/299 | 3.037194265059546E-5 | *ZFHX3;KLF12;BCL11A;WNT5A;PELI2;TMTC1;LPP;TANC1;CDK6;NFIA;DLC1;HMCN1;ZNF521* |
| ZNF610 | 13/299 | 3.037194265059546E-5 | *THRB;BCL11A;MX1;GDPD1;TMTC2;TMTC1;SLC7A11;AFF3;ZNF607;ZNF429;DLC1;ZNF615;ZNF665* |
| ZNF621 | 13/299 | 3.037194265059546E-5 | *ZFHX3;KLF12;KMT2A;NEDD4L;SLC7A11;LPP;KLF7;CDK6;NFIA;TBL1XR1;NFIB;BBX;APBB2* |
| ZNF644 | 13/299 | 3.037194265059546E-5 | *RERE;KLF12;KMT2A;BCL11A;LPP;ARID1B;MED13L;CDK6;NFIA;TBL1XR1;NFIB;UBR5;BBX* |
| ZNF652 | 13/299 | 3.037194265059546E-5 | *NCOA2;ZFHX3;KMT2A;NEDD4L;SLC7A11;LPP;MED13L;MBTD1;CDK6;NFIA;TBL1XR1;NFIB;BBX* |
| ZNF667 | 13/299 | 3.037194265059546E-5 | *THRB;DAPK1;BCL11A;PRICKLE2;NEDD4L;TMTC1;SORBS2;SLC7A11;AFF3;KLF7;NFIB;DLC1;KCNQ5* |
| ZNF678 | 13/299 | 3.037194265059546E-5 | *ZFHX3;KLF12;KMT2A;TMTC2;SLC7A11;LPP;CDK6;NFIA;TBL1XR1;NFIB;DLC1;BBX;RAB3GAP2* |
| ZNF70 | 13/299 | 3.037194265059546E-5 | *ZFHX3;KLF12;THRB;SLC7A11;LPP;TTC28;CTIF;KLF7;CDK6;NFIA;NFIB;DLC1;BBX* |
| ZNF774 | 13/299 | 3.037194265059546E-5 | *ZFHX3;THRB;KMT2A;BCL11A;GDPD1;SORBS2;SLC7A11;EFNA5;NR3C2;MED13L;NFIA;DLC1;SRGAP3* |
| ZNF780B | 13/299 | 3.037194265059546E-5 | *KLF12;KMT2A;VPS13B;SLC7A11;IKZF2;LPP;MBTD1;CDK6;NFIA;NFIB;ZNF429;BBX;ZNF234* |
| ZNF836 | 13/299 | 3.037194265059546E-5 | *ZFHX3;THRB;DAPK1;MX1;SORBS2;AFF3;CLDN1;ZNF607;NFIB;ZNF429;DLC1;ZNF615;ZNF234* |
| ZNF98 | 13/299 | 3.037194265059546E-5 | *ACSS3;PDE1C;ANGPT1;EYA2;COL12A1;TMTC1;SLC7A11;NPAS3;NFIA;CHL1;NFIB;ZNF429;DLC1* |
| ZSCAN20 | 13/299 | 3.037194265059546E-5 | *RERE;ZFHX3;NEDD4L;TMTC2;VPS13B;SORBS2;LPP;ARID1B;TANC1;FMNL2;NFIA;NFIB;DLC1* |
| ZSCAN5A | 13/299 | 3.037194265059546E-5 | *ZFHX3;BCL11A;ZMYND8;NEDD4L;SHROOM3;SORBS2;SLC7A11;AFF3;CDK6;NFIA;NFIB;DLC1;CDK14* |
| ARX | 12/299 | 1.1541230319715339E-4 | *THRB;NFIA;BCL11A;CHL1;NFIB;ALDH1A2;DLC1;WNT5A;ETV1;EFNA5;PAK3;EPHA3* |
| ASCL1 | 12/299 | 1.1541230319715339E-4 | *ZFHX3;NFIA;BCL11A;CHL1;NFIB;ALDH1A2;DLC1;WNT5A;ETV1;LHFPL3;ESR1;NPAS3* |
| ATF2 | 12/299 | 1.1541230319715339E-4 | *NCOA2;CAMK2D;CDK6;KMT2A;NFIA;TBL1XR1;NFIB;ZMYND8;UBR5;BBX;LPP;ESR1* |
| BAZ2A | 12/299 | 1.1541230319715339E-4 | *RERE;NCOA2;ZFHX3;KMT2A;NFIA;TBL1XR1;NFIB;ZMYND8;UBR5;LPP;ARID1B;MED13L* |
| CREB5 | 12/299 | 1.1541230319715339E-4 | *KLF7;ZFHX3;CDK6;NFIA;NFIB;DLC1;COL12A1;NEDD4L;ETV1;SORBS2;SLC7A11;LPP* |
| DBX2 | 12/299 | 1.1541230319715339E-4 | *ZFHX3;MEGF10;NFIA;CHL1;EYA2;NFIB;DLC1;COL12A1;LHFPL3;SLC7A11;NR3C2;NPAS3* |
| DLX5 | 12/299 | 1.1541230319715339E-4 | *BCL11A;EYA2;ALDH1A2;DLC1;COL12A1;WNT5A;MPPED2;RARB;PPARG;SORBS2;LHFPL3;EPHA3* |
| DLX6 | 12/299 | 1.154123031971 | *ZFHX3;NFIA;BCL11A;EYA2;NFIB;ALDH1A2;DLC1;WNT5A;MPPED2;RARB;LHFPL3;EFNA5* |

| | | | |
|---|---|---|---|
| | | 5339E-4 | |
| DMRT2 | 12/299 | 1.154123031971 5339E-4 | *NFIA;BCL11A;CHL1;EYA2;NFIB;ALDH1A2;DLC1;COL12A1;PPARG;LHFPL3;ESR1;NR3C2* |
| ELF5 | 12/299 | 1.154123031971 5339E-4 | *THRB;NFIA;BCL11A;EYA2;NFIB;DLC1;MPPED2;ETV1;PPARG;SLC7A11;CLDN1;ESR1* |
| EN1 | 12/299 | 1.154123031971 5339E-4 | *ZFHX3;NFIA;BCL11A;EYA2;NFIB;ALDH1A2;DLC1;COL12A1;WNT5A;PPARG;SLC7A11;AFF3* |
| ERG | 12/299 | 1.154123031971 5339E-4 | *CDK6;THRB;ANGPT1;NFIA;BCL11A;NFIB;DLC1;COL12A1;PPARG;HMCN1;AFF3;ESR1* |
| ESR1 | 12/299 | 1.154123031971 5339E-4 | *ZFHX3;CDK6;THRB;ANGPT1;NFIA;NFIB;DLC1;NEDD4L;RARB;PPARG;SORBS2;LPP* |
| FMNL2 | 12/299 | 1.154123031971 5339E-4 | *KLF7;ZFHX3;CDK6;NFIA;NFIB;DLC1;NEDD4L;APBB2;LAMB1;SLC7A11;AFF3;LPP* |
| FOXO3 | 12/299 | 1.154123031971 5339E-4 | *RERE;ZFHX3;CDK6;NFIA;NFIB;ZMYND8;DLC1;NEDD4L;PPARG;LPP;ESR1;MED13L* |
| GATA4 | 12/299 | 1.154123031971 5339E-4 | *ZFHX3;CDK6;THRB;NFIA;BCL11A;NFIB;ALDH1A2;DLC1;WNT5A;RARB;PPARG;ESR1* |
| GPATCH8 | 12/299 | 1.154123031971 5339E-4 | *RERE;ZFHX3;KMT2A;NFIA;TBL1XR1;NFIB;ZMYND8;UBR5;NEDD4L;LPP;ARID1B;MED13L* |
| GRHL2 | 12/299 | 1.154123031971 5339E-4 | *ZFHX3;THRB;NFIA;BCL11A;ATP8B1;NFIB;DLC1;COL12A1;NEDD4L;SHROOM3;EFNA5;ESR1* |
| GTF2IRD2B | 12/299 | 1.154123031971 5339E-4 | *RERE;ZFHX3;CDK6;NFIA;NFIB;DLC1;SORBS2;LHFPL3;SRGAP3;SLC7A11;ACACB;NR3C2* |
| HIF3A | 12/299 | 1.154123031971 5339E-4 | *ANGPT1;NFIA;BCL11A;NFIB;DLC1;COL12A1;TMTC1;SORBS2;SLC7A11;AFF3;FBLN2;ACACB* |
| HOXA11 | 12/299 | 1.154123031971 5339E-4 | *ZFHX3;CDK6;BCL11A;NFIB;ALDH1A2;DLC1;COL12A1;WNT5A;ETV1;PPARG;SLC7A11;ESR1* |
| HOXA9 | 12/299 | 1.154123031971 5339E-4 | *ZFHX3;CDK6;ANGPT1;NFIA;BCL11A;NFIB;DLC1;COL12A1;WNT5A;RARB;PPARG;ESR1* |
| HOXC11 | 12/299 | 1.154123031971 5339E-4 | *ZFHX3;NFIA;BCL11A;NFIB;ALDH1A2;DLC1;COL12A1;WNT5A;ETV1;PPARG;AFF3;ESR1* |
| IKZF2 | 12/299 | 1.154123031971 5339E-4 | *KLF7;ZFHX3;KLF12;CDK6;NFIA;BCL11A;NFIB;DLC1;IL1RAP;SLC7A11;AFF3;LPP* |
| LHX4 | 12/299 | 1.154123031971 5339E-4 | *ZFHX3;NFIA;BCL11A;EYA2;NFIB;DLC1;NEDD4L;LHFPL3;HMCN1;EFNA5;ESR1;NPAS3* |
| MIER3 | 12/299 | 1.154123031971 5339E-4 | *MBTD1;KLF7;ZFHX3;KLF12;CDK6;NFIA;TBL1XR1;NFIB;DLC1;BBX;LPP;MED13L* |
| MLLT3 | 12/299 | 1.154123031971 5339E-4 | *ZFHX3;KLF12;CAMK2D;CDK6;NFIA;DAPK1;BCL11A;NFIB;DLC1;NEDD4L;IKZF2;EFNA5* |
| MSGN1 | 12/299 | 1.154123031971 5339E-4 | *ZFHX3;ANGPT1;NFIA;ST8SIA1;BCL11A;ALDH1A2;DLC1;WNT5A;SLC7A11;EFNA5;FBLN2;ESR1* |
| NFYA | 12/299 | 1.154123031971 5339E-4 | *KLF7;ZFHX3;CDK6;KMT2A;NFIA;TBL1XR1;NFIB;ZMYND8;DLC1;BBX;LPP;ESR1* |
| NPAS2 | 12/299 | 1.154123031971 5339E-4 | *NFIA;DAPK1;NFIB;DLC1;COL12A1;WNT5A;NEDD4L;PPARG;SORBS2;SLC7A11;EFNA5;CLDN1* |
| NR2E1 | 12/299 | 1.154123031971 5339E-4 | *NFIA;BCL11A;CHL1;NFIB;ALDH1A2;DLC1;WNT5A;RARB;LHFPL3;SLC7A11;ESR1;NPAS3* |
| NR2F2 | 12/299 | 1.154123031971 5339E-4 | *ZFHX3;CDK6;NFIA;BCL11A;NFIB;DLC1;COL12A1;WNT5A;ETV1;PPARG;LAMB1;EFNA5* |
| PAX5 | 12/299 | 1.154123031971 5339E-4 | *ZFHX3;KLF12;CDK6;NFIA;BCL11A;NFIB;DLC1;PPARG;CACNA1C;AFF3;ESR1;ARID1B* |
| PAX7 | 12/299 | 1.154123031971 5339E-4 | *ZFHX3;NFIA;BCL11A;EYA2;NFIB;DLC1;WNT5A;ETV1;LHFPL3;EFNA5;ESR1;NPAS3* |
| PBX1 | 12/299 | 1.154123031971 5339E-4 | *ZFHX3;CDK6;THRB;NFIA;BCL11A;NFIB;DLC1;NEDD4L;ETV1;SORBS2;EFNA5;LPP* |
| PBX3 | 12/299 | 1.154123031971 5339E-4 | *ZFHX3;CDK6;NFIA;BCL11A;NFIB;DLC1;MPPED2;NEDD4L;RARB;ETV1;EFNA5;LPP* |
| PITX2 | 12/299 | 1.154123031971 5339E-4 | *ZFHX3;NFIA;BCL11A;NFIB;ALDH1A2;DLC1;COL12A1;WNT5A;RARB;ETV1;PPARG;EFNA5* |

| | | | |
|---|---|---|---|
| PKHD1L1 | 12/299 | 1.154123031971 5339E-4 | *ADGRD1;COL24A1;ANGPT1;ALDH1A2;DLC1;COL12A1;SORBS2;HMCN1;FBLN2;ACACB;EPHA3;TTN* |
| POGZ | 12/299 | 1.154123031971 5339E-4 | *RERE;ZFHX3;KMT2A;NFIA;TBL1XR1;BCL11A;NFIB;ZMYND8;UBR5;LPP;ARID1B;MED13L* |
| POU6F1 | 12/299 | 1.154123031971 5339E-4 | *ZFHX3;KLF12;GABBR1;NFIA;BCL11A;NFIB;DLC1;NEDD4L;SRGAP3;AFF3;NR3C2;TTC28* |
| PROX1 | 12/299 | 1.154123031971 5339E-4 | *KLF7;ZFHX3;NFIA;BCL11A;NFIB;DLC1;WNT5A;ETV1;SORBS2;EFNA5;EPHA3;NR3C2* |
| RAPGEF4 | 12/299 | 1.154123031971 5339E-4 | *GNAO1;NFIA;BCL11A;NFIB;DLC1;PRICKLE2;NEDD4L;TMTC1;DLGAP1;SORBS2;SLC7A11;NR3C2* |
| RUNX2 | 12/299 | 1.154123031971 5339E-4 | *ZFHX3;CDK6;NFIA;BCL11A;NFIB;DLC1;COL12A1;WNT5A;PPARG;SLC7A11;LPP;ESR1* |
| SALL3 | 12/299 | 1.154123031971 5339E-4 | *ZFHX3;KLF12;CDK6;NFIA;BCL11A;CHL1;EYA2;NFIB;DLC1;LHFPL3;EFNA5;NPAS3* |
| SMAD2 | 12/299 | 1.154123031971 5339E-4 | *NCOA2;ZFHX3;CDK6;NFIA;TBL1XR1;NFIB;DLC1;NEDD4L;PPARG;LPP;ESR1;ARID1B* |
| SOX2 | 12/299 | 1.154123031971 5339E-4 | *CDK6;MEGF10;NFIA;BCL11A;NFIB;DLC1;WNT5A;ETV1;PPARG;SLC7A11;ESR1;EPHA3* |
| SSH2 | 12/299 | 1.154123031971 5339E-4 | *RERE;NCOA2;KLF7;ZFHX3;CDK6;NFIA;NFIB;DLC1;NEDD4L;LPP;ARID1B;MED13L* |
| TBX4 | 12/299 | 1.154123031971 5339E-4 | *ZFHX3;NFIA;BCL11A;NFIB;ALDH1A2;DLC1;WNT5A;EFNA5;AFF3;FBLN2;ESR1;NPAS3* |
| TFAP2C | 12/299 | 1.154123031971 5339E-4 | *CDK6;NFIA;BCL11A;NFIB;DLC1;WNT5A;ETV1;PPARG;SLC7A11;EFNA5;CLDN1;ESR1* |
| TGIF2LX | 12/299 | 1.154123031971 5339E-4 | *ZFHX3;KLF12;THRB;ADGRB3;BCL11A;NFIB;RARB;ETV1;PPARG;ESR1;EPHA3;NR3C2* |
| TOX3 | 12/299 | 1.154123031971 5339E-4 | *ZFHX3;BCL11A;NFIB;DLC1;WNT5A;MPPED2;NEDD4L;ETV1;SORBS2;LHFPL3;EFNA5;AFF3* |
| TP63 | 12/299 | 1.154123031971 5339E-4 | *ZFHX3;CDK6;THRB;NFIA;BCL11A;NFIB;DLC1;ETV1;PPARG;SLC7A11;CLDN1;ESR1* |
| TRPS1 | 12/299 | 1.154123031971 5339E-4 | *ZFHX3;CDK6;ANGPT1;NFIA;NFIB;DLC1;WNT5A;ETV1;SLC7A11;EFNA5;LPP;CDK14* |
| YEATS2 | 12/299 | 1.154123031971 5339E-4 | *RERE;NCOA2;ZFHX3;CDK6;KMT2A;TBL1XR1;ZMYND8;UBR5;BBX;LPP;ARID1B;MED13L* |
| ZBTB39 | 12/299 | 1.154123031971 5339E-4 | *RERE;KLF7;ZFHX3;CDK6;KMT2A;NFIA;DAPK1;NFIB;DLC1;LPP;ARID1B;MED13L* |
| ZCCHC11 | 12/299 | 1.154123031971 5339E-4 | *RERE;ZFHX3;KMT2A;NFIA;NFIB;ZMYND8;DLC1;UBR5;BBX;LPP;ARID1B;MED13L* |
| ZFHX2 | 12/299 | 1.154123031971 5339E-4 | *RERE;ZFHX3;NFIA;DAPK1;BCL11A;NFIB;ZMYND8;DLC1;NEDD4L;SORBS2;CACNA1C;SRGAP3* |
| ZFP2 | 12/299 | 1.154123031971 5339E-4 | *KLF12;THRB;NFIA;BCL11A;NFIB;DLC1;MPPED2;PELI2;RARB;IKZF2;NR3C2;RAPGEF4* |
| ZFP28 | 12/299 | 1.154123031971 5339E-4 | *KLF7;ZFHX3;KLF12;CAMK2D;CDK6;NFIA;NFIB;DLC1;NEDD4L;KCNQ5;APBB2;SLC7A11* |
| ZFP3 | 12/299 | 1.154123031971 5339E-4 | *ZFHX3;THRB;NFIA;BCL11A;NFIB;DLC1;MPPED2;MX1;PPARG;LHFPL3;LPP;NR3C2* |
| ZFP37 | 12/299 | 1.154123031971 5339E-4 | *ZFHX3;KLF12;PDE1C;ANGPT1;NFIA;BCL11A;CHL1;NFIB;DLC1;ETV1;SORBS2;SLC7A11* |
| ZFX | 12/299 | 1.154123031971 5339E-4 | *NCOA2;ZFHX3;CDK6;KMT2A;NFIA;TBL1XR1;NFIB;ZMYND8;BBX;LPP;ARID1B;MED13L* |
| ZKSCAN1 | 12/299 | 1.154123031971 5339E-4 | *RERE;NCOA2;ZFHX3;CDK6;KMT2A;NFIA;NFIB;ZMYND8;BBX;VPS13B;LPP;MED13L* |
| ZNF10 | 12/299 | 1.154123031971 5339E-4 | *KLF12;CDK6;NFIA;BCL11A;NFIB;DLC1;PELI2;ZNF615;NEDD4L;SLC7A11;IKZF2;CFAP44* |
| ZNF135 | 12/299 | 1.154123031971 5339E-4 | *MAPK10;THRB;NFIA;BCL11A;CHL1;NFIB;DLC1;MX1;SORBS2;LDLRAD4;AFF3;NR3C2* |
| ZNF141 | 12/299 | 1.154123031971 5339E-4 | *MBTD1;KLF12;CDK6;BCL11A;ZNF429;DLC1;ZNF615;LHFPL3;IKZF2;AFF3;LPP;NR3C2* |
| ZNF208 | 12/299 | 1.154123031971 | *PDE1C;NFIA;ST8SIA1;CHL1;ZNF429;DLC1;AKAP6;LHFPL3;HMCN1;CACNA1C;FBLN2;TTN* |

| | | | |
|---|---|---|---|
| | | 5339E-4 | |
| ZNF221 | 12/299 | 1.154123031971 5339E-4 | *KLF12;ZNF607;ZNF429;DLC1;COL12A1;ZNF615;VPS13B;ETV1;HMCN1;SLC7A11;ZNF234;TTN* |
| ZNF234 | 12/299 | 1.154123031971 5339E-4 | *MBTD1;CDK6;ZNF607;NFIB;ZMYND8;ZNF429;MX1;ZNF615;GDPD1;VPS13B;SLC7A11;LPP* |
| ZNF287 | 12/299 | 1.154123031971 5339E-4 | *ZFHX3;KLF12;CDK6;NFIA;BCL11A;DLC1;ETV1;LDLRAD4;SLC7A11;IKZF2;LPP;EPHA3* |
| ZNF320 | 12/299 | 1.154123031971 5339E-4 | *MBTD1;KLF12;ZNF607;NFIA;NFIB;ZNF429;DLC1;ZNF615;GDPD1;NEDD4L;ZNF665;ZNF234* |
| ZNF347 | 12/299 | 1.154123031971 5339E-4 | *CDK6;ZNF607;BCL11A;NFIB;ZNF429;DLC1;ZNF615;BBX;IKZF2;ZNF665;LPP;ZNF234* |
| ZNF396 | 12/299 | 1.154123031971 5339E-4 | *ZFHX3;NFIA;NFIB;DLC1;PELI2;TMTC2;SORBS2;LDLRAD4;SRGAP3;IKZF2;ESR1;NPAS3* |
| ZNF398 | 12/299 | 1.154123031971 5339E-4 | *ZFHX3;KLF12;CDK6;KMT2A;NFIA;BCL11A;NFIB;ZMYND8;DLC1;NEDD4L;LHFPL3;ARID1B* |
| ZNF44 | 12/299 | 1.154123031971 5339E-4 | *MBTD1;ZFHX3;KLF12;KMT2A;DAPK1;BCL11A;DLC1;ZNF615;NEDD4L;SORBS2;SLC7A11;NR3C2* |
| ZNF454 | 12/299 | 1.154123031971 5339E-4 | *PDE1C;CHL1;NFIB;DLC1;TMTC1;ETV1;LHFPL3;CACNA1C;ZNF665;ZNF521;NPAS3;HCN1* |
| ZNF470 | 12/299 | 1.154123031971 5339E-4 | *KLF12;ZNF607;BCL11A;ZNF429;DLC1;MX1;ZNF615;NEDD4L;LHFPL3;SLC7A11;ZNF234;NR3C2* |
| ZNF490 | 12/299 | 1.154123031971 5339E-4 | *RERE;ZFHX3;NFIA;TBL1XR1;BCL11A;NFIB;GDPD1;NEDD4L;SRGAP3;ARID1B;NR3C2;MED13L* |
| ZNF493 | 12/299 | 1.154123031971 5339E-4 | *MBTD1;KLF12;CDK6;NFIA;BCL11A;NFIB;ZNF429;BBX;SORBS2;LPP;NR3C2;TTN* |
| ZNF502 | 12/299 | 1.154123031971 5339E-4 | *THRB;ZNF607;NFIA;BCL11A;CHL1;NFIB;DLC1;PELI2;LHFPL3;SLC7A11;AFF3;NR3C2* |
| ZNF518A | 12/299 | 1.154123031971 5339E-4 | *MBTD1;CDK6;KMT2A;NFIA;BCL11A;NFIB;ZMYND8;UBR5;DMXL2;BBX;VPS13B;LPP* |
| ZNF521 | 12/299 | 1.154123031971 5339E-4 | *ANGPT1;NFIA;BCL11A;NFIB;DLC1;COL12A1;WNT5A;ETV1;HMCN1;AFF3;FBLN2;EPHA3* |
| ZNF527 | 12/299 | 1.154123031971 5339E-4 | *KLF7;CDK6;THRB;ZNF607;NFIA;NFIB;ZNF429;ZNF615;GDPD1;NEDD4L;LAMB1;ZNF234* |
| ZNF565 | 12/299 | 1.154123031971 5339E-4 | *KLF7;ZFHX3;KLF12;KMT2A;NFIA;NFIB;ZMYND8;DLC1;PELI2;TMTC2;LAMB1;SLC7A11* |
| ZNF566 | 12/299 | 1.154123031971 5339E-4 | *KLF12;CDK6;ZNF607;NFIA;BCL11A;ZNF429;DLC1;ZNF615;SLC7A11;IKZF2;LPP;ZNF234* |
| ZNF572 | 12/299 | 1.154123031971 5339E-4 | *MBTD1;ZNF607;ANGPT1;NFIA;DAPK1;BCL11A;DLC1;WNT5A;SORBS2;SLC7A11;CLDN1;NR3C2* |
| ZNF583 | 12/299 | 1.154123031971 5339E-4 | *KLF7;ZFHX3;KLF12;CDK6;ZNF607;NFIA;ZNF429;DLC1;ZNF615;SLC7A11;IKZF2;CDK14* |
| ZNF585A | 12/299 | 1.154123031971 5339E-4 | *ZFHX3;KLF12;CDK6;ZNF607;ANGPT1;NFIB;ZNF429;DLC1;ZNF615;BBX;SLC7A11;LPP* |
| ZNF596 | 12/299 | 1.154123031971 5339E-4 | *ZFHX3;NFIA;BCL11A;NFIB;DLC1;ZNF615;PRICKLE2;PTK2B;SORBS2;SRGAP3;SLC7A11;NR3C2* |
| ZNF619 | 12/299 | 1.154123031971 5339E-4 | *NCOA2;ZFHX3;KMT2A;NFIA;DLC1;ZNF615;BBX;CEP192;HMCN1;SLC7A11;IKZF2;LPP* |
| ZNF624 | 12/299 | 1.154123031971 5339E-4 | *KLF7;ZFHX3;KLF12;ZNF197;CDK6;NFIA;DLC1;ZNF615;BBX;AKAP6;SLC7A11;IKZF2* |
| ZNF626 | 12/299 | 1.154123031971 5339E-4 | *ZNF607;NFIA;BCL11A;NFIB;ZNF429;DLC1;ZNF615;NEDD4L;AFF3;LPP;ACACB;NR3C2* |
| ZNF711 | 12/299 | 1.154123031971 5339E-4 | *CDK6;NFIA;BCL11A;CHL1;NFIB;DLC1;COL12A1;WNT5A;ETV1;SLC7A11;AFF3;EPHA3* |
| ZNF74 | 12/299 | 1.154123031971 5339E-4 | *KLF7;ZFHX3;KLF12;CDK6;BCL11A;NFIB;ZMYND8;DLC1;SORBS2;LPP;ESR1;NPAS3* |
| ZNF75A | 12/299 | 1.154123031971 5339E-4 | *KLF7;ZFHX3;KLF12;CDK6;BCL11A;NFIB;ZMYND8;DLC1;ZNF615;NEDD4L;SHROOM3;SLC7A11* |
| ZNF772 | 12/299 | 1.154123031971 5339E-4 | *ZFHX3;KLF12;CDK6;THRB;ZNF607;NFIB;ZNF429;ZNF615;NEDD4L;IKZF2;AFF3;LPP* |

| | | | |
|---|---|---|---|
| ZNF782 | 12/299 | 1.1541230319715339E-4 | *MBTD1;NCOA2;ZFHX3;THRB;NFIA;DLC1;NEDD4L;TMTC2;VPS13B;SLC7A11;ACACB;CFAP44* |
| ZNF841 | 12/299 | 1.1541230319715339E-4 | *KLF7;ZFHX3;CDK6;KMT2A;NFIB;ZNF429;DLC1;ZNF615;NEDD4L;SLC7A11;CLDN1;LPP* |
| ZXDC | 12/299 | 1.1541230319715339E-4 | *RERE;KLF7;ZFHX3;KMT2A;NFIA;TBL1XR1;NFIB;DLC1;NEDD4L;LPP;ARID1B;MED13L* |
| ADNP | 11/299 | 4.3472804228551277E-4 | *RERE;ZFHX3;KMT2A;NFIA;TBL1XR1;BCL11A;NFIB;ZMYND8;UBR5;ARID1B;MED13L* |
| AFF4 | 11/299 | 4.3472804228551277E-4 | *RERE;NCOA2;ZFHX3;KMT2A;TBL1XR1;NFIB;UBR5;BBX;NEDD4L;LPP;MED13L* |
| AHDC1 | 11/299 | 4.3472804228551277E-4 | *RERE;ZFHX3;NFIA;TBL1XR1;BCL11A;NFIB;ZMYND8;DLC1;SRGAP3;ARID1B;MED13L* |
| ASCL4 | 11/299 | 4.3472804228551277E-4 | *ZFHX3;NFIA;EYA2;ALDH1A2;DLC1;RARB;LHFPL3;LDLRAD4;AFF3;ESR1;NPAS3* |
| CREB1 | 11/299 | 4.3472804228551277E-4 | *NCOA2;KLF7;CDK6;KMT2A;NFIA;TBL1XR1;NFIB;BBX;PPARG;LPP;ESR1* |
| CREBZF | 11/299 | 4.3472804228551277E-4 | *MBTD1;CDK6;KMT2A;NFIA;TBL1XR1;NFIB;ZMYND8;UBR5;BBX;SLC7A11;LPP* |
| DLX2 | 11/299 | 4.3472804228551277E-4 | *ZFHX3;NFIA;BCL11A;NFIB;DLC1;COL12A1;WNT5A;ETV1;PPARG;SLC7A11;EFNA5* |
| DMRTB1 | 11/299 | 4.3472804228551277E-4 | *ZFHX3;AFAP1L2;NFIA;EYA2;ALDH1A2;DLC1;WNT5A;LHFPL3;EFNA5;FBLN2;KBTBD11* |
| EMX2 | 11/299 | 4.3472804228551277E-4 | *ZFHX3;NFIA;BCL11A;NFIB;ALDH1A2;DLC1;COL12A1;WNT5A;ETV1;PPARG;FBLN2* |
| ESRRB | 11/299 | 4.3472804228551277E-4 | *ZFHX3;THRB;NFIA;BCL11A;NFIB;DLC1;ETV1;PPARG;LHFPL3;CACNA1C;ESR1* |
| EZH1 | 11/299 | 4.3472804228551277E-4 | *RERE;KLF7;ZFHX3;KMT2A;NFIA;NFIB;ZMYND8;DLC1;NEDD4L;LPP;MED13L* |
| FAM170A | 11/299 | 4.3472804228551277E-4 | *MEGF10;ANGPT1;ADGRB3;BCL11A;DLC1;LHFPL3;SLC7A11;EFNA5;ESR1;EPHA3;NR3C2* |
| FOXD3 | 11/299 | 4.3472804228551277E-4 | *GNAO1;ZFHX3;MEGF10;NFIA;BCL11A;CHL1;NFIB;ALDH1A2;DLC1;ETV1;LHFPL3* |
| FOXD4L6 | 11/299 | 4.3472804228551277E-4 | *ZFHX3;ADGRB3;BCL11A;ALDH1A2;DLC1;COL12A1;AGAP4;TMTC1;LHFPL3;EFNA5;ESR1* |
| FOXL1 | 11/299 | 4.3472804228551277E-4 | *ZFHX3;KLF12;NFIA;BCL11A;DLC1;COL12A1;WNT5A;PPARG;SLC7A11;EFNA5;ESR1* |
| FOXO1 | 11/299 | 4.3472804228551277E-4 | *ZFHX3;CDK6;NFIA;DAPK1;BCL11A;NFIB;DLC1;NEDD4L;PPARG;LPP;ESR1* |
| GLI1 | 11/299 | 4.3472804228551277E-4 | *CDK6;NFIA;BCL11A;DLC1;COL12A1;WNT5A;ETV1;PPARG;HMCN1;CLDN1;FBLN2* |
| GRM6 | 11/299 | 4.3472804228551277E-4 | *ZFHX3;GABBR1;BCL11A;EYA2;DLC1;ETV1;LHFPL3;CACNA1C;ESR1;TTN;HCN1* |
| HEY2 | 11/299 | 4.3472804228551277E-4 | *NFIA;BCL11A;NFIB;DLC1;WNT5A;MPPED2;ETV1;SORBS2;HMCN1;SLC7A11;EPHA3* |
| HIVEP2 | 11/299 | 4.3472804228551277E-4 | *RERE;KLF7;ZFHX3;CAMK2D;CDK6;NFIA;NFIB;DLC1;NEDD4L;SLC7A11;LPP* |
| HOXA1 | 11/299 | 4.3472804228551277E-4 | *CDK6;NFIA;BCL11A;NFIB;ALDH1A2;DLC1;WNT5A;RARB;PPARG;LAMB1;SLC7A11* |
| HOXA2 | 11/299 | 4.3472804228551277E-4 | *ZFHX3;NFIA;BCL11A;NFIB;ALDH1A2;DLC1;COL12A1;WNT5A;RARB;PPARG;LAMB1* |
| HOXB3 | 11/299 | 4.3472804228551277E-4 | *ZFHX3;CDK6;NFIA;BCL11A;NFIB;DLC1;COL12A1;WNT5A;PPARG;AFF3;ESR1* |
| HOXC4 | 11/299 | 4.3472804228551277E-4 | *ZFHX3;NFIA;BCL11A;NFIB;ALDH1A2;DLC1;COL12A1;WNT5A;PPARG;AFF3;ESR1* |
| HOXD13 | 11/299 | 4.3472804228551277E-4 | *CDK6;BCL11A;ALDH1A2;DLC1;COL12A1;WNT5A;ETV1;PPARG;LHFPL3;EFNA5;ESR1* |
| IRX1 | 11/299 | 4.3472804228551277E-4 | *ZFHX3;BCL11A;EYA2;NFIB;DLC1;COL12A1;WNT5A;LHFPL3;EFNA5;FBLN2;EPHA3* |
| ISL1 | 11/299 | 4.347280422855 | *ZFHX3;NFIA;BCL11A;NFIB;ALDH1A2;DLC1;WNT5A;RARB;ETV1;EFNA5;EPHA3* |

| | | | |
|---|---|---|---|
| | | 1277E-4 | |
| KLF8 | 11/299 | 4.347280422855 1277E-4 | *NFIA;ST8SIA1;BCL11A;DLC1;TMTC1;PPARG;SLC7A11;IKZF2;CLDN1;FBLN2;NR3C2* |
| LGR4 | 11/299 | 4.347280422855 1277E-4 | *CDK6;NFIA;ATP8B1;NFIB;DLC1;WNT5A;SORBS2;LAMB1;SLC7A11;EFNA5;LPP* |
| LHX8 | 11/299 | 4.347280422855 1277E-4 | *ZFHX3;NFIA;NFIB;ALDH1A2;DLC1;COL12A1;WNT5A;RARB;LHFPL3;CACNA1C;SLC7A11* |
| LMX1A | 11/299 | 4.347280422855 1277E-4 | *ZFHX3;NFIA;EYA2;NFIB;ALDH1A2;DLC1;WNT5A;LHFPL3;EFNA5;AFF3;ESR1* |
| MBNL2 | 11/299 | 4.347280422855 1277E-4 | *ZFHX3;CAMK2D;CDK6;NFIA;NFIB;DLC1;BBX;NEDD4L;SORBS2;SLC7A11;LPP* |
| MEOX2 | 11/299 | 4.347280422855 1277E-4 | *ANGPT1;NFIB;ALDH1A2;DLC1;COL12A1;ETV1;PPARG;SORBS2;HMCN1;FBLN2;EPHA3* |
| MKX | 11/299 | 4.347280422855 1277E-4 | *NFIA;NFIB;DLC1;COL12A1;WNT5A;RARB;TMTC1;HMCN1;SLC7A11;EFNA5;EPHA3* |
| MSX2 | 11/299 | 4.347280422855 1277E-4 | *ZFHX3;THRB;NFIA;DLC1;COL12A1;WNT5A;PPARG;SLC7A11;EFNA5;FBLN2;ESR1* |
| NCOR1 | 11/299 | 4.347280422855 1277E-4 | *RERE;NCOA2;ZFHX3;KMT2A;TBL1XR1;NFIB;ZMYND8;UBR5;LPP;ARID1B;MED13L* |
| NCOR2 | 11/299 | 4.347280422855 1277E-4 | *RERE;NCOA2;ZFHX3;CDK6;NFIA;NFIB;ZMYND8;DLC1;NEDD4L;LPP;ARID1B* |
| NFATC3 | 11/299 | 4.347280422855 1277E-4 | *RERE;ZFHX3;CDK6;KMT2A;NFIA;ZMYND8;DLC1;PPARG;APBB2;LPP;MED13L* |
| NHLH2 | 11/299 | 4.347280422855 1277E-4 | *ZFHX3;NFIA;BCL11A;NFIB;DLC1;LHFPL3;SRGAP3;EFNA5;AFF3;EPHA3;NPAS3* |
| NR2F1 | 11/299 | 4.347280422855 1277E-4 | *ANGPT1;NFIA;BCL11A;NFIB;DLC1;COL12A1;WNT5A;RARB;ETV1;EFNA5;EPHA3* |
| OTX1 | 11/299 | 4.347280422855 1277E-4 | *ZFHX3;CDK6;NFIA;BCL11A;NFIB;DLC1;WNT5A;MPPED2;SLC7A11;EFNA5;ESR1* |
| PDS5B | 11/299 | 4.347280422855 1277E-4 | *RERE;NCOA2;KMT2A;NFIA;TBL1XR1;NFIB;ZMYND8;UBR5;BBX;LPP;ARID1B* |
| PHOX2B | 11/299 | 4.347280422855 1277E-4 | *ZFHX3;NFIA;BCL11A;CHL1;NFIB;ALDH1A2;DLC1;WNT5A;LHFPL3;EPHA3;NPAS3* |
| PLXNA2 | 11/299 | 4.347280422855 1277E-4 | *KLF7;ZFHX3;CDK6;NFIA;NFIB;DLC1;NEDD4L;SORBS2;HMCN1;CACNA1C;EFNA5* |
| POU3F1 | 11/299 | 4.347280422855 1277E-4 | *NFIA;BCL11A;NFIB;ALDH1A2;DLC1;RARB;ETV1;LHFPL3;EFNA5;AFF3;KBTBD11* |
| PPARA | 11/299 | 4.347280422855 1277E-4 | *ZFHX3;CDK6;THRB;NFIA;NFIB;DLC1;NEDD4L;PPARG;LPP;ACACB;ESR1* |
| RNF113B | 11/299 | 4.347280422855 1277E-4 | *RERE;KLF12;NFIA;DLC1;COL12A1;PRICKLE2;NEDD4L;SORBS2;LHFPL3;EFNA5;ESR1* |
| RXRA | 11/299 | 4.347280422855 1277E-4 | *ZFHX3;CDK6;NFIA;DAPK1;NFIB;ZMYND8;DLC1;NEDD4L;PPARG;LPP;ESR1* |
| SALL1 | 11/299 | 4.347280422855 1277E-4 | *ZFHX3;CDK6;NFIA;BCL11A;NFIB;DLC1;WNT5A;SHROOM3;ETV1;EFNA5;NPAS3* |
| SCRT2 | 11/299 | 4.347280422855 1277E-4 | *ZFHX3;NFIA;BCL11A;NFIB;DLC1;MPPED2;DLGAP1;LHFPL3;CACNA1C;AFF3;NPAS3* |
| SIX2 | 11/299 | 4.347280422855 1277E-4 | *ZFHX3;NFIA;BCL11A;EYA2;NFIB;ALDH1A2;DLC1;COL12A1;WNT5A;ETV1;EFNA5* |
| SIX3 | 11/299 | 4.347280422855 1277E-4 | *ZFHX3;NFIA;BCL11A;CHL1;NFIB;DLC1;WNT5A;RARB;LHFPL3;EFNA5;NPAS3* |
| SMAD1 | 11/299 | 4.347280422855 1277E-4 | *ZFHX3;CDK6;NFIA;DAPK1;BCL11A;NFIB;DLC1;PPARG;SLC7A11;LPP;ESR1* |
| SMAD3 | 11/299 | 4.347280422855 1277E-4 | *ZFHX3;CDK6;NFIA;DAPK1;NFIB;DLC1;NEDD4L;PTK2B;PPARG;LPP;ESR1* |
| SMARCA1 | 11/299 | 4.347280422855 1277E-4 | *NFIA;DAPK1;BCL11A;NFIB;DLC1;COL12A1;WNT5A;ETV1;SORBS2;LAMB1;SLC7A11* |
| SOX11 | 11/299 | 4.347280422855 1277E-4 | *ZFHX3;NFIA;BCL11A;NFIB;DLC1;COL12A1;WNT5A;ETV1;SLC7A11;EFNA5;EPHA3* |

| | | | |
|---|---|---|---|
| SP1 | 11/299 | 4.347280422855 1277E-4 | *NCOA2;ZFHX3;CDK6;KMT2A;NFIA;TBL1XR1;NFIB;DLC1;PPARG;ESR1;MED13L* |
| SP8 | 11/299 | 4.347280422855 1277E-4 | *ZFHX3;CDK6;NFIA;BCL11A;NFIB;DLC1;COL12A1;WNT5A;ETV1;LHFPL3;EFNA5* |
| SSH1 | 11/299 | 4.347280422855 1277E-4 | *RERE;KLF7;ZFHX3;CDK6;ZMYND8;DLC1;NEDD4L;SLC7A11;ASAP2;LPP;MED13L* |
| TBX18 | 11/299 | 4.347280422855 1277E-4 | *ANGPT1;NFIA;NFIB;ALDH1A2;DLC1;COL12A1;WNT5A;HMCN1;EFNA5;FBLN2;EPHA3* |
| TCF3 | 11/299 | 4.347280422855 1277E-4 | *RERE;ZFHX3;CDK6;KMT2A;NFIA;TBL1XR1;BCL11A;NFIB;ZMYND8;ARID1B;MED13L* |
| TSHZ2 | 11/299 | 4.347280422855 1277E-4 | *ZFHX3;NFIA;NFIB;DLC1;COL12A1;SORBS2;HMCN1;EFNA5;AFF3;LPP;EPHA3* |
| ZBTB34 | 11/299 | 4.347280422855 1277E-4 | *RERE;ZFHX3;CDK6;KMT2A;NFIA;TBL1XR1;NFIB;DLC1;NEDD4L;LPP;MED13L* |
| ZC3H7B | 11/299 | 4.347280422855 1277E-4 | *RERE;ZFHX3;CDK6;NFIA;NFIB;ZMYND8;DLC1;NEDD4L;LPP;ARID1B;MED13L* |
| ZFP1 | 11/299 | 4.347280422855 1277E-4 | *ZFHX3;KLF12;CDK6;NFIA;TBL1XR1;ZMYND8;DLC1;BBX;NEDD4L;IKZF2;LPP* |
| ZFP82 | 11/299 | 4.347280422855 1277E-4 | *KLF12;CDK6;ZNF607;BCL11A;NFIB;DLC1;NEDD4L;LHFPL3;SLC7A11;AFF3;KBTBD11* |
| ZFP92 | 11/299 | 4.347280422855 1277E-4 | *THRB;CHL1;DLC1;PRKG2;TMTC1;ETV1;PPARG;LHFPL3;CACNA1C;SLC7A11;PAK3* |
| ZKSCAN5 | 11/299 | 4.347280422855 1277E-4 | *RERE;KLF7;ZFHX3;CDK6;NFIA;TBL1XR1;NFIB;ZMYND8;DLC1;NEDD4L;ETV1* |
| ZNF169 | 11/299 | 4.347280422855 1277E-4 | *KLF12;CDK6;BCL11A;NFIB;ZMYND8;DLC1;COL12A1;SORBS2;SLC7A11;LPP;ZNF234* |
| ZNF19 | 11/299 | 4.347280422855 1277E-4 | *ZFHX3;CAMK2D;NFIA;DLC1;MX1;NEDD4L;SORBS2;SRGAP3;SLC7A11;AFF3;ACACB* |
| ZNF2 | 11/299 | 4.347280422855 1277E-4 | *RERE;ZFHX3;CDK6;NFIA;BCL11A;DLC1;GDPD1;NEDD4L;ETV1;APBB2;AFF3* |
| ZNF224 | 11/299 | 4.347280422855 1277E-4 | *KLF12;CDK6;KMT2A;NFIB;DMXL2;ZNF615;BBX;VPS13B;LPP;ZNF234;MED13L* |
| ZNF280B | 11/299 | 4.347280422855 1277E-4 | *ZFHX3;CDK6;DAPK1;BCL11A;NFIB;DLC1;NEDD4L;ETV1;PPARG;LHFPL3;SLC7A11* |
| ZNF300 | 11/299 | 4.347280422855 1277E-4 | *GABBR1;CDK6;NFIA;BCL11A;NFIB;DLC1;WNT5A;TMTC1;LAMB1;SLC7A11;AFF3* |
| ZNF33A | 11/299 | 4.347280422855 1277E-4 | *MBTD1;ZFHX3;KLF12;CDK6;KMT2A;NFIA;TBL1XR1;BCL11A;NFIB;BBX;MED13L* |
| ZNF33B | 11/299 | 4.347280422855 1277E-4 | *ZFHX3;KLF12;CDK6;NFIA;DAPK1;TBL1XR1;BCL11A;NFIB;NEDD4L;SLC7A11;CLDN1* |
| ZNF341 | 11/299 | 4.347280422855 1277E-4 | *RERE;ZFHX3;CDK6;NFIA;TBL1XR1;ZMYND8;DLC1;NEDD4L;ETV1;PPARG;SORBS2* |
| ZNF382 | 11/299 | 4.347280422855 1277E-4 | *MAPK10;KLF12;CDK6;ZNF607;ZNF429;DLC1;ZNF615;NEDD4L;KCNQ5;SLC7A11;AFF3* |
| ZNF441 | 11/299 | 4.347280422855 1277E-4 | *ZFHX3;KLF12;CDK6;NFIA;ZNF429;DLC1;ZNF615;IL1RAP;SLC7A11;AFF3;CFAP44* |
| ZNF449 | 11/299 | 4.347280422855 1277E-4 | *ZFHX3;KLF12;CDK6;TBL1XR1;MAOA;NFIB;DLC1;BBX;RARB;SLC7A11;LPP* |
| ZNF479 | 11/299 | 4.347280422855 1277E-4 | *ADGRD1;ZFHX3;BCL11A;DLC1;ZNF536;SORBS2;LHFPL3;EPHA3;NR3C2;TTN;HCN1* |
| ZNF506 | 11/299 | 4.347280422855 1277E-4 | *KLF12;CDK6;NFIA;BCL11A;NFIB;ZNF429;MX1;ZNF615;IKZF2;AFF3;NR3C2* |
| ZNF529 | 11/299 | 4.347280422855 1277E-4 | *KLF12;CDK6;ZNF607;BCL11A;NFIB;ZNF429;DLC1;ZNF615;NEDD4L;LPP;ZNF234* |
| ZNF570 | 11/299 | 4.347280422855 1277E-4 | *KLF7;KLF12;ZNF607;NFIA;ZNF429;DLC1;ZNF615;TMTC2;SLC7A11;ZNF234;TTC28* |
| ZNF585B | 11/299 | 4.347280422855 1277E-4 | *ZFHX3;CDK6;ZNF607;NFIA;NFIB;ZNF429;DLC1;ZNF615;SLC7A11;CDK14;ZNF234* |
| ZNF599 | 11/299 | 4.347280422855 | *ZFHX3;KLF12;ZNF607;NFIA;NFIB;DLC1;GDPD1;NEDD4L;SRGAP3;AFF3;TTC28* |

| | | | |
|---|---|---|---|
| | | 1277E-4 | |
| ZNF606 | 11/299 | 4.347280422855 1277E-4 | *ZFHX3;KLF12;ZNF607;BCL11A;NFIB;ZNF429;DLC1;ZNF615;NEDD4L;ZNF665;ZNF234* |
| ZNF625 | 11/299 | 4.347280422855 1277E-4 | *CECR2;ZFHX3;ZNF607;KMT2A;NFIA;NFIB;ZNF429;ZNF615;TMTC1;ZNF665;ZNF234* |
| ZNF648 | 11/299 | 4.347280422855 1277E-4 | *ZFHX3;NFIA;BCL11A;NFIB;DLC1;SORBS2;LHFPL3;HMCN1;EFNA5;ESR1;EPHA3* |
| ZNF7 | 11/299 | 4.347280422855 1277E-4 | *RERE;ZFHX3;CDK6;DAPK1;NFIB;ZMYND8;DLC1;UBR5;BBX;NEDD4L;ARID1B* |
| ZNF701 | 11/299 | 4.347280422855 1277E-4 | *KLF7;KLF12;CDK6;ZNF429;DLC1;ZNF615;BBX;SLC7A11;ZNF665;LPP;ZNF234* |
| ZNF763 | 11/299 | 4.347280422855 1277E-4 | *ZFHX3;KLF12;ZNF607;NFIA;BCL11A;ZNF429;DLC1;ZNF615;SLC7A11;AFF3;TTC28* |
| ZNF780A | 11/299 | 4.347280422855 1277E-4 | *NCOA2;KLF12;CDK6;TBL1XR1;NFIB;ZNF429;DMXL2;BBX;SLC7A11;LPP;ZNF234* |
| ZSCAN12 | 11/299 | 4.347280422855 1277E-4 | *RERE;KLF7;ZFHX3;KLF12;NFIA;BCL11A;NFIB;DLC1;NEDD4L;SLC7A11;IKZF2* |
| ZZZ3 | 11/299 | 4.347280422855 1277E-4 | *RERE;NCOA2;ZFHX3;CAMK2D;CDK6;TBL1XR1;NFIB;UBR5;BBX;LPP;MED13L* |
| ARGFX | 10/299 | 0.001574203548 8365202 | *MAPK10;KLF7;ZFHX3;THRB;NFIB;APBB2;AFF3;LPP;ESR1;NPAS3* |
| BNC1 | 10/299 | 0.001574203548 8365202 | *NFIA;NFIB;ALDH1A2;DLC1;COL12A1;WNT5A;PPARG;SLC7A11;CLDN1;FBLN2* |
| CTCFL | 10/299 | 0.001574203548 8365202 | *ZFHX3;NFIA;BCL11A;DLC1;NEDD4L;PPARG;LAMB1;HMCN1;ESR1;NR3C2* |
| DUXA | 10/299 | 0.001574203548 8365202 | *PDE11A;NFIA;ST8SIA1;BCL11A;NFIB;DLC1;ETV1;LHFPL3;ZNF665;EPHA3* |
| DVL3 | 10/299 | 0.001574203548 8365202 | *RERE;ZFHX3;CDK6;KMT2A;NFIA;TBL1XR1;DLC1;LPP;ARID1B;MED13L* |
| EN2 | 10/299 | 0.001574203548 8365202 | *ZFHX3;NFIA;BCL11A;NFIB;ALDH1A2;DLC1;WNT5A;ETV1;LHFPL3;NPAS3* |
| ESR2 | 10/299 | 0.001574203548 8365202 | *CDK6;THRB;NFIA;BCL11A;DLC1;RARB;PPARG;CACNA1C;ESR1;NR3C2* |
| FOXB1 | 10/299 | 0.001574203548 8365202 | *ZFHX3;NFIA;BCL11A;EYA2;ALDH1A2;DLC1;WNT5A;LHFPL3;EFNA5;NPAS3* |
| FOXC1 | 10/299 | 0.001574203548 8365202 | *ZFHX3;CDK6;NFIA;NFIB;ALDH1A2;DLC1;COL12A1;WNT5A;PPARG;EFNA5* |
| FOXE1 | 10/299 | 0.001574203548 8365202 | *BCL11A;EYA2;ALDH1A2;DLC1;COL12A1;WNT5A;PPARG;LHFPL3;SLC7A11;ESR1* |
| FOXG1 | 10/299 | 0.001574203548 8365202 | *NFIA;BCL11A;CHL1;NFIB;DLC1;WNT5A;RARB;KCNQ5;ETV1;LHFPL3* |
| FOXN2 | 10/299 | 0.001574203548 8365202 | *KLF7;ZFHX3;CDK6;NFIA;TBL1XR1;NFIB;DLC1;BBX;SLC7A11;LPP* |
| FOXP4 | 10/299 | 0.001574203548 8365202 | *RERE;ZFHX3;CDK6;NFIA;BCL11A;NFIB;ZMYND8;DLC1;AFF3;ARID1B* |
| GON4L | 10/299 | 0.001574203548 8365202 | *RERE;ZFHX3;KMT2A;ZMYND8;UBR5;VPS13B;CEP192;LPP;ARID1B;MED13L* |
| HLF | 10/299 | 0.001574203548 8365202 | *THRB;NFIA;BCL11A;NFIB;DLC1;ETV1;PPARG;SORBS2;AFF3;NR3C2* |
| HMGA2 | 10/299 | 0.001574203548 8365202 | *ZFHX3;CDK6;NFIA;NFIB;DLC1;COL12A1;WNT5A;ETV1;PPARG;SLC7A11* |
| HOXC9 | 10/299 | 0.001574203548 8365202 | *ZFHX3;NFIA;EYA2;NFIB;ALDH1A2;DLC1;COL12A1;WNT5A;PPARG;FBLN2* |
| HOXD3 | 10/299 | 0.001574203548 8365202 | *ZFHX3;NFIA;NFIB;ALDH1A2;DLC1;WNT5A;RARB;ETV1;LHFPL3;EPHA3* |
| HOXD8 | 10/299 | 0.001574203548 8365202 | *NFIA;NFIB;ALDH1A2;DLC1;COL12A1;WNT5A;PPARG;LAMB1;HMCN1;FBLN2* |
| IRX6 | 10/299 | 0.001574203548 8365202 | *ZFHX3;CHL1;EYA2;NFIB;ALDH1A2;DLC1;TMTC1;PPARG;LHFPL3;EFNA5* |

| | | | |
|---|---|---|---|
| JRKL | 10/299 | 0.0015742035488365202 | *MBTD1;NFIA;TBL1XR1;BCL11A;NFIB;DLC1;BBX;LAMB1;SLC7A11;LPP* |
| KLF14 | 10/299 | 0.0015742035488365202 | *ZFHX3;NFIA;BCL11A;ALDH1A2;DLC1;PPARG;LHFPL3;EFNA5;ESR1;HCN1* |
| KLF3 | 10/299 | 0.0015742035488365202 | *KLF7;ZFHX3;CDK6;NFIA;TBL1XR1;NFIB;DLC1;NEDD4L;LPP;MED13L* |
| MET | 10/299 | 0.0015742035488365202 | *CDK6;NFIB;DLC1;COL12A1;NEDD4L;PPARG;LAMB1;SLC7A11;CLDN1;ESR1* |
| NEUROD1 | 10/299 | 0.0015742035488365202 | *GNAO1;NFIA;BCL11A;CHL1;NFIB;DLC1;ETV1;LHFPL3;EFNA5;EPHA3* |
| NFRKB | 10/299 | 0.0015742035488365202 | *RERE;NCOA2;ZFHX3;KMT2A;TBL1XR1;ZMYND8;DLC1;NEDD4L;ARID1B;MED13L* |
| NOBOX | 10/299 | 0.0015742035488365202 | *ZFHX3;KLF12;NFIA;DLC1;ETV1;PPARG;SLC7A11;EFNA5;ESR1;EPHA3* |
| NR1I2 | 10/299 | 0.0015742035488365202 | *THRB;NFIA;NFIB;DLC1;NEDD4L;RARB;PPARG;ESR1;NR3C2;RAPGEF4* |
| NR3C1 | 10/299 | 0.0015742035488365202 | *ZFHX3;CAMK2D;CDK6;NFIA;NFIB;DLC1;PPARG;APBB2;LPP;ESR1* |
| NRL | 10/299 | 0.0015742035488365202 | *ZFHX3;THRB;NFIA;BCL11A;NFIB;DLC1;PPARG;LHFPL3;SLC7A11;ESR1* |
| OTX2 | 10/299 | 0.0015742035488365202 | *ZFHX3;BCL11A;NFIB;ALDH1A2;DLC1;WNT5A;MPPED2;RARB;ETV1;LHFPL3* |
| PAX1 | 10/299 | 0.0015742035488365202 | *ZFHX3;NFIA;BCL11A;EYA2;NFIB;ALDH1A2;DLC1;WNT5A;LHFPL3;ESR1* |
| PBRM1 | 10/299 | 0.0015742035488365202 | *NCOA2;CDK6;KMT2A;TBL1XR1;NFIB;ZMYND8;UBR5;BBX;LPP;ARID1B* |
| PITX1 | 10/299 | 0.0015742035488365202 | *NFIA;BCL11A;NFIB;DLC1;COL12A1;WNT5A;PPARG;LAMB1;SLC7A11;ESR1* |
| PKNOX1 | 10/299 | 0.0015742035488365202 | *RERE;ZFHX3;CDK6;NFIA;TBL1XR1;NFIB;ZMYND8;DLC1;NEDD4L;LPP* |
| PRDM7 | 10/299 | 0.0015742035488365202 | *ZFHX3;NFIA;BCL11A;DLC1;MX1;NEDD4L;LHFPL3;SLC7A11;EFNA5;AFF3* |
| PRDM8 | 10/299 | 0.0015742035488365202 | *ANGPT1;NFIA;BCL11A;CHL1;NFIB;DLC1;NEDD4L;KCNQ5;SLC7A11;AFF3* |
| PRRX1 | 10/299 | 0.0015742035488365202 | *ANGPT1;NFIA;NFIB;DLC1;COL12A1;WNT5A;LAMB1;HMCN1;FBLN2;EPHA3* |
| SCML4 | 10/299 | 0.0015742035488365202 | *ZFHX3;KLF12;NFIA;ST8SIA1;BCL11A;DLC1;AFF3;ESR1;KBTBD11;RAPGEF4* |
| SIX4 | 10/299 | 0.0015742035488365202 | *ZFHX3;CDK6;NFIA;NFIB;DLC1;COL12A1;WNT5A;ETV1;SLC7A11;EFNA5* |
| SORBS2 | 10/299 | 0.0015742035488365202 | *GNAO1;CAMK2D;NFIA;NFIB;DLC1;COL12A1;PRICKLE2;NEDD4L;SHROOM3;LPP* |
| SOX1 | 10/299 | 0.0015742035488365202 | *ZFHX3;NFIA;BCL11A;NFIB;DLC1;WNT5A;LHFPL3;EFNA5;EPHA3;NPAS3* |
| SOX21 | 10/299 | 0.0015742035488365202 | *MEGF10;NFIA;BCL11A;EYA2;NFIB;DLC1;WNT5A;LHFPL3;SLC7A11;NPAS3* |
| SRCAP | 10/299 | 0.0015742035488365202 | *RERE;ZFHX3;KMT2A;TBL1XR1;NFIB;DLC1;UBR5;LPP;ARID1B;MED13L* |
| TAL2 | 10/299 | 0.0015742035488365202 | *BCL11A;ALDH1A2;DLC1;COL12A1;RARB;ETV1;PPARG;LHFPL3;SLC7A11;EFNA5* |
| TBX1 | 10/299 | 0.0015742035488365202 | *NFIA;BCL11A;EYA2;NFIB;ALDH1A2;DLC1;WNT5A;PPARG;CACNA1C;ESR1* |
| TBX2 | 10/299 | 0.0015742035488365202 | *ZFHX3;NFIA;NFIB;DLC1;COL12A1;WNT5A;ETV1;PPARG;LAMB1;EPHA3* |
| TP73 | 10/299 | 0.0015742035488365202 | *CDK6;THRB;NFIA;BCL11A;NFIB;DLC1;PPARG;LHFPL3;CACNA1C;ESR1* |
| TSHZ1 | 10/299 | 0.0015742035488365202 | *ZFHX3;KLF12;NFIA;BCL11A;NFIB;ZMYND8;DLC1;EFNA5;AFF3;ARID1B* |
| WT1 | 10/299 | 0.001574203548 | *CDK6;NFIA;BCL11A;NFIB;ALDH1A2;DLC1;COL12A1;WNT5A;PPARG;ESR1* |

| | | | |
|---|---|---|---|
| | | 8365202 | |
| YY2 | 10/299 | 0.001574203548 8365202 | *ZFHX3;KLF12;THRB;NFIA;NFIB;DLC1;ETV1;PPARG;SLC7A11;LPP* |
| ZBED3 | 10/299 | 0.001574203548 8365202 | *ZFHX3;CDK6;NFIA;BCL11A;NFIB;DLC1;NEDD4L;LAMB1;SLC7A11;LPP* |
| ZBTB41 | 10/299 | 0.001574203548 8365202 | *NCOA2;KLF7;KLF12;CDK6;NFIA;TBL1XR1;NFIB;BBX;SLC7A11;LPP* |
| ZBTB47 | 10/299 | 0.001574203548 8365202 | *CTIF;KLF7;ZFHX3;KLF12;CDK6;NFIA;NFIB;DLC1;FBLN2;LPP* |
| ZFYVE26 | 10/299 | 0.001574203548 8365202 | *RERE;NCOA2;ZFHX3;CDK6;KMT2A;ZMYND8;DLC1;NEDD4L;LPP;MED13L* |
| ZHX1 | 10/299 | 0.001574203548 8365202 | *NCOA2;ZFHX3;CDK6;ANGPT1;NFIA;TBL1XR1;NFIB;DLC1;BBX;LPP* |
| ZIC3 | 10/299 | 0.001574203548 8365202 | *CECR2;ZFHX3;BCL11A;NFIB;ALDH1A2;DLC1;WNT5A;MPPED2;ETV1;LHFPL3* |
| ZMAT1 | 10/299 | 0.001574203548 8365202 | *ZFHX3;KLF12;NFIA;DAPK1;NFIB;DLC1;SORBS2;SLC7A11;IKZF2;NR3C2* |
| ZNF157 | 10/299 | 0.001574203548 8365202 | *MEGF10;DLC1;MX1;DLGAP1;SORBS2;LHFPL3;CACNA1C;PAK3;CLDN1;NR3C2* |
| ZNF18 | 10/299 | 0.001574203548 8365202 | *RERE;KLF7;ZFHX3;NFIA;BCL11A;ZMYND8;DLC1;NEDD4L;AFF3;ARID1B* |
| ZNF253 | 10/299 | 0.001574203548 8365202 | *MBTD1;KLF12;CDK6;ZNF607;NFIA;BCL11A;NFIB;ZNF429;DLC1;ZNF615* |
| ZNF264 | 10/299 | 0.001574203548 8365202 | *KLF7;ZFHX3;KLF12;CDK6;KMT2A;NFIA;NFIB;DLC1;SLC7A11;LPP* |
| ZNF28 | 10/299 | 0.001574203548 8365202 | *KLF12;CDK6;ZNF607;NFIB;ZNF429;ZNF615;APBB2;SLC7A11;LPP;ZNF234* |
| ZNF286A | 10/299 | 0.001574203548 8365202 | *KLF12;CDK6;DAPK1;BCL11A;NFIB;DLC1;BBX;NEDD4L;SLC7A11;LPP* |
| ZNF343 | 10/299 | 0.001574203548 8365202 | *ZFHX3;CDK6;NFIA;ZMYND8;DLC1;MX1;SLC7A11;AFF3;ZNF521;ZNF234* |
| ZNF37A | 10/299 | 0.001574203548 8365202 | *KLF7;ZFHX3;KLF12;CDK6;KMT2A;NFIA;NFIB;BBX;SLC7A11;LPP* |
| ZNF420 | 10/299 | 0.001574203548 8365202 | *KLF12;CDK6;ZNF607;DAPK1;BCL11A;NFIB;DLC1;ZNF615;SLC7A11;NR3C2* |
| ZNF431 | 10/299 | 0.001574203548 8365202 | *MBTD1;KLF12;CDK6;NFIA;BCL11A;NFIB;ZNF429;DLC1;BBX;LPP* |
| ZNF461 | 10/299 | 0.001574203548 8365202 | *KLF7;KLF12;ZNF607;ZNF429;DLC1;ZNF615;SLC7A11;ZNF665;ZNF234;NR3C2* |
| ZNF484 | 10/299 | 0.001574203548 8365202 | *ZFHX3;KLF12;CDK6;TBL1XR1;COL12A1;ZNF615;BBX;VPS13B;SLC7A11;LPP* |
| ZNF510 | 10/299 | 0.001574203548 8365202 | *MBTD1;NCOA2;KLF12;ZNF197;KMT2A;NFIA;ZNF615;BBX;RAB3GAP2;CEP192* |
| ZNF543 | 10/299 | 0.001574203548 8365202 | *KLF7;ZFHX3;NFIA;BCL11A;NFIB;ZNF429;ZNF615;SLC7A11;ZNF665;ZNF234* |
| ZNF559 | 10/299 | 0.001574203548 8365202 | *CDK6;NFIA;TBL1XR1;BCL11A;NFIB;LAMB1;ARID1B;NR3C2;MED13L;RAPGEF4* |
| ZNF560 | 10/299 | 0.001574203548 8365202 | *CECR2;PDE1C;CHL1;DLC1;ZNF536;PPARG;LHFPL3;HMCN1;CACNA1C;FBLN2* |
| ZNF571 | 10/299 | 0.001574203548 8365202 | *ZNF607;NFIA;BCL11A;DLC1;MX1;ZNF615;SLC7A11;IKZF2;ZNF234;NR3C2* |
| ZNF607 | 10/299 | 0.001574203548 8365202 | *NFIA;BCL11A;NFIB;ZNF429;DLC1;ZNF615;NEDD4L;ZNF665;AFF3;ZNF234* |
| ZNF613 | 10/299 | 0.001574203548 8365202 | *ZNF607;DAPK1;NFIB;ZNF429;DLC1;MX1;ZNF615;NEDD4L;SLC7A11;ZNF234* |
| ZNF615 | 10/299 | 0.001574203548 8365202 | *CDK6;BCL11A;NFIB;ZNF429;DLC1;MX1;BBX;NEDD4L;LPP;ZNF234* |
| ZNF623 | 10/299 | 0.001574203548 8365202 | *NCOA2;KLF7;ZFHX3;KLF12;CDK6;NFIB;DLC1;NEDD4L;ARID1B;MED13L* |

| | | | |
|---|---|---|---|
| ZNF645 | 10/299 | 0.0015742035488365202 | *MAPK10;ZFHX3;NFIB;DLC1;ETV1;LHFPL3;SLC7A11;LPP;ESR1;CDK14* |
| ZNF708 | 10/299 | 0.0015742035488365202 | *MBTD1;ZFHX3;KLF12;CDK6;NFIA;NFIB;ZNF429;DLC1;BBX;LPP* |
| ZNF710 | 10/299 | 0.0015742035488365202 | *RERE;KLF7;ZFHX3;CDK6;NFIA;ZMYND8;DLC1;SLC7A11;LPP;ARID1B* |
| ZNF716 | 10/299 | 0.0015742035488365202 | *PDE1C;BCL11A;DLC1;TMTC1;LHFPL3;PRKD1;SLC7A11;LPP;EPHA3;HCN1* |
| ZNF765 | 10/299 | 0.0015742035488365202 | *KLF12;CDK6;ZNF429;DLC1;ZNF615;BBX;IKZF2;ZNF665;LPP;ZNF234* |
| ZNF805 | 10/299 | 0.0015742035488365202 | *KLF12;CAMK2D;CDK6;KMT2A;NFIA;DLC1;VPS13B;SLC7A11;LPP;MED13L* |
| ZNF81 | 10/299 | 0.0015742035488365202 | *NCOA2;ZFHX3;KLF12;CDK6;KMT2A;UBR5;DMXL2;BBX;VPS13B;LPP* |
| ZNF835 | 10/299 | 0.0015742035488365202 | *DAPK1;ST8SIA1;BCL11A;DLC1;DLGAP1;APBB2;SORBS2;LHFPL3;HMCN1;NR3C2* |
| ZNF99 | 10/299 | 0.0015742035488365202 | *ZFHX3;NFIB;ZNF429;DLC1;COL12A1;LHFPL3;ZNF665;AFF3;TTN;HCN1* |
| ADNP2 | 9/299 | 0.005196923563465128 | *ZFHX3;CDK6;TBL1XR1;ZMYND8;DLC1;NEDD4L;SLC7A11;ARID1B;MED13L* |
| BDP1 | 9/299 | 0.005196923563465128 | *NCOA2;KMT2A;TBL1XR1;UBR5;DMXL2;BBX;VPS13B;LPP;MED13L* |
| CREB3L2 | 9/299 | 0.005196923563465128 | *ZFHX3;CDK6;NFIA;NFIB;DLC1;NEDD4L;LAMB1;SLC7A11;LPP* |
| DHX57 | 9/299 | 0.005196923563465128 | *ZFHX3;NFIA;NFIB;ZMYND8;DLC1;UBR5;NEDD4L;SORBS2;LPP* |
| DMRTA2 | 9/299 | 0.005196923563465128 | *ZFHX3;NFIA;BCL11A;EYA2;NFIB;DLC1;WNT5A;LHFPL3;EFNA5* |
| DMTF1 | 9/299 | 0.005196923563465128 | *CDK6;DLC1;BBX;ETV1;PPARG;APBB2;SLC7A11;LPP;EPHA3* |
| DZIP1L | 9/299 | 0.005196923563465128 | *ZFHX3;DLC1;COL12A1;WNT5A;LAMB1;HMCN1;SLC7A11;FBLN2;GULP1* |
| EBF4 | 9/299 | 0.005196923563465128 | *NFIA;EYA2;DLC1;COL12A1;WNT5A;LHFPL3;EFNA5;AFF3;FBLN2* |
| EHF | 9/299 | 0.005196923563465128 | *NFIA;ATP8B1;NFIB;DLC1;WNT5A;SHROOM3;PPARG;SLC7A11;CLDN1* |
| EMX1 | 9/299 | 0.005196923563465128 | *ZFHX3;NFIA;BCL11A;NFIB;ALDH1A2;LHFPL3;EFNA5;AFF3;NPAS3* |
| EXOC2 | 9/299 | 0.005196923563465128 | *ZFHX3;CAMK2D;CDK6;NFIA;TBL1XR1;DLC1;UBR5;LPP;ARID1B* |
| FEZF1 | 9/299 | 0.005196923563465128 | *ZFHX3;MEGF10;NFIA;BCL11A;CHL1;NFIB;ALDH1A2;LHFPL3;EFNA5* |
| FEZF2 | 9/299 | 0.005196923563465128 | *NFIA;BCL11A;CHL1;NFIB;DLC1;DLGAP1;LHFPL3;EFNA5;HCN1* |
| FOXD4L5 | 9/299 | 0.005196923563465128 | *NFIA;TBL1XR1;BCL11A;NFIB;ALDH1A2;DLC1;LHFPL3;EPHA3;HCN1* |
| FOXO6 | 9/299 | 0.005196923563465128 | *GNAO1;ZFHX3;NFIA;BCL11A;NFIB;DLC1;LHFPL3;EFNA5;KBTBD11* |
| GATA3 | 9/299 | 0.005196923563465128 | *ZFHX3;CDK6;NFIA;BCL11A;NFIB;DLC1;WNT5A;PPARG;ESR1* |
| GATA6 | 9/299 | 0.005196923563465128 | *ZFHX3;NFIA;NFIB;DLC1;COL12A1;WNT5A;PPARG;EFNA5;CLDN1* |
| GBX2 | 9/299 | 0.005196923563465128 | *ZFHX3;BCL11A;NFIB;ALDH1A2;DLC1;WNT5A;ETV1;EFNA5;AFF3* |
| GCM1 | 9/299 | 0.005196923563465128 | *ZFHX3;THRB;NFIA;DLC1;WNT5A;PPARG;SLC7A11;ESR1;NR3C2* |
| GTF2IRD2 | 9/299 | 0.005196923563465128 | *ZFHX3;NFIA;DAPK1;NFIB;ZMYND8;DLC1;SORBS2;SRGAP3;SLC7A11* |
| HOXA13 | 9/299 | 0.005196923563 | *ZFHX3;NFIA;BCL11A;NFIB;ALDH1A2;DLC1;WNT5A;PPARG;SLC7A11* |

| | | | |
|---|---|---|---|
| | | 465128 | |
| HOXA6 | 9/299 | 0.005196923563 465128 | *ZFHX3;NFIA;BCL11A;NFIB;ALDH1A2;DLC1;COL12A1;WNT5A;ESR1* |
| HOXC5 | 9/299 | 0.005196923563 465128 | *ZFHX3;NFIA;NFIB;ALDH1A2;DLC1;COL12A1;WNT5A;RARB;EFNA5* |
| HOXC8 | 9/299 | 0.005196923563 465128 | *ZFHX3;NFIA;NFIB;ALDH1A2;DLC1;COL12A1;WNT5A;PPARG;FBLN2* |
| HOXD10 | 9/299 | 0.005196923563 465128 | *NFIA;BCL11A;NFIB;ALDH1A2;DLC1;COL12A1;WNT5A;PPARG;ESR1* |
| HOXD9 | 9/299 | 0.005196923563 465128 | *ZFHX3;EYA2;ALDH1A2;DLC1;COL12A1;WNT5A;PPARG;LHFPL3;FBLN2* |
| HSF5 | 9/299 | 0.005196923563 465128 | *ZFHX3;AFAP1L2;NFIA;BCL11A;DLC1;LHFPL3;AFF3;NPAS3;TTN* |
| INSM2 | 9/299 | 0.005196923563 465128 | *NFIA;BCL11A;CHL1;EYA2;DLGAP1;LHFPL3;EFNA5;NPAS3;RAPGEF4* |
| IRX4 | 9/299 | 0.005196923563 465128 | *BCL11A;EYA2;NFIB;ALDH1A2;DLC1;COL12A1;WNT5A;PPARG;LHFPL3* |
| ISL2 | 9/299 | 0.005196923563 465128 | *ZFHX3;NFIA;BCL11A;NFIB;DLC1;WNT5A;LHFPL3;SLC7A11;ESR1* |
| ISX | 9/299 | 0.005196923563 465128 | *THRB;NFIA;DLC1;PRKG2;PPARG;LHFPL3;SLC7A11;ESR1;NR3C2* |
| KLF13 | 9/299 | 0.005196923563 465128 | *RERE;KLF7;ZFHX3;CDK6;NFIA;NFIB;DLC1;NEDD4L;LPP* |
| KLF7 | 9/299 | 0.005196923563 465128 | *ZFHX3;CDK6;NFIA;NFIB;DLC1;NEDD4L;APBB2;SLC7A11;LPP* |
| LEF1 | 9/299 | 0.005196923563 465128 | *CDK6;NFIA;BCL11A;NFIB;DLC1;WNT5A;PPARG;SLC7A11;AFF3* |
| LHX1 | 9/299 | 0.005196923563 465128 | *ZFHX3;NFIA;BCL11A;NFIB;ALDH1A2;DLC1;WNT5A;RARB;LHFPL3* |
| LHX6 | 9/299 | 0.005196923563 465128 | *ZFHX3;NFIA;BCL11A;NFIB;ALDH1A2;DLC1;COL12A1;PPARG;FBLN2* |
| MEF2C | 9/299 | 0.005196923563 465128 | *CAMK2D;CDK6;ANGPT1;NFIA;BCL11A;NFIB;DLC1;ETV1;SORBS2* |
| MINK1 | 9/299 | 0.005196923563 465128 | *RERE;CAMK2D;DAPK1;ZMYND8;DLC1;NEDD4L;PTK2B;SORBS2;ARID1B* |
| MYCN | 9/299 | 0.005196923563 465128 | *CDK6;NFIA;BCL11A;NFIB;DLC1;WNT5A;MPPED2;ETV1;ESR1* |
| MYSM1 | 9/299 | 0.005196923563 465128 | *MBTD1;NCOA2;KMT2A;NFIB;DMXL2;BBX;VPS13B;LPP;MED13L* |
| NEUROG1 | 9/299 | 0.005196923563 465128 | *ZFHX3;NFIA;ALDH1A2;DLC1;WNT5A;RARB;PPARG;LHFPL3;NPAS3* |
| NFATC4 | 9/299 | 0.005196923563 465128 | *NFIA;DLC1;COL12A1;WNT5A;ETV1;PPARG;LAMB1;FBLN2;EPHA3* |
| NFIC | 9/299 | 0.005196923563 465128 | *RERE;ZFHX3;CDK6;NFIA;NFIB;ZMYND8;DLC1;LPP;ARID1B* |
| NR0B1 | 9/299 | 0.005196923563 465128 | *CDK6;ALDH1A2;DLC1;WNT5A;MPPED2;RARB;PPARG;SLC7A11;ESR1* |
| OLIG2 | 9/299 | 0.005196923563 465128 | *NFIA;BCL11A;NFIB;DLC1;ETV1;PPARG;LHFPL3;SLC7A11;NPAS3* |
| PAX2 | 9/299 | 0.005196923563 465128 | *ZFHX3;NFIA;BCL11A;NFIB;DLC1;ETV1;LHFPL3;EFNA5;ESR1* |
| PRDM13 | 9/299 | 0.005196923563 465128 | *NFIA;BCL11A;CHL1;NFIB;ALDH1A2;DLC1;WNT5A;LHFPL3;EFNA5* |
| RC3H2 | 9/299 | 0.005196923563 465128 | *NCOA2;ZFHX3;KMT2A;TBL1XR1;NFIB;UBR5;BBX;LPP;MED13L* |
| RUNX1 | 9/299 | 0.005196923563 465128 | *ZFHX3;CDK6;NFIA;DLC1;COL12A1;PPARG;SLC7A11;LPP;ESR1* |
| SATB1 | 9/299 | 0.005196923563 465128 | *CDK6;THRB;NFIA;BCL11A;NFIB;DLC1;ETV1;PPARG;AFF3* |

| | | | |
|---|---|---|---|
| SETDB1 | 9/299 | 0.005196923563465128 | *RERE;NCOA2;ZFHX3;KMT2A;NFIA;TBL1XR1;ZMYND8;UBR5;ARID1B* |
| SMAD5 | 9/299 | 0.005196923563465128 | *ZFHX3;CDK6;NFIA;TBL1XR1;NFIB;DLC1;BBX;LPP;MED13L* |
| SMARCC2 | 9/299 | 0.005196923563465128 | *RERE;KMT2A;NFIA;TBL1XR1;NFIB;ZMYND8;UBR5;ARID1B;MED13L* |
| SOX10 | 9/299 | 0.005196923563465128 | *MEGF10;NFIA;BCL11A;CHL1;NFIB;ALDH1A2;DLC1;ETV1;LHFPL3* |
| SP2 | 9/299 | 0.005196923563465128 | *RERE;NCOA2;ZFHX3;KMT2A;NFIA;NFIB;ZMYND8;DLC1;NEDD4L* |
| SP3 | 9/299 | 0.005196923563465128 | *NCOA2;ZFHX3;NFIA;TBL1XR1;NFIB;UBR5;LPP;ARID1B;MED13L* |
| TBX19 | 9/299 | 0.005196923563465128 | *COL24A1;NFIA;BCL11A;DLC1;HMCN1;SLC7A11;EFNA5;AFF3;ESR1* |
| TCF7L1 | 9/299 | 0.005196923563465128 | *ZFHX3;CDK6;NFIA;BCL11A;NFIB;DLC1;WNT5A;ETV1;LAMB1* |
| TERF2 | 9/299 | 0.005196923563465128 | *RERE;NCOA2;ZFHX3;CDK6;NFIA;TBL1XR1;ZMYND8;UBR5;ARID1B* |
| THAP6 | 9/299 | 0.005196923563465128 | *ZFHX3;KLF12;CAMK2D;CDK6;TBL1XR1;DLC1;BBX;SLC7A11;LPP* |
| TSC22D2 | 9/299 | 0.005196923563465128 | *KLF7;ZFHX3;CDK6;NFIA;NFIB;DLC1;BBX;LPP;MED13L* |
| UNKL | 9/299 | 0.005196923563465128 | *RERE;ZFHX3;NFIA;NFIB;ZMYND8;DLC1;NEDD4L;SLC7A11;LPP* |
| ZBTB16 | 9/299 | 0.005196923563465128 | *ZFHX3;NFIA;MAOA;NFIB;DLC1;NEDD4L;PPARG;AFF3;ACACB* |
| ZBTB24 | 9/299 | 0.005196923563465128 | *ZFHX3;CDK6;NFIA;BCL11A;ZMYND8;DLC1;NEDD4L;LPP;ARID1B* |
| ZC3H6 | 9/299 | 0.005196923563465128 | *KLF7;ZFHX3;KLF12;NFIA;NFIB;DLC1;NEDD4L;SLC7A11;LPP* |
| ZCCHC6 | 9/299 | 0.005196923563465128 | *RERE;NCOA2;ZFHX3;ZMYND8;UBR5;BBX;NEDD4L;LPP;MED13L* |
| ZNF177 | 9/299 | 0.005196923563465128 | *CDK6;BCL11A;NFIB;DLC1;PELI2;NEDD4L;LHFPL3;LAMB1;NR3C2* |
| ZNF184 | 9/299 | 0.005196923563465128 | *ZFHX3;KLF12;CDK6;NFIA;TBL1XR1;BCL11A;NFIB;DLC1;SLC7A11* |
| ZNF20 | 9/299 | 0.005196923563465128 | *ZFHX3;ZNF607;NFIA;BCL11A;ZNF429;DLC1;WNT5A;MX1;ZNF615* |
| ZNF215 | 9/299 | 0.005196923563465128 | *KIF18A;BCL11A;DLC1;NEDD4L;ETV1;PPARG;APBB2;LHFPL3;SLC7A11* |
| ZNF250 | 9/299 | 0.005196923563465128 | *ZFHX3;KMT2A;NFIA;BCL11A;NFIB;DLC1;PELI2;BBX;NEDD4L* |
| ZNF3 | 9/299 | 0.005196923563465128 | *ZFHX3;CDK6;NFIA;TBL1XR1;BCL11A;NFIB;ZMYND8;DLC1;NEDD4L* |
| ZNF30 | 9/299 | 0.005196923563465128 | *KLF12;ZNF607;BCL11A;DLC1;MX1;ZNF615;NEDD4L;SLC7A11;AFF3* |
| ZNF329 | 9/299 | 0.005196923563465128 | *ZFHX3;KLF12;CDK6;DLC1;MX1;ZNF615;NEDD4L;SLC7A11;AFF3* |
| ZNF333 | 9/299 | 0.005196923563465128 | *RERE;KLF7;ZFHX3;KLF12;KMT2A;NFIA;DLC1;VPS13B;MED13L* |
| ZNF34 | 9/299 | 0.005196923563465128 | *RERE;ZFHX3;KMT2A;NFIB;DLC1;PELI2;ZNF615;NEDD4L;SORBS2* |
| ZNF41 | 9/299 | 0.005196923563465128 | *NCOA2;KLF12;NFIA;DMXL2;ZNF615;BBX;VPS13B;IKZF2;ZNF234* |
| ZNF415 | 9/299 | 0.005196923563465128 | *BCL11A;NFIB;DLC1;MX1;NEDD4L;SORBS2;ZNF665;ZNF521;NR3C2* |
| ZNF418 | 9/299 | 0.005196923563465128 | *BCL11A;DLC1;MX1;TMTC2;SORBS2;LHFPL3;ZNF665;AFF3;NR3C2* |
| ZNF426 | 9/299 | 0.005196923563 | *KLF7;KLF12;CDK6;NFIB;ZNF429;DLC1;ZNF615;BBX;SLC7A11* |

| | | | |
|---|---|---|---|
| | | 465128 | |
| ZNF43 | 9/299 | 0.005196923563 465128 | *MBTD1;CDK6;BCL11A;NFIB;ZNF429;DLC1;NEDD4L;ETV1;NR3C2* |
| ZNF451 | 9/299 | 0.005196923563 465128 | *CDK6;KMT2A;TBL1XR1;NFIB;ZMYND8;UBR5;BBX;LPP;MED13L* |
| ZNF501 | 9/299 | 0.005196923563 465128 | *ZNF197;DLC1;C2ORF88;ZNF615;LHFPL3;IL1RAP;CACNA1C;CLDN1;LPP* |
| ZNF528 | 9/299 | 0.005196923563 465128 | *MAOA;NFIB;ZNF429;DLC1;COL12A1;MX1;ZNF615;ZNF665;ZNF234* |
| ZNF552 | 9/299 | 0.005196923563 465128 | *NCOA2;ZFHX3;NFIA;DAPK1;ATP8B1;MX1;NEDD4L;AFF3;ESR1* |
| ZNF557 | 9/299 | 0.005196923563 465128 | *KLF12;CDK6;TBL1XR1;BCL11A;NFIB;DLC1;BBX;SLC7A11;LPP* |
| ZNF578 | 9/299 | 0.005196923563 465128 | *NFIA;CHL1;ZNF429;DLC1;TMTC1;SORBS2;LHFPL3;SLC7A11;ZNF665* |
| ZNF595 | 9/299 | 0.005196923563 465128 | *CDK6;NFIA;NFIB;ZMYND8;ZNF429;DLC1;SLC7A11;AFF3;EPHA3* |
| ZNF641 | 9/299 | 0.005196923563 465128 | *KLF7;ZFHX3;KLF12;CDK6;NFIA;NFIB;DLC1;SLC7A11;LPP* |
| ZNF658 | 9/299 | 0.005196923563 465128 | *BCL11A;EYA2;DLC1;WNT5A;HMCN1;SLC7A11;CLDN1;ZNF521;NR3C2* |
| ZNF680 | 9/299 | 0.005196923563 465128 | *MBTD1;CDK6;NFIA;BCL11A;NFIB;ZNF429;ZNF615;BBX;SLC7A11* |
| ZNF740 | 9/299 | 0.005196923563 465128 | *RERE;KLF7;ZFHX3;KMT2A;NFIA;TBL1XR1;NFIB;ZMYND8;NEDD4L* |
| ZNF776 | 9/299 | 0.005196923563 465128 | *MBTD1;NCOA2;KLF12;KMT2A;NFIB;ZNF615;BBX;LPP;ZNF234* |
| ZNF783 | 9/299 | 0.005196923563 465128 | *RERE;KLF7;ZFHX3;GABBR1;NFIA;BCL11A;DLC1;NEDD4L;SLC7A11* |
| ZNF792 | 9/299 | 0.005196923563 465128 | *KLF7;ZFHX3;KLF12;CDK6;NFIA;BCL11A;DLC1;MX1;ZNF521* |
| ZNF829 | 9/299 | 0.005196923563 465128 | *KLF12;CDK6;ZNF607;NFIB;ZNF429;ZNF615;SLC7A11;IKZF2;ZNF234* |
| ZSCAN2 | 9/299 | 0.005196923563 465128 | *RERE;ZFHX3;NFIA;BCL11A;NFIB;ZMYND8;DLC1;NEDD4L;TTC28* |
| ZSCAN22 | 9/299 | 0.005196923563 465128 | *KLF7;ZFHX3;CDK6;NFIA;DAPK1;NFIB;ZMYND8;DLC1;ETV1* |
| ZSCAN4 | 9/299 | 0.005196923563 465128 | *THRB;NFIA;BCL11A;NFIB;DLC1;PPARG;LHFPL3;EPHA3;NR3C2* |
| ZXDB | 9/299 | 0.005196923563 465128 | *ZFHX3;KLF12;CDK6;NFIB;DLC1;BBX;NEDD4L;SLC7A11;LPP* |
| ARID5B | 8/299 | 0.015532229583 974648 | *KLF7;ZFHX3;CDK6;NFIA;NFIB;DLC1;NEDD4L;LPP* |
| ATF6 | 8/299 | 0.015532229583 974648 | *RERE;CDK6;NFIA;TBL1XR1;DLC1;PTK2B;PPARG;LPP* |
| ATOH7 | 8/299 | 0.015532229583 974648 | *THRB;NFIA;BCL11A;DLC1;KCNQ5;ETV1;LHFPL3;SLC7A11* |
| ATOH8 | 8/299 | 0.015532229583 974648 | *NFIA;NFIB;DLC1;COL12A1;WNT5A;PPARG;SORBS2;FBLN2* |
| BARHL2 | 8/299 | 0.015532229583 974648 | *ZFHX3;NFIA;BCL11A;EYA2;ALDH1A2;DLC1;LHFPL3;EFNA5* |
| CDX2 | 8/299 | 0.015532229583 974648 | *ZFHX3;CDK6;BCL11A;NFIB;DLC1;WNT5A;PPARG;ESR1* |
| CDX4 | 8/299 | 0.015532229583 974648 | *ALDH1A2;DLC1;WNT5A;PPARG;LHFPL3;SLC7A11;EFNA5;PAK3* |
| CPXCR1 | 8/299 | 0.015532229583 974648 | *ADGRB3;ST8SIA1;CHL1;DLC1;KCNQ5;LHFPL3;EPHA3;NPAS3* |
| CTCF | 8/299 | 0.015532229583 974648 | *ZFHX3;KMT2A;TBL1XR1;BCL11A;NFIB;UBR5;ARID1B;MED13L* |

| | | | |
|---|---|---|---|
| DEAF1 | 8/299 | 0.015532229583974648 | *RERE;ZFHX3;NFIA;BCL11A;ZMYND8;DLC1;NEDD4L;ARID1B* |
| DLX4 | 8/299 | 0.015532229583974648 | *ZFHX3;NFIA;NFIB;ALDH1A2;DLC1;WNT5A;PPARG;ESR1* |
| DMBX1 | 8/299 | 0.015532229583974648 | *NFIA;BCL11A;NFIB;ALDH1A2;DLC1;WNT5A;LHFPL3;EFNA5* |
| DMRT1 | 8/299 | 0.015532229583974648 | *ZFHX3;NFIA;BCL11A;NFIB;DLC1;PPARG;LHFPL3;ESR1* |
| DMRT3 | 8/299 | 0.015532229583974648 | *ZFHX3;NFIA;BCL11A;EYA2;NFIB;ALDH1A2;DLC1;LHFPL3* |
| FOXL2 | 8/299 | 0.015532229583974648 | *BCL11A;ALDH1A2;DLC1;COL12A1;WNT5A;PPARG;LHFPL3;ESR1* |
| FOXR2 | 8/299 | 0.015532229583974648 | *PDE1C;DLC1;WNT5A;ETV1;SORBS2;LHFPL3;SLC7A11;PAK3* |
| GCM2 | 8/299 | 0.015532229583974648 | *GNAO1;ZFHX3;THRB;NFIA;DLC1;PPARG;LHFPL3;ESR1* |
| GRHL1 | 8/299 | 0.015532229583974648 | *THRB;NFIA;NFIB;DLC1;PPARG;SLC7A11;IKZF2;CLDN1* |
| HNF1B | 8/299 | 0.015532229583974648 | *ZFHX3;THRB;NFIA;BCL11A;NFIB;DLC1;PPARG;NR3C2* |
| HOXA4 | 8/299 | 0.015532229583974648 | *ZFHX3;NFIA;BCL11A;NFIB;ALDH1A2;DLC1;WNT5A;PPARG* |
| HOXA7 | 8/299 | 0.015532229583974648 | *ZFHX3;NFIA;NFIB;ALDH1A2;DLC1;COL12A1;WNT5A;PPARG* |
| HOXB1 | 8/299 | 0.015532229583974648 | *ZFHX3;NFIA;BCL11A;ALDH1A2;DLC1;WNT5A;RARB;ESR1* |
| HOXB5 | 8/299 | 0.015532229583974648 | *ZFHX3;CDK6;NFIA;ALDH1A2;DLC1;COL12A1;WNT5A;PPARG* |
| HOXB9 | 8/299 | 0.015532229583974648 | *CDK6;DLC1;COL12A1;WNT5A;PPARG;LAMB1;SLC7A11;CLDN1* |
| HOXC13 | 8/299 | 0.015532229583974648 | *BCL11A;NFIB;DLC1;COL12A1;WNT5A;PPARG;SLC7A11;CLDN1* |
| HOXC6 | 8/299 | 0.015532229583974648 | *NFIB;DLC1;COL12A1;WNT5A;PPARG;LAMB1;SLC7A11;FBLN2* |
| HOXD11 | 8/299 | 0.015532229583974648 | *BCL11A;ALDH1A2;DLC1;COL12A1;WNT5A;PPARG;LHFPL3;ESR1* |
| HSF2 | 8/299 | 0.015532229583974648 | *KLF12;CAMK2D;NFIA;BCL11A;NFIB;DLC1;ETV1;SLC7A11* |
| INSM1 | 8/299 | 0.015532229583974648 | *GNAO1;ZFHX3;NFIA;BCL11A;EYA2;NFIB;LHFPL3;EFNA5* |
| KLF11 | 8/299 | 0.015532229583974648 | *KLF7;ZFHX3;CDK6;NFIA;NFIB;DLC1;NEDD4L;LPP* |
| KLF17 | 8/299 | 0.015532229583974648 | *DLC1;NEDD4L;PPARG;LHFPL3;HMCN1;SLC7A11;AFF3;ESR1* |
| MBNL1 | 8/299 | 0.015532229583974648 | *ZFHX3;CAMK2D;CDK6;NFIA;TBL1XR1;NFIB;DLC1;LPP* |
| MEF2D | 8/299 | 0.015532229583974648 | *RERE;KLF7;ZFHX3;CDK6;NFIA;NFIB;ZMYND8;NEDD4L* |
| MLLT1 | 8/299 | 0.015532229583974648 | *RERE;ZFHX3;CDK6;NFIA;NFIB;ZMYND8;DLC1;ARID1B* |
| MYF5 | 8/299 | 0.015532229583974648 | *PDE1C;NFIA;DLC1;COL12A1;WNT5A;PPARG;SLC7A11;ESR1* |
| MYNN | 8/299 | 0.015532229583974648 | *KLF12;CAMK2D;KMT2A;NFIA;TBL1XR1;BBX;VPS13B;LPP* |
| NEUROD6 | 8/299 | 0.015532229583974648 | *NFIA;BCL11A;CHL1;NFIB;DLGAP1;LHFPL3;EPHA3;HCN1* |
| NFE2L3 | 8/299 | 0.015532229583974648 | *CDK6;DLC1;WNT5A;MX1;NEDD4L;PPARG;SLC7A11;CLDN1* |
| NHLH1 | 8/299 | 0.015532229583 | *NFIA;BCL11A;EYA2;NFIB;DLC1;LHFPL3;SLC7A11;EPHA3* |

| | | | |
|---|---|---|---|
| | | 974648 | |
| OLIG3 | 8/299 | 0.015532229583974648 | *NFIA;BCL11A;ALDH1A2;DLC1;WNT5A;LHFPL3;EFNA5;ESR1* |
| ONECUT1 | 8/299 | 0.015532229583974648 | *ZFHX3;NFIA;BCL11A;NFIB;DLC1;PPARG;LHFPL3;ESR1* |
| ONECUT3 | 8/299 | 0.015532229583974648 | *ZFHX3;NFIA;BCL11A;NFIB;LHFPL3;EFNA5;ESR1;NPAS3* |
| PATZ1 | 8/299 | 0.015532229583974648 | *RERE;ZFHX3;CDK6;NFIA;BCL11A;NFIB;ZMYND8;ARID1B* |
| PAWR | 8/299 | 0.015532229583974648 | *CDK6;NFIB;DLC1;COL12A1;NEDD4L;SHROOM3;SLC7A11;LPP* |
| PBX2 | 8/299 | 0.015532229583974648 | *RERE;ZFHX3;NFIA;TBL1XR1;NFIB;ZMYND8;DLC1;LPP* |
| PCSK6 | 8/299 | 0.015532229583974648 | *NFIA;NFIB;DLC1;COL12A1;PPARG;SORBS2;SLC7A11;FBLN2* |
| PLAGL1 | 8/299 | 0.015532229583974648 | *ZFHX3;NFIA;NFIB;DLC1;COL12A1;WNT5A;SORBS2;LAMB1* |
| PLXNC1 | 8/299 | 0.015532229583974648 | *ZFHX3;CDK6;NFIA;DAPK1;DLC1;NEDD4L;SLC7A11;AFF3* |
| POU1F1 | 8/299 | 0.015532229583974648 | *THRB;NFIA;DLC1;RARB;ETV1;PPARG;ESR1;EPHA3* |
| POU2F3 | 8/299 | 0.015532229583974648 | *BCL11A;NFIB;DLC1;PPARG;SLC7A11;CLDN1;ESR1;NR3C2* |
| PROP1 | 8/299 | 0.015532229583974648 | *ZFHX3;NFIA;DLC1;ETV1;PPARG;LHFPL3;SLC7A11;ESR1* |
| RBPJ | 8/299 | 0.015532229583974648 | *ZFHX3;CDK6;NFIA;TBL1XR1;NFIB;DLC1;NEDD4L;LPP* |
| SIX1 | 8/299 | 0.015532229583974648 | *NFIA;NFIB;ALDH1A2;DLC1;COL12A1;WNT5A;PPARG;SLC7A11* |
| SKIL | 8/299 | 0.015532229583974648 | *KLF7;CDK6;TBL1XR1;NFIB;DLC1;NEDD4L;SLC7A11;LPP* |
| SMAD6 | 8/299 | 0.015532229583974648 | *ZFHX3;CDK6;NFIA;NFIB;DLC1;WNT5A;PPARG;FBLN2* |
| SOHLH2 | 8/299 | 0.015532229583974648 | *NFIA;BCL11A;DLC1;PPARG;SORBS2;SLC7A11;AFF3;TTN* |
| SOX12 | 8/299 | 0.015532229583974648 | *ZFHX3;CDK6;NFIA;BCL11A;NFIB;ZMYND8;DLC1;SLC7A11* |
| SOX30 | 8/299 | 0.015532229583974648 | *ZFHX3;BCL11A;CHL1;ALDH1A2;DLC1;PPARG;LHFPL3;CACNA1C* |
| SOX9 | 8/299 | 0.015532229583974648 | *NFIA;NFIB;DLC1;COL12A1;WNT5A;PPARG;SLC7A11;CLDN1* |
| TAL1 | 8/299 | 0.015532229583974648 | *ANGPT1;NFIA;BCL11A;NFIB;ALDH1A2;DLC1;PPARG;ESR1* |
| TBX3 | 8/299 | 0.015532229583974648 | *ZFHX3;NFIA;NFIB;DLC1;COL12A1;WNT5A;NEDD4L;PPARG* |
| THAP2 | 8/299 | 0.015532229583974648 | *KLF7;KLF12;CDK6;NFIA;BCL11A;DLC1;SLC7A11;LPP* |
| THRA | 8/299 | 0.015532229583974648 | *RERE;ZFHX3;NFIA;BCL11A;NFIB;DLC1;NEDD4L;ESR1* |
| TLX1 | 8/299 | 0.015532229583974648 | *BCL11A;EYA2;NFIB;DLC1;WNT5A;PPARG;LHFPL3;ESR1* |
| UNK | 8/299 | 0.015532229583974648 | *RERE;ZFHX3;KMT2A;TBL1XR1;NFIB;ZMYND8;NEDD4L;LPP* |
| XPA | 8/299 | 0.015532229583974648 | *ZFHX3;CDK6;NFIA;ZMYND8;DLC1;NEDD4L;PPARG;ESR1* |
| YOD1 | 8/299 | 0.015532229583974648 | *KLF7;CDK6;NFIA;TBL1XR1;NFIB;DLC1;SLC7A11;LPP* |
| ZBED4 | 8/299 | 0.015532229583974648 | *NCOA2;ZFHX3;CDK6;TBL1XR1;NFIB;DLC1;NEDD4L;ARID1B* |

| | | | |
|---|---|---|---|
| ZBTB1 | 8/299 | 0.015532229583974648 | *ZFHX3;NFIA;TBL1XR1;NFIB;ZMYND8;DLC1;BBX;LPP* |
| ZBTB2 | 8/299 | 0.015532229583974648 | *KLF7;ZFHX3;CDK6;TBL1XR1;ZMYND8;NEDD4L;SLC7A11;ARID1B* |
| ZBTB26 | 8/299 | 0.015532229583974648 | *MBTD1;NCOA2;KLF12;NFIA;BBX;SLC7A11;IKZF2;LPP* |
| ZC3H4 | 8/299 | 0.015532229583974648 | *RERE;ZFHX3;KMT2A;NFIA;TBL1XR1;NFIB;ZMYND8;ARID1B* |
| ZEB1 | 8/299 | 0.015532229583974648 | *KLF7;ZFHX3;CDK6;NFIA;NFIB;DLC1;WNT5A;LPP* |
| ZFY | 8/299 | 0.015532229583974648 | *CDK6;BCL11A;NFIB;DLC1;WNT5A;MX1;PPARG;SLC7A11* |
| ZIM3 | 8/299 | 0.015532229583974648 | *ZFHX3;DLC1;SORBS2;LHFPL3;HMCN1;RAPGEF4;TTN;HCN1* |
| ZKSCAN4 | 8/299 | 0.015532229583974648 | *RERE;ZFHX3;NFIA;BCL11A;DLC1;PELI2;SORBS2;SLC7A11* |
| ZNF155 | 8/299 | 0.015532229583974648 | *KLF7;ZNF607;DLC1;ZNF615;NEDD4L;SLC7A11;ZNF234;NR3C2* |
| ZNF160 | 8/299 | 0.015532229583974648 | *MBTD1;KLF12;ATP8B1;NFIB;BBX;NEDD4L;LAMB1;LPP* |
| ZNF182 | 8/299 | 0.015532229583974648 | *MBTD1;NFIA;NFIB;ZMYND8;DLC1;BBX;SLC7A11;LPP* |
| ZNF197 | 8/299 | 0.015532229583974648 | *RERE;ZFHX3;KLF12;KMT2A;ZNF615;BBX;VPS13B;LPP* |
| ZNF23 | 8/299 | 0.015532229583974648 | *ZFHX3;KLF12;BCL11A;NFIB;DLC1;NEDD4L;SLC7A11;NR3C2* |
| ZNF230 | 8/299 | 0.015532229583974648 | *KLF12;ZNF197;ZNF607;ZNF429;DLC1;ZNF615;SLC7A11;ZNF234* |
| ZNF26 | 8/299 | 0.015532229583974648 | *RERE;CDK6;NFIB;DLC1;ZNF615;NEDD4L;SLC7A11;MED13L* |
| ZNF268 | 8/299 | 0.015532229583974648 | *ZFHX3;KLF12;CDK6;NFIB;ZNF615;BBX;SLC7A11;LPP* |
| ZNF283 | 8/299 | 0.015532229583974648 | *CDK6;ZNF607;NFIB;ZNF429;ZNF615;CEP192;SLC7A11;ZNF234* |
| ZNF284 | 8/299 | 0.015532229583974648 | *ZNF607;ZNF429;DLC1;ZNF615;HMCN1;SRGAP3;SLC7A11;ZNF234* |
| ZNF302 | 8/299 | 0.015532229583974648 | *CDK6;NFIA;TBL1XR1;BCL11A;NFIB;ZNF615;BBX;LPP* |
| ZNF365 | 8/299 | 0.015532229583974648 | *NFIA;DLC1;PRICKLE2;NEDD4L;ETV1;SORBS2;EFNA5;CDK14* |
| ZNF366 | 8/299 | 0.015532229583974648 | *ZFHX3;NFIA;TBL1XR1;ALDH1A2;DLC1;PPARG;HMCN1;SLC7A11* |
| ZNF383 | 8/299 | 0.015532229583974648 | *KLF7;ZNF607;NFIA;ZNF429;ZNF615;SLC7A11;CLDN1;ZNF234* |
| ZNF417 | 8/299 | 0.015532229583974648 | *ZFHX3;KMT2A;ZNF429;DLC1;ZNF615;LPP;ZNF234;NR3C2* |
| ZNF425 | 8/299 | 0.015532229583974648 | *DLC1;MX1;NEDD4L;PTK2B;SORBS2;SRGAP3;SLC7A11;AFF3* |
| ZNF433 | 8/299 | 0.015532229583974648 | *ZFHX3;CDK6;ZNF429;DLC1;ZNF615;NEDD4L;PPARG;SLC7A11* |
| ZNF436 | 8/299 | 0.015532229583974648 | *TANC1;ZFHX3;NFIA;NFIB;ZMYND8;DLC1;NEDD4L;SLC7A11* |
| ZNF460 | 8/299 | 0.015532229583974648 | *KLF7;ZFHX3;CDK6;KMT2A;TBL1XR1;NFIB;SLC7A11;LPP* |
| ZNF485 | 8/299 | 0.015532229583974648 | *CDK6;ZNF607;NFIA;BCL11A;MX1;SLC7A11;CLDN1;NR3C2* |
| ZNF488 | 8/299 | 0.015532229583974648 | *AFAP1L2;DLC1;NEDD4L;ETV1;PPARG;LHFPL3;SLC7A11;CLDN1* |
| ZNF547 | 8/299 | 0.015532229583 | *ZNF607;NFIB;DLC1;ZNF615;NEDD4L;PTK2B;SLC7A11;ZNF234* |

| | | | |
|---|---|---|---|
| | | 974648 | |
| ZNF563 | 8/299 | 0.015532229583 974648 | *ZFHX3;KLF12;NFIA;NFIB;ZNF429;DLC1;SORBS2;SLC7A11* |
| ZNF564 | 8/299 | 0.015532229583 974648 | *RERE;NCOA2;ZFHX3;NFIA;DAPK1;NFIB;ZNF429;ACACB* |
| ZNF569 | 8/299 | 0.015532229583 974648 | *KLF12;CDK6;ZNF607;NFIB;DLC1;DMXL2;ZNF615;LPP* |
| ZNF587 | 8/299 | 0.015532229583 974648 | *ZFHX3;CDK6;KMT2A;BBX;LPP;ARID1B;ZNF234;MED13L* |
| ZNF616 | 8/299 | 0.015532229583 974648 | *ZFHX3;CDK6;ZNF607;DAPK1;BCL11A;DLC1;ZNF615;ZNF234* |
| ZNF629 | 8/299 | 0.015532229583 974648 | *RERE;ZFHX3;CDK6;NFIA;BCL11A;ZMYND8;DLC1;NEDD4L* |
| ZNF671 | 8/299 | 0.015532229583 974648 | *BCL11A;DLC1;MX1;ZNF615;SORBS2;SRGAP3;SLC7A11;AFF3* |
| ZNF697 | 8/299 | 0.015532229583 974648 | *KLF7;ZFHX3;CDK6;NFIA;BCL11A;DLC1;NEDD4L;SLC7A11* |
| ZNF71 | 8/299 | 0.015532229583 974648 | *CACNB1;ZFHX3;ZNF607;NFIA;NFIB;DLC1;ZNF615;ARID1B* |
| ZNF749 | 8/299 | 0.015532229583 974648 | *ZNF607;DAPK1;BCL11A;DLC1;MX1;LHFPL3;SLC7A11;ZNF234* |
| ZNF778 | 8/299 | 0.015532229583 974648 | *RERE;ZFHX3;NFIA;NFIB;ZMYND8;DLC1;SLC7A11;ARID1B* |
| ZNF839 | 8/299 | 0.015532229583 974648 | *RERE;ZFHX3;NFIA;BCL11A;ZMYND8;NEDD4L;LPP;ARID1B* |
| ZNF845 | 8/299 | 0.015532229583 974648 | *CDK6;ZNF607;ZNF429;ZNF615;BBX;NEDD4L;VPS13B;ZNF234* |
| ZSCAN5C | 8/299 | 0.015532229583 974648 | *ADGRD1;PDE1C;ALDH1A2;DLC1;VPS13B;ETV1;LPP;TTN* |
| BACH1 | 7/299 | 0.041511514779 02033 | *KLF7;ZFHX3;CDK6;DLC1;SLC7A11;LPP;MED13L* |
| CIC | 7/299 | 0.041511514779 02033 | *RERE;ZFHX3;KMT2A;NFIA;ZMYND8;ARID1B;MED13L* |
| CREBL2 | 7/299 | 0.041511514779 02033 | *ZFHX3;KLF12;NFIA;NFIB;DLC1;SLC7A11;NR3C2* |
| DBX1 | 7/299 | 0.041511514779 02033 | *ZFHX3;NFIA;BCL11A;NFIB;WNT5A;LHFPL3;EFNA5* |
| DLX3 | 7/299 | 0.041511514779 02033 | *BCL11A;DLC1;COL12A1;WNT5A;PPARG;SLC7A11;EFNA5* |
| DPRX | 7/299 | 0.041511514779 02033 | *ZFHX3;NFIA;DLC1;RARB;ETV1;PPARG;ESR1* |
| DRGX | 7/299 | 0.041511514779 02033 | *ZFHX3;NFIA;BCL11A;EYA2;PPARG;LHFPL3;NPAS3* |
| E2F5 | 7/299 | 0.041511514779 02033 | *CDK6;NFIA;BCL11A;NFIB;DLC1;ETV1;SLC7A11* |
| E2F6 | 7/299 | 0.041511514779 02033 | *ZFHX3;CDK6;NFIA;NFIB;DLC1;PPARG;ESR1* |
| EEA1 | 7/299 | 0.041511514779 02033 | *CAMK2D;CDK6;TBL1XR1;NFIB;DLC1;BBX;LPP* |
| EPAS1 | 7/299 | 0.041511514779 02033 | *NFIA;NFIB;DLC1;COL12A1;PPARG;SLC7A11;LPP* |
| ETV5 | 7/299 | 0.041511514779 02033 | *CDK6;NFIA;DAPK1;NFIB;DLC1;ETV1;SLC7A11* |
| FOXA1 | 7/299 | 0.041511514779 02033 | *ZFHX3;NFIA;ATP8B1;NFIB;DLC1;PPARG;ESR1* |
| FOXD2 | 7/299 | 0.041511514779 02033 | *ZFHX3;NFIA;BCL11A;EYA2;DLC1;PPARG;EFNA5* |
| FOXD4L1 | 7/299 | 0.041511514779 02033 | *ZFHX3;DLC1;LHFPL3;CACNA1C;AFF3;NR3C2;NPAS3* |

| | | | |
|---|---|---|---|
| FOXI1 | 7/299 | 0.04151151477902033 | *PDE1C;TRPV6;EYA2;DLC1;PPARG;CLDN1;ESR1* |
| FOXK2 | 7/299 | 0.04151151477902033 | *RERE;ZFHX3;NFIB;ZMYND8;DLC1;NEDD4L;ARID1B* |
| FOXN1 | 7/299 | 0.04151151477902033 | *EYA2;DLC1;PPARG;SLC7A11;CLDN1;FBLN2;ESR1* |
| FOXN4 | 7/299 | 0.04151151477902033 | *NFIA;BCL11A;EYA2;DLC1;PPARG;LHFPL3;EFNA5* |
| GATA2 | 7/299 | 0.04151151477902033 | *ZFHX3;CDK6;NFIB;DLC1;WNT5A;ETV1;PPARG* |
| GMEB1 | 7/299 | 0.04151151477902033 | *RERE;NCOA2;KLF7;ZFHX3;NFIA;ZMYND8;LPP* |
| GSC | 7/299 | 0.04151151477902033 | *EYA2;ALDH1A2;DLC1;WNT5A;PPARG;EFNA5;ESR1* |
| GSX2 | 7/299 | 0.04151151477902033 | *ZFHX3;NFIA;BCL11A;EYA2;NFIB;ALDH1A2;LHFPL3* |
| GZF1 | 7/299 | 0.04151151477902033 | *KLF7;ZFHX3;NFIA;TBL1XR1;ZMYND8;DLC1;SLC7A11* |
| H1FOO | 7/299 | 0.04151151477902033 | *AFAP1L2;EYA2;DLC1;PPARG;LHFPL3;SLC7A11;FBLN2* |
| HELT | 7/299 | 0.04151151477902033 | *ZFHX3;NFIA;BCL11A;DLC1;LHFPL3;EFNA5;ESR1* |
| HILS1 | 7/299 | 0.04151151477902033 | *NFIB;ZMYND8;DLC1;NEDD4L;LHFPL3;HMCN1;FBLN2* |
| HMGB4 | 7/299 | 0.04151151477902033 | *CHL1;EYA2;DLC1;PPARG;LHFPL3;HMCN1;EPHA3* |
| HOMEZ | 7/299 | 0.04151151477902033 | *ZFHX3;NFIA;NFIB;ZMYND8;DLC1;TMTC2;ETV1* |
| HOXB4 | 7/299 | 0.04151151477902033 | *ZFHX3;CDK6;NFIA;DLC1;COL12A1;WNT5A;PPARG* |
| HOXB7 | 7/299 | 0.04151151477902033 | *NFIA;NFIB;DLC1;WNT5A;PPARG;LAMB1;SLC7A11* |
| HOXB8 | 7/299 | 0.04151151477902033 | *ZFHX3;NFIA;BCL11A;DLC1;COL12A1;WNT5A;PPARG* |
| ID4 | 7/299 | 0.04151151477902033 | *NFIA;NFIB;DLC1;WNT5A;ETV1;SORBS2;SLC7A11* |
| IRF4 | 7/299 | 0.04151151477902033 | *CDK6;BCL11A;DLC1;MX1;PPARG;SLC7A11;ESR1* |
| IRX2 | 7/299 | 0.04151151477902033 | *ZFHX3;BCL11A;NFIB;DLC1;WNT5A;LHFPL3;EFNA5* |
| IRX5 | 7/299 | 0.04151151477902033 | *ZFHX3;NFIA;BCL11A;NFIB;DLC1;WNT5A;EFNA5* |
| LHX2 | 7/299 | 0.04151151477902033 | *NFIA;BCL11A;NFIB;DLC1;WNT5A;MPPED2;SLC7A11* |
| LHX5 | 7/299 | 0.04151151477902033 | *ZFHX3;BCL11A;EYA2;NFIB;DLC1;LHFPL3;EFNA5* |
| MAEL | 7/299 | 0.04151151477902033 | *ADGRD1;ADGRB3;DLC1;LHFPL3;HMCN1;SLC7A11;AFF3* |
| MAX | 7/299 | 0.04151151477902033 | *ZFHX3;CDK6;NFIA;TBL1XR1;NFIB;DLC1;ESR1* |
| MBD2 | 7/299 | 0.04151151477902033 | *NFIA;TBL1XR1;NFIB;ZMYND8;DLC1;NEDD4L;LPP* |
| MBNL3 | 7/299 | 0.04151151477902033 | *CDK6;NFIA;BCL11A;NFIB;DLC1;SLC7A11;LPP* |
| MEOX1 | 7/299 | 0.04151151477902033 | *ANGPT1;ALDH1A2;DLC1;COL12A1;PPARG;HMCN1;FBLN2* |
| MIER1 | 7/299 | 0.04151151477902033 | *RERE;CAMK2D;NFIA;TBL1XR1;NFIB;BBX;LPP* |
| MNT | 7/299 | 0.041511514779 | *RERE;KLF7;ZFHX3;NFIA;NFIB;ZMYND8;DLC1* |

| | | | |
|---|---|---|---|
| | | 02033 | |
| MZF1 | 7/299 | 0.041511514779 02033 | *ZFHX3;NFIA;BCL11A;NFIB;ZMYND8;PPARG;ESR1* |
| NEUROD4 | 7/299 | 0.041511514779 02033 | *BCL11A;CHL1;EYA2;DLC1;LHFPL3;EFNA5;EPHA3* |
| NFATC1 | 7/299 | 0.041511514779 02033 | *ZFHX3;CDK6;NFIA;BCL11A;DLC1;AFF3;LPP* |
| NFIX | 7/299 | 0.041511514779 02033 | *RERE;ZFHX3;NFIA;NFIB;DLC1;NEDD4L;LPP* |
| NFYB | 7/299 | 0.041511514779 02033 | *ZFHX3;CDK6;NFIA;TBL1XR1;BCL11A;NFIB;DLC1* |
| NOTO | 7/299 | 0.041511514779 02033 | *BCL11A;EYA2;NFIB;DLC1;PPARG;LHFPL3;ESR1* |
| OSR2 | 7/299 | 0.041511514779 02033 | *NFIA;NFIB;DLC1;COL12A1;WNT5A;PPARG;FBLN2* |
| OTOP3 | 7/299 | 0.041511514779 02033 | *ATP4A;CHL1;EYA2;DLC1;PPARG;HMCN1;ESR1* |
| OTP | 7/299 | 0.041511514779 02033 | *ZFHX3;NFIA;BCL11A;NFIB;LHFPL3;ESR1;NPAS3* |
| PAX8 | 7/299 | 0.041511514779 02033 | *CDK6;NFIB;DLC1;PPARG;LHFPL3;SLC7A11;ESR1* |
| PDX1 | 7/299 | 0.041511514779 02033 | *THRB;DLC1;WNT5A;PPARG;LHFPL3;EFNA5;ESR1* |
| PITX3 | 7/299 | 0.041511514779 02033 | *THRB;NFIA;NFIB;DLC1;PPARG;LHFPL3;ESR1* |
| PLAGL2 | 7/299 | 0.041511514779 02033 | *KLF7;ZFHX3;CDK6;KMT2A;DLC1;SLC7A11;LPP* |
| PLXNA1 | 7/299 | 0.041511514779 02033 | *ZFHX3;CDK6;DAPK1;DLC1;NEDD4L;LAMB1;SLC7A11* |
| POGK | 7/299 | 0.041511514779 02033 | *ZFHX3;CDK6;FMNL2;NFIB;DLC1;SLC7A11;LPP* |
| POU4F2 | 7/299 | 0.041511514779 02033 | *ZFHX3;BCL11A;NFIB;DLC1;WNT5A;LHFPL3;NPAS3* |
| RARA | 7/299 | 0.041511514779 02033 | *ZFHX3;CDK6;NFIA;ZMYND8;DLC1;PPARG;ESR1* |
| RGS9 | 7/299 | 0.041511514779 02033 | *GNAO1;DLC1;RARB;ETV1;LHFPL3;SLC7A11;AFF3* |
| RPA4 | 7/299 | 0.041511514779 02033 | *CDK6;MAOA;DLC1;PELI2;MX1;PPARG;SLC7A11* |
| RXRG | 7/299 | 0.041511514779 02033 | *NFIA;CHL1;DLC1;RARB;ETV1;SORBS2;ESR1* |
| SALL2 | 7/299 | 0.041511514779 02033 | *NFIA;BCL11A;NFIB;DLC1;WNT5A;ETV1;SORBS2* |
| SALL4 | 7/299 | 0.041511514779 02033 | *CECR2;ZFHX3;CDK6;DLC1;COL12A1;WNT5A;MPPED2* |
| SLC39A10 | 7/299 | 0.041511514779 02033 | *CAMK2D;CDK6;TBL1XR1;NFIB;DLC1;SLC7A11;LPP* |
| SP5 | 7/299 | 0.041511514779 02033 | *CDK6;BCL11A;ALDH1A2;DLC1;WNT5A;HMCN1;EFNA5* |
| SP7 | 7/299 | 0.041511514779 02033 | *COL24A1;MEGF10;ANGPT1;NFIA;DLC1;FBLN2;EPHA 3* |
| SRY | 7/299 | 0.041511514779 02033 | *MEGF10;NFIA;DLC1;RARB;PPARG;LHFPL3;ESR1* |
| T | 7/299 | 0.041511514779 02033 | *ZFHX3;BCL11A;EYA2;ALDH1A2;DLC1;WNT5A;LHFPL 3* |
| TFCP2 | 7/299 | 0.041511514779 02033 | *ZFHX3;NFIA;NFIB;ZMYND8;DLC1;ETV1;LPP* |
| TFDP3 | 7/299 | 0.041511514779 02033 | *ZFHX3;EYA2;DLC1;RARB;LHFPL3;PAK3;ESR1* |

| | | | |
|---|---|---|---|
| TIGD4 | 7/299 | 0.04151151477902033 | *TLR1;CACNB4;DLC1;SLC7A11;ACACB;IQCH;TTN* |
| TRIM32 | 7/299 | 0.04151151477902033 | *CDK6;BCL11A;NFIB;DLC1;NEDD4L;SORBS2;LPP* |
| TRIT1 | 7/299 | 0.04151151477902033 | *RERE;CDK6;NFIA;DAPK1;ZMYND8;DLC1;NEDD4L* |
| TWIST1 | 7/299 | 0.04151151477902033 | *ANGPT1;DLC1;COL12A1;WNT5A;PPARG;SLC7A11;FBLN2* |
| UNCX | 7/299 | 0.04151151477902033 | *ZFHX3;NFIA;BCL11A;EYA2;ALDH1A2;DLC1;LHFPL3* |
| VAX1 | 7/299 | 0.04151151477902033 | *NFIA;BCL11A;ALDH1A2;DLC1;WNT5A;LHFPL3;ESR1* |
| VEZF1 | 7/299 | 0.04151151477902033 | *KLF7;ZFHX3;KMT2A;NFIA;NFIB;BBX;LPP* |
| ZBTB33 | 7/299 | 0.04151151477902033 | *KLF12;CDK6;NFIA;TBL1XR1;NFIB;BBX;LPP* |
| ZBTB4 | 7/299 | 0.04151151477902033 | *RERE;ZFHX3;NFIA;NFIB;ZMYND8;DLC1;LPP* |
| ZBTB7A | 7/299 | 0.04151151477902033 | *RERE;ZFHX3;KMT2A;NFIA;NFIB;ZMYND8;DLC1* |
| ZEB2 | 7/299 | 0.04151151477902033 | *ZFHX3;CDK6;NFIA;BCL11A;NFIB;DLC1;LPP* |
| ZFP57 | 7/299 | 0.04151151477902033 | *GABBR1;BCL11A;NFIB;DLC1;COL12A1;WNT5A;SLC7A11* |
| ZFP91 | 7/299 | 0.04151151477902033 | *ZFHX3;CDK6;KMT2A;TBL1XR1;NFIB;BBX;LPP* |
| ZFR2 | 7/299 | 0.04151151477902033 | *GNAO1;GABBR1;BCL11A;DLC1;SORBS2;LHFPL3;CACNA1C* |
| ZKSCAN3 | 7/299 | 0.04151151477902033 | *RERE;ZFHX3;NFIA;NFIB;ZMYND8;DLC1;NEDD4L* |
| ZNF117 | 7/299 | 0.04151151477902033 | *CDK6;NFIA;NFIB;ZNF429;DLC1;SLC7A11;LPP* |
| ZNF138 | 7/299 | 0.04151151477902033 | *MBTD1;CDK6;NFIA;BCL11A;NFIB;ZNF429;NEDD4L* |
| ZNF175 | 7/299 | 0.04151151477902033 | *KLF7;KLF12;CDK6;NFIA;NFIB;ZMYND8;DLC1* |
| ZNF181 | 7/299 | 0.04151151477902033 | *KLF12;CAMK2D;NFIA;ZNF429;ZNF615;SLC7A11;ZNF234* |
| ZNF195 | 7/299 | 0.04151151477902033 | *MBTD1;CDK6;BCL11A;NFIB;ZMYND8;NEDD4L;SLC7A11* |
| ZNF217 | 7/299 | 0.04151151477902033 | *ZFHX3;CDK6;TBL1XR1;NFIB;ZMYND8;DLC1;LPP* |
| ZNF25 | 7/299 | 0.04151151477902033 | *NFIA;NFIB;DLC1;COL12A1;PRICKLE2;SORBS2;LPP* |
| ZNF256 | 7/299 | 0.04151151477902033 | *ZNF607;DAPK1;BCL11A;DLC1;ZNF615;SLC7A11;CLDN1* |
| ZNF281 | 7/299 | 0.04151151477902033 | *KLF7;CDK6;TBL1XR1;NFIB;DLC1;SLC7A11;LPP* |
| ZNF35 | 7/299 | 0.04151151477902033 | *ZFHX3;NFIA;BCL11A;NFIB;DLC1;WNT5A;SLC7A11* |
| ZNF350 | 7/299 | 0.04151151477902033 | *ZFHX3;MAOA;BCL11A;NFIB;DLC1;MX1;ZNF615* |
| ZNF354B | 7/299 | 0.04151151477902033 | *KLF7;ZFHX3;FMNL2;KMT2A;NFIB;DLC1;SLC7A11* |
| ZNF419 | 7/299 | 0.04151151477902033 | *NFIB;ZNF429;MX1;ZNF615;SLC7A11;LPP;ZNF234* |
| ZNF438 | 7/299 | 0.04151151477902033 | *KLF7;CAMK2D;CDK6;NFIA;DLC1;LPP;ZNF521* |
| ZNF439 | 7/299 | 0.041511514779 | *NFIA;BCL11A;ZNF429;DLC1;MX1;ZNF615;AFF3* |

| | | | |
|---|---|---|---|
| | | 02033 | |
| ZNF45 | 7/299 | 0.04151151477902033 | *KLF12;CDK6;ZNF607;DLC1;ZNF615;CEP192;ZNF234* |
| ZNF496 | 7/299 | 0.04151151477902033 | *RERE;ZFHX3;CDK6;BCL11A;ZMYND8;DLC1;ARID1B* |
| ZNF500 | 7/299 | 0.04151151477902033 | *RERE;CTIF;KLF7;ZFHX3;KMT2A;NFIB;LPP* |
| ZNF512B | 7/299 | 0.04151151477902033 | *CTIF;ZFHX3;CDK6;NFIA;ZMYND8;DLC1;LAMB1* |
| ZNF514 | 7/299 | 0.04151151477902033 | *MBTD1;KLF12;GABBR1;BCL11A;DLC1;BBX;SLC7A11* |
| ZNF541 | 7/299 | 0.04151151477902033 | *ADGRD1;ZFHX3;DLC1;ZNF536;LHFPL3;HMCN1;FBLN2* |
| ZNF544 | 7/299 | 0.04151151477902033 | *ZNF607;NFIA;DAPK1;BCL11A;DLC1;MX1;NEDD4L* |
| ZNF549 | 7/299 | 0.04151151477902033 | *KLF12;CDK6;MX1;SLC7A11;LPP;SHROOM4;ZNF234* |
| ZNF550 | 7/299 | 0.04151151477902033 | *KLF12;KMT2A;DLC1;ZNF615;SLC7A11;ZNF234;NR3C2* |
| ZNF561 | 7/299 | 0.04151151477902033 | *CDK6;KMT2A;TBL1XR1;NFIB;ZNF429;DLC1;LPP* |
| ZNF562 | 7/299 | 0.04151151477902033 | *ZFHX3;CDK6;KMT2A;NFIB;ZMYND8;BBX;LPP* |
| ZNF638 | 7/299 | 0.04151151477902033 | *KMT2A;TBL1XR1;NFIB;ZMYND8;UBR5;BBX;LPP* |
| ZNF649 | 7/299 | 0.04151151477902033 | *CECR2;CDK6;BCL11A;DLC1;MX1;ZNF615;SLC7A11* |
| ZNF681 | 7/299 | 0.04151151477902033 | *KLF12;CDK6;ZNF607;BCL11A;ZNF429;DLC1;ZNF665* |
| ZNF682 | 7/299 | 0.04151151477902033 | *MBTD1;ZNF607;BCL11A;NFIB;DLC1;MX1;SHROOM4* |
| ZNF705F | 7/299 | 0.04151151477902033 | *PHLPP1;FMNL2;ZNF607;NFIA;ZMYND8;SHROOM3;SRGAP3* |
| ZNF720 | 7/299 | 0.04151151477902033 | *KLF12;KMT2A;BCL11A;DLC1;BBX;VPS13B;APBB2* |
| ZNF77 | 7/299 | 0.04151151477902033 | *ZFHX3;NFIA;BCL11A;NFIB;DLC1;MX1;SLC7A11* |
| ZNF790 | 7/299 | 0.04151151477902033 | *ZNF607;ZNF429;DLC1;ZNF615;GDPD1;ZNF665;ZNF234* |
| ZNF800 | 7/299 | 0.04151151477902033 | *ZFHX3;CAMK2D;CDK6;KMT2A;TBL1XR1;BBX;LPP* |
| ZNF846 | 7/299 | 0.04151151477902033 | *NFIA;NFIB;DLC1;PTK2B;ETV1;HMCN1;SLC7A11* |
| ZNF90 | 7/299 | 0.04151151477902033 | *CECR2;BCL11A;ZNF429;DLC1;MX1;LHFPL3;SLC7A11* |
| ZSCAN29 | 7/299 | 0.04151151477902033 | *KLF7;ZFHX3;CDK6;DLC1;BBX;LPP;ARID1B* |
